# Unlocking a Nitrosuccinate Lyase for Decarboxylative Enzymatic Hydronitration

**DOI:** 10.1002/anie.7821033

**Published:** 2026-05-28

**Authors:** Matteo Aleotti, Hannah Dreisbach, Rémi Corlay, Clara Weber, Tamara Reiter, Wael Elaily, Bastian Daniel, Klaus Zangger, Pedro A. Sánchez‐Murcia, Mélanie Hall

**Affiliations:** ^1^ Institute of Chemistry University of Graz Graz Austria; ^2^ Institute of Molecular Biosciences University of Graz Graz Austria; ^3^ BioHealth University of Graz Graz Austria; ^4^ BioTechMed‐Graz Graz Austria; ^5^ Otto‐Loewi Research Center Medical University of Graz Graz Austria

**Keywords:** biocatalysis, enzymes, hydronitration, mutagenesis, reaction mechanisms

## Abstract

Nitro compounds are central to synthetic chemistry, yet mild and selective biocatalytic routes to these motifs remain elusive. We report an enzymatic strategy for the unique decarboxylative hydronitration of fumarate, achieved by repurposing the nitrosuccinate lyase CreD from the aspartase/fumarase superfamily for the synthetic direction. CreD from *Streptomyces cremeus* and three related bacterial homologues catalyze hydronitration using sodium nitrite salt with remarkable efficiency (turnover numbers up to 102,000) and high atom economy. With an already exceptionally broad functional group tolerance across the superfamily, this feature underscores a conserved yet adaptable activity landscape. Guided by comprehensive mutagenesis and computational analysis across all functionally distinct superfamily members, we uncovered key molecular determinants that govern nucleophile selectivity and preserve the structural integrity required for active tetramer assembly. We also define a diagnostic fingerprint for predicting hydronitration activity and propose a reaction mechanism supported by extensive QM/MM simulations. These molecular insights provide a foundation for expanding biocatalytic Michael‐type additions under environmentally benign aqueous conditions.

## Graphical abstract

The nitrosuccinate lyase CreD catalyzes C–NO_2_ bond formation using nitrite in water and shows synthetic practicality with high turnover numbers up to 102,000. A combination of protein engineering and computational methods helped to reveal the mechanistic principles that underpin this unique enzymatic activity.

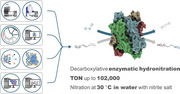

## 1 Introduction

Nitro‐containing organic molecules represent a diverse and highly relevant class of compounds in organic synthesis [1], primarily due to the strong electron‐withdrawing properties of the nitro group. This characteristic enhances their reactivity and confers them a fundamental role in synthetic chemistry [2]. Besides, nitro compounds are not only valuable precursors for amines, particularly in the chemistry of aromatics, they also find extensive applications across various industries, including pesticides, dyes, explosives, and polymer precursors, as well as in the development of prodrugs. A variety of synthetic methods have become broadly available to access nitro compounds [1, 3, 4], including nitration of alkyl halides and alkenes, and the six electron‐oxidation of amine precursors. However, these methods often require harsh conditions, exhibit poor selectivity, and involve hazardous reagents. Despite the natural occurrence of nitro compounds [5, 6], milder and efficient enzymatic methods have yet to be established. Current research has primarily focused on the nitration of aromatics using oxidative enzymes such as peroxygenases and cytochrome P450 monooxygenases. In these cases, achieving selectivity remains a challenge, and product yields are often low despite advancements in protein engineering [7, 8]. Rare cases of nitration of alkenes and alkyl chains have been identified in biosynthetic pathways. The olefin nitrating cytochrome P450 enzyme Laj2 uses NO, O_2,_ and NADPH for nitration in the final step of the biosynthesis of lajollamycin B isomers [9]. The nonheme iron‐ and α‐ketoglutarate‐dependent halogenase SyrB2 catalyzes the direct nitration of the terminal sp^3^ carbon atom of l‐2‐aminobutyrate in the presence of nitrite [10]. While halohydrin dehalogenases catalyze the ring‐opening of epoxides by nucleophilic substitution using nitrite [11], generally, oxidative pathways dominate for the formation of non‐aromatic nitro compounds, typically involving aliphatic amines or amino acids as precursors [6]. The 6‐electron oxidation of l‐aspartate to nitrosuccinate by FAD‐dependent monooxygenases is unique because the metabolic purpose of this reaction is not to introduce the nitro functionality in natural products. Instead, this reaction is part of the aspartate‐nitrosuccinate pathway (ANS) [12], whose role is to liberate nitrous acid through the combined action of the FAD‐dependent monooxygenase and a nitrosuccinate lyase. Nitrous acid plays a crucial role in the formation of naturally occurring diazo compounds [13], either as intermediates in the biosynthesis of N─N bond‐containing natural products [14] (e.g., fosfazinomycin A [15] and spinamycin [16]), or as end products (e.g., alazopeptin [17] and cremeomycin [18]). The biosynthesis of the diazoquinone cremeomycin in *S. cremeus* is one of the first reported cases involving the ANS pathway, which consists of the FAD‐dependent monooxygenase *Sc*CreE [19] and the nitrosuccinate lyase *Sc*CreD [18]. In this pathway, nitrous acid is liberated via β‐elimination from nitrosuccinate by *Sc*CreD, and is subsequently involved in the final diazotation step to cremeomycin catalyzed by the ATP‐dependent *Sc*CreM [20].

**FIGURE 1:**
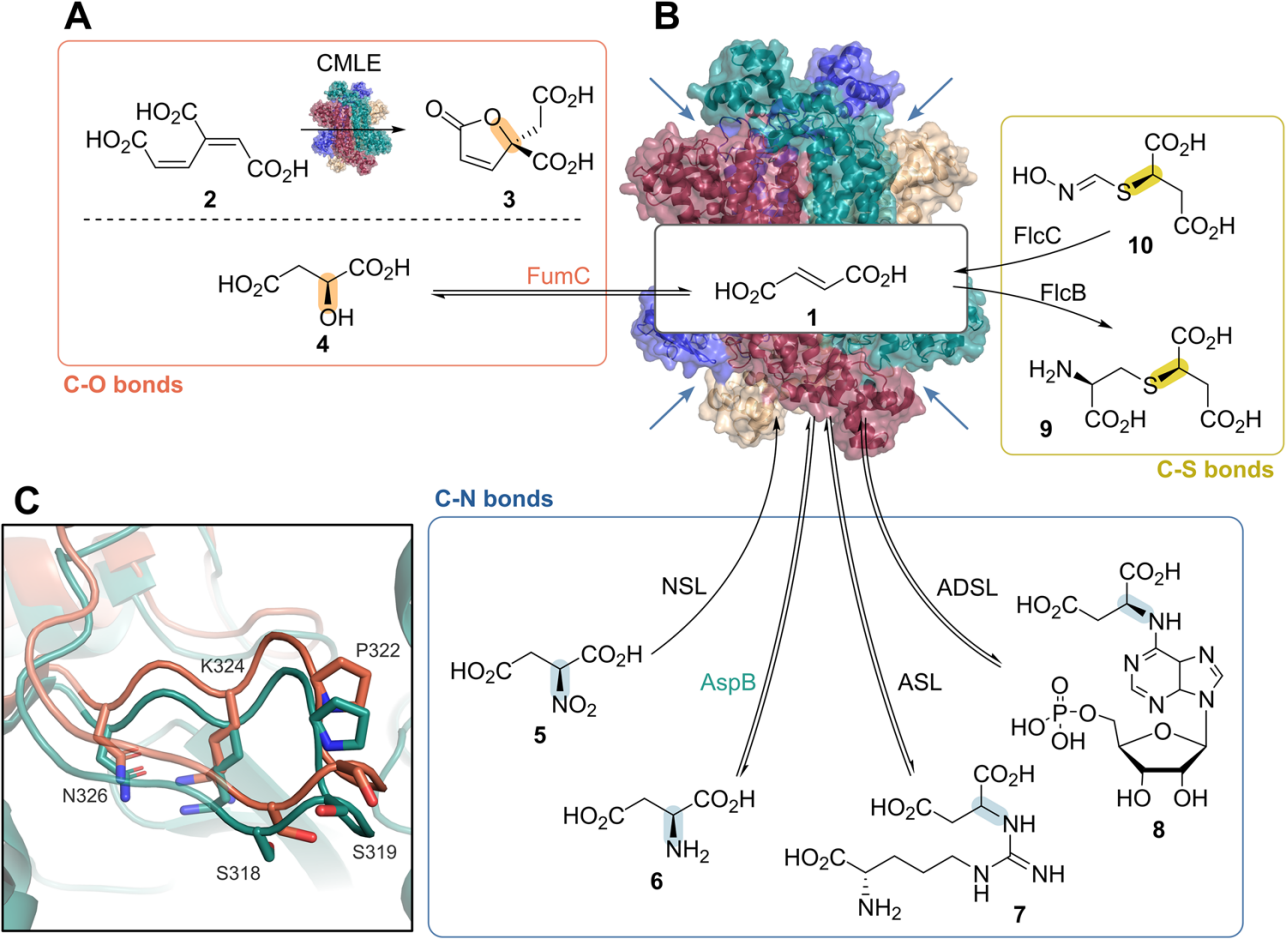
(A). Reactions catalyzed by representative members of the aspartase/fumarase superfamily via β‐elimination from succinate derivatives to fumarate, except for CMLE (3‐carboxy‐*cis*,*cis*‐muconate lactonizing enzyme), which catalyzes bond‐forming reaction. FumC: Fumarase from class II, NSL: nitrosuccinate lyase, AspB: aspartate‐ammonia lyase, ASL: argininosuccinate lyase, ADSL: adenylosuccinate lyase, FlcB and FlcC: Both homologues from *P. aeruginosa* involved in the biosynthesis of fluopsin C. (B). Conserved structure of the homotetrameric assembly within the aspartase/fumarase superfamily (PDB 3R6V). The arrows indicate the position of the four identical active sites. (C). Overlay of the crystal structures of two representative lyases *Ec*FumC (orange; PDB 6NZ9) and *B*spAspB (turquoise; PDB 3R6V) showing the highly conserved SS‐loop with characteristic GSSxxPxKxN fingerprint.

*Sc*CreD has been identified as a member of the aspartase/fumarase superfamily, a group of homologous enzymes that catalyze the elimination of several functional groups from succinate derivatives and generally cleave C─N and C─O bonds (Figure 1) [21]. The most prominent member of the family is the aspartate ammonia‐lyase (also called aspartase), which has been used for the industrial production of l‐aspartic acid since the 1970s [22]. The enzyme, which naturally catalyzes a deamination reaction, is employed in the reverse synthetic direction for the hydroamination of fumarate in the presence of ammonium. Similarly, fumarase from class II catalyzes the reversible addition of water to fumarate to form l‐malate and is used in industrial context for the large‐scale production of the latter [23]. Argininosuccinate lyase (ASL) catalyzes the reversible elimination of l‐arginine from l‐argininosuccinate [24, 25], while adenylosuccinate lyase (ADSL) liberates adenosine monophosphate (AMP) and fumarate from adenylosuccinate. The reaction is also reversible [26] and in addition, ADSL is active on 5‐aminoimidazole‐(*N*‐succinylcarboxamide) ribonucleotide in the purine biosynthetic pathway. The 3‐carboxy‐*cis*,*cis*‐muconate lactonizing enzyme (CMLE) is unique because this homologue is naturally active in an intramolecular bond‐forming reaction that converts 3‐carboxy‐*cis*,*cis*‐muconate into (*R*)‐4‐carboxymuconolactone [27]. Finally, two members of the family with activity on C─S bonds were recently identified in *Pseudomonas aeruginosa* as part of the biosynthetic pathway of the broad‐spectrum antibiotic fluopsin C: FlcB catalyzes the conjugate addition of l‐cysteine to fumarate, while FlcC cleaves 2‐(((hydroxyimino)methyl)thio)succinate into thiohydroxamate and fumarate [28]. The concerted action of these two enzymes allows for fumarate recycling in the pathway. Surprisingly, the reported stereoselectivity of FlcB/C is the opposite of that of the other members (Figure 1).

Members of the aspartase/fumarase superfamily adopt a conserved quaternary architecture, assembling as homotetramers with active sites formed at the interface of three monomers [29]. A hallmark of this superfamily is the catalytically essential SS‐loop, defined by a GSSxxPxKxN consensus sequence [21]. This flexible loop plays a central role in both substrate binding and catalysis. Across characterized family members, β‐anti‐elimination proceeds via general acid‐base catalysis: deprotonation of the β‐carbon by a conserved serine residue within the SS‐loop initiates the reaction, while protonation of the leaving group (typically mediated by a histidine) facilitates elimination. Intriguingly, this histidine is absent in *Sc*CreD, raising questions about alternative catalytic strategies. Despite extensive studies, key mechanistic features remain unresolved, including the mode of serine activation and the necessity of leaving group protonation

Motivated by the striking structural conservation within the enzyme superfamily, contrasted by their remarkable diversity in functional group tolerance, we set out to explore the catalytic potential of *Sc*CreD for synthetic purpose, inspired by the reversible nature of many of the reactions catalyzed within the superfamily. This study was driven by the motivation to develop an enzymatic nitration tool, which is presently lacking from existing synthetic methodologies. By integrating biocatalytic and kinetic analyses with protein engineering, biophysical characterization, molecular dynamics, and a systematic investigation of nucleophile preferences across the aspartase/fumarase superfamily, we uncovered previously unrecognized determinants of enzyme selectivity and report a unique decarboxylative enzymatic hydronitration reaction. These findings redefine our understanding of catalytic promiscuity within the aspartase/fumarase superfamily. They also open new avenues for functional and mechanistic exploration and provide an exciting starting point for the development of a biocatalytic hydronitration platform.

## 2 Results and Discussion

### 2.1 In vitro Bienzymatic Cascade

**FIGURE 2:**
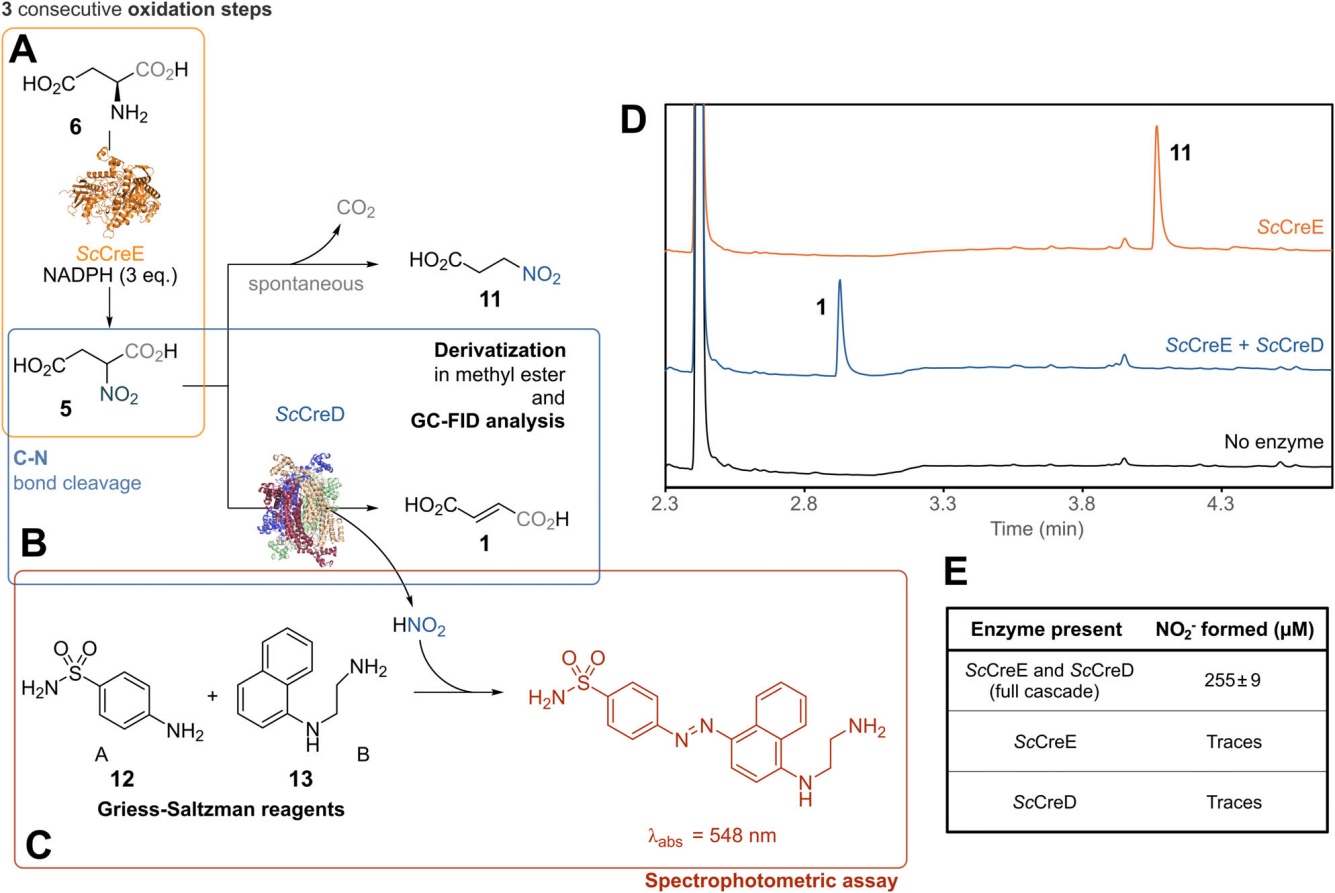
In vitro reconstitution of the ANS pathway from *S. cremeus*: enzymatic cascade catalyzed by *Sc*CreE and *Sc*CreD. (A). *Sc*CreE catalyzes the NADPH‐dependent 6‐electron oxidation of l‐aspartate to (*S*)‐nitrosuccinate, (B). which either decarboxylates spontaneously to nitropropanoate, or undergoes denitration to fumarate in the presence of the nitrosuccinate lyase *Sc*CreD (see main text). (C). The denitration releases nitrous acid, which can be monitored spectrophotometrically using the Griess‐Saltzmann reagent. (D). Both carboxylic acid products were analyzed by GC‐FID after derivatization to their methyl esters with BF_3_·MeOH (orange trace: **11** is produced in presence of *Sc*CreE, blue trace: **1** is produced in presence of *Sc*CreE and ScCreD, black trace: no product was observed in absence of enzymes). (E). Nitrous acid is detected only when both enzymes are present at the onset of the reaction. Reaction conditions: cascade performed for GC‐FID analysis (D) with 5 mM l‐aspartate, 4 eq. NADPH, 0.2 mol% *Sc*CreE, 0.2 mol% *Sc*CreD, Tris buffer (10 mM, pH 7.5), 30 °C, 120 rpm, 16 h; cascade performed for monitoring nitrous acid release (E) with 5 mM l‐aspartate, 0.1 eq. NADPH, 0.2 mol% *Sc*CreE, 0.2 mol% *Sc*CreD, 0.2 mol% ADH, isopropanol (5 vol%), Tris buffer (50 mM, pH 7.5), 1 mM MgCl_2_, 30°C, 120 rpm, 16 h. The reactions were performed in triplicates.

Both *Sc*CreE and *Sc*CreD were produced as soluble proteins by heterologous overexpression in *E. coli* and purified to homogeneity through the presence of a C‐terminal and an N‐terminal His‐tag, respectively. Their natural activity was tested in a simultaneous one‐pot cascade by combining l‐aspartate, NADPH, and both enzymes in Tris buffer at pH 7.5 and 30°C. To comply with the six‐electron oxidation requirement of *Sc*CreE, an excess of NADPH was employed. The product formation and distribution were monitored by gas chromatography after derivatization of the carboxylate groups to their methyl esters by BF_3_‐MeOH and subsequent liquid‐liquid extraction. The use of 0.2 mol% of enzyme was sufficient to detect the formation of derivatized fumarate as single product, observed only when both enzymes were present. In absence of *Sc*CreD, no fumarate was formed and a new product peak was detected, which could be identified as the derivatized nitropropanoate (Figure 2A, B, D). The full oxidation of l‐aspartate to nitrosuccinate has indeed been proposed to be accompanied by the spontaneous decarboxylation of this unstable compound [15, 19]. This instability also prevents the isolated characterization of *Sc*CreD on nitrosuccinate [30]. The concomitant formation of nitrous acid as side product was detected by spectrophotometric assay using the Griess‐Saltzman reagent (see Supporting Information, section 3.3) [18], and was observed only in the coupled *Sc*CreE/*Sc*CreD reaction (Figure 2C, E). This preliminary assay confirmed the functional activity of both *Sc*CreE and *Sc*CreD.

### 2.2 Enzymatic Hydronitration of Fumarate by *Sc*CreD

**FIGURE 3:**
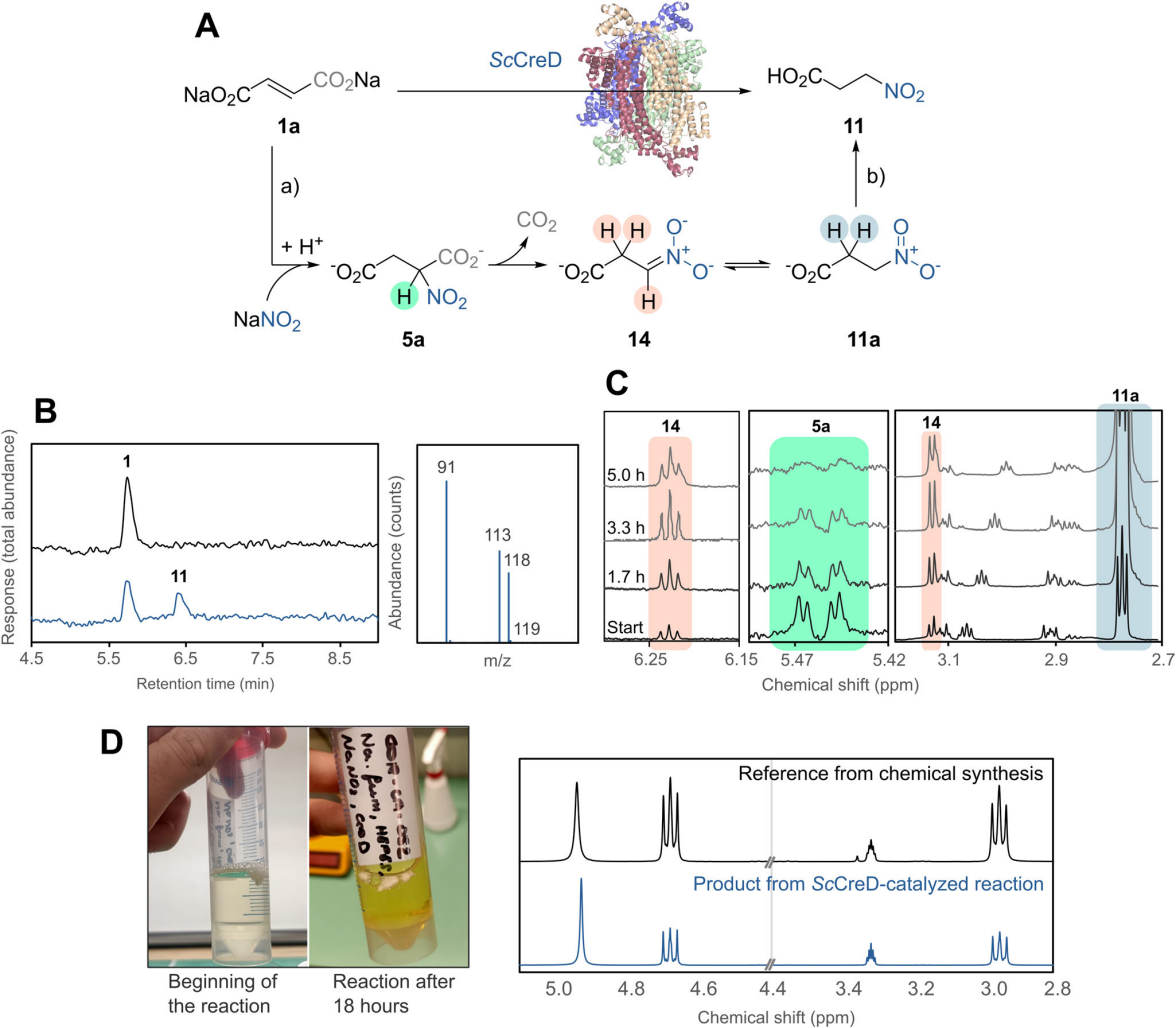
(A). Decarboxylative *Sc*CreD‐catalyzed hydronitration of fumarate **1a** to nitropropanoate **11a** via the intermediate nitrosuccinate **5a**: (a) enzymatic reaction, (b) acidic work‐up for analysis. (B). Preliminary analytical scale reaction monitored via HPLC‐MS, showing the appearance of a new peak with *m/z* of 118 (blue trace), no product was detected in absence of enzyme (black trace). Traces from total ion current (left) and extracted mass from peak at 6.5 min (right) (see Figure S4.4 for analysis of extracted ion in negative mode). (C). Time‐resolved NMR monitoring of the enzymatic reaction revealing early formation of nitrosuccinate (green), nitronate (pink) and nitropropanoate (blue) products and their evolution over time (start indicates the first data acquisition a few minutes following mixing of all components). The various regions of the NMR were separated to allow readability of the peaks, especially in the 5.45 ppm region (see Table S4.1 for relative concentration of the species over time). (D). Scale‐up of the hydronitration reaction for product isolation and characterization (left) and ^1^H‐NMR characterization (right) of the isolated reaction product (blue) and comparison with the chemically synthesized nitropropanoic acid (black). Reaction conditions: analytical scale (B) with 5 mM **1a**, 50 eq. sodium nitrite, 0.2 mol% *Sc*CreD, Tris.HCl buffer (10 mM, pH 7.0), 30 °C, 120 rpm, 16 h; Time‐resolved NMR (C) with 100 mM **1a**, 1 eq. sodium nitrite, 0.01 mol% *Sc*CreD, KPi buffer (150 mM, pH 7.0), 10 vol% D_2_O, 30°C; 0.75 mmol scale reaction for NMR analysis (D) with 50 mM **1a**, 5 eq. sodium nitrite, 0.02 mol% *Sc*CreD, HEPES buffer (100 mM, pH 7.0), 30°C, 120 rpm, 16 h.

We next explored the catalytic activity of *Sc*CreD in the reverse synthetic direction of the β‐elimination reaction by studying the hydronitration of fumarate (5 mM) in the presence of a large excess of sodium nitrite (50 eq.). This excess was anticipated to function as a thermodynamic drive by preventing the natural reaction to occur, a situation analogous to the reaction of hydroamination catalyzed by AspB, which requires large excess of ammonium salt [31]. First analysis by HPLC‐MS revealed the promising presence of a new peak with a *m*/*z* value of 118 (M‐1, Figure 3B). Carrying out the reaction on 120 mg of fumarate, which reached 44% conversion, enabled the unequivocal identification of the product as nitropropanoic acid after acidic work‐up, extraction, isolation, and NMR spectroscopy analysis (Figure 3D). This confirmed that the enzymatic hydronitration of fumarate to nitrosuccinate occurred, followed by decarboxylation. This sequence is consistent with the chemical synthesis of nitropropanoic acid from bromosuccinic acid, where spontaneous decarboxylation takes place following the nucleophilic substitution step (see Supporting Information, Section 11 and Figure S12.1). Additional experiments were performed to exclude the possibility that the acidic work‐up contributed to decarboxylation. Monitoring of the reaction by time‐resolved NMR revealed the rapid formation of nitropropanoate within a few minutes of enzymatic reaction. Minor amounts of a species tentatively assigned as nitrosuccinate were detected and confirmed by TOCSY NMR, as well as by isotopic labeling experiments in which nitrite was replaced in the enzymatic reaction with ^15^N‐labeled nitrite (see Supporting Information, Section 4.6 and Figure S12.5–S12.7). This compound, however, never accumulated beyond 0.04%. In addition, the nitronate species of nitropropanoate was detected in solution (Figure 3C), first as a transient intermediate of the decarboxylation (0.04%), and as time progresses, in equilibrium with the nitropropanoate product as its conjugated acid (*K*_eq_ ∼ 0.01, in accordance with the pH of the reaction medium and p*K*_a_ value [32], see Supporting Information, Section 4.5, Table S4.1). Careful comparison with NMR data previously reported for the *Sc*CreE‐catalyzed oxidation of l‐aspartate suggests that the intermediate then detected was not nitrosuccinate, as initially proposed, but rather the nitronate form **14** of nitropropanoate **11a** (Figure 3A) [18].

**FIGURE 4:**
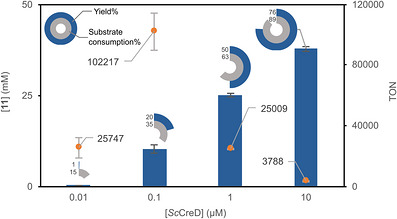
Determination of turnover number (TON) of *Sc*CreD in the decarboxylative hydronitration of fumarate at various enzyme loadings. Each blue column represents the concentration (mM) of nitropropanoic acid (**11**) reached; the orange dots give the TON calculated as [**11**]_final_/[*Sc*CreD]; the grey circles give the substrate consumption calculated based on substrate concentration obtained after the reaction [([**1**]_start_—[**1**]_final_)/ [**1**]_start_]*100; the blue circles give the reaction yield to the product. Reaction conditions: 50 mM **1a**, 10 eq. sodium nitrite, varying enzyme concentration as indicated (from 0.01 µM/0.00002 mol% to 10 µM/0.02 mol%), HEPES buffer (400 mM, pH 7.0), 30°C, 120 rpm, 18 h. The reactions were performed in triplicates.

Further improvements in the conversion were obtained by varying the buffer composition, the substrate and enzyme concentrations, and the nitrite loading. Product formation up to 38 mM could be obtained from 50 mM of fumarate (76% conversion to the product) in HEPES buffer at pH 7 and 30°C. *Sc*CreD proved to be catalytically very robust and turnover numbers (TON) up to ∼102,000 were reached (Figure 4). This is notable considering that nitropropanoic acid is a precursor to commercially important β‐alanine, which was recently obtained by enzymatic hydroamination of acrylic acid after extensive engineering of an aspartase to reach comparable TON [33]. The preparative efficiency of *Sc*CreD was counterbalanced by an unexpectedly slow reaction, in particular compared to the reported hydroamination of fumarate catalyzed by the aspartase AspB from *Bacillus* sp. YM55‐1 (*B*spAspB) and the hydration of fumarate by the fumarase FumC from *E. coli* (*Ec*FumC). *Sc*CreD turnover was 45‐ and 215‐fold slower, respectively, with a *k*_cat,app_ of 1.3 s^−1^. With its poor affinity for fumarate (*K*_m_ of 143 mM), the catalytic efficiency of *Sc*CreD in the hydronitration was three to five orders of magnitude lower than that of *B*spAspB and *Ec*FumC (Table 1). This slow reaction is surprising, as the reaction involves a spontaneous decarboxylation step, which effect was accounted for in the kinetic parameter determination (see Supporting Information, Section 4.7, Figure S4.17) and is expected to drive the overall process, while preventing the reverse reaction to occur. This likely reflects the weak nucleophilic character of nitrite and suggests fundamental differences in the catalytic strategies of the three enzymes (vide infra). Another major distinction between *Sc*CreD and aspartase and fumarase is their affinity for nitronates. The nitronate form of nitropropanoate was found to be a strong competitive inhibitor for both aspartase and fumarase, and proposed as a transition state analogue for both enzymes [32]. In contrast, while the nitronate is rapidly detected as intermediate during the reaction with *Sc*CreD and in equilibrium with nitropropanoate (Figure 3A), the enzyme does not seem to be affected and high concentration of product (up to 40 mM) is obtained. In addition, the effect of nitropropanoate on the reaction outcome was assessed by monitoring product formation in the presence of added product at the start of the reaction. No significant effect on product formation was observed over a nitropropanoate concentration range of 5–50 mM, consistent with the absence of product inhibition under these conditions (see Supporting Information, Section 4.3 and Figure S4.1).

**TABLE 1:** Kinetic parameters (*K*_m_ and *k*_cat,app_) determined for the decarboxylative *Sc*CreD‐catalyzed hydronitration of sodium fumarate^a^ and comparison with the reported values for the *B*spAspB‐catalyzed hydroamination and *Ec*FumC‐catalyzed hydration.

Entry	Enzyme	*K*_m_ ^b^ [mM]	*k*_cat_ [s^−1^]	*k*_cat_ */K*_m_ [M^−1^ s^−1^]	Nucleophile	Reference
1	*Sc*CreD	143 ± 50	1.3 ± 0.2^c^	9.2	NO_2_^−^	This work
2	*B*spAspB	1.61 ± 0.76	59 ± 17	3.6 × 10^4^	NH_3_	[34]
3	*Ec*FumC	0.15 ± 0.02	280 ± 10	1.9 × 10^6^	H_2_O	[35]

^a^ Reaction conditions: 50–550 mM of **1a**, 1 M NaNO_2_, HEPES buffer (400 mM, pH 7.0), 10 µM *Sc*CreD, 30°C, 300 rpm, 1 h.

^b^ Value for fumarate.

^c^ The value of *k*_cat,app_ is the apparent value for *k*_cat_ that takes into account hydronitration and decarboxylation. Its determination is based on the monitoring of nitropropanoic acid formation.

### 2.3 Characterization of Cross‐Activity Between Members of the Superfamily

**FIGURE 5:**
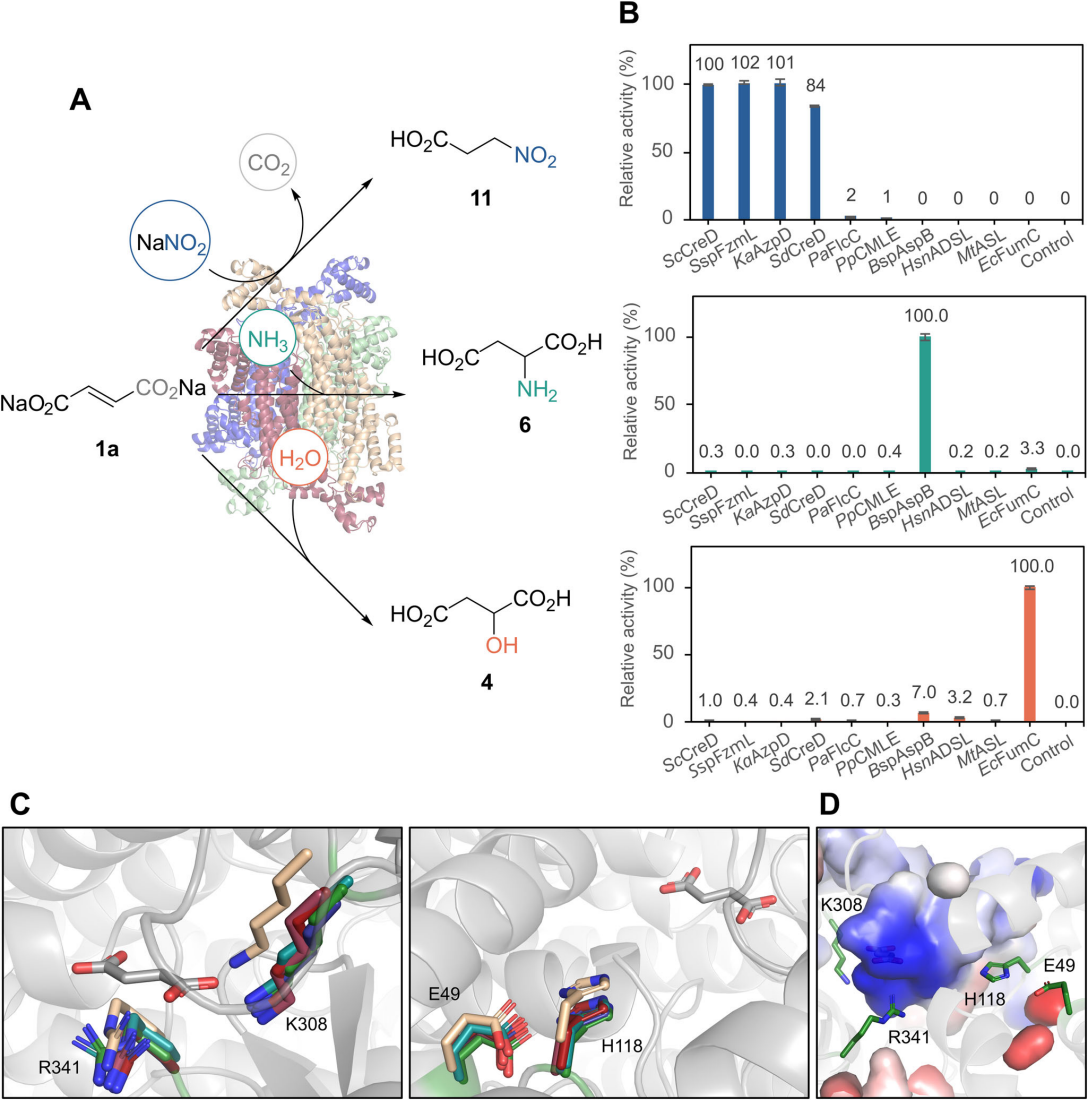
(A). Michael‐type addition reactions on fumarate with NO_2_^−^ (hydronitration), NH_3_ (hydroamination), and H_2_O (hydration) forming 3‐nitropropanoic acid, aspartic acid, and malic acid, respectively, investigated with all selected members of the aspartase/fumarase superfamily. (B). Relative activity values (based on normalized peak areas of the products obtained with each reference enzyme) of the homologues compared to the activity of the reference enzyme for each reaction (100%, *Sc*CreD for hydronitration, blue; *B*spAspB for hydroamination, green; *Ec*FumC for hydration, orange). (C). Overlay of a closed‐up view of the active site of the nitrating enzymes (fumarate shown in grey) with left: the conserved Arg and the conserved Lys, right: The conserved E/H dyad (green, *Sc*CreD; pink, *S*spFzmL; dark blue, *Ka*AzpD; beige, *Pa*FlcC; red, *Sd*CreD; turquoise, *Pp*CMLE). Noteworthy is that *Sd*CreD could not be purified to homogeneity (see Figure S2.1). The relative activity level is however in line with the corresponding purity. (D). Electrostatic potential mapping of the active site of *Sc*CreD, showing a strongly positively charged environment where fumarate binds (key residues are shown for orientation). Numbering of residues according to *Sc*CreD primary sequence. Reaction conditions: Hydronitration, 50 mM **1a**, 10 eq. NaNO_2_, 0.02 mol% enzyme, HEPES buffer (400 mM, pH 7.0), 30°C, 120 rpm, 18 h; Hydroamination, 50 mM **1a**, 60 eq. NH_3_, 0.02 mol% enzyme, pH 9.0, 30 °C, 120 rpm, 18 h; Hydration, 50 mM **1a**, HEPES buffer (400 mM, pH 7.0), 30°C, 120 rpm, 18 h. The reactions were performed in triplicates (quantification of all species available in the Supporting Information, section 5, Table S5.1).

Analysis of the sequence alignment between *Sc*CreD, *B*spAspB, and *Ec*FumC revealed a low sequence identity, likely accounting for the specific structural features necessary for the natural activity on chemically distinct substrates (nitrosucccinate, aspartate and malate, respectively). This also puts *Sc*CreD apart with only 18% and 21% identity to *B*spAspB and *Ec*FumC, respectively (see Supporting Information, Section 9, Figure S9.2). By combining BLAST search and structure similarity search with PDBeFold, we selected a set of nine homologues from the aspartase/fumarase superfamily, with a focus on those members with a reported crystal structure, evidence at protein level, and distinct substrate preference for C─N, C─O, or C─S bond. *Ka*AzpD from *Kittaspora azatica* and *S*spFzmL from *Streptomyces* sp. NRRL S‐149 have been reported to act as nitrosuccinate lyases in the ANS pathway in the context of the biosynthesis of alazopeptin [17] and fosfazinomycin [15], respectively, while *Sd*CreD is a putative nitrosuccinate lyase from *Streptomyces davaonensis* DSM 101723 with the highest sequence identity to *Sc*CreD in the UniProt Reference Clusters (66%). To complement the pool of enzymes acting on C─N bonds, and in addition to *B*spAspB, we chose the ASL from *Mycobacterium tuberculosis* ATCC 25618, *Mt*ASL [36, 37], and the ADSL from *Homo sapiens neanderthalensis*, *Hsn*ADSL [38]. The fumarase *Ec*FumC from *E. coli* has been long known for its reversible hydration activity on fumarate [39], while the 3‐carboxy‐*cis*,*cis*‐muconate lactonizing enzyme from *Pseudomonas putida* KT2440 *Pp*CMLE acts naturally on C─O bond in a unique intramolecular bond‐forming reaction [27, 40]. Finally, one of the two recently identified homologues from *P. aeruginosa* PAO1, the lyase *Pa*FlcC [28], was chosen for its ability to break C─S bonds. The nine selected candidates were overexpressed in *E. coli* as soluble proteins carrying a His‐tag and purified by immobilized metal‐affinity chromatography (see Supporting Information, Section 2, Figure S2.1). To gain insights into activity pattern, nucleophile promiscuity and cross‐activity within the superfamily, the catalytic activities of these homologues were investigated in the hydronitration, hydroamination, and hydration of fumarate (Figure 5A). Their performance was benchmarked against the reference enzymes *Sc*CreD, *B*spAspB, and *Ec*FumC, respectively, with relative activity expressed as a percentage of the activity of these reference enzymes (Figure 5B). The chosen reaction conditions allowed for maximum product formation with the reference enzymes (see Table S5.1).

The three nitrosuccinate lyases showed comparable activity to that of *Sc*CreD in the hydronitration reaction (84% to 102% relative activity). With 2% relative activity, both *Pa*FlcC and *Pp*CMLE showed catalytic promiscuity with nitrite for C─N bond formation and the ability to catalyze the hydronitration reaction with TON up to 100. This is in stark contrast to their natural chemical preference for C─S and C─O bonds, respectively. Analysis of their primary sequence showed that Arg341, identified as crucial for the denitration activity of *Sc*CreD [30], was conserved in these enzymes and absent in all inactive homologues except *Hsn*ADSL (Figures 5C, S9.3, and see section 9). Activity in the hydroamination was only identified with *Ec*FumC (3% relative activity compared to *B*spAspB), which is the enzyme the most related to *B*spAspB (40% sequence identity). This low level of activity can be partly explained by the competition with the hydration reaction, which remained predominant with *Ec*FumC under hydroamination condition (see Table S5.1). Only traces of the product aspartate were found with the other homologues. Finally, significant activity in the hydration reaction was detected with *B*spAspB and *Hsn*ADSL, with 7% and 3% relative activity, respectively, compared to *Ec*FumC. The data collectively highlight the challenge associated with activating the weak nucleophile nitrite, in comparison to ammonia and water (vide infra). Noteworthy is that, beyond the strength of the nucleophile, the reaction profile and activation energy will also vary strongly between the reactions with the three different nucleophiles, which involve distinct transition states.

### 2.4 Identification of Key Catalytic and Structurally Relevant Residues by Site‐Directed Mutagenesis

**FIGURE 6:**
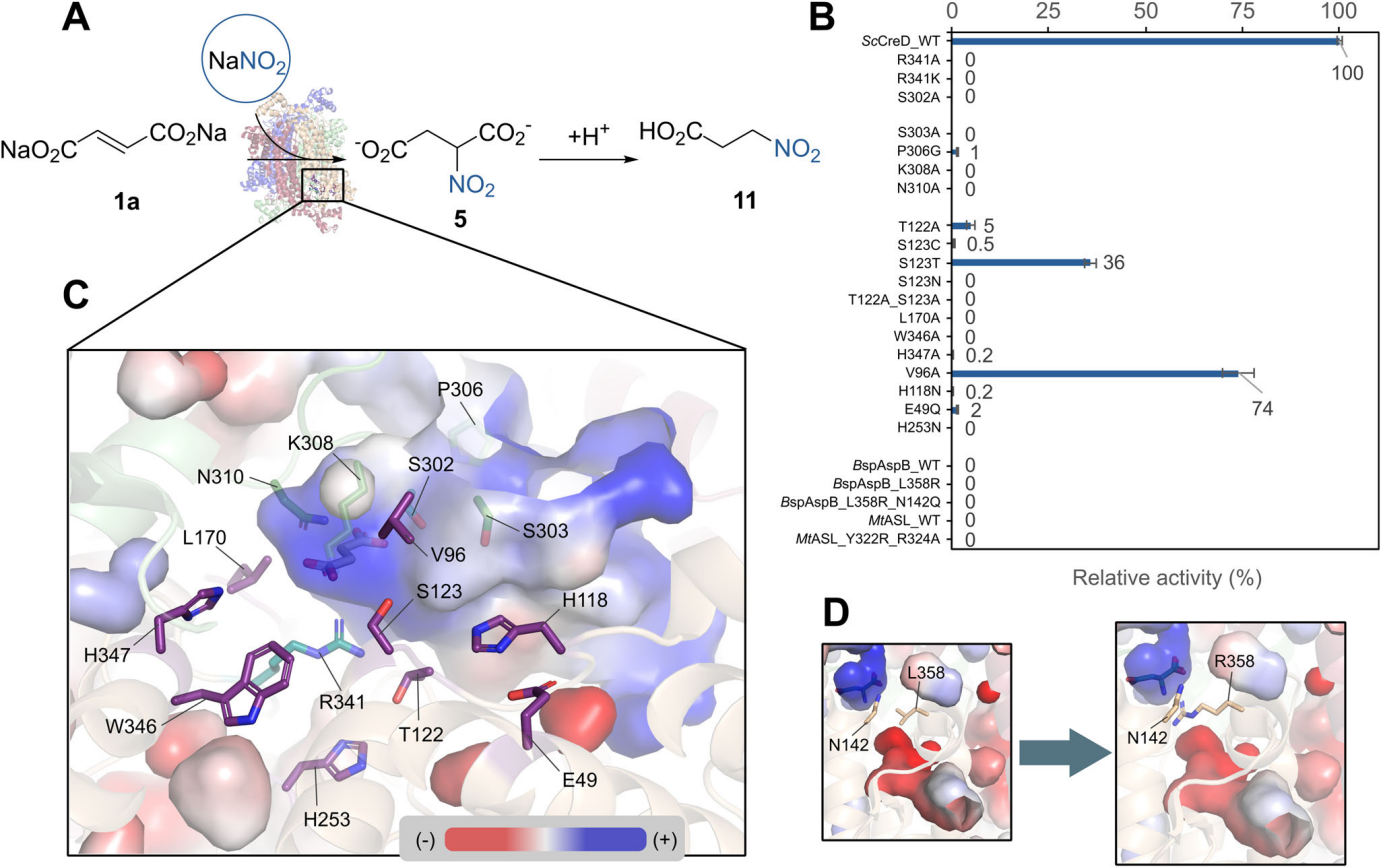
(A). Site‐directed mutagenesis study with *Sc*CreD with focus on hydronitration reaction. (B). Activity of the variants in the hydronitration. The relative activity is given as a percentage of the product concentration obtained with *Sc*CreD wild‐type. (C). View of the active site from the structure modelled using AlphaFold2 with the targeted residues highlighted (fumarate coordinates obtained from PDB 5XNZ). The amino acids were selected based on their anticipated role as catalytic residues (in blue: S302 and R341), loop residues (in green: S303, P306, K308, N310), structurally relevant residues and/or involved in binding (in dark purple: T122, S123, L170, W346, H347, V96, H118, E49, and H253). (D). Site‐directed mutagenesis study with *B*spAspB with focus on hydronitration, left: wild‐type, as seen in the crystal structure (PDB 3R6V), right: L358R (model obtained with PyMOL predictive mutagenesis tool). While the active site is positively charged, the access tunnel is negatively charged, as shown with electrostatic potential surfaces. In C and D, electrostatic potentials are given as calculated by the PyMOL built‐in function ‘Vacuum Electrostatics’. The polarities are displayed in: red (negative), white (neutral), and blue (positive). Reaction conditions: 50 mM **1a**, 10 eq. NaNO_2_, 0.02 mol% enzyme, HEPES buffer (500 mM, pH 7.0), 30 °C, 300 rpm, 18 h. The reactions were performed in triplicates.

In order to correlate substrate preference in the synthetic reaction with protein sequence and structure, a number of residues in *Sc*CreD were targeted for mutation, mostly to alanine (see Supporting Information, Section 2, Figure S2.2), and their impact on activity in the hydronitration of fumarate was evaluated (Figure 6A, B). The structural integrity of all variants was verified by size‐exclusion chromatography (SEC) and circular dichroism (CD). Most proteins were found to assemble as tetramers with no major changes observable in the secondary structures (see Supporting Information, section 8, Figures S8.1 and S8.3), so that any impact on activity could not be correlated to a misfolded protein in these cases. The crystal structure of *Sc*CreD has been determined and is available both as substrate‐free form (PDB 5XNY) and in complex with fumarate (PDB 5XNZ), however, electron density for the residues of the loop could not be observed. Therefore, for this study, a structure modelled by AlphaFold2 guided the selection of point mutations (Figure 6C). Despite a similar quaternary structure, a comparable organization of the active site, the presence of a conserved SS‐loop and key substrate binding residues, and the shared ability to selectively bind fumarate as electrophile, members of the superfamily are able to differentiate between a variety of nucleophiles, pointing at subtle structural elements involved in recognition and/or activation of the nucleophile for Michael‐type addition onto fumarate. A seminal study on *Sc*CreD identified essential residues for the natural denitration reaction [30]. They include three canonical residues of the loop, Lys308 and Asn310, which form hydrogen bonds with one of the carboxylate groups of **1a** in the so‐called α‐pocket (corresponding to the α‐carboxylate of α‐nitrosuccinate, which we now refer to as carbon C1), and Ser302, the canonical residue proposed as a general base in the superfamily that initiates the β‐elimination reaction by deprotonation of the β‐carbon of the succinate derivatives (carbon C3). The complete loss of activity of the single variants S302A, K308A, and N310A in the hydronitration reaction confirmed their equal relevance in the addition reaction. Ser303, the second conserved serine of the SS‐loop, may provide essential interactions during SS‐loop dynamics [35] and is involved, in the superfamily, in binding of the substrate β‐carboxylate (carbon C4), together with T122 and S123 [21]. While variant S303A was inactive, variant S123A could not be expressed, and instead we investigated double variant T122A_S123A (inactive), S123N (inactive), S123C, and T122A (0.5% and 5% relative activity, respectively). For the inactive T122A_S123A variant, extensive analysis of CD [41] and SEC data pointed at weakened inter‐helix coupling, affecting hydrodynamic radius, and suggesting alterations destabilizing the oligomeric state of the double variant (see Supporting Information, Section 8, Table S8.3). Variants R341A, R341K, and H253N were completely inactive, confirming the key role of Arg341 and His253 in the synthetic direction (vide infra).

Mutations of additional positions, L170A, W346A, and H347A, and the resulting loss of activity of the corresponding single variants, collectively highlight the complex network of interactions among residues and their contributions to the overall shape of the active site. Any minor change in proximity to the active site may perturb the overall arrangement, and possibly the motions of the loop, as seen with other family members [42]. Because the loop controls the opening and closing of the active site [21, 42], any geometric or electrostatic perturbation that affects the loop structural integrity will impact binding and thus activity. Exception was found for instance with Val96, which, although relatively centrally positioned, appears not relevant for catalysis. Interestingly, Leu170 is replaced by a histidine in the non‐nitrating enzymes AspB, ADSL, ASL, and FumC. This histidine residue, which is equally essential for activity and is usually part of a His‐Glu charge relay pair, was recently suggested to favor the departure of the leaving group through protonation [21, 43]. In *Sc*CreD, Leu170, as well as His347, appear instead to be involved in tetramer assembly, as biophysical analysis revealed for both L170A and H347A single variants an impacted packing and the likely formation of assemblies with an increased hydrodynamic radius. Finally, residual activity was observed with the single variants H118N and E49Q (0.2% and 2% relative activity, respectively). We identified this dyad E49/H118 only within the nitrosuccinate lyases, CMLE and *Pa*FlcC, all able to catalyze hydronitration reaction. His118 is located at the end of a positively polarized pouch leading into the active site (vide infra), with Glu49 ideally providing charge complementarity or H‐bonding partner for stabilization. Noteworthy, a strongly impaired oligomeric state or assembly was also observed for variant H118N (see Supporting Information, Section 8, Table S8.3).

### 2.5 Relevance of Tunnel Electrostatics in Determining Nucleophile Preference in the Superfamily

Detailed structural analysis revealed that the active site of *Sc*CreD is highly positively polarized (Figure 5D), a conserved feature across all homologues, which is congruent with the need to stabilize incoming dianionic substrates. Comparative mapping of putative tunnels using CAVER 3.0 [44] and their electrostatic potentials uncovered striking differences in tunnel polarity across the superfamily (see Supporting Information, section 10, Figures S10.1–S10.10). Specifically, the electrostatic character of the access tunnels from the protein surface to the active site varies between enzymes and strongly correlates with their nucleophile preferences. Enzymes catalyzing denitration/nitration reactions consistently exhibited a positively charged access tunnel, with the conserved arginine residue (Arg341 in *Sc*CreD) positioned at the entrance of the active site. In contrast, *B*spAspB displayed a negatively charged access tunnel (Figure 6D), in line with its ability to shuttle positively charged species, such as ammonium. A similar charge complementarity was observed in *Mt*ASL, which processes the cationic amino acid arginine as co‐substrate. These findings highlight the importance of electrostatic complementarity in nucleophile selection. They help explain why our efforts to engineer nitration activity into *B*spAspB by introducing only one positively charged residue at the position equivalent to Arg341 in *Sc*CreD (L358R) was unsuccessful (Figure 6B), despite the essential role of arginine for catalysis in *Sc*CreD. In *Mt*ASL, an analogous mutation (Y322R), designed to mimic *Sc*CreD, required the compensatory removal of a nearby arginine (R324A) to avoid electrostatic repulsion; nonetheless, the resulting double variant remained inactive in hydronitration (Figure 6B). While *Hsn*ADSL displays this arginine (Arg332), its positioning further away from the substrate (data not shown) likely prevents a functional role and explains the absence of hydronitration activity with this homologue. In addition, *Hsn*ADSL lacks the critical dyad E49/H118 found in all other enzymes exhibiting hydronitration activity (members of the ANS pathway as well as *Pa*FlcC and *Pp*CMLE, vide supra and Figure 5D) and we propose this E49/H118 dyad to be an additional signature motif for nitrating enzymes, in addition to D125/H253, previously suggested for nitrosuccinate lyases [30], and found in both *Pa*FlcC and *Pp*CMLE. Briefly, H253 was proposed to be stabilized as cation by D125 and to provide a key cationic environment for R341, crucial for the β‐elimination reaction. We focused on the role of H253 in the addition reaction and confirmed its involvement in product formation. Collectively, these data suggest a fingerprint E49/H118/D125/H253/R341 (*Sc*CreD numbering) characteristic of members of the aspartase/fumarase superfamily able to utilize nitrite as nucleophile. Finally, the SS‐loop appears slightly modified in the nitrating enzymes with the first Gly not necessarily directly adjacent to the two Ser. We therefore propose the sequence SSxMPxKRNP as a more accurate consensus signal for the loop of nitrating enzymes.

### 2.6 Reaction Mechanism of the Hydronitration Reaction

Several mechanistic aspects of the hydronitration reaction remain indeterminate, particularly the essential roles of Lys308, Arg341’ and Ser302. While Arg341’ and Ser302 have been proposed to function as general acid and base, respectively, in the denitration direction [30], their precise protonation states within the active site are unclear, and a consistent model for their activation and stabilization is lacking. Lys308 in turn has been proposed to establish hydrogen bonds with one of the carboxylates of the substrate (C1) in *Sc*CreD. However, the lack of electron density for the SS‐loop in the experimentally resolved structure of *Sc*CreD in complex with fumarate (PDB 5XNZ), to which S302 and K308 belong, has prevented the unambiguous understanding of their role in catalysis. Moreover, the essential involvement of these amino acids in catalyzing the reverse reaction challenges current understanding and prompts broader questions about the general mechanism of the enzymatic hydronitration reaction. To shed light on potential contributors to catalysis, we ran extensive molecular dynamics (MD) simulations with fumarate and nitrite, and with (*S*)‐nitrosuccinate, at each of the four active sites of the homotetramer of *Sc*CreD with the SS‐loop in place (see Supporting Information, section 13 and Figure S13.1–S13.2).

**FIGURE 7:**
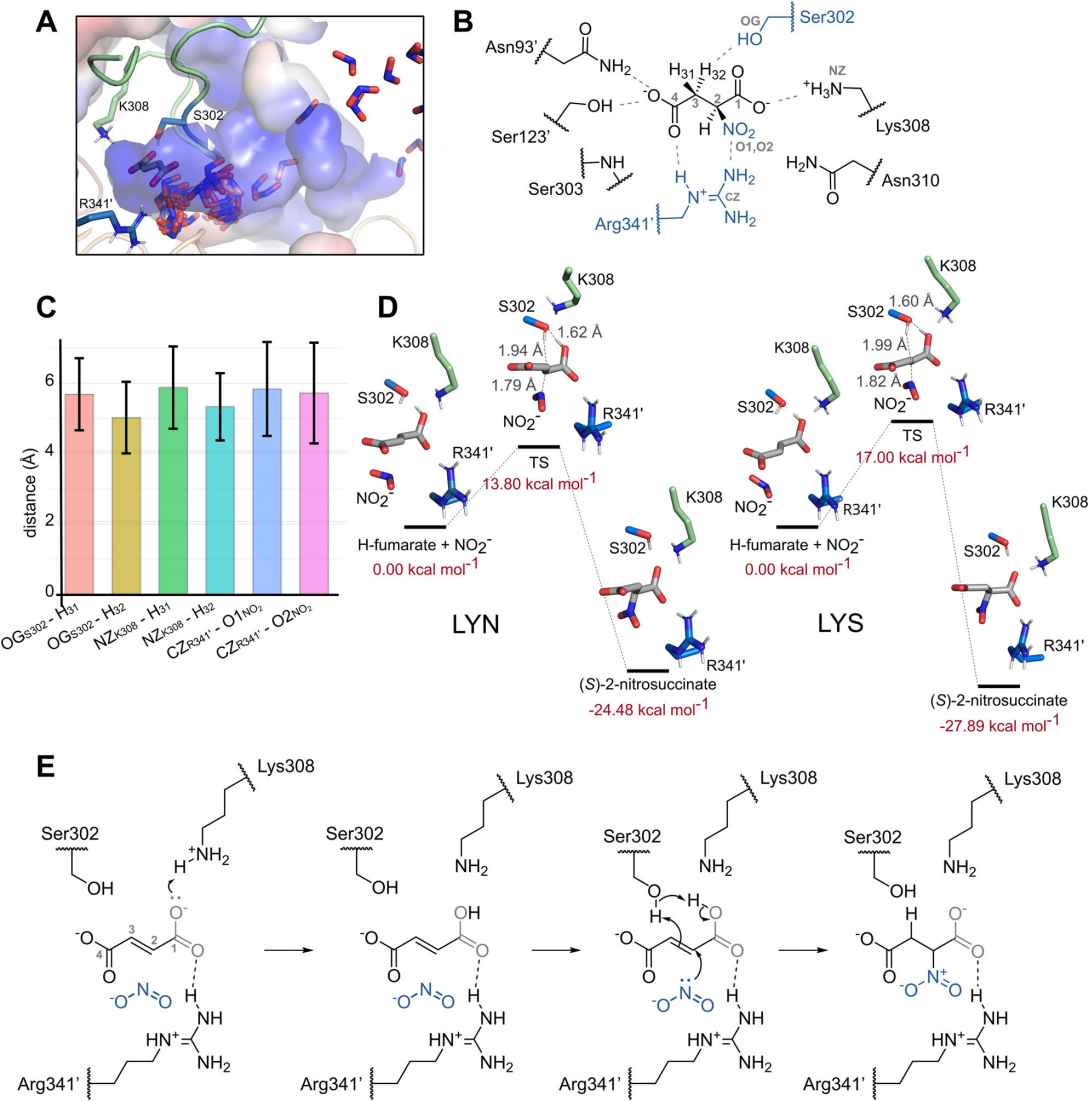
(A). View of the nitrite access tunnel of *Sc*CreD. (B). Scheme of the interactions of (*S*)‐nitrosuccinate at the active site of *Sc*CreD. Arg341’, Ser123’ and Asn93’ indicate that these three residues belong to a monomer distinct from the one bearing Ser302. (C). Distribution of selected distances (Å) along the MD simulation of *Sc*CreD in complex with (*S*)‐nitrosuccinate (in case of protonated K308). The mean values (± SD) are calculated from three independent simulations at the four active sites of *Sc*CreD. (D). Detail of the geometries and energy profile (kcal mol^−1^) of the hydronitration of hydrogen fumarate (H‐fumarate) catalyzed by *Sc*CreD (PBE0‐D3/def2‐TZVP/CPCM(water)/B3LYP‐D3/def2‐TZVP/CPCM(water)) with K308 deprotonated (LYN) or protonated (LYS). The energies contain zero‐point corrections obtained by vibrational frequency calculation. (E). Proposed mechanism of the *Sc*CreD‐catalyzed hydronitration involving proton exchange between Lys308 and the C1‐carboxylate (in grey), and attack of the nitrite onto C2 to form 2‐nitrosuccinate. Binding residues are not shown for clarity, see panel B and Figure S13.1.

Fumarate interacts via its C1‐carboxylate with K308 and N310, and occasionally, with S302. On the other hand, the C4‐carboxylate interacts with R341’ and occasionally with S123’ and S303. Nitrite, as an electronic bioisostere of the carboxylate group, was found to visit the same spatial regions as the fumarate carboxylates. Noteworthy, nitrite accesses *Sc*CreD active site via the positively charged tunnel described above, as evident from the superimposition of the snapshots from the MD simulation (Figures 7A, S13.1C), and approaches fumarate assisted by the positively charged side chain of R341’.

For (*S*)‐nitrosuccinate, one oxygen of the C1‐carboxylate group was found to interact with S302, K308, and N310, while one oxygen of the C4‐carboxylate group interacts with S123 and the second oxygen with R341’ (Figure 7B). The two oxygens of the C4‐carboxylate interact with S123 and N93, and with the backbone nitrogen of S303, respectively. In such a disposition, the hydroxyl group (OG) of the side chain of S302 is in proximity to the H32 proton on C3 of (*S*)‐nitrosuccinate (Figure 7C). This observation supports the proposed role of S302 as general base for the denitration reaction. Interestingly, the side chain of K308 was found near the C1‐carboxylate, but not in a hydrogen bonding distance to the OG of S302. Additionally, the protonation state of K308 affects the binding to the C1‐carboxylate of (*S*)‐nitrosuccinate (see Figure S13.2). Finally, the K308 NZ atom is not in proximity to the H31/H32 protons on C3 of (*S*)‐nitrosuccinate. Overall, these observations suggest that K308 may assist the activation of S302 for the (de)protonation of C3, with the C1‐carboxylate of the substrate mediating the proton exchange between these residues.

Using the information gained from the MD simulations, we explored the hydronitration of fumarate using steered QM/MM MD simulations and nudged elastic band (NEB) (see SI, sections 13.2 and 13.3). The reaction follows a classical Michael addition of nitrite onto the *Si*‐face of fumarate at C2, assisted by R341’, which also helps properly orient and activate fumarate (Figure 7D). Additionally, in such context of polarization, the C1‐carboxylate is rotated by 90° with respect to the double bond. In a first step, the C1 carboxylate is protonated by K308’. Then, upon attack of fumarate by nitrite, the negative charge accumulated in C3 attacks the proton of S302 OG. The reprotonation of S302 is assisted by the C1‐carboxylic acid. The protonation of C1‐carboxylate by K308 is in line with the suggested involvement of the substrate carboxylate as proton shuttle in the reaction catalyzed by ADSL [45]. It is also aligned with the proposed role of the conserved lysine as a base in deprotonating the catalytic serine in fumarase [46], with the major difference that in *Sc*CreD, the substrate carboxylate plays the role of a proton relay between the two residues. In the NEB profile of the hydronitration reaction, the nucleophilic attack by nitrite happens before the proton transfer from S302 to C3 (proton H32). The rate‐determining step of the reaction is the nucleophilic attack onto C2 (energy barrier of 13.80 kcal mol^−1^). If K308 stays protonated and does not participate in the protonation of C1‐carboxylate (that is nonetheless modeled as protonated species), the reaction has a larger energy barrier (17.00 kcal mol^−1^, Figure 7D). Collectively, these data support the suggested role of the C1‐carboxylate as proton shuttle during the reaction. The reaction is exergonic to form the more stable (*S*)‐nitrosuccinate, which eventually decarboxylates to yield 3‐nitropropanoate.

Overall, our mechanistic data provide for the first‐time strong evidence for the essential role of K308 in catalysis, beyond substrate binding, by protonating the C1‐carboxylate of fumarate. This step is essential to allow the reprotonation of S302 following proton transfer onto C3, after nitrite attack on C2 (Figure 7E). R341’ assists the approach of nitrite into the active site and the anchoring of fumarate for a productive binding that is compatible with nucleophile attack on the *Si*‐face.

### 2.7 Substrate Scope

**FIGURE 8:**
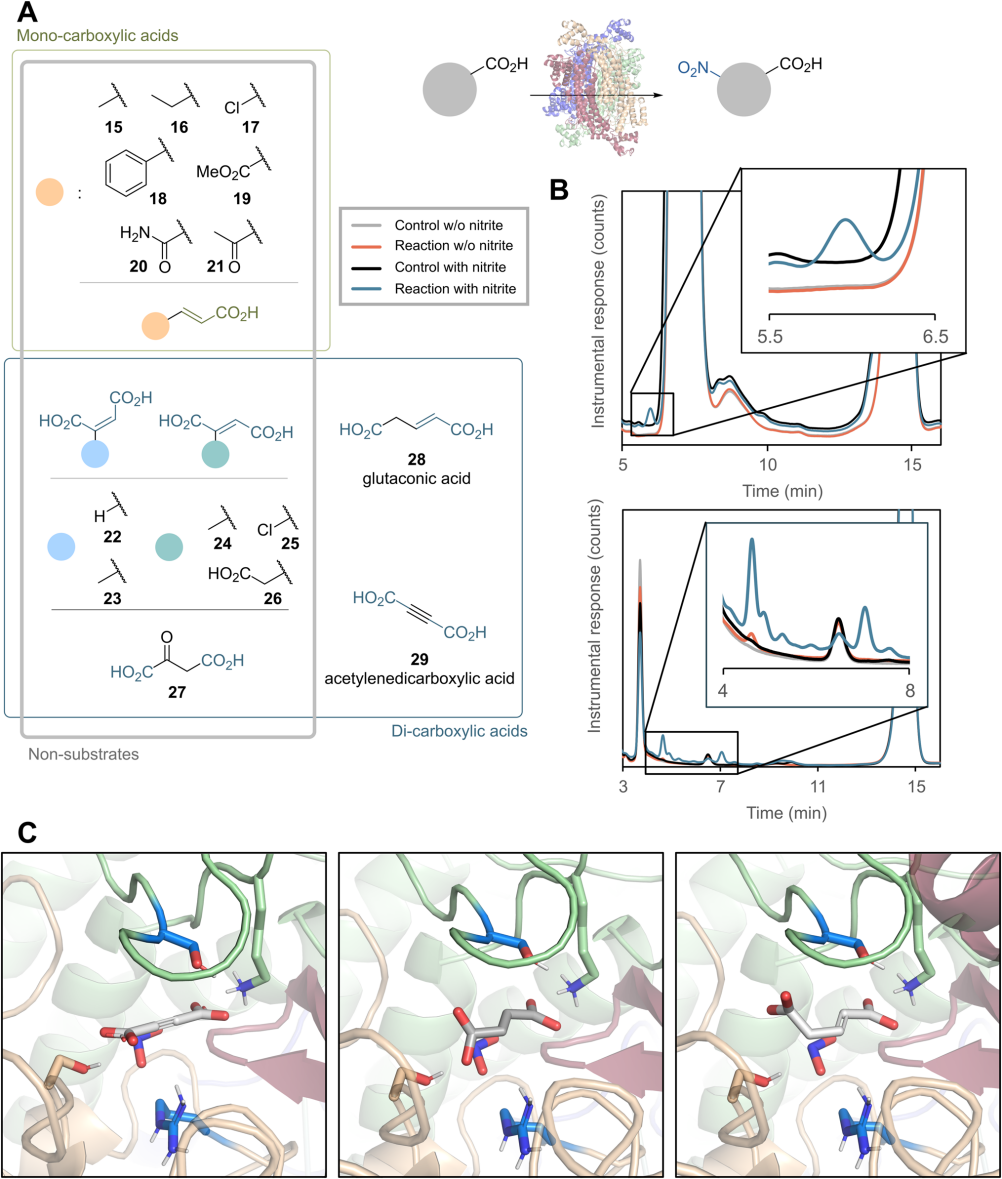
(A). Substrates tested with *Sc*CreD wild‐type. Grey box: No product formation was detected by HPLC‐UV for both mono‐carboxylic acids (green box) and di‐carboxylic acids (blue box). For glutaconic acid (**28**) and acetylenedicarboxylic acid (**29**), new products were detected. (B). HPLC‐UV chromatograms from the samples of the biotransformations of **28** (above) and **29** (below). A single product was formed with glutaconic acid (6.0 min). With acetylenedicarboxylic acid, two major peaks were formed, one formed with enzyme, but in absence of nitrite (4.7 min) and one formed with enzyme and in presence of nitrite (7.1 min). (C). Binding model of **28** (left), fumarate (middle), and **29** (right) at the active site of *Sc*CreD. **28** and **29** were manually docked to match the orientation of fumarate in its chemically active conformation used for the QM/MM calculations. Reaction conditions: 100 mM **15–27**, 5 eq. NaNO_2_, 0.05 mol% *Sc*CreD, HEPES buffer (400 mM), pH 7.0, 30 °C, 300 rpm, 18 h. 200 mM **28–29**, 2.5 eq. NaNO_2_, 0.025 mol% *Sc*CreD, HEPES buffer (400 mM), pH 7.0, 30°C, 300 rpm, 18 h (see Supporting Information, Section 7 for details).

Members of the aspartase/fumarase superfamily are distinguished by a remarkably strict electrophile selectivity, a feature that confers high fidelity, but has so far confined their synthetic application largely to fumarate. Only in rare cases has extensive protein engineering expanded this scope, as exemplified by engineered variants of *B*spAspB capable of hydroamination of non‐native substrates (e.g., crotonic acid, (*E*)‐cinnamic acid, acrylic acid) [31, 33, 47]. We therefore probed the substrate tolerance of *Sc*CreD using a panel of α,β‐unsaturated carboxylic acids and observed a complete lack of activity toward monoacid substrates (Figure 8A, section 7). Our mechanistic analysis with fumarate indicated the involvement in catalysis of the C1 carboxylate, which is bonded to the carbon being attacked by the nitrite. In addition, the C4 carboxylate contributes to stabilization of the negative charge on C3 [30] upon nucleophile attack on C2. In the case of monoacids, this cooperative role cannot be achieved, rationalizing their lack of reactivity. In contrast, a small subset of dicarboxylic acids exhibited detectable, albeit low, turnover: enzyme‐ and nitrite‐dependent substrate consumption was observed for glutaconic acid and acetylenedicarboxylic acid, accompanied by the emergence of new HPLC peaks in both cases (Figure 8B). Although product titers were insufficient for structural identification, these results establish *Sc*CreD as a promising starting point for systematic protein engineering.

To obtain a rationale for the observed substrate scope of *Sc*CreD, we superimposed the different substrates by applying the orientation of fumarate in its chemically active conformation in the active site (Figure 8C). Minor changes resulting from the addition of a single methyl group onto any of the two carbon atoms C2 and C3 of the C═C‐bond of fumarate (i.e., non‐reactive mesaconic acid **24**) impacted the critical interactions necessary for catalysis of the substrate with S302, R341’, and K308 (data not shown). In contrast, in the case of substrates like glutaconic acid (**28**) or acetylenedicarboxylic acid (**29**), no major alterations of these interactions were observed, in agreement with the detection of turnovers with both compounds (Figure 8C).

## 3 Conclusion

In summary, this work establishes that the nitrosuccinate lyase from *S. cremeus* can be harnessed to catalyze the unique decarboxylative hydronitration of fumarate, selectively forming nitropropanoate and using nitrite as a mild, water‐compatible nitrating agent. Despite its modest catalytic efficiency, the enzyme operates with high turnover numbers and substrate loadings, underscoring its robustness and suitability for process‐relevant conditions, particularly if considering the product as a key precursor to commercially important β‐alanine. Unlike conventional nitration reagents, nitrite enables this transformation under benign aqueous conditions, offering a promising foundation for an enzymatic hydronitration platform. Expanding the scope of the reaction will necessitate dedicated protein engineering efforts. This biotransformation therefore exemplifies the essential role of biocatalysts in enabling carbon‐nitrogen bond formation between inherently weak electrophiles and nucleophiles. In comparison, current chemical equivalents are limited to two protocols relying on complex catalytic systems and specialized reagents [2, 48]. Through targeted mutagenesis and computational analysis, we elucidated how electrostatic complementarity, particularly within the access tunnels, governs nucleophile compatibility within the aspartase/fumarase superfamily. The identification of multiple functionally and structurally critical residues underscores the intricate architecture of the active site in the aspartase/fumarase superfamily, and suggests that even subtle changes can impact loop dynamics and tetrameric assembly, both key determinants of catalysis. Collectively, these findings establish a comparative framework for understanding nucleophile selectivity across the aspartase/fumarase superfamily, and pave the way toward expanding its synthetic potential for carbon‐heteroatom bond formation.

## Author Contributions

**Matteo Aleotti**: conceptualization, methodology, investigation, data curation, and writing – original draft. **Hannah Dreisbach**: investigation, data curation. **Rémi Corlay**: investigation. **Clara Weber**: investigation. **Tamara Reiter**: investigation. **Wael Elaily**: investigation, data curation. **Bastian Daniel**: methodology, investigation, and data curation. **Klaus Zangger**: methodology, investigation, and data curation. **Pedro A. Sánchez‐murcia**: methodology, investigation, resources, data curation, and writing – original draft. **Mélanie Hall**: conceptualization, methodology, investigation, resources, data curation, writing – original draft, supervision, project administration, and funding acquisition.

## Conflicts of Interest

The authors declare no conflicts of interest.

## Supporting information



The authors have cited additional references within the Supporting Information [49, 50, 51, 52, 53, 54, 55, 56, 57, 58, 59, 60, 61, 62, 63, 64, 65, 66, 67, 68]. The MD simulation data of *Sc*CreD simulated in the presence of the substrates as well as the optimized geometries for the calculation of the energy barriers are available in the repository Zenodo (https://doi.org/10.5281/zenodo.18184253).**Supporting File**: anie72821‐sup‐0001‐SuppMat.docx.

## Data Availability

The data that support the findings of this study are openly available in Zenodo at https://doi.org/10.5281/zenodo.18184253.
