# Supplementary material for: Unlocking a Nitrosuccinate Lyase for Decarboxylative Enzymatic Hydronitration
Source: Angew Chem Int Ed Engl. 2026 May 28;65(31):e7821033. doi: 10.1002/anie.7821033 (PMC13411527; doi:10.1002/anie.7821033)
Supplement: Supplementary file 1 — The authors have cited additional references within the Supporting Information [49, 50, 51, 52, 53, 54, 55, 56, 57, 58, 59, 60, 61, 62, 63, 64, 65, 66, 67, 68]. The MD simulation data of ScCreD simulated in the presence of the substrates as well as the optimized geometries for the calculation of the energy barriers are available in the repository Zenodo (https://doi.org/10.5281/zenodo.18184253).Supporting File: anie72821‐sup‐0001‐SuppMat.docx. [file ANIE-65-e7821033-s001.docx]

**Supplementary Information**

**Unlocking a Nitrosuccinate Lyase for Decarboxylative Enzymatic Hydronitration**

Matteo Aleotti,^1^ Hannah Dreisbach,^1^ Rémi Corlay,^1^ Clara Weber,^1^ Tamara Reiter,^1^ Wael Elaily,^2^ Bastian Daniel,^2^ Klaus Zangger,^1,3,5^ Pedro A. Sánchez-Murcia,^4,5^ Mélanie Hall^1,3,5*^

^1^ Institute of Chemistry, University of Graz, Graz, Austria

^2^ Institute of Molecular Biosciences, University of Graz, Austria

^3^ BioHealth, University of Graz, Austria

^4^ Otto-Loewi Research Center, Medical University of Graz, Austria

^5^ BioTechMed-Graz, Graz, Austria

* Email: [melanie.hall@uni-graz.at](mailto:melanie.hall@uni-graz.at)

**Table of Contents**

[**1** **Analytical methods** 4](#_Toc226714509)

[**2** **Access to proteins** 7](#_Toc226714510)

[2.1 Overview of the enzymes 7](#_Toc226714511)

[2.2 Protein sequences 7](#_Toc226714512)

[2.3 Gene sequences 10](#_Toc226714513)

[2.4 List of mutations 15](#_Toc226714514)

[2.5 Enzyme expression of wild-type proteins and variants 15](#_Toc226714515)

[2.6 Protein purification by Immobilized Metal Affinity Chromatography 16](#_Toc226714516)

[2.7 Determination of the concentration of the proteins. 16](#_Toc226714517)

[2.8 SDS-PAGES of the protein samples 18](#_Toc226714518)

[3 *Sc*CreE/*Sc*CreD cascade 20](#_Toc226714519)

[3.1 Conversion of l-aspartic acid to fumaric acid 20](#_Toc226714520)

[3.2 Cascade setup for the analysis of HNO_2_ 20](#_Toc226714521)

[3.3 Griess test for analysis of HNO_2_^[12]^ 21](#_Toc226714522)

[3.3.1 Generation of calibration curve 21](#_Toc226714523)

[3.3.2 Quantification of HNO_2_ 21](#_Toc226714524)

[4 Hydronitration catalyzed by *Sc*CreD 21](#_Toc226714525)

[4.1 Protocol for the screening of the reaction conditions (pH value, buffer, and concentration of NaNO_2_) 21](#_Toc226714526)

[4.2 Protocol for the determination of enzyme turnover number (TON) 22](#_Toc226714527)

[4.3 Inhibition studies – Impact of nitropropanoate on reaction outcome 22](#_Toc226714528)

[4.4 Chromatograms 23](#_Toc226714529)

[4.4.1 Detection of the formation of nitropropanoic acid by HPLC-MS (qualitative analyses performed with **Method MS-01**) 23](#_Toc226714530)

[4.4.2 Quantification of nitropropanoic acid by HPLC-UV (analyses performed with **Method UV-01**) 28](#_Toc226714531)

[4.5 Isolation and characterization of the enzymatic reaction product 11 31](#_Toc226714532)

[4.6 Time-resolved NMR analysis of the enzymatic reaction and intermediate product identification 31](#_Toc226714533)

[4.7 Michaelis-Menten kinetics 32](#_Toc226714534)

[4.7.1 Time study 32](#_Toc226714535)

[4.7.2 Time study with varying NaNO_2_ concentrations 33](#_Toc226714536)

[4.7.3 Determination of *K*_m_ (fumarate) and *k*_cat_ of *Sc*CreD 34](#_Toc226714537)

[4.7.4 Preparation of calibration curve for **11** 35](#_Toc226714538)

[5 Scope of catalytic promiscuity in the aspartase/fumarase superfamily 35](#_Toc226714539)

[5.1 Experimental procedure – hydronitration reaction 35](#_Toc226714540)

[5.2 Preparation of the calibration curves - hydronitration reaction 35](#_Toc226714541)

[5.2.1 Sodium fumarate and 3-nitropropanoic acid 35](#_Toc226714542)

[5.2.2 Malic acid 36](#_Toc226714543)

[5.2.3 Calibration curves 36](#_Toc226714544)

[5.3 Results 37](#_Toc226714545)

[5.4 Chromatograms 38](#_Toc226714546)

[5.4.1 HPLC-UV traces (analyses performed with **Method UV-01**) 38](#_Toc226714547)

[5.4.2 HPLC-MS traces (analyses performed with **Method MS-02**) 44](#_Toc226714548)

[5.5 Experimental procedure - hydroamination reaction 50](#_Toc226714549)

[5.6 Preparation of the calibration curves – hydroamination reaction 50](#_Toc226714550)

[5.6.1 Sodium fumarate and l-aspartic acid 50](#_Toc226714551)

[5.6.2 Malic acid 50](#_Toc226714552)

[5.6.3 Calibration curves 51](#_Toc226714553)

[5.7 Chromatograms 52](#_Toc226714554)

[5.7.1 HPLC-UV traces (analyses performed with **Method UV-02**) 52](#_Toc226714555)

[5.7.2 HPLC-MS traces (analyses performed with **Method MS-03**) 63](#_Toc226714556)

[5.8 Experimental procedure - hydration reaction 66](#_Toc226714557)

[5.9 Preparation of the calibration curves 66](#_Toc226714558)

[5.9.1 Sodium fumarate and *rac-*malic acid 66](#_Toc226714559)

[5.9.2 Calibration curves 66](#_Toc226714560)

[5.10 Chromatograms 67](#_Toc226714561)

[5.10.1 HPLC-MS traces (analyses performed with **Method MS-04**) 67](#_Toc226714562)

[6 Study of the variants - Nitration reaction 73](#_Toc226714563)

[6.1 Experimental procedure – nitration reaction 73](#_Toc226714564)

[6.2 Chromatograms 74](#_Toc226714565)

[6.2.1 HPLC-UV traces (analyses performed with **Method UV-03**) 74](#_Toc226714566)

[6.2.2 HPLC-MS traces (analyses performed with Method MS-05) 83](#_Toc226714567)

[7 Substrate scope – hydronitration reaction 99](#_Toc226714568)

[7.1 Tested substrates 99](#_Toc226714569)

[7.2 Experimental procedure – substrate scope 99](#_Toc226714570)

[7.3 Substrate concentration optimization on the best candidates (28 and 29) 99](#_Toc226714571)

[7.4 HPLC-UV traces (analyses performed with Method UV-04) 101](#_Toc226714572)

[8 Structural characterization of *Sc*CreD enzyme variants 102](#_Toc226714573)

[9 Alignment of protein sequences 106](#_Toc226714574)

[10 Analysis of tunnels among the studied proteins 108](#_Toc226714575)

[10.1 Procedure for the preparation of the structures 108](#_Toc226714576)

[10.2 Analysis of tunnels and electrostatic potential surfaces 110](#_Toc226714577)

[11 Chemical synthesis of 3-nitropropanoic acid 120](#_Toc226714578)

[12 Nuclear magnetic resonance (NMR) 120](#_Toc226714579)

[13 Computational methods 126](#_Toc226714580)

[13.1 Molecular Dynamics (MD) simulations of *Sc*CreD 126](#_Toc226714581)

[13.2 Quantum mechanics/molecular mechanics (QM/MM) MD simulations of *Sc*CreD 128](#_Toc226714582)

[13.3 Nudged elastic band studies, geometry optimizations and calculation of frequencies 130](#_Toc226714583)

[14 References 130](#_Toc226714584)

# **Analytical methods**

**NMR**

NMR spectra were measured on a Bruker Avance III 300 MHz NMR spectrometer. Chemical shifts are reported in ppm relative to TMS (δ = 0.00 ppm) and the coupling constants (*J*) in Hertz (Hz). For the NMR time-resolved experiments, all NMR spectra were recorded on a Bruker Avance III 700 MHz NMR spectrometer equipped with a 5 mm TCI cryo probe at 298 K. For the 2D multiplicity-edited ^1^H-^13^C-HSQC spectra, a data matrix of 2048x128 points was recorded with 256 scans per increment and the 2D TOCSY was recorded with 16 scans for each of the 128 increments. For the 1D ^1^H and 2D TOCSY spectra, the water signal was suppressed using two rounds of excitation sculpting using 2 ms squared selective pulses on the water resonance.

**GC-FID**

GC analyses were carried out on an Agilent Technologies 7890A GC system with H2 as carrier gas and equipped with an FID-detector (FID 200R4Q, G3431-60531) and a 7693A Series Injector in combination with a 7693 Series Autosampler.

**Method GC-01 (cascade):** Agilent DB-1701. Injector temperature 250 °C, detector temperature 280 °C. Method: flow 1.5 mL/min, injection volume 1 μL, split ratio 50:1; temperature program: 100 °C, hold 0.5 min; 10 °C/min to 280 °C, hold 4 min. Retention times are reported in Table S1.1.

**HPLC-MS**

Low resolution mass spectra were recorded on an Agilent Technologies 6100 Quadrupole LC/MS detector in combination with an Agilent Technologies 1260 Infinity HPLC system, equipped with a 1260 quaternary pump (G1311B), 1260 multisampler (G7167A), and the column Phenomenex Luna 5 μM C18(2) 100 Å column (250 x 4.6 mm). Retention times are reported in Table S1.1. The methods used were as follows:

- **Method MS-01 (nitration)**: gradient CH_3_CN (+0.1 vol% formic acid)/ H_2_O (+0.1 vol% formic acid) (HPLC grade): 2:98 to 80:20 in 10 min. Flow: 1 mL/min. Isothermal 30 °C. Detection mode: total ion current.
- **Method MS-02 (nitration)**: gradient CH_3_CN (+0.1 vol% formic acid)/ H_2_O (+0.1 vol% formic acid) (HPLC grade): 2:98 to 80:20 in 10 min. Flow: 1 mL/min. Isothermal 30 °C. Detection mode: SIM (detection ions M-1, 115 fumaric acid, 133 malic acid), UV at 245 nm (internal standard). Internal standard: *cis,cis*-muconic acid (**30**).
- **Method MS-03 (amination)**: gradient CH_3_CN (+0.1 vol% formic acid)/ H_2_O (+0.1 vol% formic acid) (HPLC grade): 2:98 to 80:20 in 10 min. Flow: 1 mL/min. Isothermal 30 °C. Detection mode: SIM (detection ions M-1, 115 fumaric acid, 133 malic acid), UV at 254 nm (internal standard). Internal standard: 1-(4-hydroxy-3-methylphenyl)ethan-1-one (**31**).
- **Method MS-04 (hydration)**: gradient CH_3_CN (+0.1 vol% formic acid)/ H_2_O (+0.1 vol% formic acid) (HPLC grade): 2:98 to 80:20 in 10 min. Flow: 1 mL/min. Isothermal 30 °C. Detection mode: SIM (detection ions M-1, 115, 133), UV at 230 nm (internal standard). Internal standard: benzoic acid (**32**).
- **Method MS-05 (nitration)**: gradient CH_3_CN (+0.1 vol% formic acid)/ H_2_O (+0.1 vol% formic acid) (HPLC grade): 2:98 to 80:20 in 10 min. Flow: 1 mL/min. Isothermal 30 °C. Detection mode: SIM (detection ions M-1, 115 fumaric acid, 133 malic acid), 215 nm (detection internal standard). Internal standard: crotonic acid (**15**).

**Reversed-phase HPLC-UV**

Analyses were performed by RP-HPLC-UV using a Shimadzu HPLC system (Communication Bus Module CBM-40, Column Oven CTO-40C, Degasser DGU-405, Solvent delivery module LC-40D, Auto sampler SIL-40C, Diode Array Detector SPD-M40). Retention times reported in Table S1.1. The methods used were as follows:

- **Method UV-01** (nitration – quantification of fumaric acid (**1**) and 3-nitropropanoic acid (**11**)): Grace^TM^ Alltech^TM^ OA-1000 Organic acids column (300 x 6.5 mm, particle size 9 μm, catalog: 9046) eluting with isocratic flow of water (HPLC grade) with H_2_SO_4_ (2.5 mM, pH 2.4). Flow: 0.8 mL/min. λ_detection_ (**1** and **11**) = 215 nm; λ_detection_ (IS) = 254 nm**.** Internal standard: *cis,cis*-muconic acid (**30**)
- **Method UV-02** (amination – quantification of **1** and aspartic acid (**6**)): gradient CH_3_CN (+0.1 vol% trifluoroacetic acid)/ H_2_O (+0.1 vol% trifluoroacetic acid) (HPLC grade): 25:75 to 40:60 in 25 min. Flow: 1 mL/min. λ_detection_(derivatized-**6**) = 350 nm; λ_detection_(**1**) = 215 nm; λ_detection_(IS) = 254 nm. Internal standard: 1-(4-hydroxy-3-methylphenyl)ethan-1-one (**31**)
- **Method UV-03** (nitration – quantification of **1** and **11**): Grace^TM^ Alltech^TM^ IOA-2000 Organic Acids (150 x 6.5 mm, particle size 8 μm, catalog: 9648) eluting with isocratic flow of water (HPLC grade) with H_2_SO_4_ (2.5 mM, pH 2.4). Flow: 0.6 mL/min. Analysis time: 12 min. λ_detection_ (**1**, **11**, IS) = 215 nm**.** Internal standard: crotonic acid (**15**)
- **Method UV-04** (nitration – substrate scope): Grace^TM^ Alltech^TM^ IOA-2000 Organic Acids (150 x 6.5 mm, particle size 8 μm, catalog: 9648) eluting with isocratic flow of water (HPLC grade) with H_2_SO_4_ (2.5 mM, pH 2.4). Flow: 0.6 mL/min. Analysis time: 16 min. λ_detection_ = 215 nm. Internal standard: 3,3-dimethylacrylic acid (**33**)

Table S1.1: Analytical methods and retention times of analytes.

| Method name | Section | λ analysis | m/z analysis | Compounds analysed | Retention time (min) | Procedure for analysis | Analysis type^1^ |
| --- | --- | --- | --- | --- | --- | --- | --- |
| GC-01 | 3 | / | / | **1** | 2.9 | Derivatization to methyl esters (with BF_3_·MeOH) | Qual. |
|  |  |  |  | **11** | 4.1 |  |  |
| MS-01 | 4.1 | / | Total ion current (TIC) | **1** | 5.7 | / | Qual. |
|  |  |  |  | **11** | 6.5 |  |  |
| MS-02 | 5.2 | 245 | / | **30** (IS) | 7.3 | / | Quant. |
|  |  | / | 133 | **4** | 3.8 |  |  |
| MS-03 | 5.5 | 254 | / | **31** (IS) | 9.7 | Dilution 1:2 of UV-02 samples | Quant. |
|  |  | / | 133 | **4** | 3.8 |  |  |
| MS-04 | 5.8 | 230 | / | **32** (IS) | 9.7 | Dilution and direct analysis | Quant. |
|  |  | / | 133 | **4** | 3.7 |  |  |
|  |  | / | 115 | **1** | 5.6 |  |  |
| MS-05 | 6.1 | 215 | / | **15** (IS) | 7.6 | Direct analysis from samples UV-03 | Quant. |
|  |  |  | 133 | **4** | 3.8 |  |  |
| UV-01 | 4.2, 4.5,4.7, 5.2 | 215 | / | **1** | 8.3 | Quenching of NO_2_^-^ with urea | Quant. |
|  |  |  |  | **11** | 10.0 |  |  |
|  |  | 254 | / | **30** (IS) | 15.2 |  |  |
| UV-02 | 5.5 | 215 | / | **1** | 3.2 | Derivatization with DNFB | Quant. |
|  |  | 254 | / | **31** (IS) | 10.0 |  |  |
|  |  | 350 | / | **6** | 12.2 |  |  |
| UV-03 | 6.1 | 215 | / | **1** | 6.4 | Quenching of NO_2_^-^ with urea | Quant. |
|  |  |  |  | **11** | 7.3 |  |  |
|  |  |  |  | **15** (IS) | 11.1 |  |  |
| UV-04 | 7.4 | 215 | / | **28** | 7.3 | Quenching of NO_2_^-^ with urea | Qual. |
|  |  |  |  | **29** | 2.4 |  |  |
|  |  |  |  | **33** (IS) | 14.5 |  |  |

^1^**Qual.** (qualitative) indicates that the analysis did not involve any quantification. **Quant.** (quantitative) indicates that the compounds indicated in the section “Compounds analyzed” were quantified using calibration curves.**IS**: internal standard.

# **Access to proteins**

## Overview of the enzymes

The genes encoding the wild-type proteins and the variants carrying the selected mutations were ordered from BioCat GmbH directly in the expression vector of choice (see Table S2.1) as codon-optimized sequences for *E. coli* BL21(DE3) and such that the expressed proteins include a terminal His-tag to facilitate purification.

Table S2.1: Summary table of the enzymes used in this study

| Enzyme name | Organism^1^ | Cloning vector | Uniprot code | Restriction enzymes | His-tag position | MW  (Da) |
| --- | --- | --- | --- | --- | --- | --- |
| ScCreD | *Streptomyces cremeus* | pColdII | A0A0K2JL82 | NdeI  BamHI | N-terminus | 50693 |
| BspAspB | *Bacillus* sp. | pColdII | Q9LCC6 | NdeI  BamHI | N-terminus | 53062 |
| SdCreD | *Streptomyces davaonensis* | pColdII | K4R6W4 | NdeI  BamHI | N-terminus | 50611 |
| KaAzpD | *Kitasatospora azatica* | pColdII | WP_035850955.1^2^ | NdeI  BamHI | N-terminus | 50962 |
| PaFlcC | *Pseudomonas aeruginosa* | pColdII | Q9HY92 | NdeI  BamHI | N-terminus | 53660 |
| SspFzmL | *Streptomyces* sp*.* | pET-28a(+) | U5YN81 | NdeI  XhoI | N-terminus | 53091 |
| MtASL | *Mycobacterium tuberculosis* | pET-28a(+) | P9WPY7 | NdeI  XhoI | N-terminus | 51937 |
| EcFumC | *E. coli* | pET-28a(+) | P05042 | NdeI  XhoI | N-terminus | 53337 |
| HsnADSL | *Homo sapiens neanderthalensis* | pET-28a(+) | A0A384E0N4 | NdeI  XhoI | N-terminus | 57024 |
| PpCMLE | *Pseudomonas putida* | pET-28a(+) | Q88N37 | NdeI  XhoI | N-terminus | 50357 |
| ScCreE | *Streptomyces cremeus* | pET-26b(+) | A0A0K2JL70 | NdeI  BamHI | C-terminus | 73875 |

^1^Native organism of the enzyme expressed

^2^NCBI reference sequence

## Protein sequences

***Note:*** *The following protein sequences correspond to the expressed proteins, which include affinity tags (yellow) and* *short sequences from the expression vector (blue). The color scheme is consistent with that used for the corresponding DNA sequences described in Section 2.3. Amino acids highlighted in bold grey indicate native residues that were targeted for site-directed mutagenesis (See* Table S2.2*).*

***Sc*CreD^[1]^**

MNHKVHHHHHHMTRPPAPPPGAPGADELLDCGLLSPVRAGTPVEALVCDSAWLQAMLDA**E**AALTRAQARTGFLPAAAAEAITAAARADRIDLLAVARGARETANPV**V**GLVAALTAAVRRDDPAAAEYV**H**RGS**TS**QDVLDTGAMLVARRALRLIGDDLDRAADALAALAADHRDTPMAGRT**L**ALHAVPTTFGLKAAGWLELVSEAAGRVARLRDGLPFSLGGAAGTLAGYFGDRTDRGDPAVLLDRLLDAYAAETGLARPVLPW**H**VLRTPVADLAAVLAFTAGALGKIAVDVQSLARTEVAEVAEPAVEGRGA**SS**AM**P**H**K**R**N**PVLSTLIRSAALQVPALATGLTQCLVSEDE**R**SAGA**WH**AEWQPLRECLRLTGGAARTAVELAAGLEVDAARMRANLDLTDGRIVSESVAVALTPLLGRQAAKELLTRAAFTAGHEGRTLGEVLGELPELDGVLPKERWEALLDPARATGVAGALVDGALARRRPPAR

***Sd*CreD^[2]^**

MNHKVHHHHHHMTFQLSPELAAVVDSGLLSPVRAGTPVEAAVSDAAWLAAMVEAETALVRAQARLGTVPESAAAAIVEAARPERLDLVALARASRETANPVVGFVKALTAVVAAEDPAAAEYVHRGSTSQDILDTATMLVVRRAGVLIRADLDRCAAALERLARTHRATPMAGRTLTLHAVPTTFGLKAAGWLHLVTEARRRTAALAAALPVELGGAAGTLAGYLEHANNPGDDYADRLVEAYAHETGLAPATLPWHVLRTPIADTGAVCAFLAAALGKIAVDVQSLARTEVGEVTEPAVAGRGASSAMPHKRNPVLATLIRSAALQVPQHAAVLYGAMLAEDERSGGAWHAEWQPLRECLRLAGGAAHTAVELLTGLTVDADRMRANLDLTGGQIVSERVAAVLTPLLGKAQARALLTRASHEAADRGTSLAEVLSAAPEVTRHLTAAELEELLDPTRYLGAAPGLVDRAVGGPDRAVPHRSAA

***S*spFzmL^[3]^**

MGSSHHHHHHSSGLVPRGSHMDGLKTTPDRGPGTGRQAGADTGLLSPVRAGTATEAALTDSAWAQAMLDAEAALARAQSRLGTVPVKAAETITACARAEAIDLPALAVRARGAGNPVVALVEELTALVAAEDPAAAEYVHRGSTSQDILDTGAMLVTARTLRLVRGELDRTAAALARLADEHRGTPAAARTLGQHAVPTTFGLKAAGWLVAVLDARRRVAALLDGGLPVQLGGAAGTLAGYLAYAQQAPAEADGGSRPGADPGAYAGELIGLFAAETGLAEPVLPWHTLRTPISDTGAVLAHAAVALGKFATDVQTLARTELGEVAEPAGVGRGVSSAMPQKRNPVLAAMIRSAALQVPSLVLTLTHCAAAAEDERPAGAWHGEWQPLREALRLVGGAACTAAELAEGLEVHPARMEANLRLTGGAVVTERLAAVLAPVLGKAGAKRLVSRVAVGAGAGGAGGSGTADSLVDKLLADAPELAAEFTAGELARLLSPAEYTGAAEALIERALAYHRSTPGTRGTTA

***Mt*ASL^[4]^**

MGSSHHHHHHSSGLVPRGSHMSTNEGSLWGGRFAGGPSDALAALSKSTHFDWVLAPYDLTASRAHTMVLFRAGLLTEEQRDGLLAGLDSLAQDVADGSFGPLVTDEDVHAALERGLIDRVGPDLGGRLRAGRSRNDQVAALFRMWLRDAVRRVATGVLDVVGALAEQAAAHPSAIMPGKTHLQSAQPILLAHHLLAHAHPLLRDLDRIVDFDKRAAVSPYGSGALAGSSLGLDPDAIAADLGFSAAADNSVDATAARDFAAEAAFVFAMIAVDLSRLAEDIIVWSSTEFGYVTLHDSWSTGSSIMPQKKNPDIAELARGKSGRLIGNLAGLLATLKAQPLA**Y**N**R**DLQEDKEPVFDSVAQLELLLPAMAGLVASLTFNVQRMAELAPAGYTLATDLAEWLVRQGVPFRSAHEAAGAAVRAAEQRGVGLQELTDDELAAISPELTPQVREVLTIEGSVSARDCRGGTAPGRVAEQLNAIGEAAERLRRQLVR

***B*spAspB^[5]^**

MNHKVHHHHHHMNTDVRIEKDFLGEKEIPKDAYYGVQTIRATENFPITGYRIHPELIKSLGIVKKSAALANMEVGLLDKEVGQYIVKAADEVIEGKWNDQFIVDPIQGGAGTSINMNANEVIANRALELMGEEKGNYSKISPNSHVNMSQST**N**DAFPTATHIAVLSLLNQLIETTKYMQQEFMKKADEFAGVIKMGRTHLQDAVPILLGQEFEAYARVIARDIERIANTRNNLYDINMGATAVGTGLNADPEYISIVTEHLAKFSGHPLRSAQHLVDATQNTDCYTEVSSALKVCMINMSKIANDLRLMASGPRAGLSEIVLPARQPGSSIMPGKVNPVMPEVMNQVAFQVFGNDLTITSASEAGQFE**L**NVMEPVLFFNLIQSISIMTNVFKSFTENCLKGIKANEERMKEYVEKSIGIITAINPHVGYETAAKLAREAYLTGESIRELCIKYGVLTEEQLNEILNPYEMTHPGIAGRK

***Ec*FumC** **^[6]^**

MGSSHHHHHHSSGLVPRGSHMNTVRSEKDSMGAIDVPADKLWGAQTQRSLEHFRISTEKMPTSLIHALALTKRAAAKVNEDLGLLSEEKASAIRQAADEVLAGQHDDEFPLAIWQTGSGTQSNMNMNEVLANRASELLGGVRGMERKVHPNDDVNKSQSSNDVFPTAMHVAALLALRKQLIPQLKTLTQTLNEKSRAFADIVKIGRTHLQDATPLTLGQEISGWVAMLEHNLKHIEYSLPHVAELALGGTAVGTGLNTHPEYARRVADELAVITCAPFVTAPNKFEALATCDALVQAHGALKGLAASLMKIANDVRWLASGPRCGIGEISIPENEPGSSIMPGKVNPTQCEALTMLCCQVMGNDVAINMGGASGNFELNVFRPMVIHNFLQSVRLLADGMESFNKHCAVGIEPNRERINQLLNESLMLVTALNTHIGYDKAAEIAKKAHKEGLTLKAAALALGYLSEAEFDSWVRPEQMVGSMKAGRHHHHH

***Hsn*ADSL^[7]^**

MGSSHHHHHHSSGLVPRGSHMAAGGDHGSPDSYRSPLASRYASPEMCFVFSDRYKFRTWRQLWLWLAEAEQTLGLPITDEQIQEMKSNLENIDFKMAAEEEKRLRHDVMAHVHTFGHCCPKAAGIIHLGATSCYVGDNTDLIILRNALDLLLPKLARVISRLADFAKERASLPTLGFTHFQPAQLTTVGKRCCLWIQDLCMDLQNLKRVRDDLRFRGVKGTTGTQASFLQLFEGDDHKVEQLDKMVTEKAGFKRAFIITGQTYTRKVDIEVLSVLASLGASVHKICTDIRLLANLKEMEEPFEKQQIGSSAMPYKRNPMRSERCCSLARHLMTLVMDPLQTASVQWFERTLDDSANRRICLAEAFLTADTILNTLQNISEGLVVYPKVIERRIRQELPFMATENIIMAMVKAGGSRQDCHEKIRVLSQQAASVVKQEGGDNDLIERIQADAYFSPIHSQLDHLLDPSSFTGRASQQVQRFLEEEVYPLLKPYESVMKVKAELCL

***Pp*CMLE**^[8]^

MGSSHHHHHHSSGLVPRGSHMSNQLFDAYFTAPAMREIFSDRGRLQGMLDFEAALARAEASAGLVPHSAVAAIEAACQAERYDVGALANAIATAGNSAIPLVKALGKVIATGVPEAERYVHLGATSQDAMDTGLVLQLRDALDLIEADLGKLADTLSQQALKHADTPLVGRTWLQHATPVTLGMKLAGVLGALTRHRQRLQELRPRLLVLQFGGASGSLAALGSKAMPVAEALAEQLKLTLPEQPWHTQRDRLVEFASVLGLVAGSLGKFGRDISLLMQTEAGEVFEPSAPGKGGSSTMPHKRNPVGAAVLIGAATRVPGLLSTLFAAMPQEHERSLGLWHAEWETLPDICCLVSGALRQAQVIAEGMEVDAARMRRNLDLTQGLVLAEAVSIVLAQRLGRDRAHHLLEQCCQRAVAEQRHLRAVLGDEPQVSAELSGEELDRLLDPAHYLGQARVWVARAVSEHQRFTA

***Ka*AzpD^[9]^**

MNHKVHHHHHHMSGRGDTTQDAGLLSPVRAGTPVEEAVCDQAWLQAMLDAEAALARAQARLGTVPAAAARTISEAARAENFELRELALAARETANPVVGLVQALTRVVSATDPAAAEYVHRGSTSQDVFDTGAMLVARRALLIIRADLARTTTALGALAKRHRDTVMAGRTLALQAVPTTFGLKAAGWRQLLLDADRRLGQVYDHGLPVALGGAAGTLAGYLEYARLDGGERGEPDLDRYLDLDRYLDALVDAFADETGLARPVLPWHVLRTPIADLAAALAFTAGALGKIAIDVQSLTRTEVGEVTEPAVAGRGSSSAMPHKRNPVLATLIRSAALQVPAIAGALSQCLVSEDERSAGVWHAEWQLLRECLRLTGGAAHTAVELAEGLDVRTDRMFANLQLTGSQIVSERIAAVLAPHLGKVAAKQLLTDAATRSQETGDPLPTVLAATPALQGILDPAALATLCDPSAYTGAASSLVDRALR

***Pa*FlcC^[10]^**

MNHKVHHHHHHMPSHITESRIHGGAYSSPAFAAIFSDTNQVRRWLDVERALAATQAEMGIIPHEAAREIDRAAQVERFDLTQLGRESLETGHLLVPTIRALARSCEGSWGEYVHYGVTTQDILDTGLMLQVKEAWGHALGLLHSIRGHLLALALRHQHTPMVARTHGQQALPTTFGYKVAVWVDEIDRHLARFDEARERVLVGNLTGAVGTLASFGAQGFELQRRTLARLGLGAPSTSWHSARDRVLEVAGLLVQVSVTLGRVANEIYHLQRSEIDEVREGSRPGQVGSSTMPHKRNPSSVDLVSALSRLVRAQMVALTDAAFQLHERDGTAWRIEWAALPELFVYAGALLTRMEAVLAEGLEVREERMRANLDLLGGLILSERVMLALAGQFGKQTAHELLHEITQASQRQGVPFREALLGQPRLRGCFSAEALEELLDPVGYVGLAAEMVDLVADKPRTGASGDRPEGAASSQGNPGQSQYVMEDR

***Sc*CreE^[1]^**

MSVRRLTVCIVGAGPRGLSVLERFCAHERKSASHPAVTVHVVDPARPGAGRVWRTGQPRQLLMNTVASQVTVFTDGSVDMAGPVEAGPSLHEWARELAALTPVEELLGGHDDATLAEARALGADSYPTRAFYGCYLEEMFRRVVCGAPAHLEVRVHRSTAVSLADETPGSGGAQSLLLADGTRLAGLDAVVLALGHVRAEEPGAPDPRAAALGLAHFPPANPADLDLSGIAPGTPVLLRGLGLNFFDHMALFTLGRGGAFSRRPHGLRYHPSGLEPRLYAGSRRGVPYHARGENEKGVDGRHTPLLLTPERIAELTGRHREGPGLSFLRTLWPLIAREVECVYYGTLLASRGRAAERDAFVTAYLAGGDDTDRGGVLERFGIGPADRWCWERTASPHPRHGFTGPDGHRRWLLEHLAQDVRRARAGNVSDPHKAALDVLRDLRNEIRLVVDHGGLDGLSHRDDLDGWYTGLNAFLSIGPPASRIEEMAALIEAGVLDVVGPGLEVDIDEADAAFVARSPLVPGRPVRAHVLIEARLPVTDLRRTADPLLRDLLRSGQCRSYRIPAGRAPEGYETGGLEVTRRPYRLVDALGRAHPRRFAFGVPTEAVHWVTAAGARPGVNSVTLGDADAIAHAVASLTPAAAPRLPAYEDPGVRCPSDDRLTEVTAADPNSSSVDKLAAALEHHHHHH

***Lk*ADH_mut_^[11]^**

The enzyme was produced as reported previously.

## Gene sequences

***Sc*CreD**

atgaatcacaaagtgcatcatcatcatcatcatatgacccgtccgccggcacctccgcctggtgcacctggtgcagatgaactgctggattgcggtctgctgagtccggtgcgcgcaggcacccctgtggaagcactggtgtgtgatagcgcatggctgcaggcaatgctggatgcagaagccgcactgacccgcgcacaggcacgtaccggcttcctgccggccgcagctgcagaagcaattaccgcagcagcccgtgcagatcgtattgatctgctggccgtggcacgcggcgcacgtgaaaccgcaaatccggtggtgggcctggtggcagccctgacagccgctgtgcgtcgtgatgatccggccgcagcagaatatgtgcatcgtggtagcaccagtcaggatgttctggataccggcgccatgctggtggcacgccgtgcactgcgcctgattggcgatgatctggatcgtgcagcagatgcactggccgcactggccgccgatcatcgcgataccccgatggcaggtcgtaccctggccctgcatgcagttccgaccaccttcggtctgaaagccgcaggctggctggaactggttagtgaagccgcaggtcgtgttgcccgtctgcgcgatggcctgccgttcagtctgggcggcgccgcaggtaccctggcaggttacttcggcgatcgtaccgatcgcggtgatccggcagttctgctggatcgcctgctggatgcatacgctgccgaaaccggcctggcacgtccggtgctgccgtggcatgttctgcgtaccccggtggccgatctggccgctgtgctggcattcaccgcaggtgccctgggcaaaattgccgttgatgttcagagtctggcacgtaccgaagtggccgaagtggcagaaccggccgttgaaggtcgcggcgccagtagcgcaatgccgcataaacgcaatccggtgctgagtaccctgattcgcagcgccgccctgcaggtgccggcattagcaaccggtctgacccagtgcctggttagcgaagatgaacgcagtgccggtgcctggcatgccgaatggcagccgctgcgtgaatgcctgcgcctgaccggtggcgccgcaagaaccgcagtggaactggccgccggcctggaagttgatgccgcacgcatgcgtgcaaatctggatctgaccgatggtcgcattgttagtgaaagcgtggccgttgccctgaccccgctgctgggtcgtcaggctgccaaagaactgctgacccgcgcggccttcaccgccggtcatgaaggtcgtaccttaggtgaagttctgggtgaactgccggaactggatggcgttctgccgaaagaacgttgggaagcactgctggaccctgcacgcgcaaccggcgtggctggtgcactggttgatggtgccctggcacgtcgtcgcccgcctgcaagataa

***Sc*CreE**

**atg**agcgtgcgtcgtctgaccgtgtgtattgttggcgcaggcccgcgtggcctgagcgttcttgaacgcttctgtgcacatgaacgtaaaagcgcaagtcatccggcagtgaccgttcatgtggttgatccggcccgcccgggcgcaggtagagtgtggagaaccggtcagccgcgccagctgctgatgaataccgtggccagtcaggttaccgtgttcaccgatggtagtgtggatatggcaggtccggtggaagcaggcccgagcctgcatgaatgggcacgtgaactggcagccctgaccccggtggaagaactgctgggtggtcatgatgatgcaaccctggccgaagcccgtgccctgggtgctgatagctatccgacccgcgcattctatggttgctatctggaagaaatgttccgccgcgtggtgtgcggcgcaccggcacacttagaagttcgtgttcatcgcagtaccgcagtgagtctggcagatgaaacacctggtagcggtggtgcccagagtctgctgctggcagatggtacccgtctggccggtctggatgcagtggtgctggccctgggccatgtgcgtgccgaagaaccgggcgccccggaccctagagccgcagcattaggtctggcacacttcccgccggccaatccggcagatctggatctgagcggcattgcaccgggcaccccggttctgctgcgcggtcttggtctgaacttcttcgatcacatggcactgttcaccctgggtcgtggtggtgccttcagccgtcgcccgcatggtctgcgctatcatccgagcggtctggaaccgcgtctgtatgccggcagtcgtcgtggtgttccgtatcatgcccgtggcgaaaatgaaaaaggtgtggatggtcgtcataccccgctgctgctgaccccggaacgcattgccgaactgaccggtcgccatcgtgaaggtccgggcctgagcttcctgcgcaccctgtggccgctgattgcccgcgaagttgaatgtgtgtattatggcaccctgctggccagtcgtggtcgtgcagcagaacgtgatgccttcgttaccgcctatctggcaggtggtgatgataccgatcgtggcggtgttctggaacgcttcggtattggtccggcagatcgctggtgttgggaacgtaccgccagtccgcatccgcgtcatggcttcaccggcccggatggccatcgccgttggctgctggaacatctggcccaggatgtgcgccgcgcacgcgcaggtaatgttagcgatccgcataaagcagcactggatgttctgcgcgatctgcgcaatgaaattcgcctggttgttgatcatggcggcctggatggcctgagtcatcgcgatgatctggatggttggtataccggcctgaatgcattcctgagtattggcccgccggcaagccgcattgaagaaatggcagccctgattgaagcaggcgtgctggatgtggttggcccgggcctggaagtggatattgatgaagccgatgccgcattcgtggcacgcagcccgctggtgccgggtagacctgtgcgcgctcatgttctgattgaagcccgtctgccggttaccgatctgcgccgtaccgcagatccgctgctgcgcgatcttctgcgcagtggtcagtgtcgtagctatcgtattccggcaggccgtgcaccggaaggctatgaaaccggtggtctggaagttacccgtcgtccgtatcgcctggttgatgccctgggccgtgcccatccgcgcagattcgcattcggtgttccgaccgaagcagttcattgggttaccgcagcaggtgcccgtccgggtgttaatagtgttaccctgggtgatgcagatgcaattgcccatgccgttgcaagcctgaccccggcagcagcaccgcgtctgcctgcttatgaagatccgggtgttcgctgtccgagcgatgatcgcctgaccgaagttaccgccgcggatccgaattcgagctccgtcgacaagcttgcggccgcactcgagcaccaccaccaccaccactga

***S*spFzmL**

atgggcagcagccatcatcatcatcatcacagcagcggcctggtgccgcgcggcagccatatggatggcctgaaaaccaccccggatcgtggcccgggcaccggtagacaggcaggtgctgataccggtctgctgagcccggttcgcgccggtaccgctaccgaagcagcactgaccgatagcgcatgggcacaggcaatgctggatgcagaagccgcactggcacgtgcccagagtcgtctgggcaccgtgccggttaaagccgccgaaaccattaccgcctgtgcccgcgcagaagcaattgatctgccggcactggcagtgcgcgcacgtggtgcaggcaatccggtggtggccctggttgaagaactgaccgccctggtggccgcagaagatccggccgctgccgaatatgttcatcgcggcagtaccagtcaggatattctggataccggcgccatgctggtgaccgcacgtaccctgcgcctggttcgtggcgaactggatcgtaccgcagcagccctggcacgcctggctgatgaacatcgtggtaccccggccgcagcccgtaccttaggtcagcatgccgttccgaccacctttggtctgaaagcagccggttggctggttgcagttctggatgcccgtcgccgtgtggcagcactgctggatggcggtctgccggtgcagctgggtggcgcagctggtaccctggccggttatctggcatacgcacagcaggccccggcagaagccgatggtggcagccgtccgggcgcagatccgggtgcatacgcaggcgaactgattggcctgtttgccgccgaaacaggcctggccgaaccggtgctgccgtggcataccctgcgtaccccgattagtgataccggtgcagttctggcccatgccgcagtggcactgggcaaatttgcaaccgatgttcagaccctggcacgtaccgaactgggcgaagttgccgaaccggccggtgttggtcgtggcgtgagcagtgccatgccgcagaaacgcaatccggttctggccgcaatgattcgtagtgccgccctgcaggtgccgagtctggttctgaccctgacccattgtgcagccgccgccgaagatgaacgtccggccggtgcctggcatggtgaatggcagccgctgcgcgaagcactgcgtctggtgggcggtgcagcctgcaccgctgctgaactggccgaaggtctggaagttcatccggcacgtatggaagccaatctgcgcctgaccggcggtgccgttgttaccgaacgtctggcagccgtgctggcaccggtgctgggtaaagcaggtgccaaacgtctggtgagccgtgttgcagttggcgccggtgccggtggtgccggtggcagcggtacagctgatagcctggttgataaactgctggcagatgccccggaactggccgccgaattcaccgccggcgaactggcccgcctgctgtcacctgccgaatataccggcgcagccgaagccctgattgaacgtgcactggcctatcatcgtagtaccccgggcacccgtggtaccaccgcctaa

***Mt*ASL**

atgggcagcagccatcatcatcatcatcacagcagcggcctggtgccgcgcggcagccatatgagcaccaatgaaggcagcctgtggggtggccgctttgccggtggccctagcgatgcactggcagcactgagcaaaagcacccattttgattgggtgctggccccgtatgatctgaccgcaagtcgcgcacataccatggtgctgtttcgcgcaggtctgctgaccgaagaacagcgtgatggtctgctggccggcctggatagcctggcacaggatgtggccgatggtagctttggcccgctggtgaccgatgaagatgtgcatgcagcactggaacgcggcctgattgatcgcgtgggcccggatctgggcggtcgtttacgcgccggtcgcagtcgcaatgatcaggttgccgcactgtttcgcatgtggctgcgcgatgccgtgcgtcgtgttgcaaccggtgttctggatgttgtgggcgcactggcagaacaggccgcagcccatccgagtgccattatgccgggcaaaacccatctgcagagtgcacagccgattctgctggcacatcatctgctggcccatgcccatccgctgctgcgcgatctggatcgcattgttgattttgataaacgcgcagcagttagtccgtatggtagtggcgccctggccggcagtagcctgggtttagatccggatgccattgccgccgatctgggttttagcgccgccgcagataatagcgttgatgcaaccgcagcccgcgattttgccgcagaagccgcatttgtgtttgccatgattgcagttgatctgagtcgtctggccgaagatattattgtgtggagtagtaccgaatttggttatgttaccctgcatgatagctggagcaccggcagtagtattatgccgcagaaaaagaatccggatattgccgaactggcccgcggtaaaagcggtcgtctgattggcaatctggcaggcctgctggccaccctgaaagcacagccgctggcatataatcgtgatctgcaggaagataaagaaccggtttttgatagcgttgcacagctggaactgctgctgccggcaatggccggcctggtggcaagtctgacctttaatgtgcagcgcatggcagaactggccccggcaggttataccctggcaaccgatctggccgaatggctggtgcgtcagggtgttccgtttcgtagtgcacatgaagccgcaggtgcagcagtgcgtgcagccgaacagcgcggtgttggcctgcaggaactgaccgatgatgaactggccgcaattagtccggaactgaccccgcaggtgcgcgaagttctgaccattgaaggtagcgttagtgcccgcgattgccgcggcggtaccgcacctggtcgcgttgctgaacagctgaatgccattggtgaagcagcagaacgtctgcgccgtcagctggtgcgttaa

***B*spAspB**

atgaatcacaaagtgcatcatcatcatcatcatatgaacaccgatgttcgtattgaaaaagattttctgggcgaaaaagaaattccgaaagatgcatattacggtgttcagaccattcgtgcaaccgaaaattttccgattaccggctatcgtattcatccggaactgattaagagcctgggcattgtgaaaaaatctgccgcactggccaatatggaagttggtctgctggataaagaagtgggtcagtatattgtgaaagcagcagatgaagttattgaaggcaaatggaatgatcagtttattgttgatccgattcagggcggtgccggcaccagcattaatatgaatgcaaatgaagttatcgccaatcgcgcactggaactgatgggtgaagaaaaaggtaattatagcaaaatcagcccgaatagtcatgtgaatatgagtcagagtaccaatgatgcctttccgaccgcaacccatattgcagtgctgagcctgctgaatcagctgattgaaaccaccaaatatatgcagcaggaattcatgaaaaaggccgatgaatttgccggtgttattaagatgggtcgcacccatctgcaggatgcagttccgattctgctgggtcaggaatttgaagcatacgctcgtgtgattgcccgtgatattgaacgtattgccaatacccgcaataatctgtatgatattaacatgggtgccaccgccgttggtaccggcctgaatgccgatccggaatatattagcattgttaccgaacatctggccaaattttctggtcatccgctgcgtagtgcacagcatctggtggatgcaacccagaataccgattgttataccgaagtgagtagtgcactgaaagtttgcatgattaatatgagtaagatcgcaaatgacctgcgcctgatggccagtggcccgcgtgcaggtctgagcgaaattgttctgccggcccgccagccgggtagtagtattatgccgggtaaagtgaatccggttatgccggaagttatgaatcaggtggcctttcaggtgtttggtaatgatctgaccattaccagtgccagcgaagccggccagtttgaactgaatgtgatggaaccggttctgtttttcaatctgattcagagcattagcattatgaccaatgtttttaagagctttaccgaaaattgcctgaaaggcattaaggccaatgaagaacgcatgaaagaatatgtggaaaaaagtattggcatcattaccgcaattaatccgcatgttggttatgaaaccgcagccaaactggcccgcgaagcatatctgaccggtgaaagcattcgcgaactgtgtattaagtatggcgttctgaccgaagaacagctgaatgaaattctgaatccgtatgaaatgacccatccgggcattgcaggtcgcaaataa

***Ec*FumC**

atgggcagcagccatcatcatcatcatcacagcagcggcctggtgccgcgcggcagccatatgaacaccgttcgcagtgaaaaagatagcatgggcgccattgatgtgccggcagataaactgtggggcgcacagacccagcgcagtctggaacattttcgtattagcaccgaaaaaatgccgaccagcctgattcatgccctggcactgaccaaacgtgccgccgccaaagttaatgaagatctgggcctgctgagcgaagaaaaagcaagcgcaattcgtcaggccgccgatgaagttctggcaggccagcatgatgatgaatttccgctggccatttggcagaccggtagcggtacccagagcaatatgaatatgaatgaagtgctggccaatcgcgcaagcgaactgctgggtggcgtgcgtggcatggaacgtaaagttcatccgaatgatgatgttaataagagtcagagcagcaatgatgtttttccgaccgcaatgcatgttgcagcactgctggcactgcgcaaacagctgattccgcagctgaaaaccctgacccagaccctgaatgaaaaaagtcgtgcctttgcagatattgtgaaaattggccgcacccatctgcaggatgcaaccccgctgaccctgggtcaggaaattagtggttgggtggccatgctggaacataatctgaaacatattgaatacagcctgccgcatgtggccgaactggcactgggtggtaccgccgttggcaccggcctgaatacccatccggaatatgcacgccgtgttgccgatgaactggccgttattacctgtgccccgtttgttaccgcaccgaataagtttgaagcactggcaacctgtgatgcactggtgcaggcccatggtgccctgaaaggtctggccgccagcctgatgaaaattgccaatgatgttcgttggctggcaagtggtccgcgttgtggtattggtgaaattagcattccggaaaatgaaccgggcagtagtattatgccgggcaaagttaatccgacccagtgtgaagcactgaccatgctgtgctgtcaggttatgggtaatgatgttgccattaatatgggtggtgccagtggtaattttgaactgaatgtttttcgcccgatggttattcataattttctgcagagcgttcgcctgctggccgatggtatggaaagctttaataagcattgcgccgttggcattgaaccgaatcgcgaacgtattaatcagctgctgaatgaaagtctgatgctggttaccgccctgaatacccacattggctatgataaagccgccgaaattgcaaaaaaggcccataaagaaggcctgaccctgaaagccgcagcactggcactgggctatctgagtgaagccgaatttgatagctgggtgcgcccggaacagatggttggtagcatgaaagcaggtcgtcatcatcatcatcactaa

***Hsn*ADSL**

atgggcagcagccatcatcatcatcatcacagcagcggcctggtgccgcgcggcagccatatggctgctggtggtgaccatggcagcccggatagctatcgcagtccgctggcaagtcgctatgccagtccggaaatgtgttttgtgtttagtgatcgctataaattccgtacctggcgtcagctgtggctgtggctggccgaagccgaacagaccctgggcctgccgattaccgatgaacagattcaggaaatgaaaagtaatctggaaaacattgacttcaagatggcagcagaagaagaaaaacgcctgcgccatgatgttatggcacatgttcatacctttggtcattgctgcccgaaagccgcaggtattattcatctgggtgcaaccagctgctatgttggcgataataccgatctgattattctgcgtaatgcactggatctgctgctgccgaaactggcacgcgttattagccgcctggcagattttgcaaaagaacgtgcaagcctgccgaccctgggttttacccattttcagccggcccagctgaccaccgtgggtaaacgctgttgcctgtggattcaggatctgtgtatggatctgcagaatctgaaacgtgtgcgcgatgatctgcgctttcgtggcgtgaaaggcaccaccggtacccaggcaagttttctgcagctgtttgaaggcgatgatcataaagttgaacagctggataaaatggttaccgaaaaagcaggttttaaacgtgcattcattattaccggtcagacctatacccgcaaagttgatattgaagtgctgagtgtgctggccagtctgggcgccagtgttcataaaatttgcaccgatattcgcctgctggccaatctgaaagaaatggaagaaccgtttgaaaaacagcagattggtagtagcgcaatgccgtataaacgtaatccgatgcgtagcgaacgttgctgtagtctggcccgtcatctgatgaccctggttatggacccgctgcagaccgccagcgttcagtggtttgaacgcaccctggatgatagtgcaaatcgtcgtatttgcctggcagaagcatttctgaccgcagataccattctgaataccctgcagaatattagcgaaggtctggtggtttatccgaaagtgattgaacgccgtattcgccaggaactgccgtttatggccaccgaaaatattattatggccatggtgaaagcaggcggcagtcgtcaggattgtcatgaaaaaattcgcgtgctgagccagcaggcagccagcgtggttaaacaggaaggtggtgacaatgatctgattgaacgtattcaggcagatgcatattttagtccgattcatagccagctggatcatctgctggacccgagtagttttaccggtcgcgcaagccagcaggttcagcgttttctggaagaagaagtttatccgctgctgaaaccgtatgaaagtgttatgaaagttaaggcagaactgtgtctgtaa

***Pp*CMLE**

atgggcagcagccatcatcatcatcatcacagcagcggcctggtgccgcgcggcagccatatgagcaatcagctgtttgatgcatattttaccgcaccggcaatgcgcgaaatttttagtgatcgcggccgcctgcagggcatgctggattttgaagccgccctggcacgcgcagaagcaagtgcaggcctggtgccgcatagtgccgttgcagccattgaagccgcatgtcaggcagaacgctatgatgtgggtgcactggcaaatgccattgcaaccgcaggtaatagtgccattccgctggttaaagcactgggtaaagtgattgcaaccggcgtgccggaagcagaacgttatgtgcatctgggtgcaaccagccaggatgcaatggataccggtctggtgctgcagctgcgtgatgccctggatctgattgaagccgatctgggcaaactggccgataccctgagtcagcaggcactgaaacatgccgataccccgctggttggccgtacctggctgcagcatgccaccccggttaccctgggcatgaaactggccggcgttctgggtgcactgacccgtcatcgccagcgtctgcaggaactgcgtccgcgtctgctggtgctgcaatttggtggtgccagtggtagcctggcagcactgggtagtaaagccatgccggtggccgaagcactggcagaacagctgaaactgaccctgccggaacagccgtggcatacccagcgtgatcgtctggttgaatttgccagcgtgctgggtctggttgcaggtagcctgggtaaatttggtcgcgatattagtctgctgatgcagaccgaagccggcgaagtgtttgaaccgagtgcaccgggcaaaggcggtagcagtaccatgccgcataaacgcaatccggtgggcgcagcagttctgattggtgccgccacccgcgtgccgggtctgttaagcaccctgtttgccgcaatgccgcaggaacatgaacgtagcctgggtctgtggcatgccgaatgggaaaccctgccggatatttgttgcctggtgagtggtgcactgcgccaggcccaggttattgcagaaggtatggaagttgatgcagcccgtatgcgtcgtaatctggatctgacccagggtctggttctggcagaagcagtgagcattgttctggcacagcgcctgggtcgcgatcgcgctcatcatctgctggaacagtgctgccagcgtgcagtggccgaacagcgccatctgcgtgcagttctgggtgacgaaccgcaggtgagtgccgaactgagcggtgaagaactggatcgcctgctggacccggcacattatctgggccaggcacgtgtgtgggtggcacgtgccgtgagtgaacatcagcgctttaccgcataa

***Ka*AzpD**

atgaatcacaaagtgcatcatcatcatcatcatatgagtggccgtggcgataccacccaggatgccggcctgctgagcccggttcgtgcaggtaccccggtggaagaagcagtttgcgatcaggcctggctgcaggcaatgctggatgcagaagccgccctggcacgtgcacaggcacgtctgggcaccgtgccggctgctgctgcacgtaccattagcgaagcagcacgtgccgaaaattttgaactgcgtgaactggcactggccgcccgcgaaaccgccaatccggtggtgggcctggtgcaggcactgacccgcgttgtgagtgcaaccgatccggcagcagccgaatatgtgcatcgcggtagtaccagtcaggatgtttttgataccggcgccatgctggtggcacgccgcgcattactgattattcgcgcagatctggcacgcaccaccaccgccctgggtgcactggctaaacgccatcgtgataccgtgatggcaggtcgtaccctggcactgcaggcagttccgaccacctttggcctgaaagcagcaggttggcgtcagctgctgctggatgcagatcgccgcctgggtcaggtttatgatcatggcctgccggttgccctgggcggtgcagcaggtaccctggctggctatctggaatatgcccgcctggatggtggtgaacgtggcgaaccggatctggatcgctatctggatctggatcgttatctggatgcactggttgatgcatttgccgatgaaaccggtctggcccgtccggtgctgccgtggcatgttctgcgtaccccgattgccgatctggcagccgccctggcctttaccgcaggtgcactgggcaaaattgcaattgatgttcagagtctgacccgcaccgaagttggtgaagttaccgaaccggcagtggcaggccgcggctcaagtagcgcaatgccgcataaacgtaatccggttctggcaaccctgattcgtagtgccgcactgcaggttccggcaattgcaggtgccctgagtcagtgcctggttagcgaagatgaacgtagtgcaggcgtttggcatgcagaatggcagctgctgcgcgaatgcctgcgcctgaccggcggtgcagcccataccgctgtggaactggcagaaggtctggatgttcgcaccgatcgtatgtttgccaatctgcagctgaccggcagtcagattgttagcgaacgcattgccgccgtgctggcaccgcatctgggcaaagttgccgcaaaacagctgctgaccgatgcagcaacccgcagccaggaaaccggcgatccgctgccgaccgtgctggcagcaacccctgccctgcagggtattctggacccggccgcactggccaccctgtgtgatccgagcgcctataccggtgcagccagcagtctggtggatcgcgccctgcgctaa

***Pa*FlcC**

atgaatcacaaagtgcatcatcatcatcatcatatgcctagtcatattaccgaaagccgtattcatggcggtgcctatagtagtccggcatttgcagccatttttagtgataccaatcaggtgcgccgttggctggatgtggaacgcgcactggccgcaacccaggcagaaatgggtattattccgcatgaagcagcacgcgaaattgatcgcgcagcacaggttgaacgttttgatctgacccagctgggccgcgaaagcctggaaaccggtcatctgctggttccgaccattcgtgcactggcccgcagttgcgaaggtagctggggcgaatatgtgcattatggtgttaccacccaggatattctggataccggtctgatgctgcaggtgaaagaagcatggggtcatgccctgggcctgctgcatagtattcgtggtcatctgttagccctggcactgcgtcatcagcataccccgatggtggcacgtacccatggtcagcaggccctgccgaccacctttggctataaagttgcagtgtgggttgatgaaattgatcgtcatctggcacgttttgatgaagcacgcgaacgcgtgctggtgggtaatctgaccggtgcagttggcaccctggccagctttggcgcacagggttttgaactgcagcgtcgcaccctggcacgcctgggtctgggtgctccgagcaccagttggcatagcgcccgcgatcgcgttctggaagttgcaggcctgctggttcaggtgagcgttaccctgggtcgtgtggccaatgaaatctatcatctgcagcgcagcgaaattgatgaagtgcgtgaaggcagccgtccgggtcaggttggcagcagtaccatgccgcataaacgtaatccgagcagtgtggatctggttagtgcactgagtcgcctggtgcgcgcacagatggtggcactgaccgatgccgcctttcagctgcatgaacgcgatggcaccgcatggcgcattgaatgggccgccctgccggaactgtttgtgtatgcaggtgcactgctgacccgcatggaagccgttctggcagaaggcctggaagttcgcgaagaacgcatgcgcgcaaatctggatctgctgggtggcctgattctgagcgaacgtgtgatgctggcactggccggccagtttggcaaacagaccgcccatgaactgctgcatgaaattacccaggccagccagcgtcagggtgttccgtttcgcgaagccctgctgggtcagccgcgcttacgtggttgttttagtgcagaagcactggaagaactgctggacccggtgggttatgtgggtctggcagcagaaatggtggatctggtggccgataaaccgcgtaccggtgccagcggcgatcgccctgaaggcgctgcaagcagtcagggtaatccgggccagagccagtatgtgatggaagatcgctaa

***Sd*CreD**

atgaatcacaaagtgcatcatcatcatcatcatatgacctttcagctgagcccggaactggccgccgttgtggatagcggcctgctgagcccggtgcgcgcaggtacaccggttgaagccgcagttagtgatgcagcctggctggcagcaatggttgaagccgaaaccgcactggttcgcgcacaggcccgtctgggcaccgtgcctgaaagcgccgccgctgcaattgttgaagcagcacgcccggaacgcctggatctggtggcactggcacgcgcaagtcgtgaaaccgccaatccggttgtgggctttgttaaagcactgaccgccgtggttgccgccgaagatccggccgcagcagaatatgttcatcgcggtagtaccagccaggatattctggataccgccaccatgctggttgttcgccgtgcaggcgttctgattcgtgcagatctggatcgctgcgccgcagccctggaacgtctggcacgtacccatcgcgccaccccgatggcaggtcgcaccttaaccctgcatgcagtgccgaccacctttggcctgaaagccgccggctggctgcatctggttaccgaagcacgccgccgcaccgcagccttagcagctgcactgccggttgaactgggcggtgcagccggtaccctggcaggttatctggaacatgcaaataatccgggtgacgattatgcagatcgcctggtggaagcatacgcacatgaaaccggtctggccccggccaccctgccttggcatgttctgcgtaccccgattgcagataccggcgccgtgtgtgcatttctggccgcagcactgggcaaaattgccgttgatgtgcagagcctggcccgtaccgaagtgggtgaagttaccgaaccggccgttgccggtcgtggtgccagtagcgcaatgccgcataaacgcaatccggttctggccaccctgattcgtagtgccgccctgcaggtgccgcagcatgcagctgtgctgtatggtgccatgctggcagaagatgaacgtagtggtggcgcatggcatgccgaatggcagccgctgcgcgaatgtctgcgtctggccggtggcgccgcacataccgcagttgaactgctgaccggcctgaccgttgatgcagatcgtatgcgcgcaaatctggatctgaccggtggccagattgtgagcgaacgtgtggcagcagtgctgaccccgctgctgggtaaagcccaggcacgtgcactgctgacccgcgctagtcatgaagcagcagatcgcggtaccagcctggcagaagtgctgagcgccgcaccggaagtgacccgtcatctgaccgcagccgaactggaagaactgctggacccgacccgctatctgggtgccgcccctggtctggtggatcgtgcagtgggtggcccggatcgcgccgttcctcatcgtagcgcagcc

## List of mutations

Table S2.2: List of mutations investigated in this study

| Parental enzyme | Mutation(s) |
| --- | --- |
| ScCreD | R341A |
|  | R341K |
|  | S302A |
|  | S303A |
|  | P306G |
|  | K308A |
|  | N310A |
|  | T122A |
|  | S123C |
|  | S123T |
|  | S123N |
|  | T122A, S123A |
|  | L170A |
|  | W346A |
|  | H347A |
|  | V96A |
|  | H118N |
|  | E49Q |
|  | H253N |
| BspAspB | L358R |
|  | L358R, N142Q |
| MtASL | R324A |
|  | Y322R, R324A |

## Enzyme expression of wild-type proteins and variants

Each plasmid was transformed into *E. coli* BL21(DE3) via heat shock (42 °C, 10 s). Each transformant was cultured at 37 °C in 0.5 L Terrific Broth (TB) media with 50 μg/mL kanamycin (in case of pET-28a(+) and pET-26b(+) plasmids) or with 100 μg/mL ampicillin (in case of pColdII plasmids) until OD_600_ reached 0.6-0.8. Isopropyl β-d-thiogalactopyranoside (IPTG) was added to the culture to reach the concentration of 0.1 mM (in case of pET-28a(+) plasmids) or 0.02 mM (in case of pColdII plasmids) to induce gene expression, and the culture was incubated at 15 °C (in case of pColdII plasmids) or 20 °C (pET-28a(+) enzymes) for 24 h. The cells were harvested by centrifugation (20 min; 4 °C; 3,600 g and resuspended in lysis buffer (see Section 2.6). After lysis by sonication on ice (t_on_ 1 sec, t_off_ 5 sec, total time 5 min, 30% amplitude), the cell debris were removed by centrifugation (4 °C, 38,900 g, 20 min) and the supernatant used for protein purification (see 2.6).

## Protein purification by Immobilized Metal Affinity Chromatography

**Purification of *Sc*CreE**

**Column:** 5 mL HisTrap FF column (GE)

| **Lysis buffer:** 50 mM KPi, 300 mM NaCl, 20 mM imidazole, pH 8.0  **Elution buffer:** 50 Mm KPi, 300 mM NaCl, 250 mM imidazole, pH 8.0  **Storage buffer:** 50 mM KPi, 300 mM NaCl, 20% glycerol, pH 8.0 |
| --- |

The purification was performed at 4 °C. After loading of the protein sample onto the column, the protein was eluted with elution buffer. The protein was concentrated with Vivaspin 20 (50 kDA) and desalted via dialysis (dilution 1:100 for 3 times) against the storage buffer. The protein showed instability overnight at 4 °C in the storage buffer, thus the precipitated protein was centrifuged and the supernatant used for the activity tests. The protein was analyzed by SDS-PAGE (Figure S2.3).

**Purification of all other proteins**

**Column:** 5 mL HisTrap FF column (GE)

| **Lysis buffer:** 20 mM HEPES, 450 mM NaCl, 50 mM imidazole, 20 vol% glycerol pH 8.0  **Elution buffer:** 20 mM HEPES, 500 mM imidazole, 20 vol% glycerol pH 8.0  **Storage buffer:** 20 mM HEPES, 20 vol% glycerol pH 8.0 |
| --- |

The purification was performed at room temperature. After loading of the supernatant onto the column, the protein was eluted with elution buffer. The protein was concentrated with Vivaspin 20 (100 kDA) and desalted with PD-10 desalting column (GE healthcare) against the storage buffer. The protein concentration was determined by NanoDrop spectrophotometer (see 2.7). The samples were stored at -20 °C until further use. The protein was analyzed by SDS-PAGE (Figure S2.1 -Figure S2.2).

## Determination of the concentration of the proteins.

The concentration of the proteins was determined using a NanoDrop spectrophotometer and using the computed extinction coefficient based on each amino acid sequence.

Table S2.3: Extinction coefficients and determination of the protein concentrations

| Enzyme | ε^1,2^ (1/(M۰cm)) | Concentration^3^ (mg/mL) | Yield  (mg/L) |
| --- | --- | --- | --- |
| ScCreD | 37470 | 12.1 | 84 |
| BspAspB | 27850 | 10.2 | 70 |
| SdCreD | 36440 | 1.0 | 8 |
| KaAzpD | 37930 | 37.0 | 260 |
| PaFlcC | 50420 | 33.7 | 236 |
| SspFzmL | 36440 | 42.8 | 300 |
| MtASL | 40450 | 103.1 | 720 |
| EcFumC | 33460 | 62.6 | 438 |
| HsnADSL | 42400 | 14.5 | 102 |
| PpCMLE | 33460 | 42.7 | 298 |
| ScCreE | 64860 | 4.0 | 12 |

^1^Extinction coefficients at 280 nm computed in water

^2^Calculated with the online tool ProtParam (link: <https://web.expasy.org/protparam/>)

^3^Determined with NanoDrop spectrophotometer

Of note: *Sd*CreD could not be obtained with purity >50% (see Fig. S2.1).

With *Sc*CreD, we have observed that conversion drops between using 10 μM and 1 μM enzyme (66% relative activity at 1 µM, Figure 4). It seems that *Sd*CreD behaves similarly and stays within this range of activity drop, in line with the lower purity (~50% purity corresponds to 5 µM, and a measured relative activity of 84%).

## SDS-PAGES of the protein samples


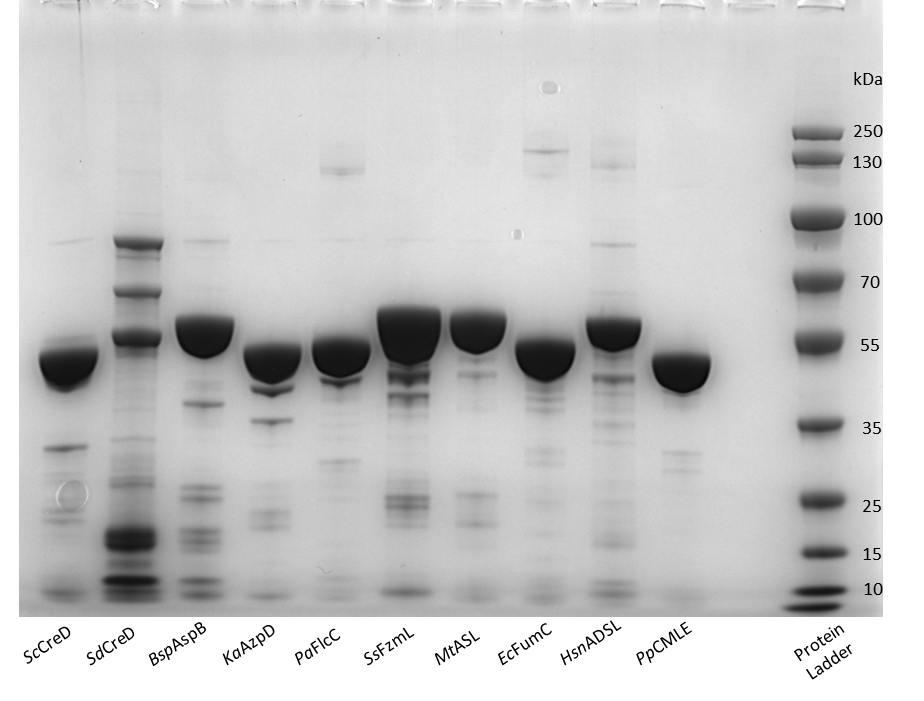


Figure S2.1: SDS-PAGE with all the over-expressed homologue proteins (Tris-MOPS-SDS running buffer; protein ladder PageRuler^TM^ Prestained protein ladder).


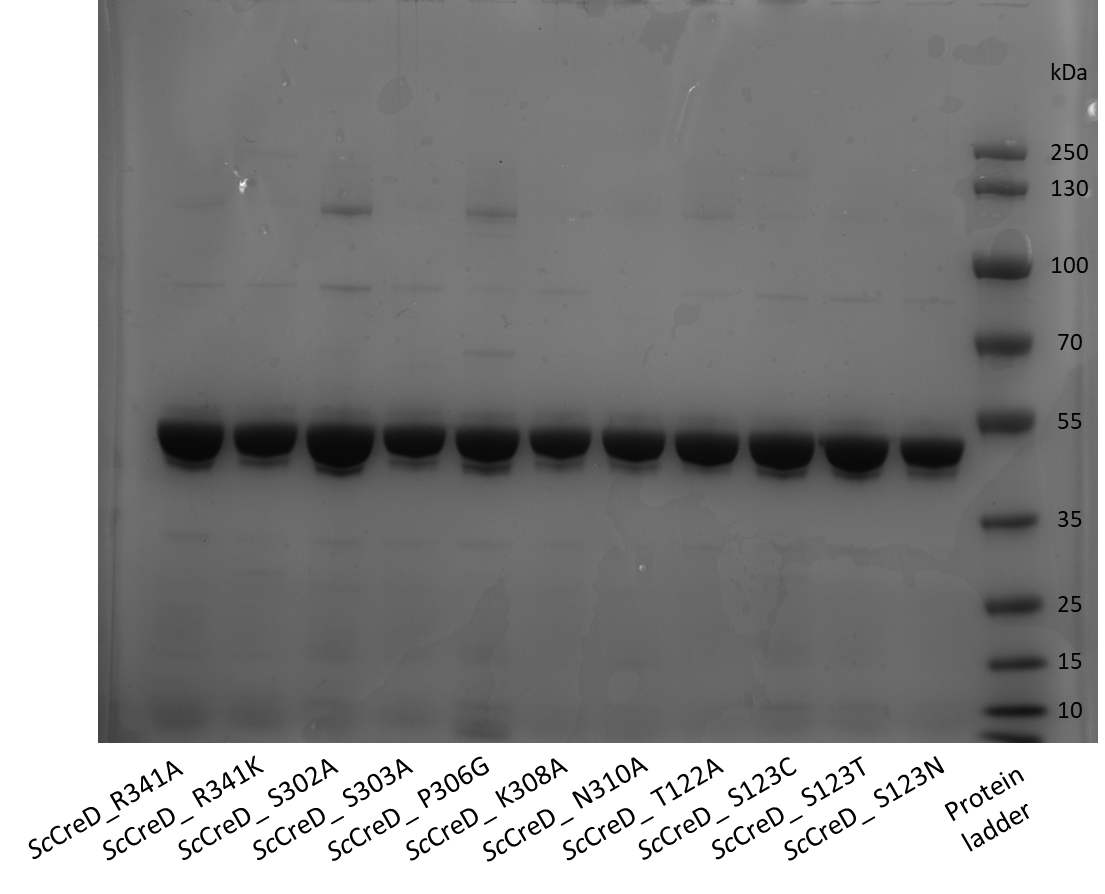


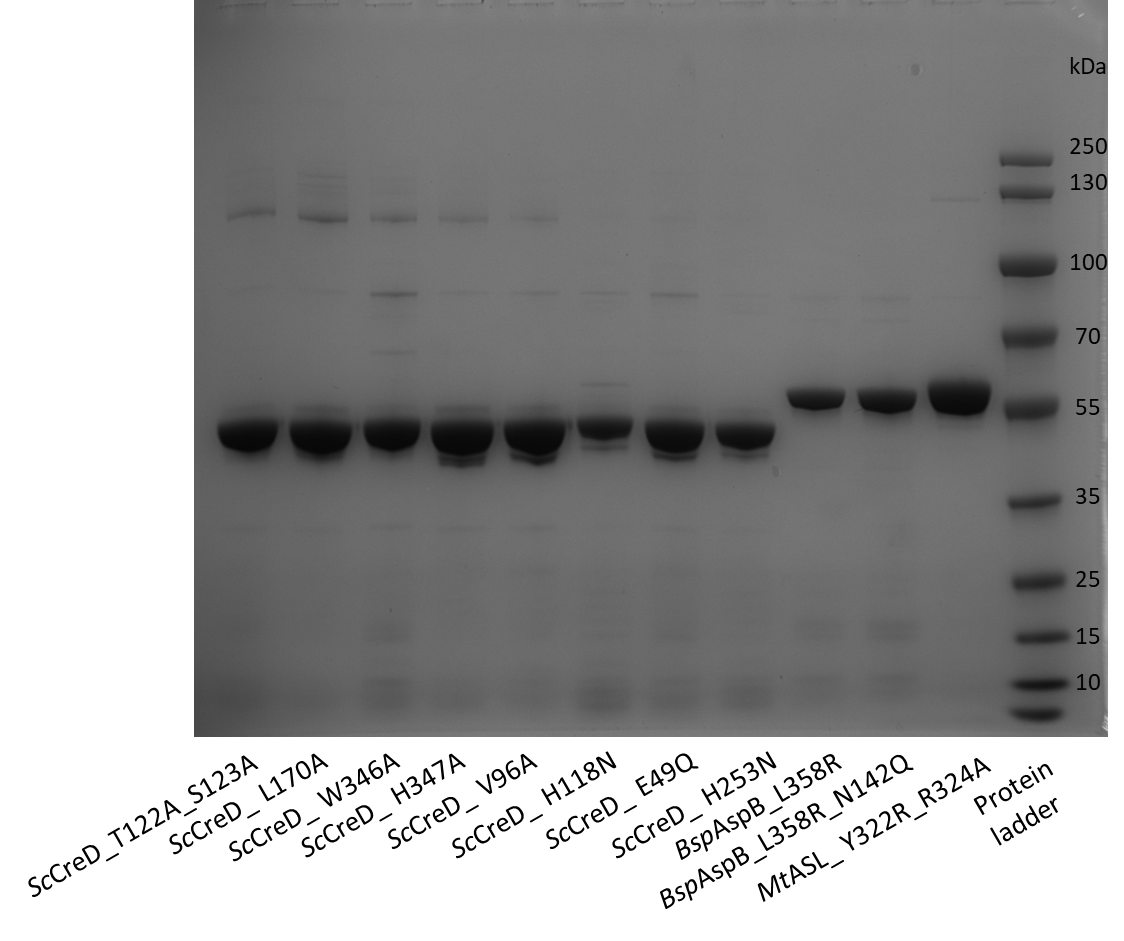


Figure S2.2: SDS-PAGES with all the over-expressed variant proteins (Tris-MOPS-SDS running buffer; protein ladder PageRulerTM Prestained protein ladder).


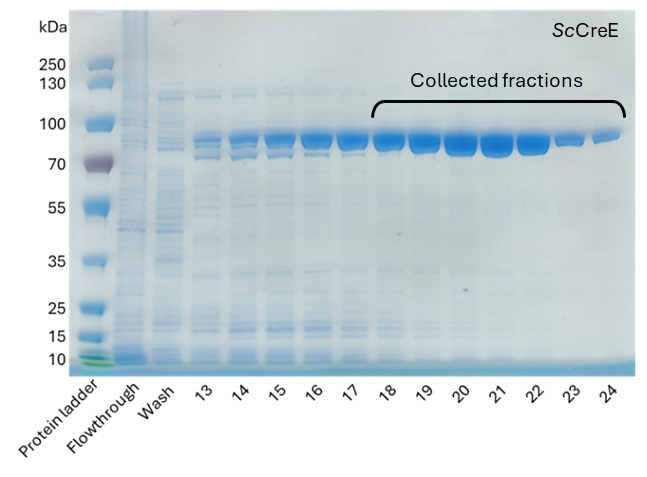


Figure S2.3: SDS-PAGE from the purification of ScCreE. The fractions from 18 to 24 were collected and used for the biotransformations (Tris-MOPS-SDS running buffer; protein ladder PageRulerTM Prestained protein ladder).

# *Sc*CreE/*Sc*CreD cascade

## Conversion of l-aspartic acid to fumaric acid

The following stock solutions were combined in a 1.5 mL microcentrifuge tube (V_fin_ = 500 μL) in the following order: Tris-HCl_(aq)_ buffer (10 mM, pH 7.5, buffer 1), L-aspartic acid (25 mM in buffer 1 to reach final concentration of 5 mM), NADPH (100 mM in buffer 1 to reach final concentration of 20 mM), purified *Sc*CreE (to reach final concentration of 10 μM) and purified *Sc*CreD (to reach final concentration of 10 μM). The reactions were incubated in a shaker incubator for 16 h at 30 °C (120 rpm). After 16 h, the samples were dried under vacuum with a Speedvac (EZ-2 Plus HCl system) for 3 h. The products fumarate and nitropropanoate were derivatized to their methyl esters by adding 200 μL of BF_3_۰MeOH (14 w%) solution to the residues and the resulting mixtures were incubated in a thermoshaker for 1 h at 65 °C (550 rpm). 300 μL of brine were then added and the mixtures were extracted two times with 250 μL of EtOAc (spiked with 10 mM of (*R*)-limonene as internal standard). The combined organic fractions were dried over anhydrous Na_2_SO_4_ and analyzed with GC-FID (Method GC-01). All biotransformations were carried out in triplicates.

## Cascade setup for the analysis of HNO_2_

The set-up of the cascade (Section 3.1) was modified to avoid the use of excess of NADPH. The coenzyme was supplied by a recycling system consisting of catalytic amounts of NADPH, an alcohol dehydrogenase (*Lk*ADH_mut_) and isopropanol as reductant. The various stock solutions were combined in a 1.5 mL microcentrifuge tube (V_fin_ = 500 μL) in the following order: Tris-HCl_(aq)_:isopropanol (95:5; 60.3 mM of Tris to reach final concentration of 50 mM, pH 7.5), L-aspartic acid (6.0 mM to reach final concentration of 5.0 mM), NADPH disodium salt (0.6 mM to reach final concentration of 0.5 mM) and MgCl_2_ (1.2 mM to reach final concentration of 1.0 mM). This solution was supplemented with *Lk*ADH_mut._ (to reach final concentration of 10 μM), purified *Sc*CreE (to reach final concentration of 10 μM) and purified *Sc*CreD (to reach final concentration of 10 μM). Reaction controls were performed with deionized H_2_O instead of the stock solution of purified enzyme (controls 1 and 2 contained: 1. *Sc*CreE and *Lk*ADH_mut_; 2. *Sc*CreD and *Lk*ADH_mut_; 3. *Sc*CreE, *Sc*CreD and *Lk*ADH_mut_)^[11]^ The reactions were incubated in an orbital thermoshaker for 18 h at 30 °C (120 rpm). Then, 150 μL were analyzed with the Griess test (see section 3.3.1). The reactions were performed in triplicates.

## Griess test for analysis of HNO_2_^[12]^

### Generation of calibration curve

Reagent A (**13**) *N*-(1-naphthyl)ethylenediamine dihydrochloride (25 mg; [**13**]_stock_ = 1 mg/mL) was solubilized in 25 mL of deionized water. Reagent B (**12**) was obtained by mixing sulfanilic acid (250 mg; [**12**]_stock_ = 10 mg/mL) with H_3_PO_4_ (1.5 equivalents in mass) in 25 mL of deionized water. A stock solution of NaNO_2_ (10 mM, solution C) was prepared in deionized water. Various concentrations between 25 µM and 300 µM were obtained by diluting solution C with deionized water. These samples (150 µL) were then mixed with solution A (25 µL) and solution B (25 µL). Additionally, a control sample was prepared with deionized water instead of NaNO_2_. Finally, deionized water (1.3 mL) was added to all samples to a total of 1.5 mL per sample. The mixtures were left to react for 30 min (room temperature), diluted by the addition of 1.3 mL of deionized H_2_O and analyzed spectrophotometrically at 548 nm.

### Quantification of HNO_2_

The reaction mixtures from 3.2 (150 µL) were mixed with solution A (25 µL) and solution B (25 µL) followed by the addition of deionized water (1.3 mL). The samples were left to react for 30 min (room temperature), diluted by the addition of 1.3 mL of deionized H_2_O and analyzed spectrophotometrically at 548 nm. The concentration was obtained by using the calibration curve (Fig. S3.1).

| **A** | **B** |
| --- | --- |
|  | 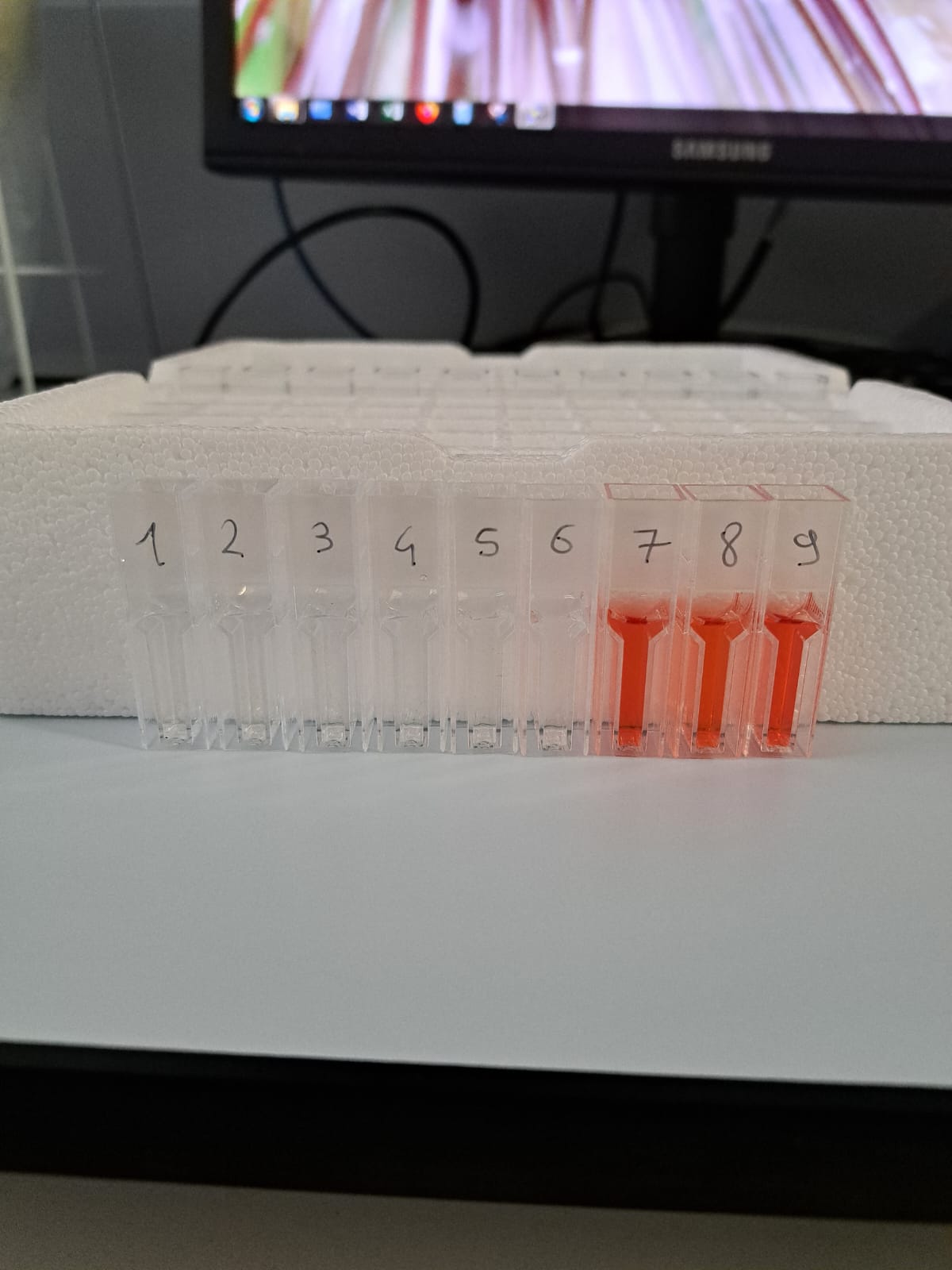 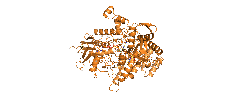 *Sc*CreE 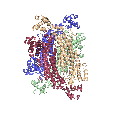 *Sc*CreD 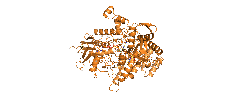 *Sc*CreE 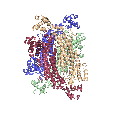 *Sc*CreD |

Figure S3.1: A: Quantification of HNO_2_ by use of a calibration curve for the biotransformations involving: ScCreE and ScCreD (yellow, 255 μM of HNO_2_ formed), ScCreE (orange), ScCreD (grey); B: color change obtained from the Griess test. In order: 1-3 ScCreE reaction, 4-6 ScCreD reaction, 7-9 ScCreE + ScCreD.

# Hydronitration catalyzed by *Sc*CreD

## Protocol for the screening of the reaction conditions (pH value, buffer, and concentration of NaNO_2_)

Preliminary screenings on 5 mM fumarate focused on the reaction conditions with variations of NaNO_2_ concentration, pH value and buffer species. The following stock solutions were combined in a 1.5 mL microcentrifuge tube (V_fin_ = 500 μL) in the following order: Tris-HCl (10 mM, pH 7.0 or 7.5) or NaPi (10 mM, pH 7.0 or 7.5), NaNO_2_ (1 or 1.25 M stock solution in corresponding buffer, to reach final concentration 50, 200, 250, 500, 750 mM), sodium fumarate (25 mM stock solution in corresponding buffer, to reach final concentration 5 mM), and *Sc*CreD (to reach final concentration 10 μM). Reactions were incubated in orbital shaker for 16 h at 30 °C (120 rpm). Then, MeOH was added to denature the protein (500 μL), the samples centrifuged for 3 min (14,000 rpm) and filtered with cotton on a microscale. The solutions were analyzed with HPLC-MS (Method MS-01) for the qualitative detection of product formation. The biotransformations were performed in duplicates.

## Protocol for the determination of enzyme turnover number (TON)

Sodium fumarate (to reach a final concentration of 50 mM), NaNO_2_ (to reach a final concentration of 500 mM), and HEPES (to reach a final concentration of 400 mM) were solubilized in deionized water and the pH adjusted to 7.0 with NaOH (10 M). This solution was supplemented with varying amounts of *Sc*CreD (to reach a final concentration of 10, 1, 0.1, 0.01 μM). The volume was adjusted to 500 μL using *Sc*CreD storage buffer (see 2.6). Reactions were incubated in an orbital shaker for 18 h at 30 °C (120 rpm). The samples were then worked up to remove excess of nitrite: 25 µL of the reaction were mixed with urea_(aq)_ (125 µL, 0.5 M; spiked with *cis,cis*-muconic acid (IS), 5 mM), HCl_(aq)_ (100 µL, 1 M) and incubated in a thermoshaker (45 °C, 45 min, 300 rpm). The reactions were centrifuged (18,000 rpm, 2 min) and filtered with cotton. **11** was quantified with HPLC-UV (Method UV-01) using a calibration curve prepared with authentic reference material. The observed difference between yield and conversion implies a non-quantitative recovery and possible formation of by-products not detected with the used method.

## Inhibition studies – Impact of nitropropanoate on reaction outcome

A stock solution containing sodium fumarate_(s)_ (to reach a final concentration of 50 mM), NaNO_2(s)_ (to reach a final concentration of 500 mM), and HEPES_(s)_ (to reach a final concentration of 100 mM) solubilized in deionized water, was mixed with a solution of **11**_(aq)_ (to reach a final concentration of 5, 15, 20, 50 mM) in HEPES_(aq)_ (to reach a final concentration of 100 mM) in a 1.5 mL microcentrifuge tube. The pH was adjusted to 7.0 with NaOH (10 M) and *Sc*CreD_(aq)_ was added (in storage buffer see 2.6, to reach a final concentration of 10 μM). The reactions (V_fin_ = 500 μL) were incubated in an orbital shaker for 22 h at 30 °C (120 rpm). The samples were then worked up to remove excess of nitrite: 25 µL of the reaction were mixed with urea_(aq)_ (125 µL, 0.5 M; spiked with *cis,cis*-muconic acid (IS), 5 mM), HCl_(aq)_ (100 µL, 1 M) and incubated in a thermoshaker (45 °C, 45 min, 300 rpm). The reactions were centrifuged (18,000 rpm, 2 min) and filtered with cotton. The formation of **11** (Figure S4.1) was detected with HPLC-UV (Method UV-01).

Figure S4.1: Impact of initial concentration of **11** (5-50 mM) on product formation in the hydronitration of fumarate. The grey bars represent **11** formed during the biotransformations (difference between the concentrations from the biotransformations and the reaction controls in absence of the enzyme). The results were performed in triplicates and displayed as the average value (± standard deviation).

## Chromatograms

### Detection of the formation of nitropropanoic acid by HPLC-MS (qualitative analyses performed with **Method MS-01**)

The following chromatograms show the results from the preliminary activity screening obtained varying [NaNO_2_], pH and buffer type. The data were analyzed qualitatively.


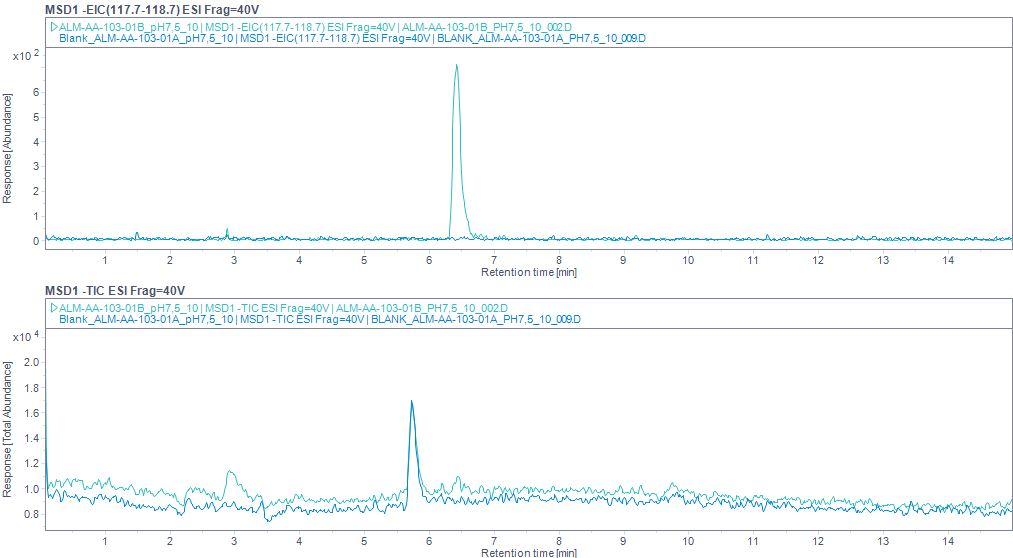


Figure S4.2: Conversion of fumarate by ScCreD according to 4.1. Retention times: 5.7 min fumaric acid, 6.5 min 3-nitropropionic acid. Overlay of traces from analysis of extracted ion (above; M-1 negative mode; m/z 118 for the detection of **11**; blue: reaction control without enzyme, green: biotransformation), and traces from analysis of TIC (below; blue: reaction control without enzyme, green: biotransformation). **Reaction conditions**: [sodium fumarate] = 5 mM, [NaNO_2_] = 50 mM, [Tris] = 10 mM, [ScCreD] = 10 µM, pH 7.5, 30 °C, 120 rpm, 16 h


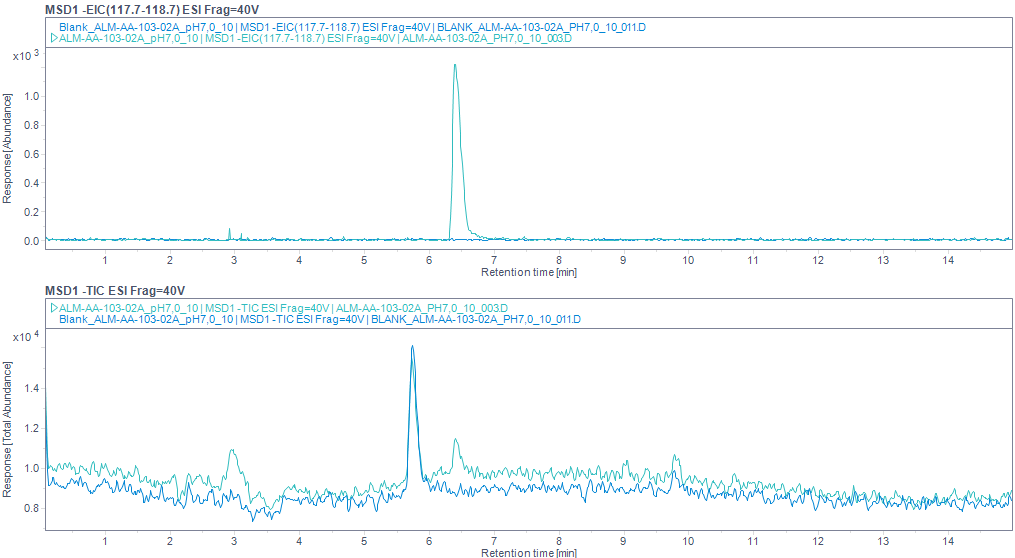


Figure S4.3: Conversion of fumarate by ScCreD according to 4.1. Retention times: 5.7 min fumaric acid, 6.5 min 3-nitropropionic acid. Overlay of traces from analysis of extracted ion (above; M-1 negative mode; m/z 118 for the detection of **11**; blue: reaction control without enzyme, green: biotransformation), and traces from analysis of TIC (below; blue: reaction control without enzyme, green: biotransformation). **Reaction conditions**: [sodium fumarate] = 5 mM, [NaNO_2_] = 50 mM, [Tris] = 10 mM, [ScCreD] = 10 µM, pH 7.0, 30 °C, 120 rpm, 16 h


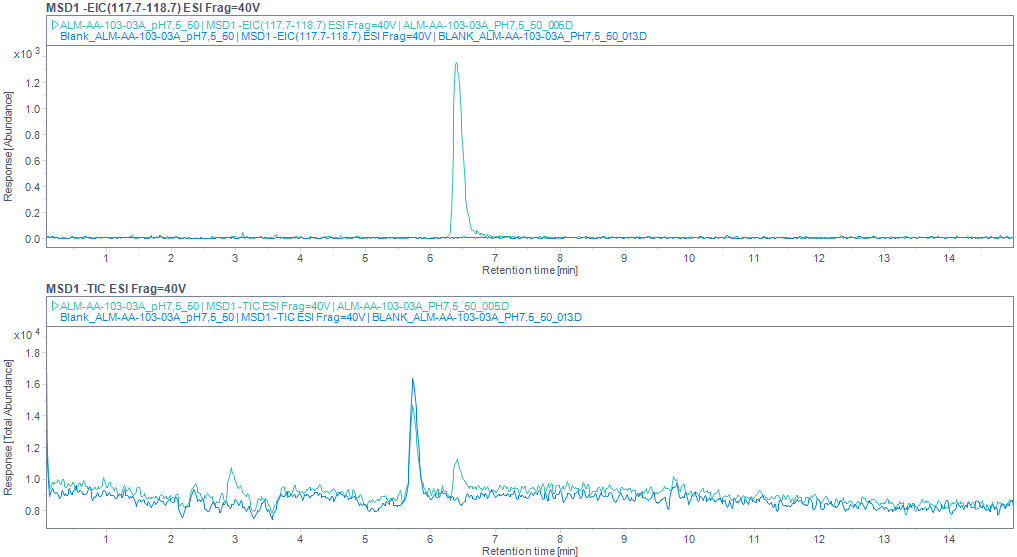


Figure S4.4: Conversion of fumarate by ScCreD according to 4.1. Retention times: 5.7 min fumaric acid, 6.5 min 3-nitropropionic acid. Overlay of traces from analysis of extracted ion (above; M-1 negative mode; m/z 118 for the detection of **11**; blue: reaction control without enzyme, green: biotransformation), and traces from analysis of TIC (below; blue: reaction control without enzyme, green: biotransformation). **Reaction conditions**: [sodium fumarate] = 5 mM, [NaNO_2_] = 250 mM, [Tris] = 10 mM, [ScCreD] = 10 µM, pH 7.5, 30 °C, 120 rpm, 16 h


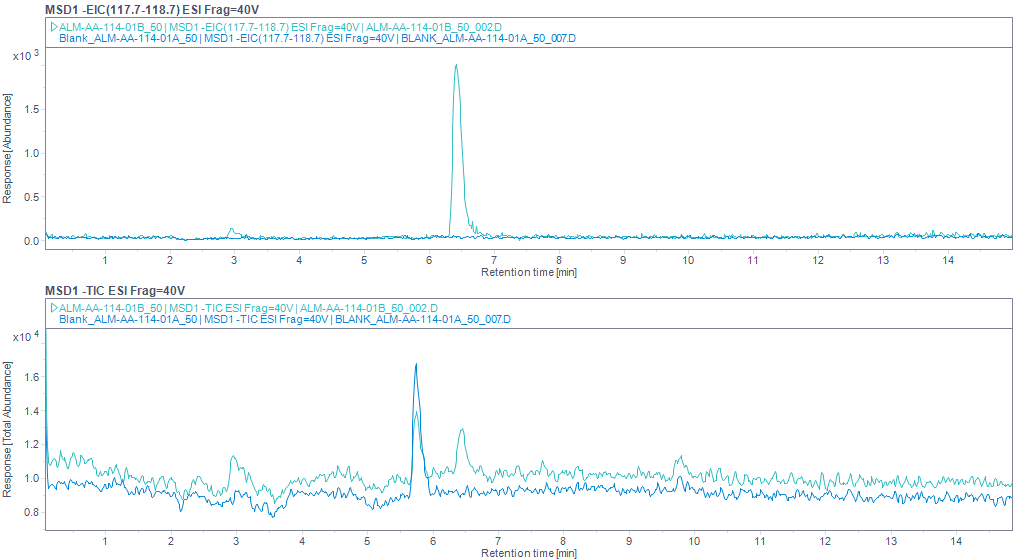


Figure S4.5: Conversion of fumarate by ScCreD according to 4.1. Retention times: 5.7 min fumaric acid, 6.5 min 3-nitropropionic acid. Overlay of traces from analysis of extracted ion (above; M-1 negative mode; m/z 118 for the detection of **11**; blue: reaction control without enzyme, green: biotransformation), and traces from analysis of TIC (below; blue: reaction control without enzyme, green: biotransformation). **Reaction conditions**: [sodium fumarate] = 5 mM, [NaNO_2_] = 250 mM, [Tris] = 10 mM, [ScCreD] = 10 µM, pH 7.0, 30 °C, 120 rpm, 16 h


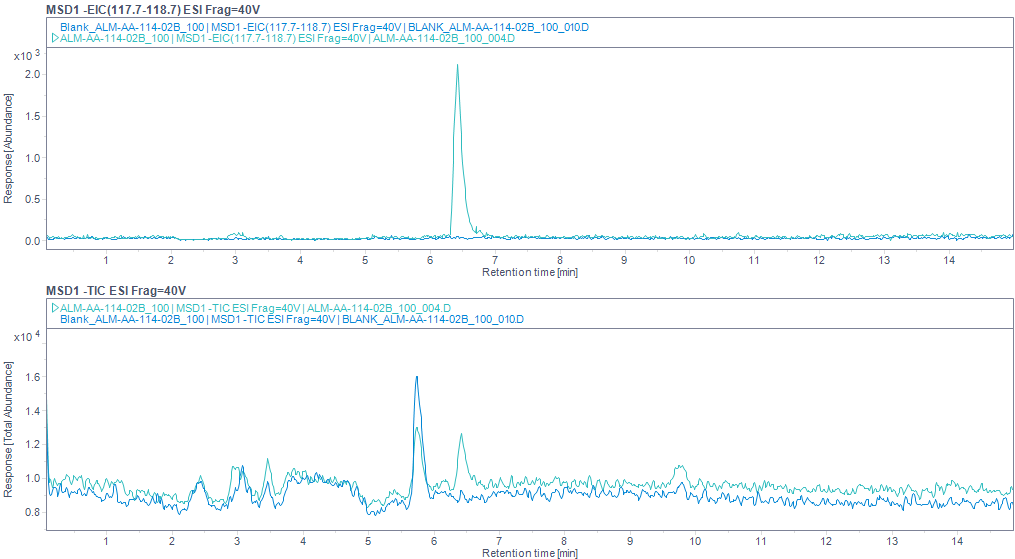


Figure S4.6: Conversion of fumarate by ScCreD according to 4.1. Retention times: 5.7 min fumaric acid, 6.5 min 3-nitropropionic acid. Overlay of traces from analysis of extracted ion (above; M-1 negative mode; m/z 118 for the detection of **11**; blue: reaction control without enzyme, green: biotransformation), and traces from analysis of TIC (below; blue: reaction control without enzyme, green: biotransformation). **Reaction conditions**: [sodium fumarate] = 5 mM, [NaNO_2_] = 500 mM, [Tris] = 10 mM, [ScCreD] = 10 µM, pH 7.0, 30 °C, 120 rpm, 16 h


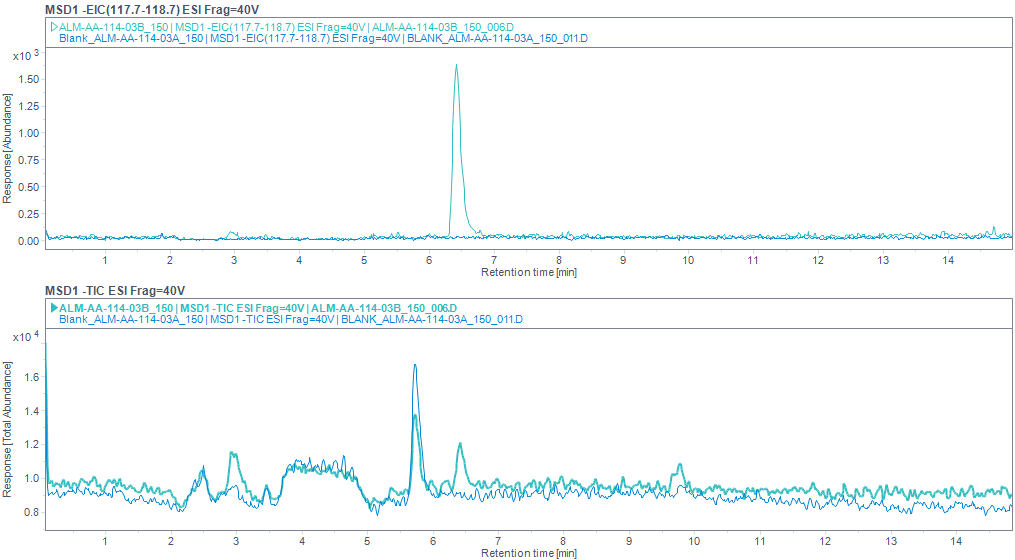


Figure S4.7: Conversion of fumarate by ScCreD according to 4.1. Retention times: 5.7 min fumaric acid, 6.5 min 3-nitropropionic acid. Overlay of traces from analysis of extracted ion (above; M-1 negative mode; m/z 118 for the detection of **11**; blue: reaction control without enzyme, green: biotransformation), and traces from analysis of TIC (below; blue: reaction control without enzyme, green: biotransformation). **Reaction conditions**: [sodium fumarate] = 5 mM, [NaNO_2_] = 750 mM, [Tris] = 10 mM, [ScCreD] = 10 µM, pH 7.0, 30 °C, 120 rpm, 16 h


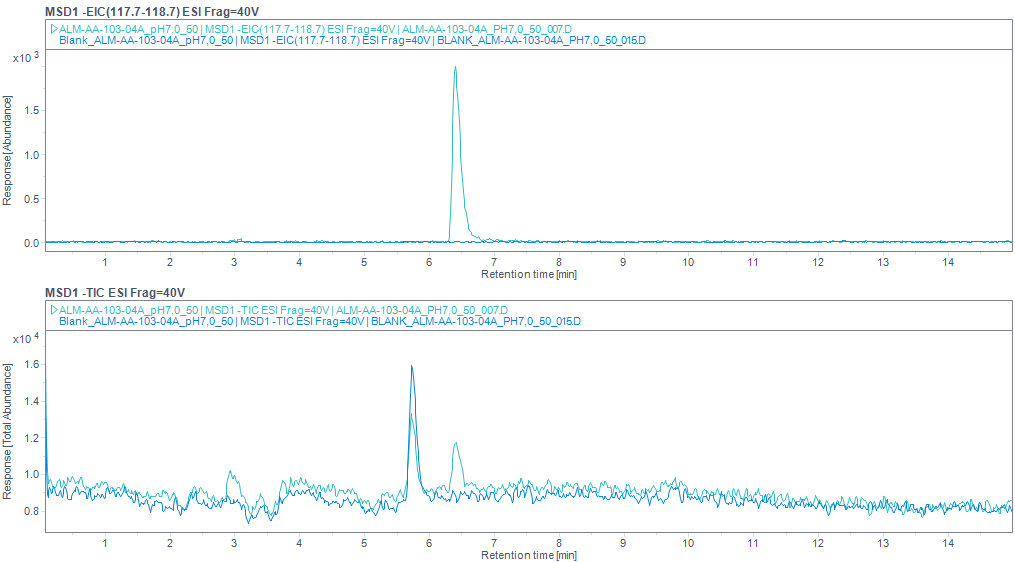


Figure S4.8: Conversion of fumarate by ScCreD according to 4.1. Retention times: 5.7 min fumaric acid, 6.5 min 3-nitropropionic acid. Overlay of traces from analysis of extracted ion (above; M-1 negative mode; m/z 118 for the detection of **11**; blue: reaction control without enzyme, green: biotransformation), and traces from analysis of TIC (below; blue: reaction control without enzyme, green: biotransformation). **Reaction conditions**: [sodium fumarate] = 5 mM, [NaNO_2_] = 250 mM, [Tris] = 10 mM, [ScCreD] = 10 µM, pH 7.0, 30 °C, 120 rpm, 16 h


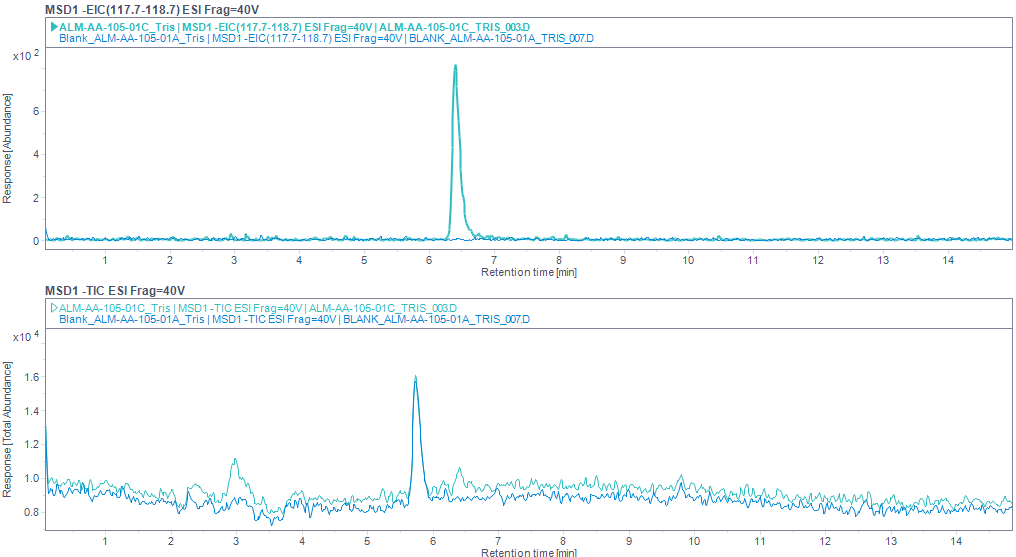


Figure S4.9: Conversion of fumarate by ScCreD according to 4.1. Retention times: 5.7 min fumaric acid, 6.5 min 3-nitropropionic acid. Overlay of traces from analysis of extracted ion (above; M-1 negative mode; m/z 118 for the detection of **11**; blue: reaction control without enzyme, green: biotransformation), and traces from analysis of TIC (below; blue: reaction control without enzyme, green: biotransformation). **Reaction conditions**: [sodium fumarate] = 5 mM, [NaNO_2_] = 25 mM, [Tris] = 10 mM, [ScCreD] = 10 µM, pH 7.0, 30 °C, 120 rpm, 16 h


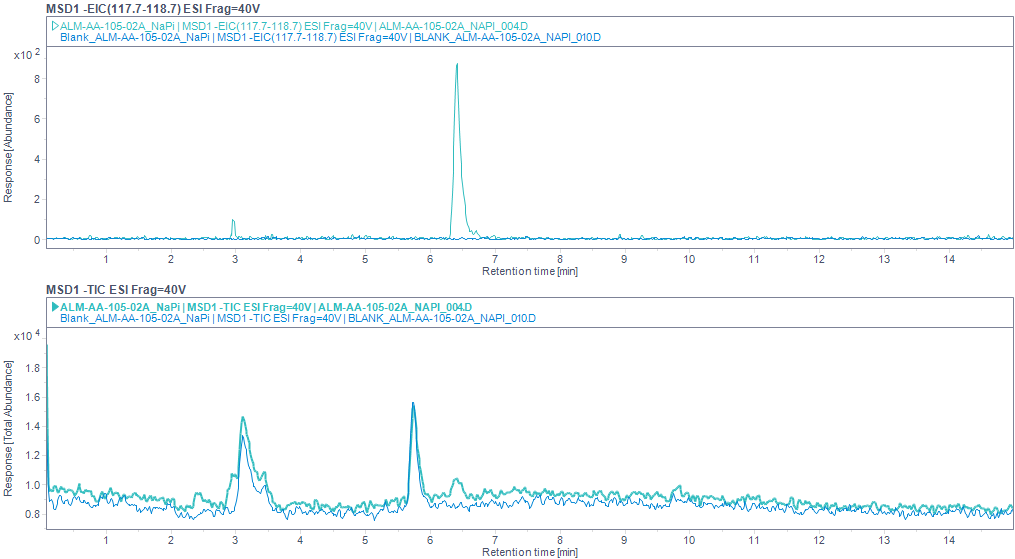


Figure S4.10: Conversion of fumarate by ScCreD according to 4.1. Retention times: 5.7 min fumaric acid, 6.5 min 3-nitropropionic acid. Overlay of traces from analysis of extracted ion (above; M-1 negative mode; m/z 118 for the detection of **11**; blue: reaction control without enzyme, green: biotransformation), and traces from analysis of TIC (below; blue: reaction control without enzyme, green: biotransformation). **Reaction conditions**: [sodium fumarate] = 5 mM, [NaNO_2_] = 25 mM, [NaPi] = 10 mM, [ScCreD] = 10 µM, pH 7.0, 30 °C, 120 rpm, 16 h

### Quantification of nitropropanoic acid by HPLC-UV (analyses performed with **Method UV-01**)


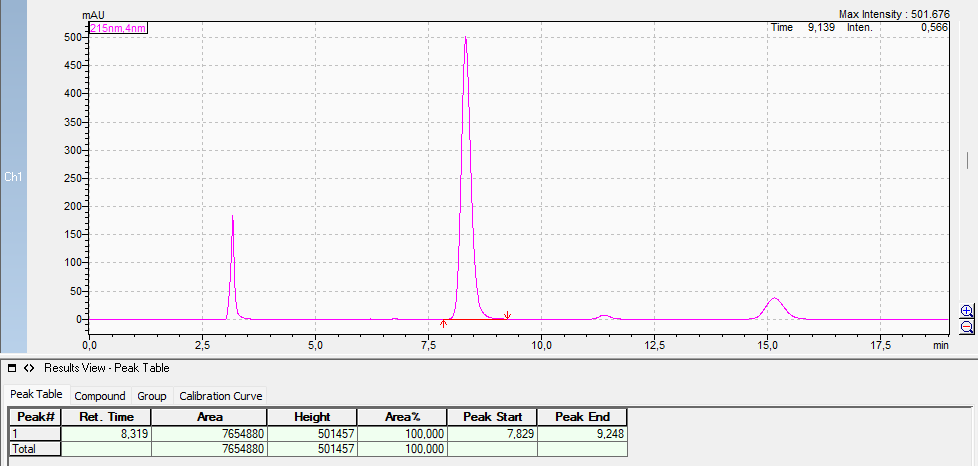


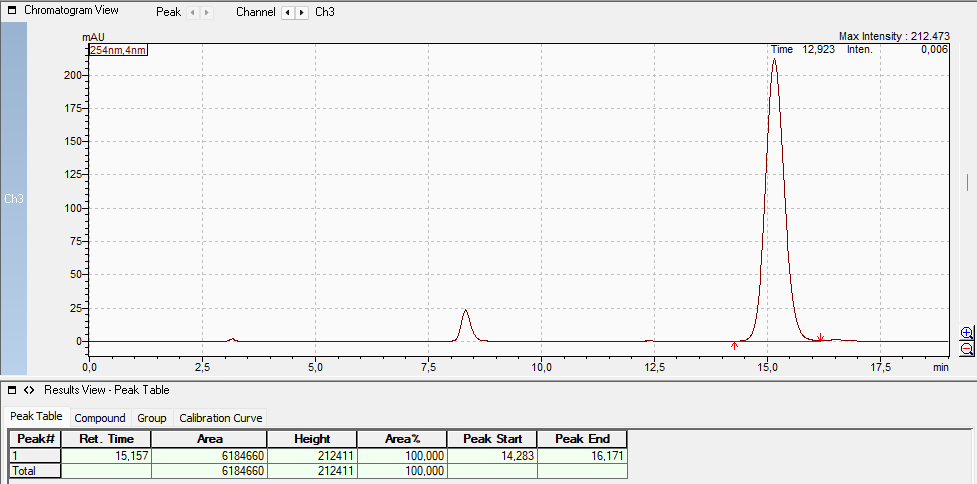


Figure S4.11: Representative chromatogram from a control reaction in absence of enzyme. Retention times: 8.3 min fumaric acid, 15.2 min cis,cis-muconic acid (internal standard). Analysis performed at 215 nm (above), 254 nm (below). **Reaction conditions**: [sodium fumarate] = 50 mM, [NaNO_2_] = 500 mM, [HEPES] = 400 mM, pH 7.0, 30 °C, 120 rpm, 18 h


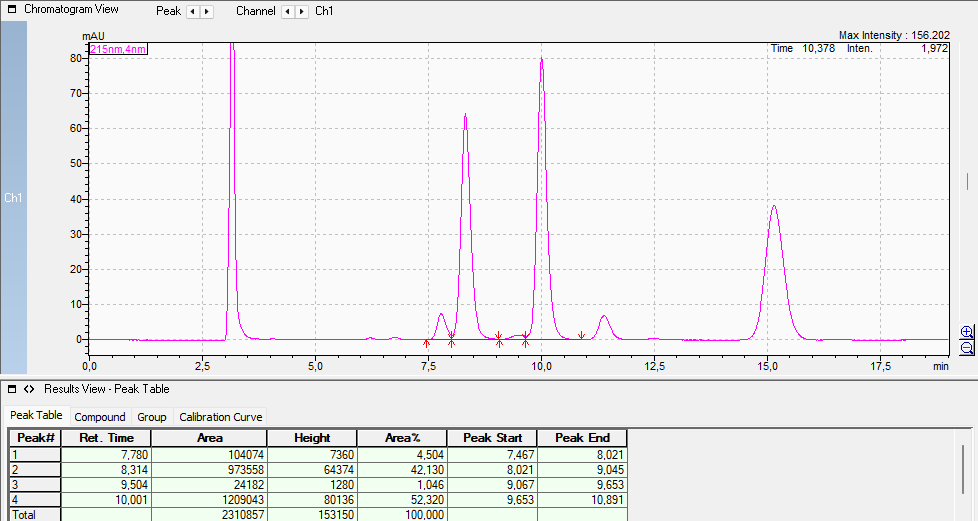


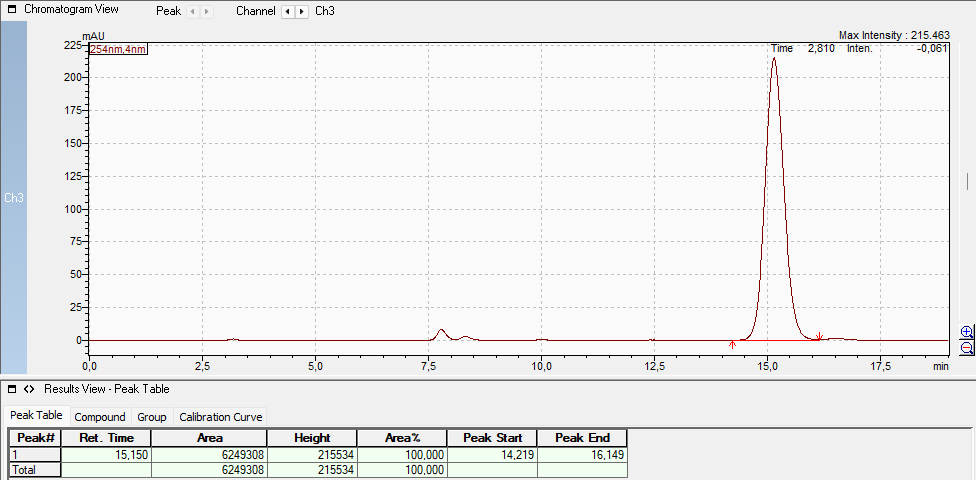


Figure S4.12: Representative chromatogram from a hydronitration reaction catalyzed by ScCreD (89% substrate consumption). Retention times: 8.3 min fumaric acid, 10.0 min 3-nitropropanoic acid, 15.2 min cis,cis-muconic acid. Analysis performed at 215 nm (above), 254 nm (below). **Reaction conditions**: [sodium fumarate] = 50 mM, [NaNO_2_] = 500 mM, [HEPES] = 400 mM, [ScCreD] = 10 µM, pH 7.0, 30 °C, 120 rpm, 18 h


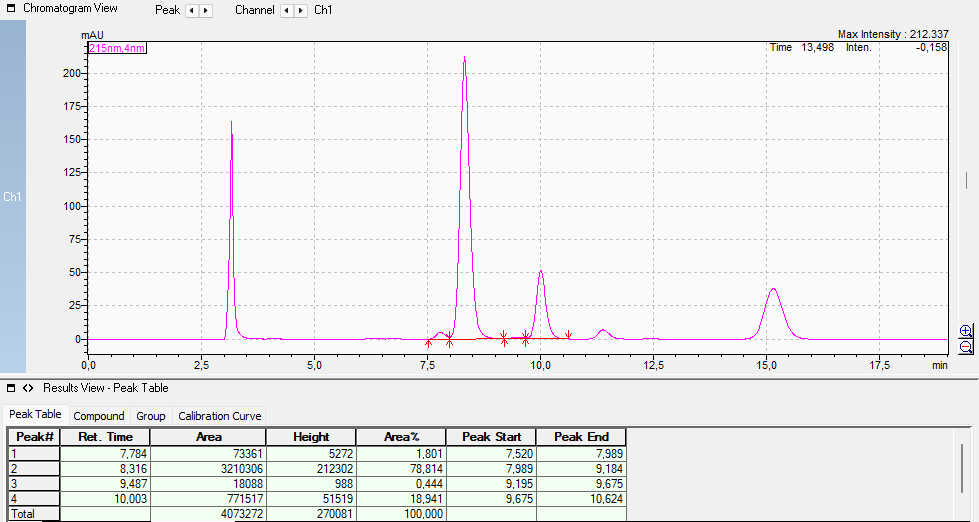


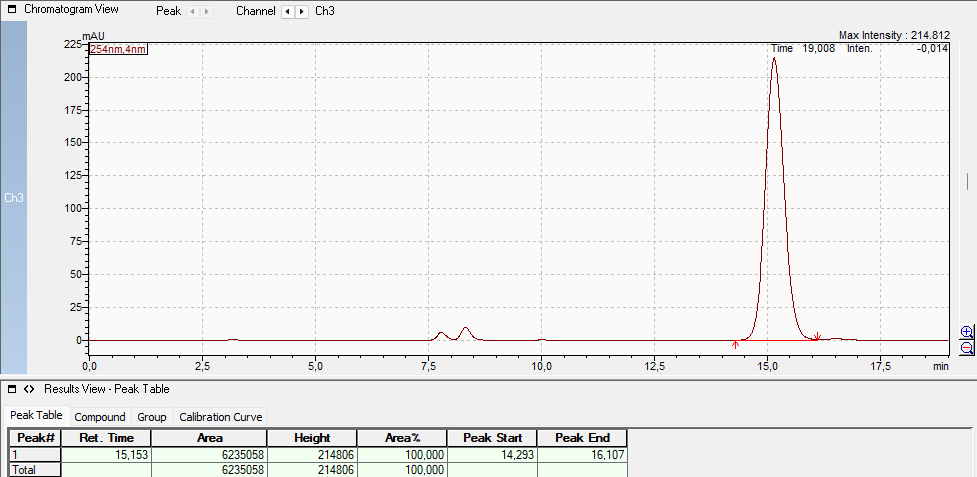


Figure S4.13: Representative chromatogram from a hydronitration reaction catalyzed by ScCreD (63% substrate consumption). Retention times: 8.3 min fumaric acid, 10.0 min 3-nitropropanoic acid, 15.2 min cis,cis-muconic acid (internal standard). Analysis performed at 215 nm (above), 254 nm (below). **Reaction conditions**: [sodium fumarate] = 50 mM, [NaNO_2_] = 500 mM, [HEPES] = 400 mM, [ScCreD] = 1 µM, pH 7.0, 30 °C, 120 rpm, 18 h

## Isolation and characterization of the enzymatic reaction product **11**

In a 50 mL plastic centrifuge tube, 120 mg of sodium fumarate_(s)_ (0.75 mmol, 50 mM), 255 mg of NaNO_2(s)_ (3.7 mmol, 250 mM) and 357 mg of HEPES_(s)_ (100 mM) were solubilized in deionized water (14.5 mL). The pH was then adjusted to 7.0 with NaOH (10 M) and the solution supplemented with *Sc*CreD (480 μL, to reach final concentration if 9 µM). The reaction mixture was incubated in an orbital shaker for 20 h (30 °C, 120 rpm). Then, the reaction was quenched with urea_(aq)_ (7.5 mL, 0.5 M), HCl_(aq)_ (7.0 mL, 1 M) and further incubated (45 °C, 45 min, 300 rpm). A sample of the reaction was taken and analyzed to determine the conversion (method UV-01). Then, the solution was extracted 3 times with EtOAc, washed with brine, dried over anhydrous Na_2_SO_4(s)_, filtered on cotton and the solvent evaporated under reduced pressure. The crude was then purified via column chromatography on silica (cyclohexane/EtOAc 1:1 + 1 vol% AcOH) to deliver a white/pale yellow crystalline solid (11.5 mg, isolated yield 10%). The NMR spectra (Section 10) were in accordance with those of nitropropanoic acid obtained by independent chemical synthesis (Section 9).

## Time-resolved NMR analysis of the enzymatic reaction and intermediate product identification

An enzyme stock solution was prepared from purified enzyme by further concentrating it using a 15 mL Vivaspin 20 centrifugal concentrator tube (30 kDa cut-off) and exchanging the buffer to 20 mM KPi (pH = 8.0) without glycerol.

A KPi buffer solution (150 mM) containing sodium fumarate (100 mM) and NaNO_2_ (100 mM) was prepared using 90 vol% dH_2_O and 10 vol% D_2_O. The pH was adjusted using 10 M NaOH. The enzyme stock solution (10 μM) was added immediately before transfer into an NMR tube and NMR measurement at 700 MHz at 30 °C (total volume 700 μL). A few minutes passed before data acquisition started, as the sample was prepared outside of the NMR instrument.

Product distribution could be followed over time, and representative data are provided in Table S4.1, along with the ratio nitronate/nitropropanoate. The latter was found constant after the onset of the reaction, and correlates well with the expected distribution of the nitropropanoate/nitronate acid/base pair, based on pH (7) and the reported p*K*_a_ value of 9 for the 3-carbon acid, with an expected theoretical *K*_eq_ ~ 0.01.^[13]^ This was further confirmed by incubating nitropropanic acid in the reaction buffer in absence of enzyme: NMR analysis provided a ratio **14**/**11a** of 0.014.

To unequivocally assign the signals to the corresponding nitronate and nitrosuccinate products, the experiments were performed with labeled nitrite (Na^15^NO_2_) under same reaction conditions. Signal splitting shows coupling of H and N.

Relevant NMR data are provided in section 10, Figures S10.5-10.7.

Table S4.1: Compound distribution in % (equivalent to mM given the starting concentration 100 mM) as measured by NMR.

| Time  (h) | Fumarate  (1a) | Nitrosuccinate  (5a) | Nitronate  (14) | Nitropropanoate  (11a) | 14/11a ratio |
| --- | --- | --- | --- | --- | --- |
| Start | 98.06 | 0.04 | 0.04 | 1.86 | 0.021 |
| 1.7 | 87.38 | 0.03 | 0.10 | 12.50 | 0.008 |
| 3.3 | 80.18 | 0.02 | 0.16 | 19.64 | 0.008 |
| 5 | 74.46 | 0.01 | 0.22 | 25.32 | 0.009 |

## Michaelis-Menten kinetics

### Time study

The reaction mixture was prepared by solubilizing in deionized water, in order: sodium fumarate_(s)_ (to reach final concentration of 50 mM), NaNO_2(s)_ (to reach final concentration of 500 mM), HEPES_(s)_ (to reach final concentration of 400 mM), and the pH was adjusted to 7.0 with NaOH (10 M). This solution was supplemented with purified *Sc*CreD (to reach final concentration of 10 μM). The reactions (final volume 500 µL) were incubated in a bench-top shaker for varying periods of time at 30 °C (300 rpm). Then, 25 µL of the reaction were mixed with urea_(aq)_ (125 µL, 0.5 M; spiked with 5 mM IS), HCl_(aq)_ (100 µL, 1 M) and incubated in a thermoshaker (45 °C, 45 min, 300 rpm). The reactions were centrifuged (18,000 rpm, 2 min) and filtered with cotton. The samples were analyzed with HPLC-UV (Method UV-01) and the product formation was quantified. The reactions were performed in triplicates. It could be observed that the reaction proceeded linearly along the time axis for about 1 h and this time window was chosen to calculate initial rates (Table S4.2 and Figure S4.14).

Table S4.2: time study of the ScCreD-catalyzed hydronitration reaction

| Time  (min) | [11] (mM) |
| --- | --- |
| 2 | 0.45 ± 0.10 |
| 5 | 0.67 ± 0.19 |
| 10 | 1.25 ± 0.31 |
| 15 | 2.66 ± 0.44 |
| 30 | 2.56 ± 0.92 |
| 60 | 8.51 ± 0.75 |
| 120 | 12.99 ± 0.56 |
| 180 | 18.44 ± 2.25 |
| 1440 | 41.41 ± 0.54 |

Figure S4.14: Time profile of the hydronitration reaction based on product formation. **Reaction conditions**: [1a] = 50 mM, [ScCreD] = 10 µM, [NaNO_2_] = 0.5 M, [HEPES] = 400 mM, pH 7.0, 30 °C.

### Time study with varying NaNO_2_ concentrations

The reaction samples were prepared as under 4.6.1, except that sodium nitrite was added to reach final concentration respectively of 0.5, 1.0, 1.5 M.

The determination of the kinetic parameters (*K*_m_ and *k*_cat_) was performed with 1 M of NaNO_2_ since the velocity of the reaction was the highest at that concentration (Figure S4.14). The following values were obtained: 0.1401 mM/min (at 0.5 M NaNO_2_), 0.1568 mM/min (at 1.0 M NaNO_2_), 0.0831 mM/min (at 1.5 M NaNO_2_).

● 0.5 M NaNO_2_ y = 0.1401x + 0.2467

● 1.0 M NaNO_2_ y = 0.1568x - 0.7203

● 1.5 M NaNO_2_ y = 0.0831x + 0.1258

Figure S4.15: Time profile of the hydronitration at varying NaNO_2_ concentrations. **Reaction conditions**: [**1a**] = 50 mM, [ScCreD] = 10 µM, [NaNO_2_] = 0.5, 1.0, 1.5 M, [HEPES] = 400 mM, pH 7.0, 30 °C.

### Determination of *K*_m_ (fumarate) and *k*_cat_ of *Sc*CreD

The reactions were performed as reported under 4.6.1 at fixed nitrite concentration (1 M, see 4.6.2), fixed enzyme concentration, and at varying fumarate concentrations. The reaction velocities *v* were determined from the average of triplicates over reaction time of 1 h (Figure S4.15). The data were analyzed using the software SigmaPlot 16.0 (Figure S4.16).

*Figure S4.16: Time profile of product formation over 1 hour obtained at increasing concentration of sodium fumarate within the range 50 – 550 mM.* ***Reaction conditions****: [****1a****] = 50 – 550 mM, [ScCreD] = 10 µM, [NaNO_2_] = 1.0 M, [HEPES] = 400 mM, pH 7.0, 30 °C. [****11****]****^1^****: the biotransformations were performed in triplicates and indicated as arithmetic average; standard deviations reported* *in Figure S4.16 for clarity.*

| \| [1a]  (mM) \| *v*  (mM/min) \| \| --- \| --- \| \| 50 \| 0.16 ± 0.01 \| \| 125 \| 0.33 ± 0.06 \| \| 200 \| 0.50 ± 0.05 \| \| 250 \| 0.51 ± 0.04 \| \| 325 \| 0.63 ± 0.10 \| \| 375 \| 0.57 ± 0.16 \| \| 450 \| 0.57 ± 0.05 \| \| 550 \| 0.57 ± 0.05 \| \|  \|  \|   *v_max_* 0.78 ± 0.09 μmol/min  *K*_m_ 143 ± 50 mM  *k*_cat,app_ 1.3 ± 0.2 s^-1^ |  |
| --- | --- | --- | --- | --- | --- | --- | --- | --- | --- | --- | --- | --- | --- | --- | --- | --- | --- | --- | --- | --- | --- |

Figure S4.17: Michaelis-Menten plot for the determination of the kinetic parameters (k_cat,app_ and K_m_).

### Preparation of calibration curve for **11**

The calibration curve (Figure S4.17) for the quantification of **11** for the determination of *K*_m_ and *k_cat_* was prepared as follows. A solution was prepared by mixing 3-nitropropanoic acid_(s)_ (24.5 mg, to reach 50 mM) in reaction buffer containing NaNO_2(aq)_ (to reach final 0.5 M), HEPES_(aq)_ (to reach final concentration 400 mM), and the pH adjusted to 7.0 with NaOH (10 M). This solution was diluted 1:2, 1:5, 1:10, 1:50, 1:500. The samples (final volume 500 µL) were supplemented with *Sc*CreD storage buffer (20 mM HEPES, pH 8.0, 20% glycerol) to obtain respectively the final concentrations of 50, 25, 10, 5, 1, 0.1 mM. The samples were incubated for 1 h (30 °C, 120 rpm). Then, 25 µL were worked up as explained in Section 4.6.1 and analysed with HPLC-UV (Method UV-01) for the quantification of **11**. Samples were prepared in triplicates.

Figure S4.18: Calibration curve for the quantification of 3-nitropropanoic acid under reaction conditions used for the determination of the kinetic parameters; **Normalized area^1^**: the calibration points were plotted as arithmetic average of triplicates calculated as [counts]_3-nitropropanic acid_/[counts]_IS_. The error bars are the standard deviation between the triplicates.

# Scope of catalytic promiscuity in the aspartase/fumarase superfamily

## Experimental procedure – hydronitration reaction

The reaction mixtures were prepared by combining in deionized water, in order: sodium fumarate_(s)_ (to reach final concentration of 50 mM), NaNO_2(s)_ (to reach final concentration of 500 mM), HEPES_(s)_ (to reach final concentration of 400 mM). The pH was adjusted to 7.0 with NaOH (10 M) and the enzyme solution was added to reach final concentration of 10 μM in a total volume of 500 µL. The reactions were incubated in an orbital shaker for 18 h at 30 °C (120 rpm). Then, 25 µL of the reaction were worked up according to the standard procedure (see Section 4.6) and **11** and **1** were quantified with HPLC-UV (Method UV-01). The side product **4** was quantified with HPLC-MS (Method MS-02) following dilution (1:2) of the samples with urea_(aq)_ (0.5 M; spiked with 5 mM IS). The reactions were performed in triplicates (average value reported with standard deviation).

## Preparation of the calibration curves - hydronitration reaction

### Sodium fumarate and 3-nitropropanoic acid

The calibration curves were obtained according to the following procedures. A solution containing sodium fumarate_(s)_ or 3-nitropropanoic acid_(s)_ (50 mM) in a mixture of *Sc*CreD storage buffer and reaction buffer containing NaNO_2(aq)_ (final concentration 0.5 M) and HEPES_(aq)_ (final concentration 400 mM) was prepared, and the pH adjusted to 7.0 with NaOH (10 M). The ratio of *Sc*CreD storage buffer and reaction buffer was the same as in the biotransformations. This solution was diluted with the storage/reaction buffer mixture to reach the desired concentrations, as shown in Figure S5.1 and Figure S5.2. The samples were incubated for 3 h (30 °C, 120 rpm) and 25 μL of the reaction were worked up according to the standard procedure (see Section 4.2) and **11** and **1** were quantified with HPLC-UV (Method UV-01).

### Malic acid

The calibration curve for the side product **4** was prepared as described in Section 5.3.1, using *rac*-malic acid as the analyte. The samples were then diluted 1:2 with the urea workup solution (0.5 M, spiked with *cis,cis*-muconic acid (IS)) and analyzed by HPLC–MS (Method MS-02).

### Calibration curves

| \| [1a]  (mM) \| Normalized area^1^ \| \| --- \| --- \| \| 0.5 \| 0.0140 ± 0.0001 \| \| 0.75 \| 0.0210 ± 0.0006 \| \| 1 \| 0.0300 ± 0.0003 \| \| 2.5 \| 0.0753 ± 0.0009 \| \| 5 \| 0.1487 ± 0.0009 \| \| 10 \| 0.3033 ± 0.0004 \| \| 25 \| 0.7554 ± 0.0048 \| \| 50 \| 1.4884 ± 0.0097 \| |  |
| --- | --- | --- | --- | --- | --- | --- | --- | --- | --- | --- | --- | --- | --- | --- | --- | --- | --- | --- | --- |

Figure S5.1: Calibration curve for fumarate. Left: table with (normalized area) vs [sodium fumarate]; Right: plot of the calibration points in the linear range. **^1^**: the calibration points were plotted as arithmetic average of triplicates calculated as [counts]_sodium fumarate_/[counts]_IS_. The error bars are the standard deviation between the triplicates. If not displayed error bars are smaller than the data points.

| \| [11]  (mM) \| Normalized area^1^ \| \| --- \| --- \| \| 0.1 \| 0.0005 ± 0.00004 \| \| 0.25 \| 0.0011 ± 0.00004 \| \| 0.5 \| 0.0021 ± 0.00014 \| \| 0.75 \| 0.0033 ± 0.0001 \| \| 1 \| 0.0048 ± 0.00012 \| \| 2.5 \| 0.0122 ± 0.00012 \| \| 5 \| 0.0251 ± 0.00031 \| \| 10 \| 0.0527 ± 0.00014 \| \| 25 \| 0.1365 ± 0.0011 \| \| 50 \| 0.2794 ± 0.0004 \| |  |
| --- | --- | --- | --- | --- | --- | --- | --- | --- | --- | --- | --- | --- | --- | --- | --- | --- | --- | --- | --- | --- | --- | --- | --- |

Figure S5.2: Calibration curve for nitropropanoic acid. Left: table with (normalized area) vs [3-nitropropanoic acid]; Right: plot of the calibration points in linear range. **^1^**: the calibration points were plotted as arithmetic average of triplicates calculated as [counts]_3-nitropropanoic acid_/[counts]_IS_. The error bars are the standard deviation between the triplicates. If not displayed error bars are smaller than the data points.

| \| [4]  (mM) \| Normalized area^1^ \| \| --- \| --- \| \| 0.1 \| 0.17 ± 0.01 \| \| 5 \| 7.28 ± 0.22 \| \| 10 \| 12.97 ± 0.09 \| \| 25 \| 28.18 ± 0.59 \| \| 50 \| 47.91 ± 1.69 \| |  |
| --- | --- | --- | --- | --- | --- | --- | --- | --- | --- | --- | --- | --- | --- |

Figure S5.3: Calibration curve for malic acid. Left: table with (normalized area) vs [malic acid]; Right: plot of the calibration points in linear range. **^1^**: the calibration points were plotted as arithmetic average of triplicates calculated as [counts]_malic acid_/[counts]_IS_. The error bars are the standard deviation between the triplicates. If not displayed error bars are smaller than the data points.

## Results

Table S5.1: Results for the catalytic promiscuity study.

|  |  | Nitration |  |  | Amination |  | Hydration |  |
| --- | --- | --- | --- | --- | --- | --- | --- | --- |
| Enzyme | [**1**]  (mM) | [**11**] (mM) | [**4**] (mM) | [**1**]  (mM) | [**4**]  (mM) | [**6**]  (mM) | [**1**]  (mM) | [**4**]  (mM) |
| *Sc*CreD | 5.1 ± 0.1 | 40.9 ± 0.4 | traces | 48.0 | n.d. | 0.1 | 50.0 ± 1.7 | 0.2 |
| *Sd*CreD | 9.9 ± 0.2 | 29.3 ± 0.2 | 1.0 ± 0.1 | 49.7 ± 1.1 | traces | traces | 48.8 | traces |
| *S*spFzmL | 4.4 | 41.5 ± 0.5 | traces | 49.9 ± 0.3 | n.d. | traces | 46.9 ± 0.4 | traces |
| *Ka*AzpD | 5.1 ± 0.1 | 41.4 ± 0.9 | traces | 50.6 ± 0.3 | n.d. | traces | 47.8 ± 0.7 | traces |
| *Pa*FlcC | 45.1 ± 1.7 | 1.0 | traces | 50.9 | n.d. | traces | 49.3 ± 1.1 | 0.1 |
| *Pp*CMLE | 44.4 ± 0.04 | 0.8 ± 0.1 | traces | 49.9 ± 0.3 | n.d. | 0.10 | 50.0 ± 0.5 | traces |
| *B*spAspB | 43.9 ± 1.1 | n.d. | 0.1 ± 0.1 | traces | n.d. | 46.9 ± 1.4 | 47.6 ± 0.7 | 2.3 ± 0.1 |
| *Hsn*ADSL | 43.9 ± 0.6 | n.d. | 0.1 ± 0.1 | 50.2 ± 0.3 | traces | traces | 49.4 ± 2.0 | 1.0 |
| *Mt*ASL | 44.4 ± 0.5 | n.d. | traces | 50.2 ± 0.2 | n.d. | traces | 47.7 ± 1.7 | 0.1 |
| *Ec*FumC | 8.4 | n.d. | 38.1 ± 0.2 | 10.0 ± 0.4 | 35.3 ± 2.1 | 1.8 ± 0.1 | 9.4 ± 0.3 | 42.0 ± 0.6 |
| Control | 46.8 ± 0.2 | n.d. | n.d. | 51.5 ± 0.3 | n.d. | n.d. | 51.0 ± 0.3 | n.d. |

Traces: below the quantification limit (0.1 mM). n.d. not detected. If no deviation standard is indicated, RMSD < 0.1 mM. The control reaction was performed in the conditions of the biotransformations in absence of enzyme. All the biotransformation were performed in triplicates.

## Chromatograms

### HPLC-UV traces (analyses performed with **Method UV-01**)


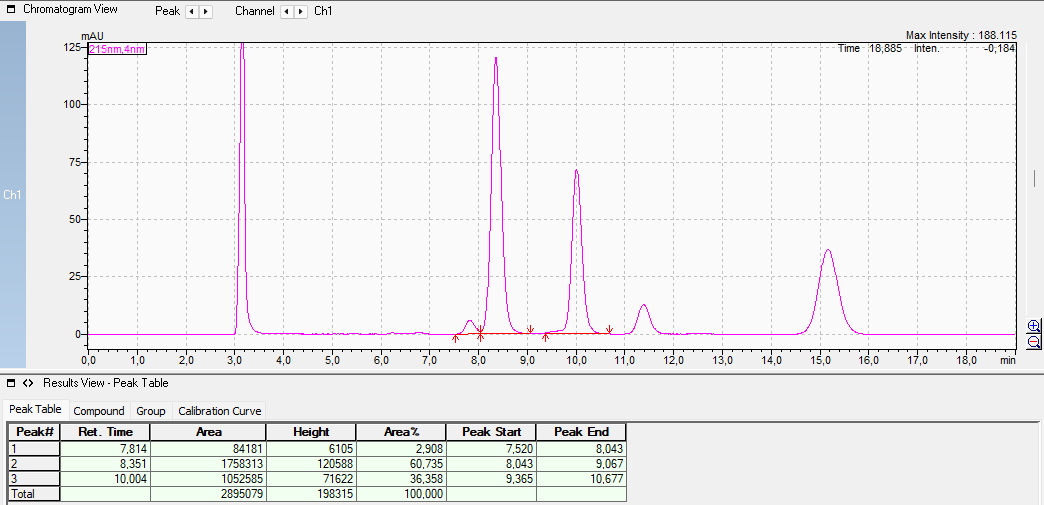


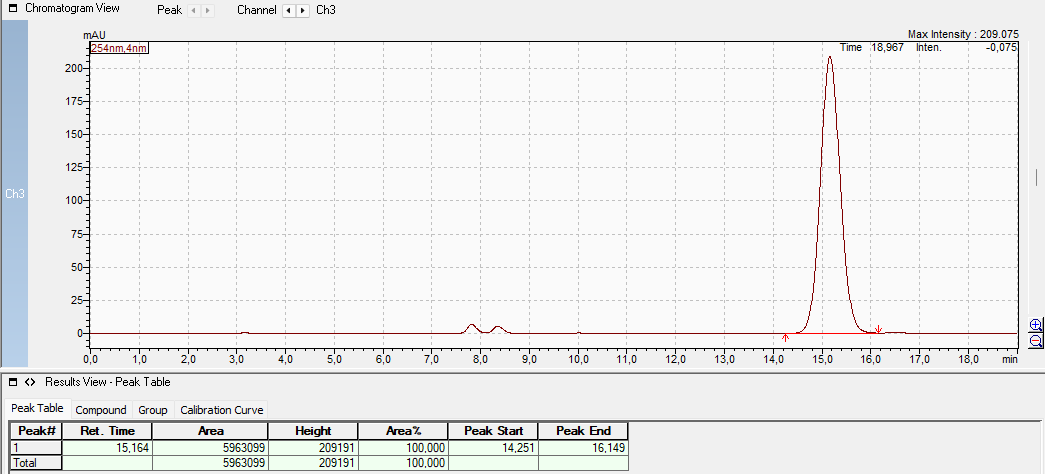


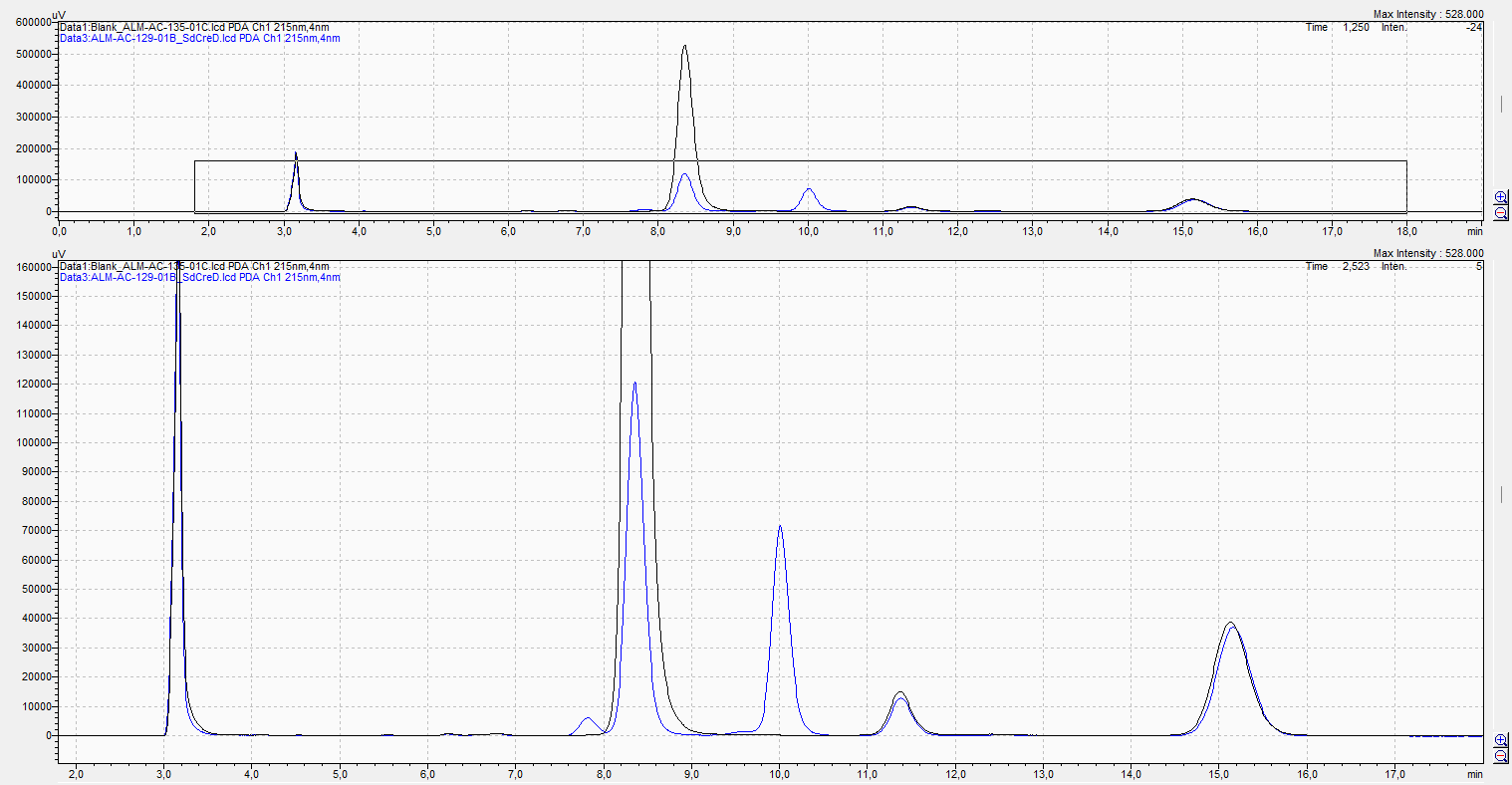


Figure S5.4: Hydronitration reaction catalysed by SdCreD. Retention times: 8.3 min fumaric acid, 10.0 min 3-nitropropanoic acid, 15.2 min cis,cis-muconic acid (IS). Analysis performed at 215 nm (above), 254 nm (middle), overlay at 215 nm (bottom; black: reaction control without enzyme, blue: biotransformation)


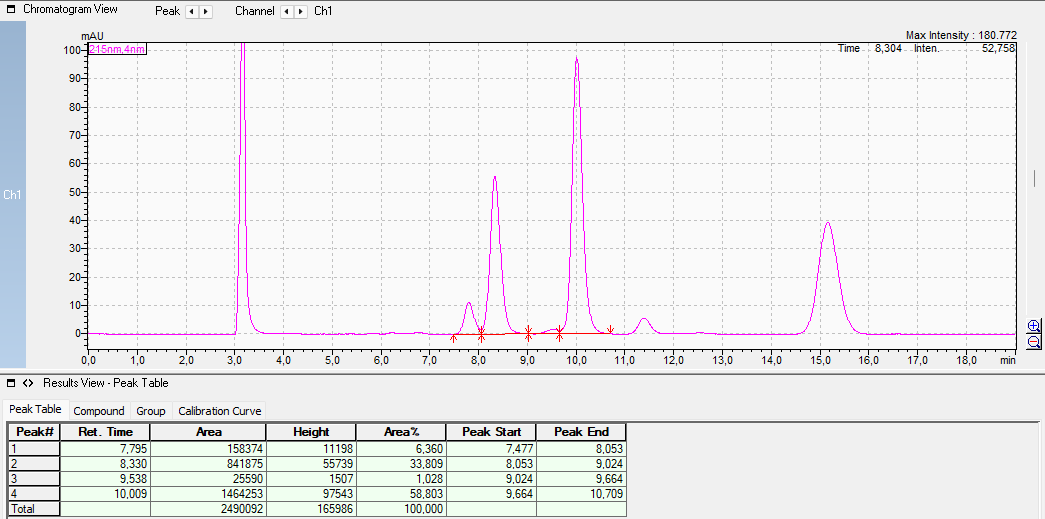


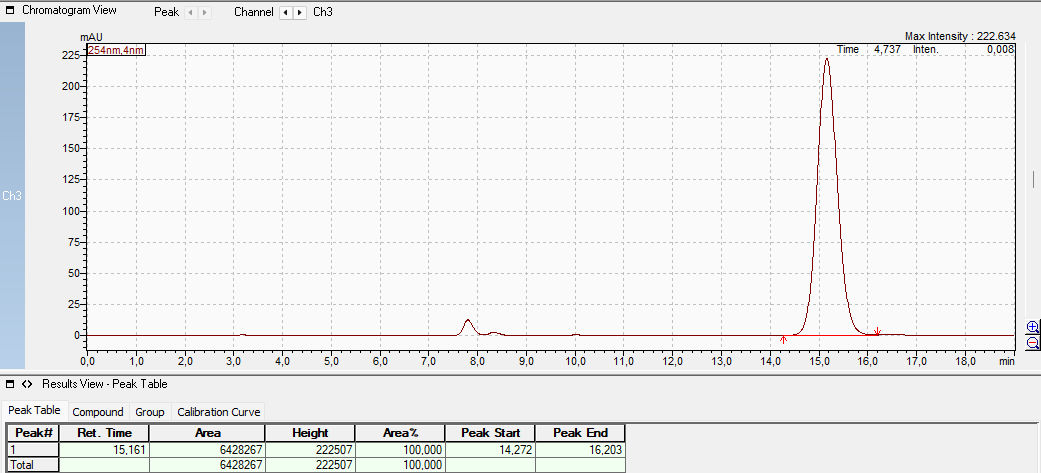


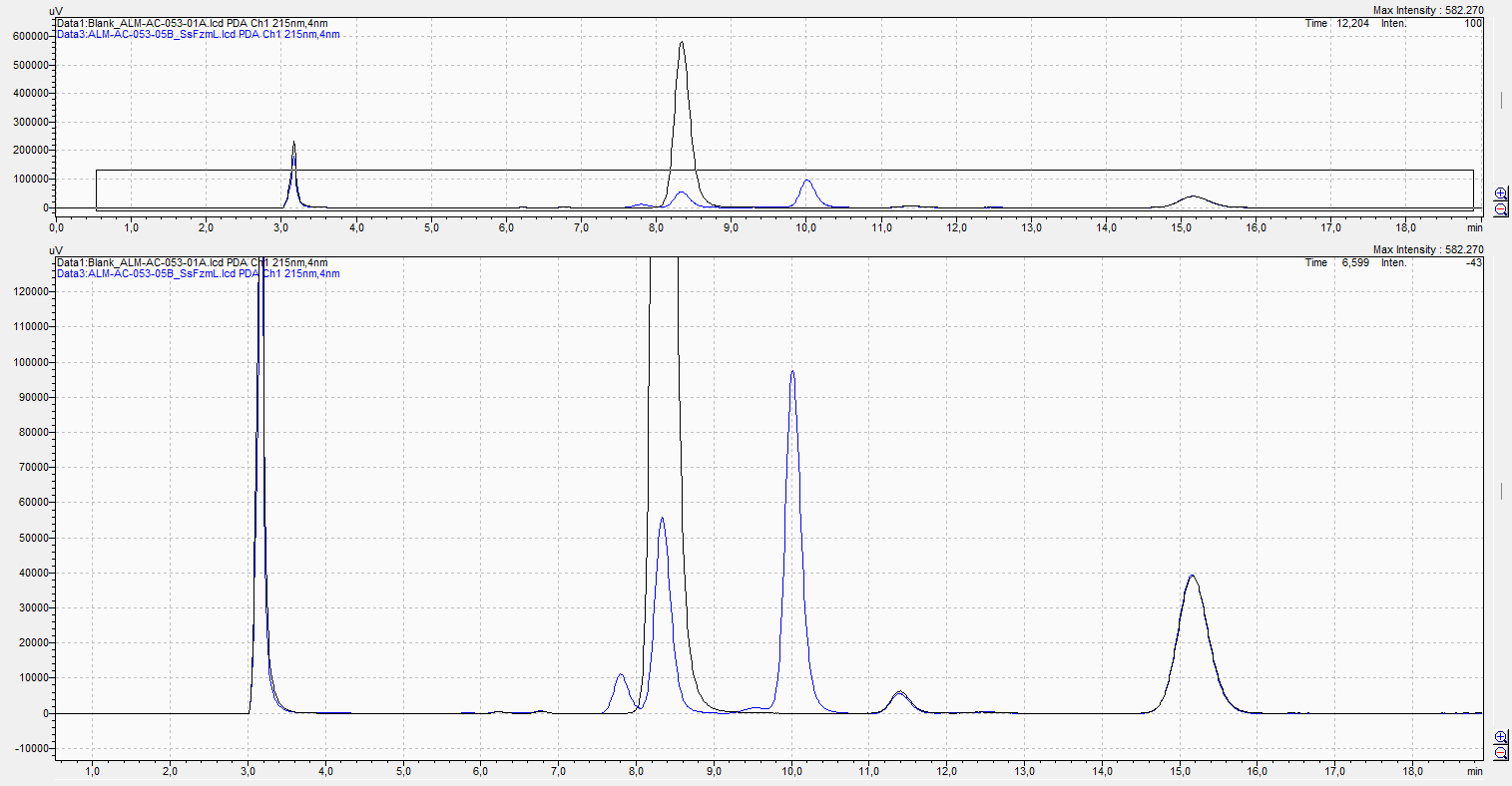


Figure S5.5: Hydronitration reaction catalysed by SsFzmL. Retention times: 8.3 min fumaric acid, 10.0 min 3-nitropropanoic acid, 15.2 min cis,cis-muconic acid (IS). Analysis performed at 215 nm (above), 254 nm (middle), overlay at 215 nm (bottom; black: reaction control without enzyme, blue: biotransformation)


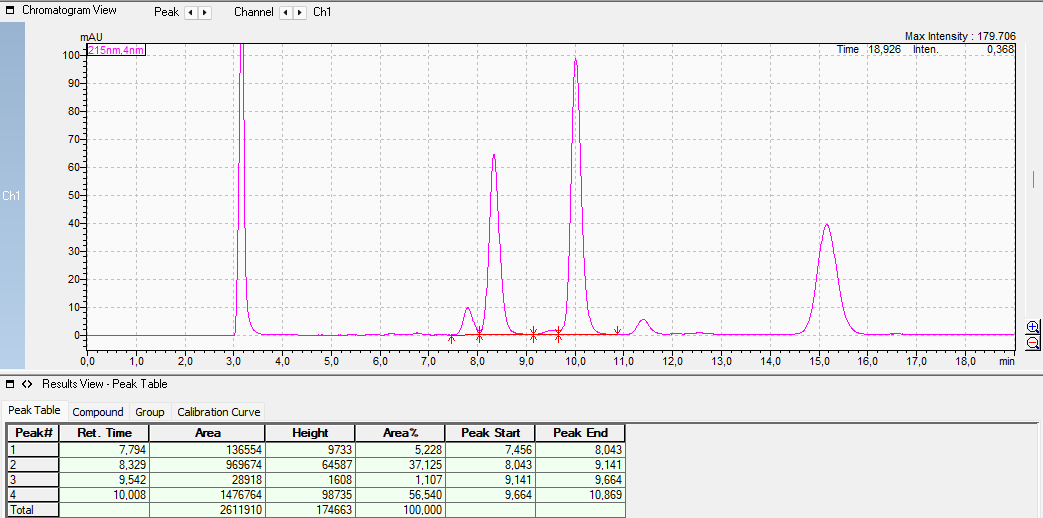

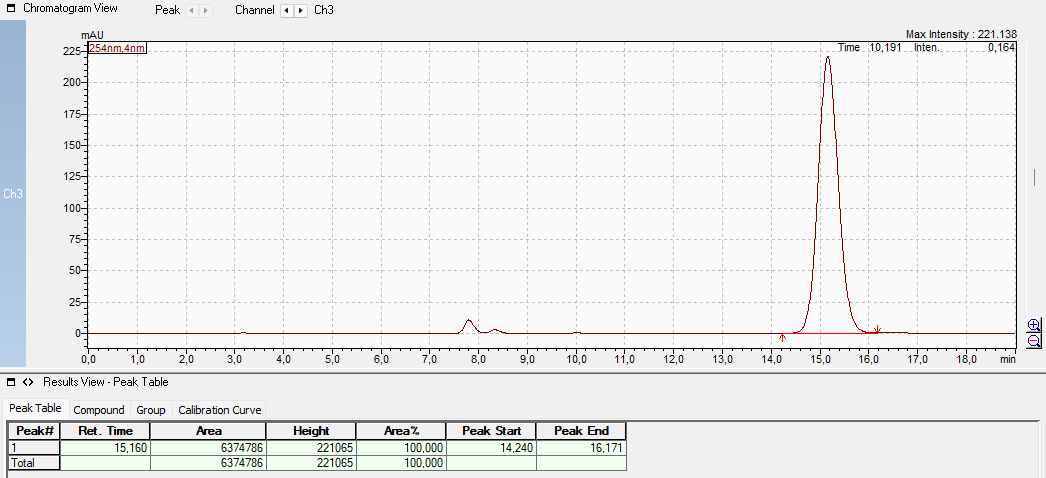

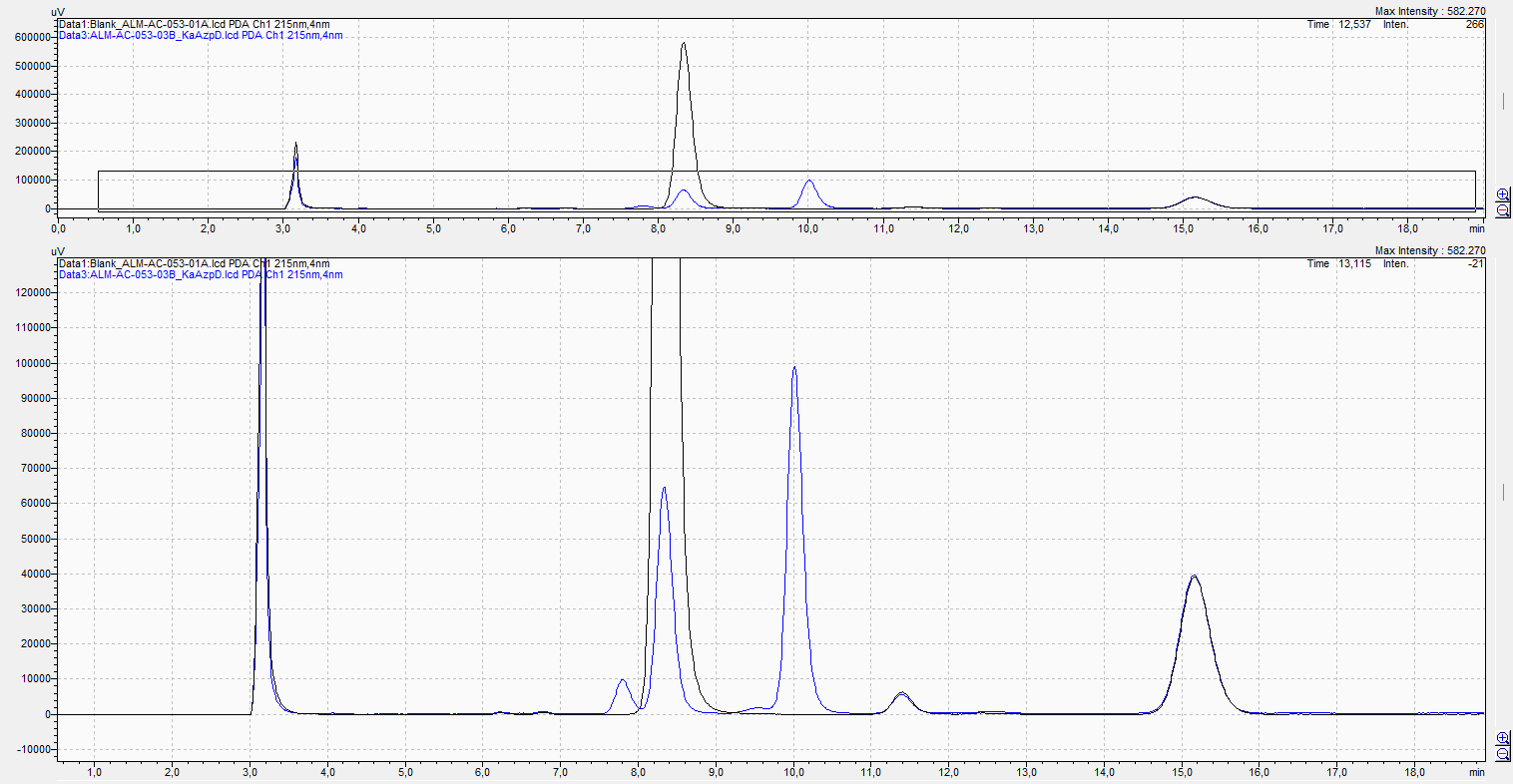


Figure S5.6: Hydronitration reaction catalysed by KaAzpD. Retention times: 8.3 min fumaric acid, 10.0 min 3-nitropropanoic acid, 15.2 min cis,cis-muconic acid (IS). Analysis performed at 215 nm (above), 254 nm (middle), overlay at 215 nm (bottom; black: reaction control without enzyme, blue: biotransformation)


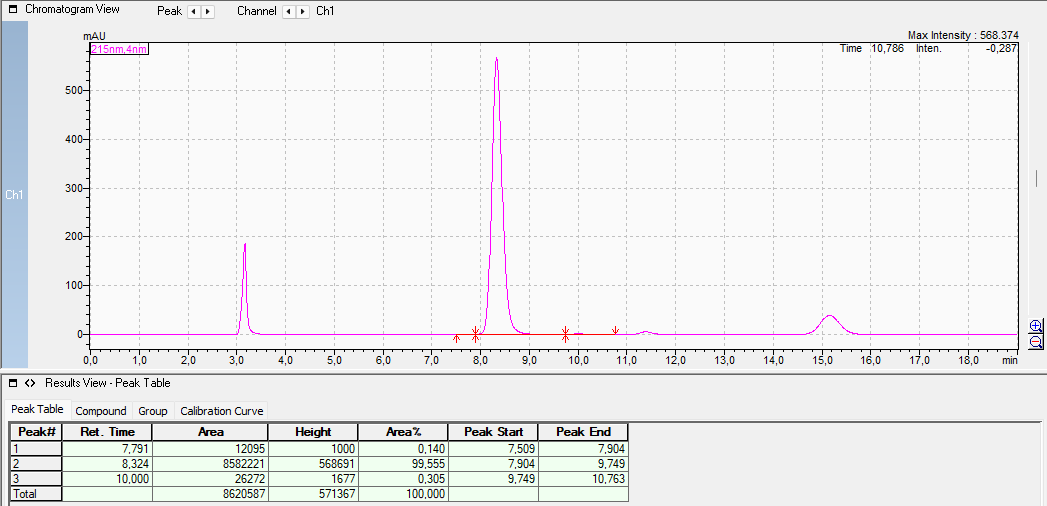

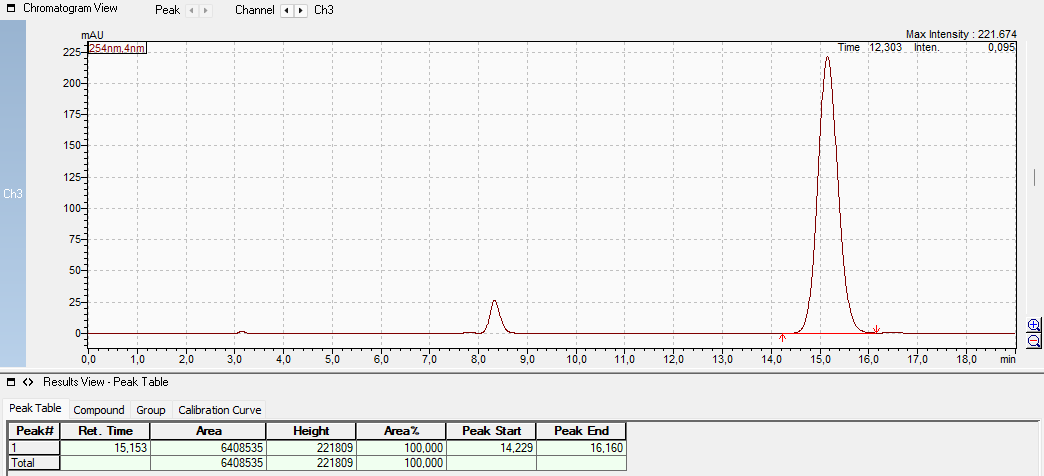

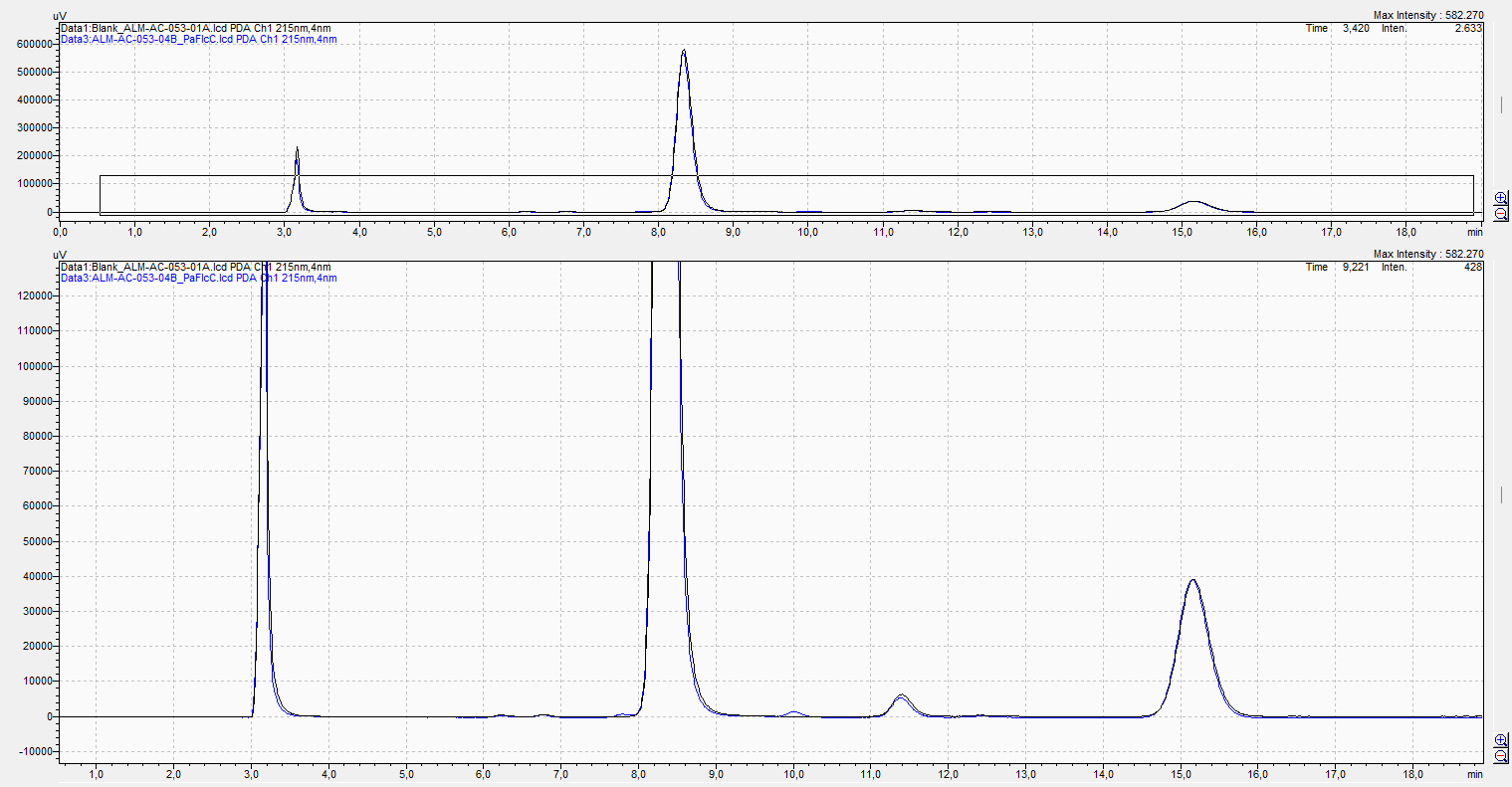


Figure S5.7: Hydronitration reaction catalysed by PaFlcC. Retention times: 8.3 min fumaric acid, 10.0 min 3-nitropropanoic acid, 15.2 min cis,cis-muconic acid (IS). Analysis performed at 215 nm (above), 254 nm (middle), overlay at 215 nm (bottom; black: reaction control without enzyme, blue: biotransformation)


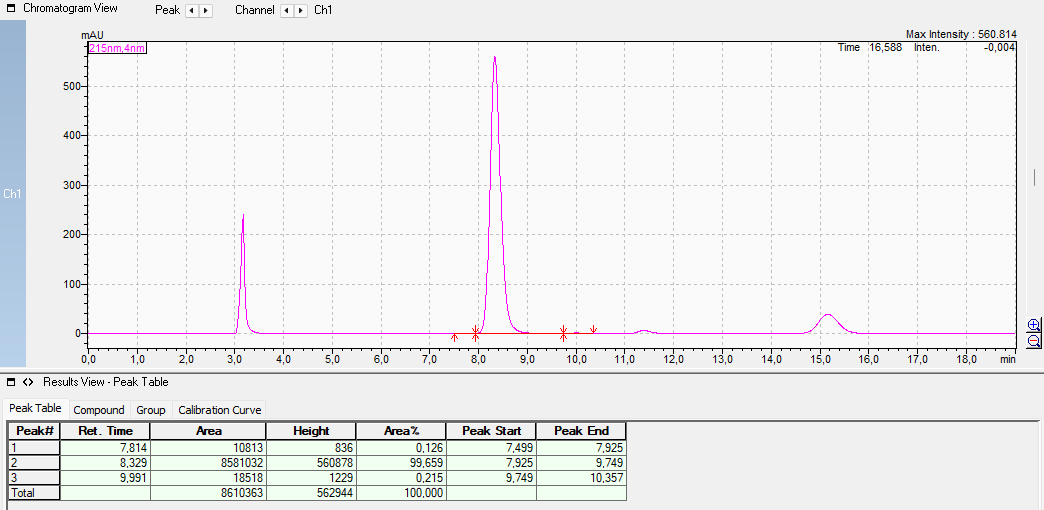

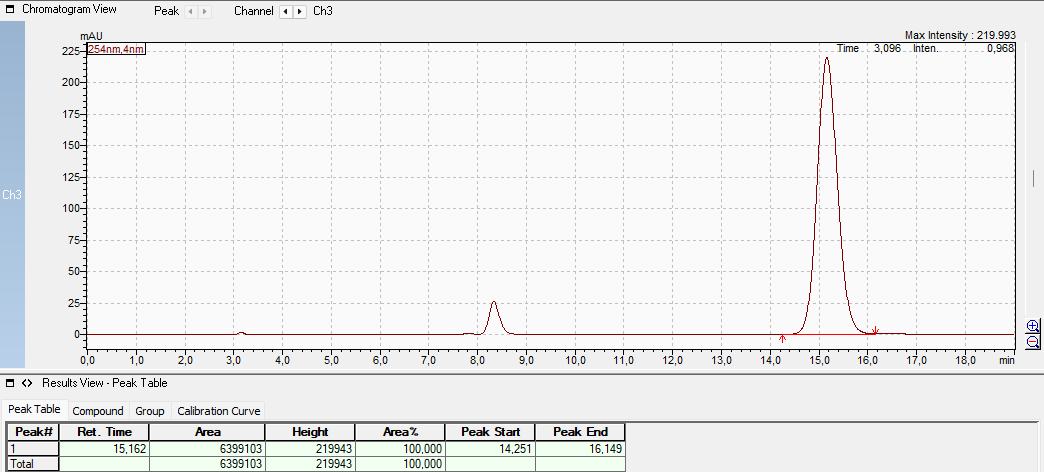

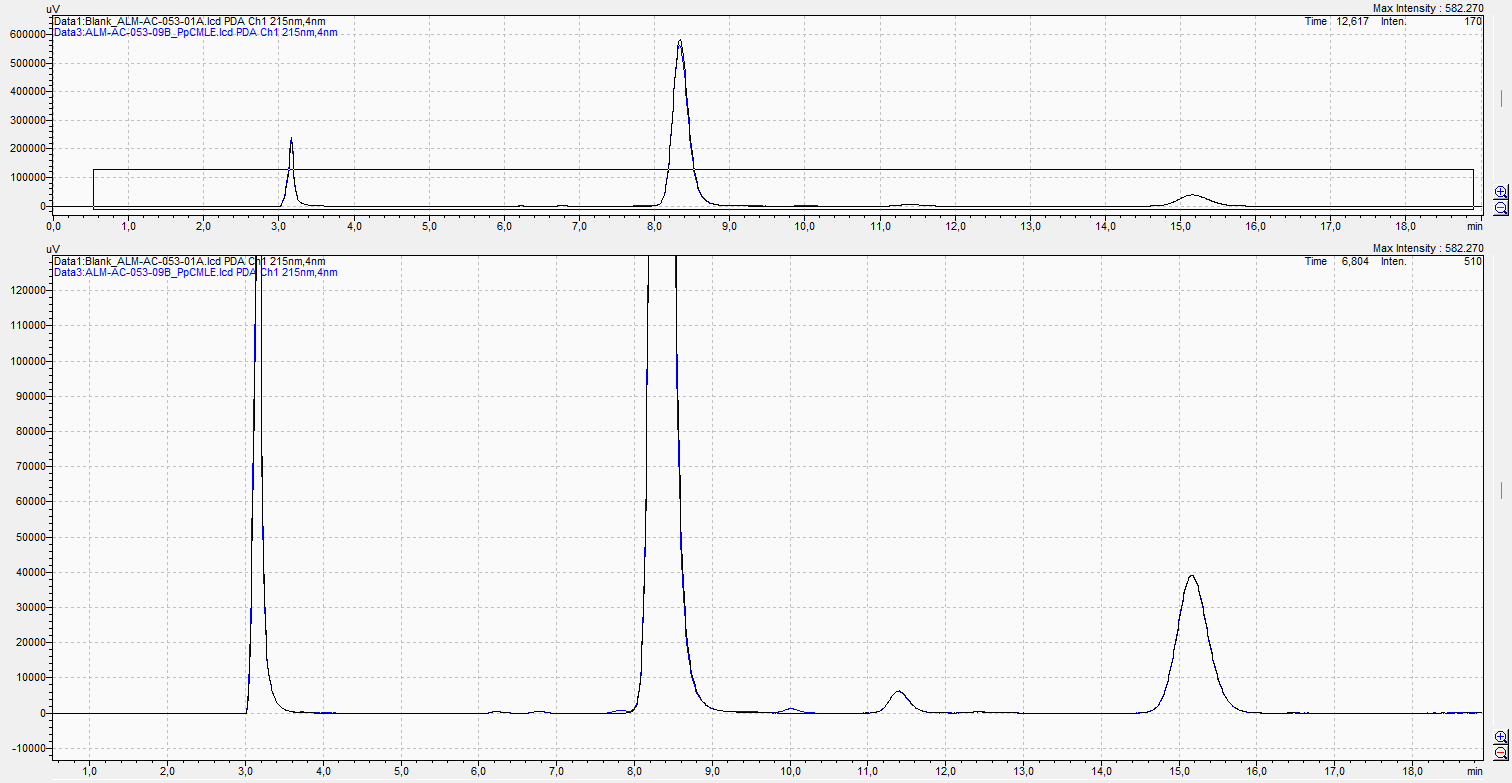


Figure S5.8: Hydronitration reaction catalysed by PpCMLE. Retention times: 8.3 min fumaric acid, 10.0 min 3-nitropropanoic acid, 15.2 min cis,cis-muconic acid (IS). Analysis performed at 215 nm (above), 254 nm (middle), overlay at 215 nm (bottom; black: reaction control without enzyme, blue: biotransformation)


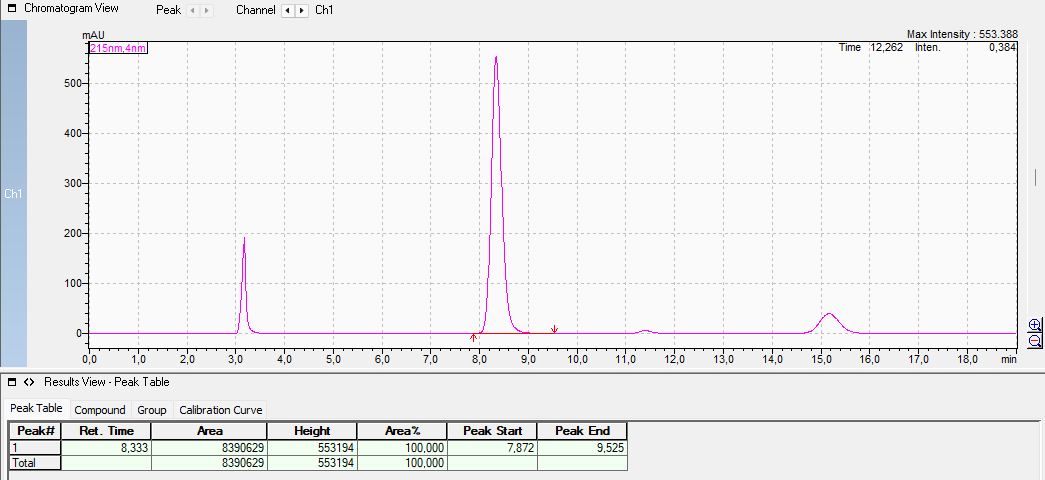

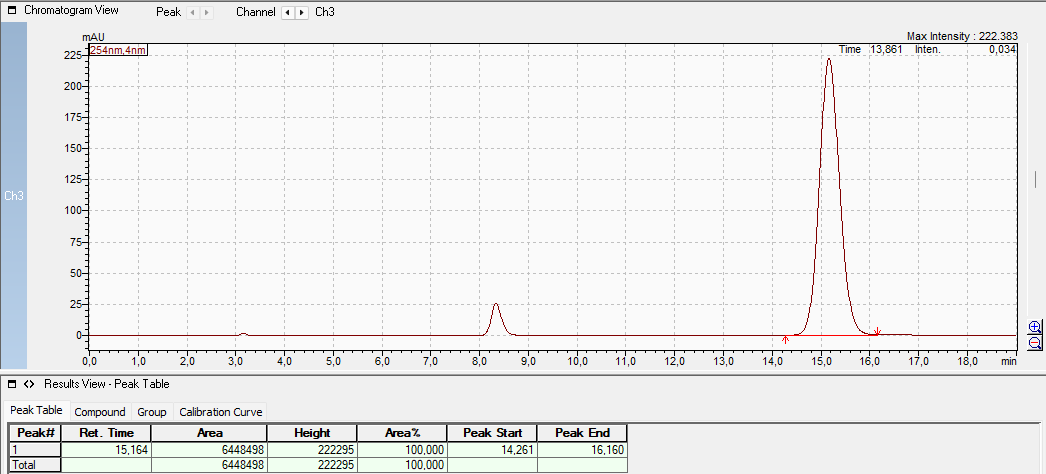

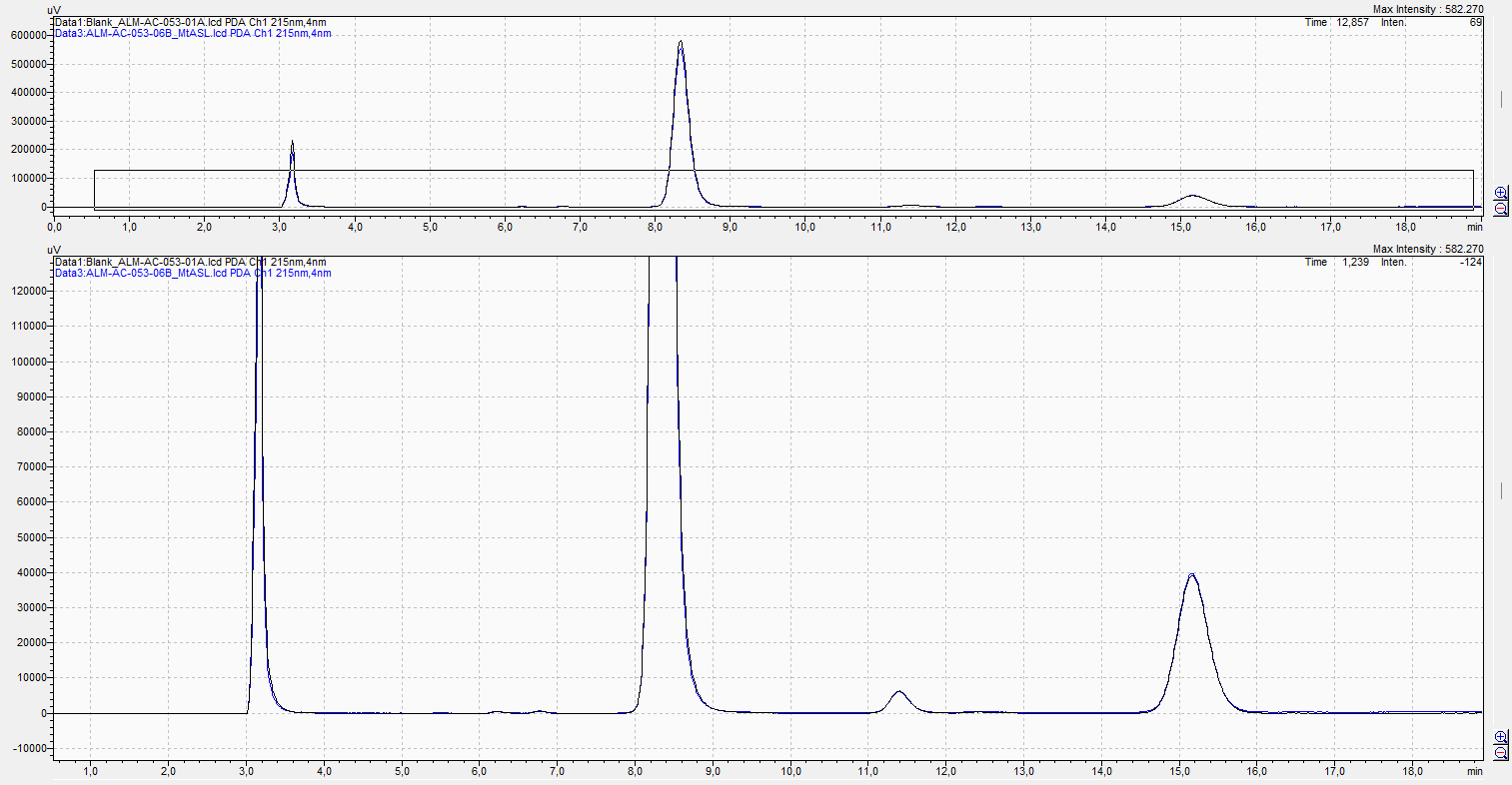


Figure S5.9: Hydronitration reaction catalysed by MtASL. Retention times: 8.3 min fumaric acid, 10.0 min 3-nitropropanoic acid, 15.2 min cis,cis-muconic acid (IS). Analysis performed at 215 nm (above), 254 nm (middle), overlay at 215 nm (bottom; black: reaction control without enzyme, blue: biotransformation)

### HPLC-MS traces (analyses performed with **Method MS-02**)


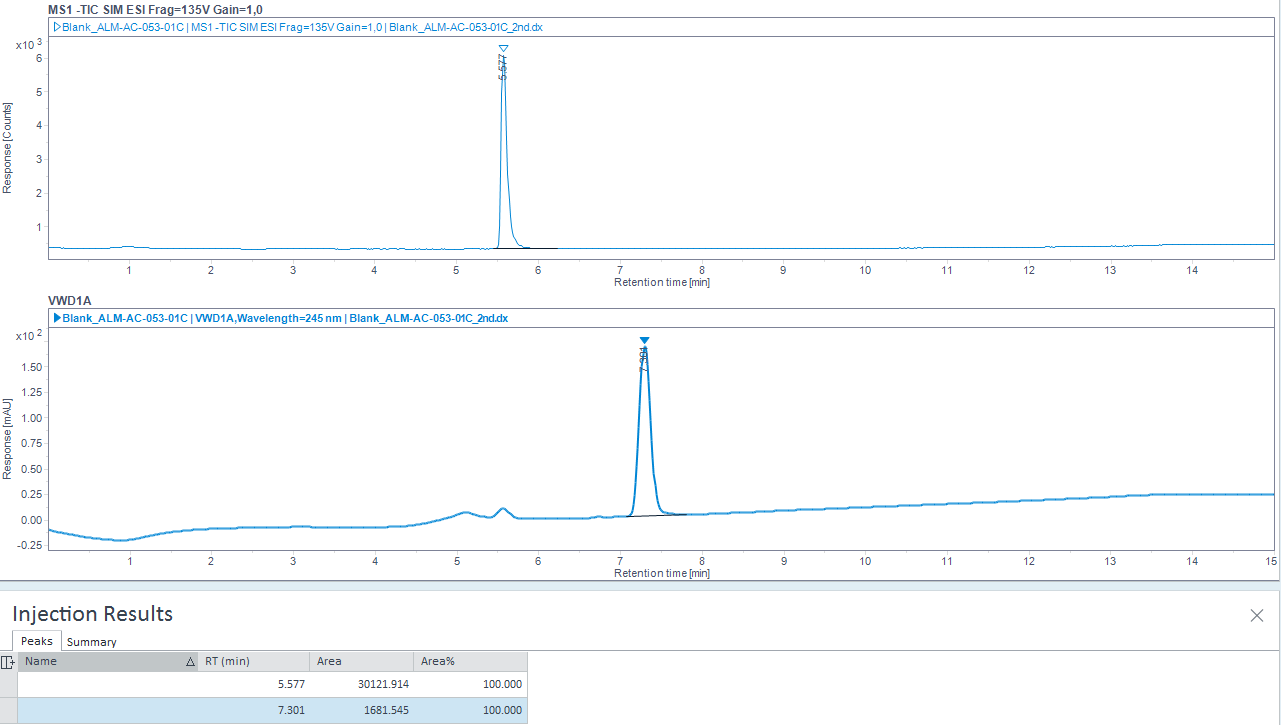


Figure S5.10: Hydronitration reaction - reaction control. Retention times: 5.6 min fumaric acid, 7.3 min cis,cis-muconic acid (IS). Analysis performed in SIM mode (top; M-1, negative mode; m/z 115, 133 for the detection of malic acid), and at 245 nm (bottom).


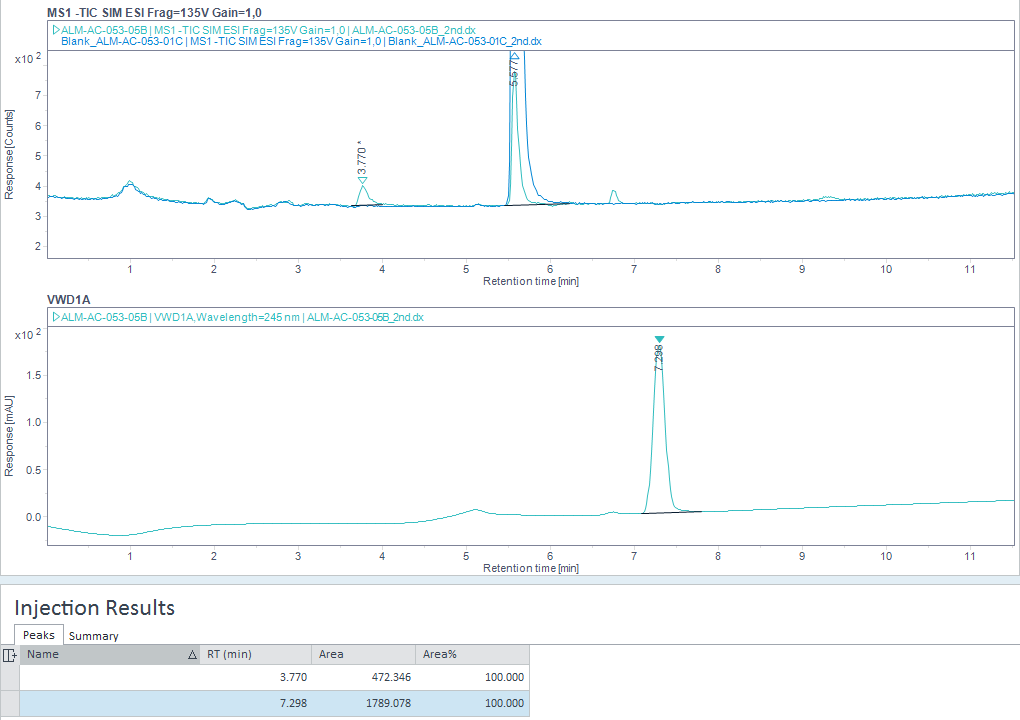


Figure S5.11: Hydronitration reaction catalysed by SsFzmL. Retention times: 3.8 malic acid, 5.6 min fumaric acid, 7.3 min cis,cis-muconic acid (IS). Analysis performed in SIM mode (top; M-1, negative mode; m/z 115, 133 for the detection of malic acid) with overlay (blue: reaction control without enzyme, green: biotransformation), and at 245 nm (bottom).


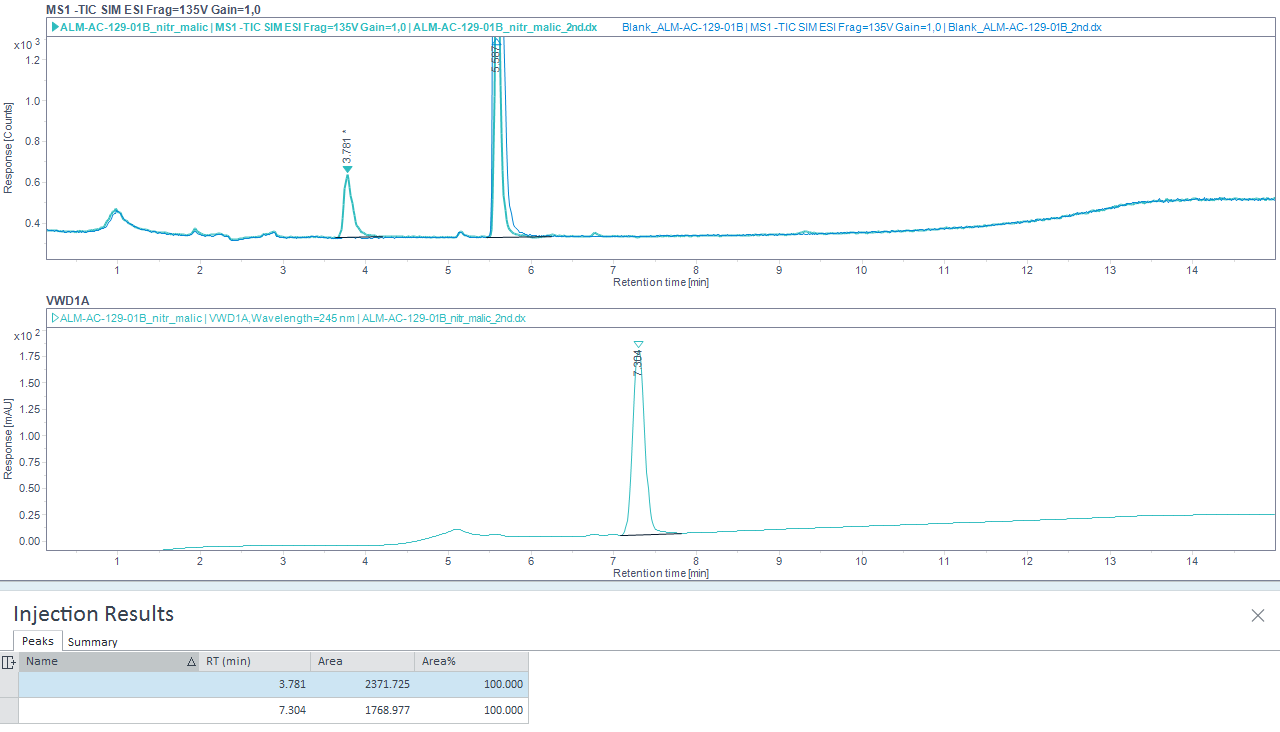


Figure S5.12: Hydronitration reaction catalysed by SdCreD. Retention times: 3.8 malic acid, 5.6 min fumaric acid, 7.3 min cis,cis-muconic acid (IS). Analysis performed in SIM mode (top; M-1, negative mode; m/z 115, 133 for the detection of malic acid) with overlay (blue: reaction control without enzyme, green: biotransformation), and at 245 nm (bottom).


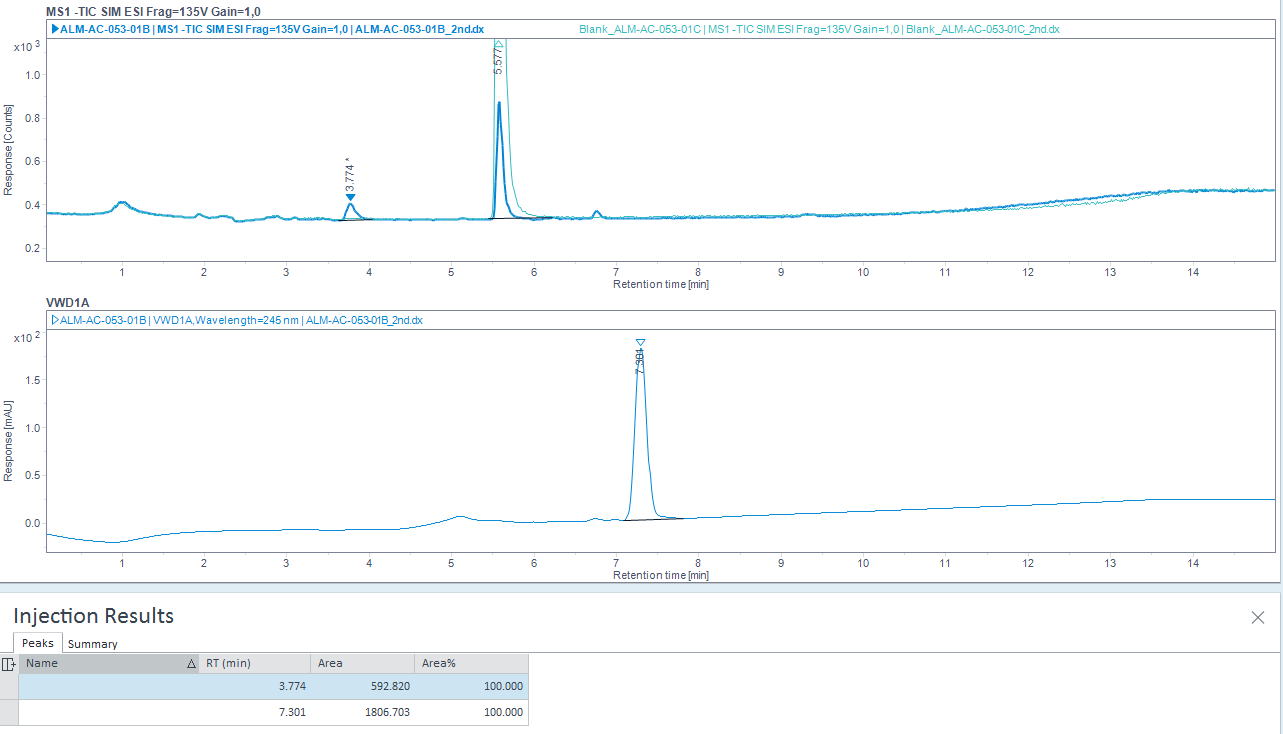


Figure S5.13: Hydronitration reaction catalysed by ScCreD. Retention times: 3.8 malic acid, 5.6 min fumaric acid, 7.3 min cis,cis-muconic acid (IS). Analysis performed in SIM mode (top; M-1, negative mode; m/z 115, 133 for the detection of malic acid) with overlay (green: reaction control without enzyme, blue: biotransformation), and at 245 nm (bottom).


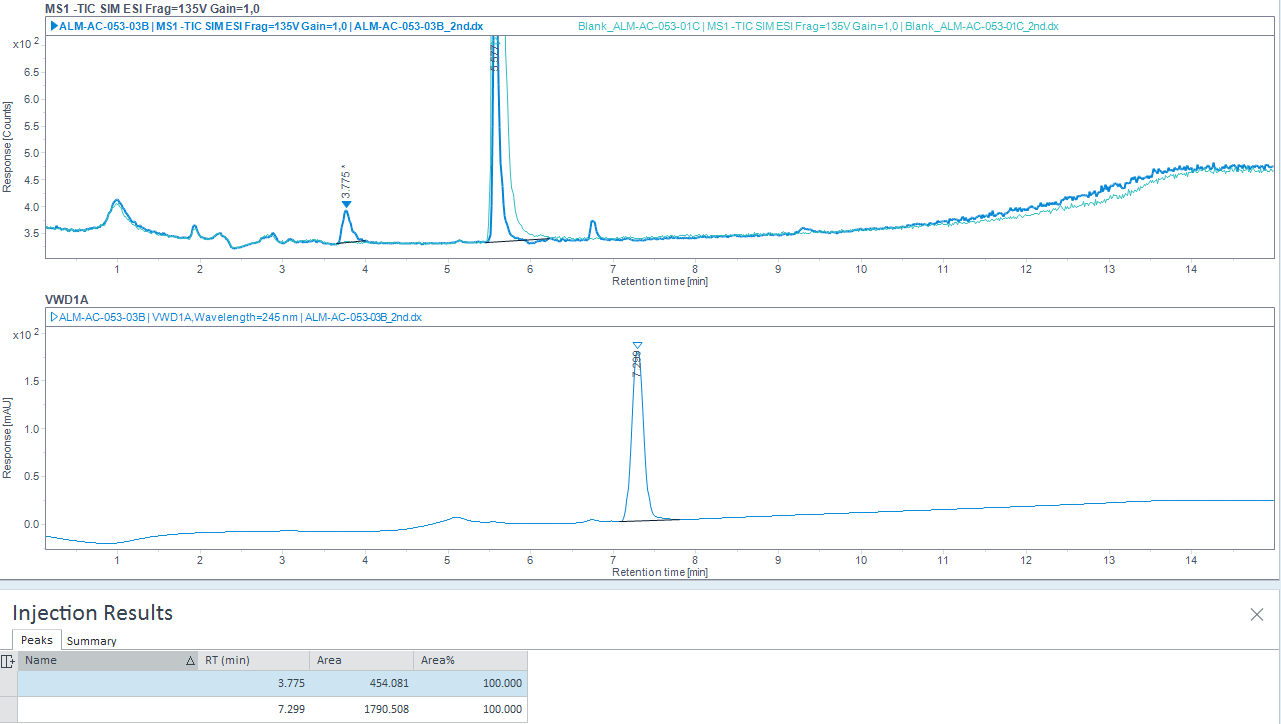


Figure S5.14: Hydronitration reaction catalysed by KaAzpD. Retention times: 3.8 malic acid, 5.6 min fumaric acid, 7.3 min cis,cis-muconic acid (IS). Analysis performed in SIM mode (top; M-1, negative mode; m/z 115, 133 for the detection of malic acid) with overlay (green: reaction control without enzyme, blue: biotransformation), and at 245 nm (bottom).


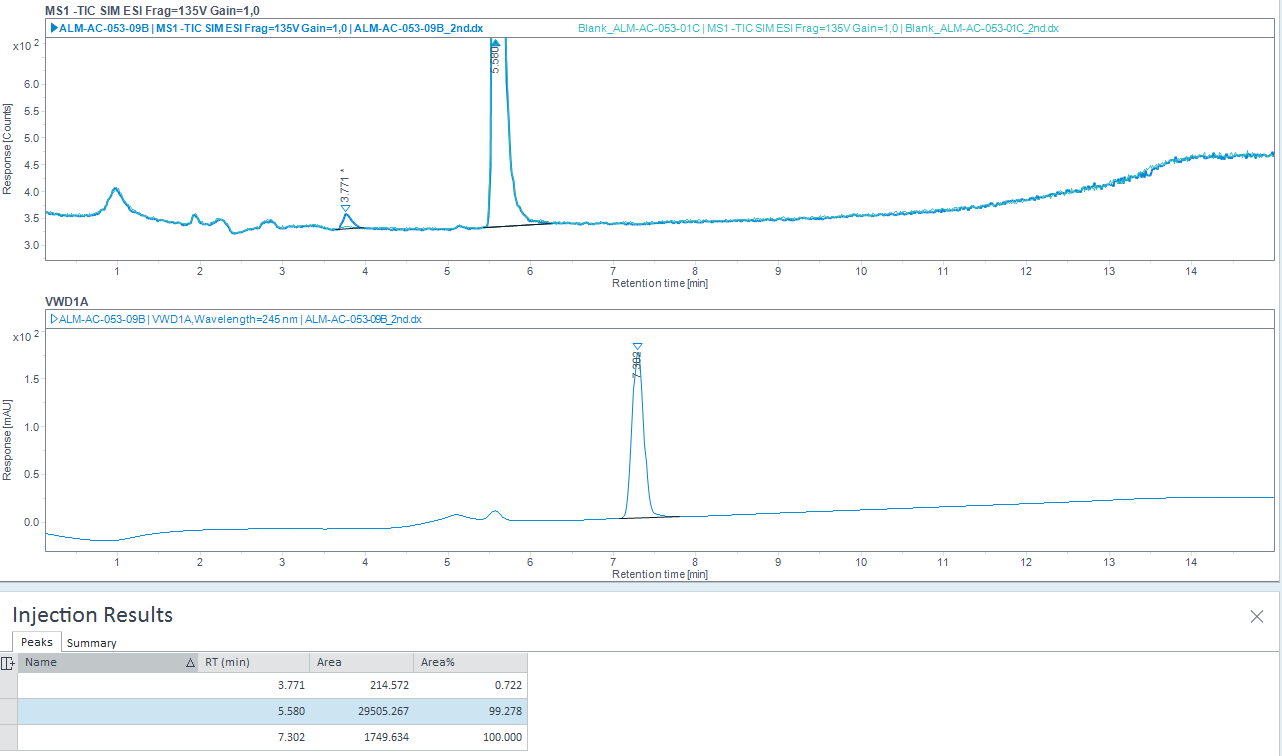


Figure S5.15: Hydronitration reaction catalysed by PpCMLE. Retention times: 3.8 malic acid, 5.6 min fumaric acid, 7.3 min cis,cis-muconic acid (IS). Analysis performed in SIM mode (top; M-1, negative mode; m/z 115, 133 for the detection of malic acid) with overlay (green: reaction control without enzyme, blue: biotransformation), and at 245 nm (bottom).


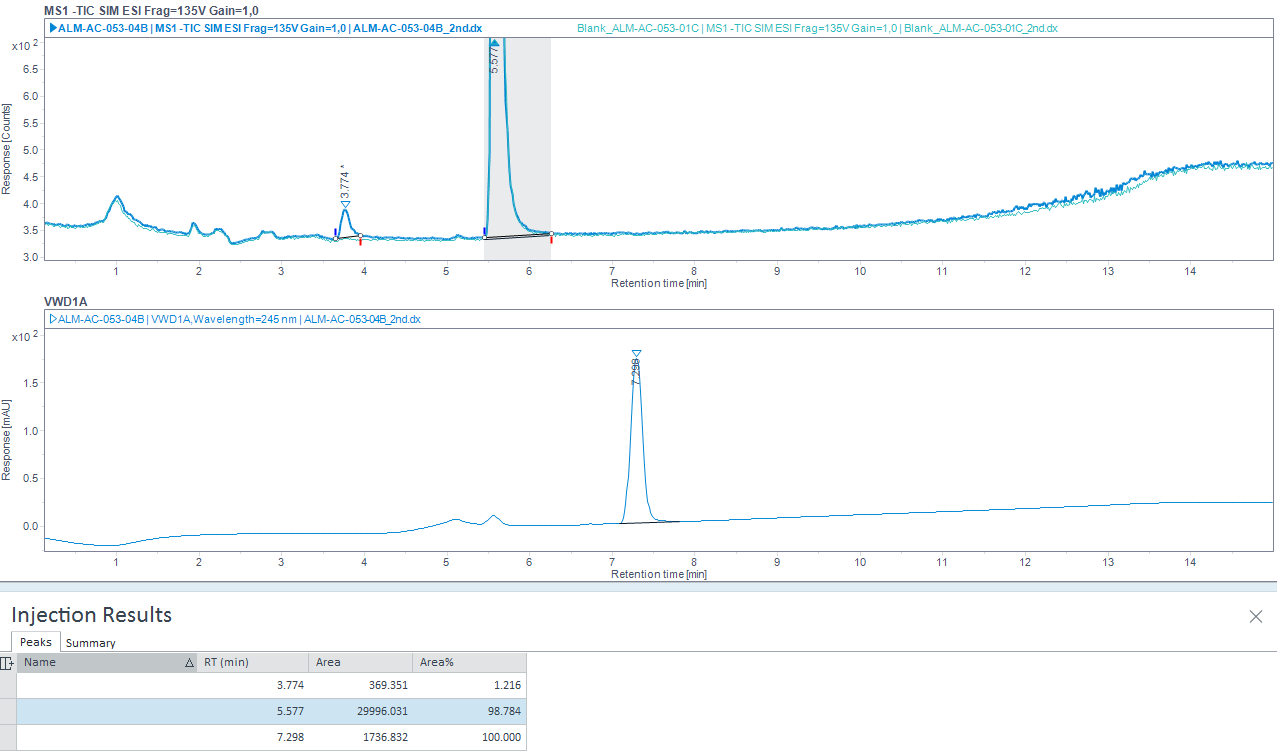


Figure S5.16: Hydronitration reaction catalysed by PaFlcC. Retention times: 3.8 malic acid, 5.6 min fumaric acid, 7.3 min cis,cis-muconic acid (IS). Analysis performed in SIM mode (top; M-1, negative mode; m/z 115, 133 for the detection of malic acid) with overlay (green: reaction control without enzyme, blue: biotransformation), and at 245 nm (bottom).


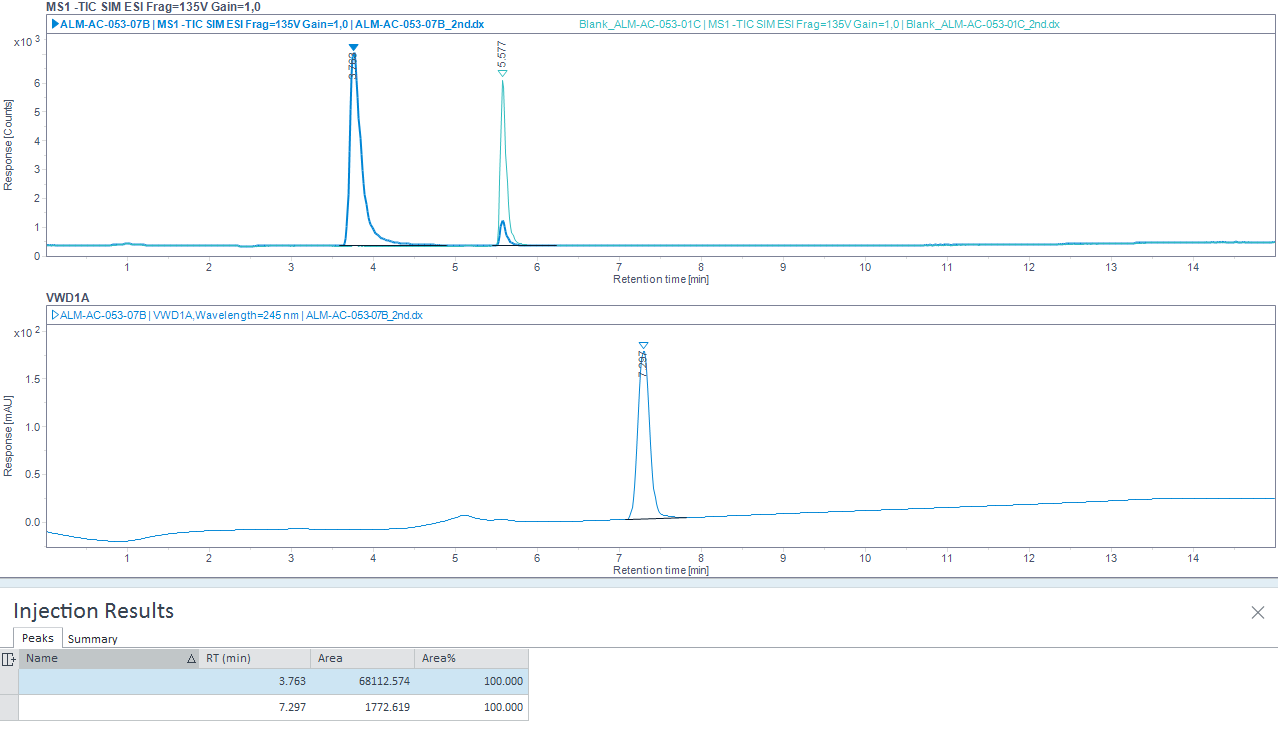


Figure S5.17: Hydronitration reaction catalysed by EcFumC. Retention times: 3.8 malic acid, 5.6 min fumaric acid, 7.3 min cis,cis-muconic acid (IS). Analysis performed in SIM mode (top; M-1, negative mode; m/z 115, 133 for the detection of malic acid) with overlay (green: reaction control without enzyme, blue: biotransformation), and at 245 nm (bottom).


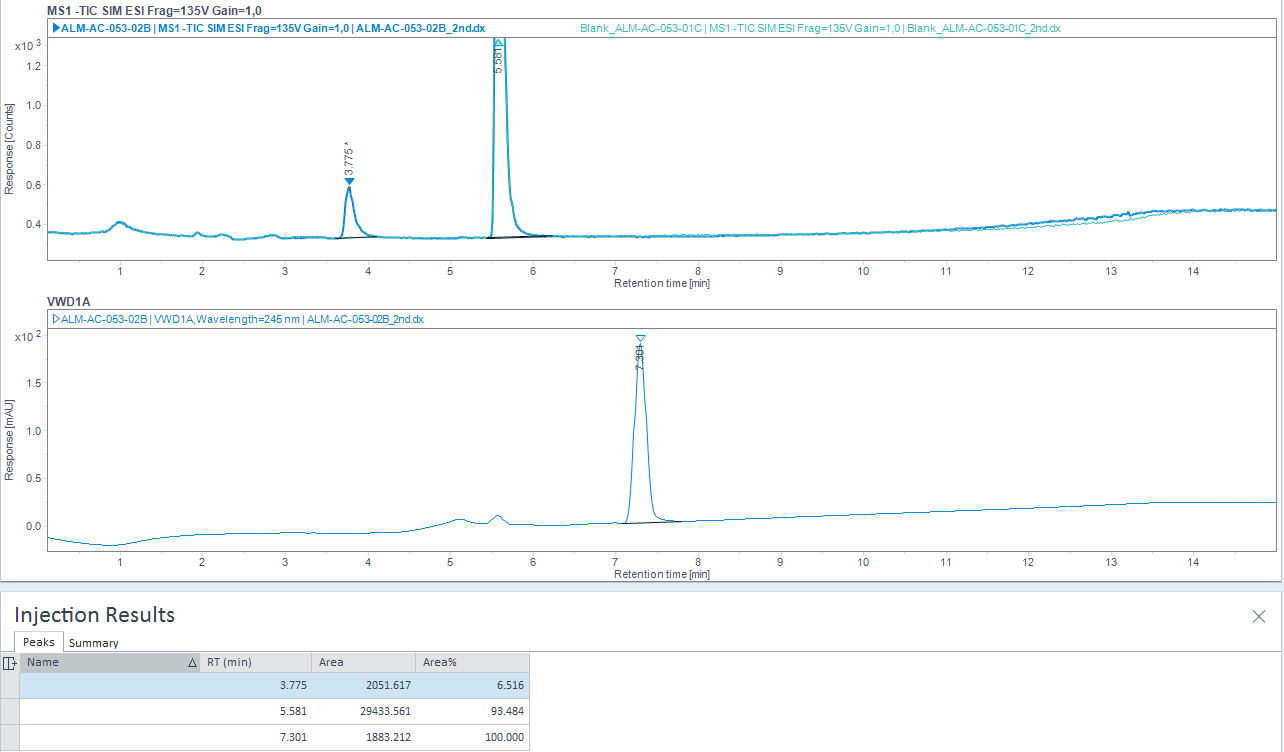


Figure S5.18: Hydronitration reaction catalysed by BspAspB. Retention times: 3.8 malic acid, 5.6 min fumaric acid, 7.3 min cis,cis-muconic acid (IS). Analysis performed in SIM mode (top; M-1, negative mode; m/z 115, 133 for the detection of malic acid) with overlay (green: reaction control without enzyme, blue: biotransformation), and at 245 nm (bottom).


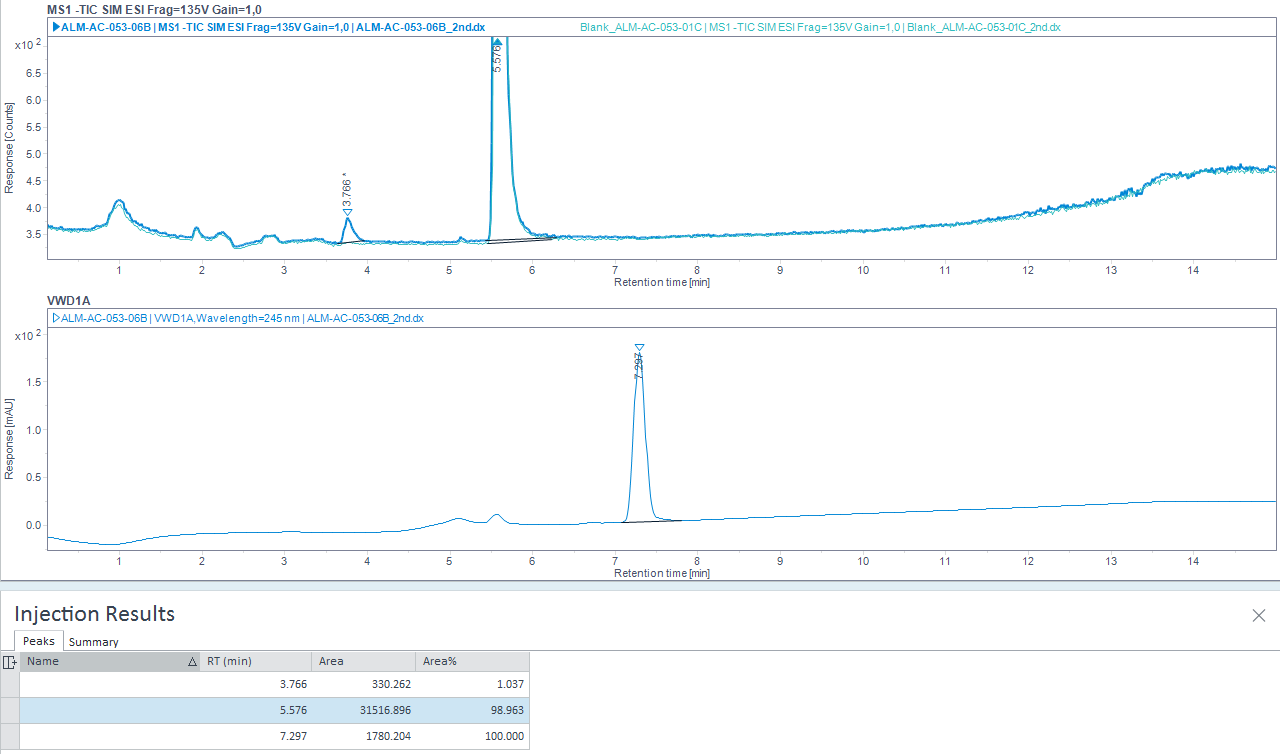


Figure S5.19: Hydronitration reaction catalysed by MtASL. Retention times: 3.8 malic acid, 5.6 min fumaric acid, 7.3 min cis,cis-muconic acid (IS). Analysis performed in SIM mode (top; M-1, negative mode; m/z 115, 133 for the detection of malic acid) with overlay (green: reaction control without enzyme, blue: biotransformation), and at 245 nm (bottom).


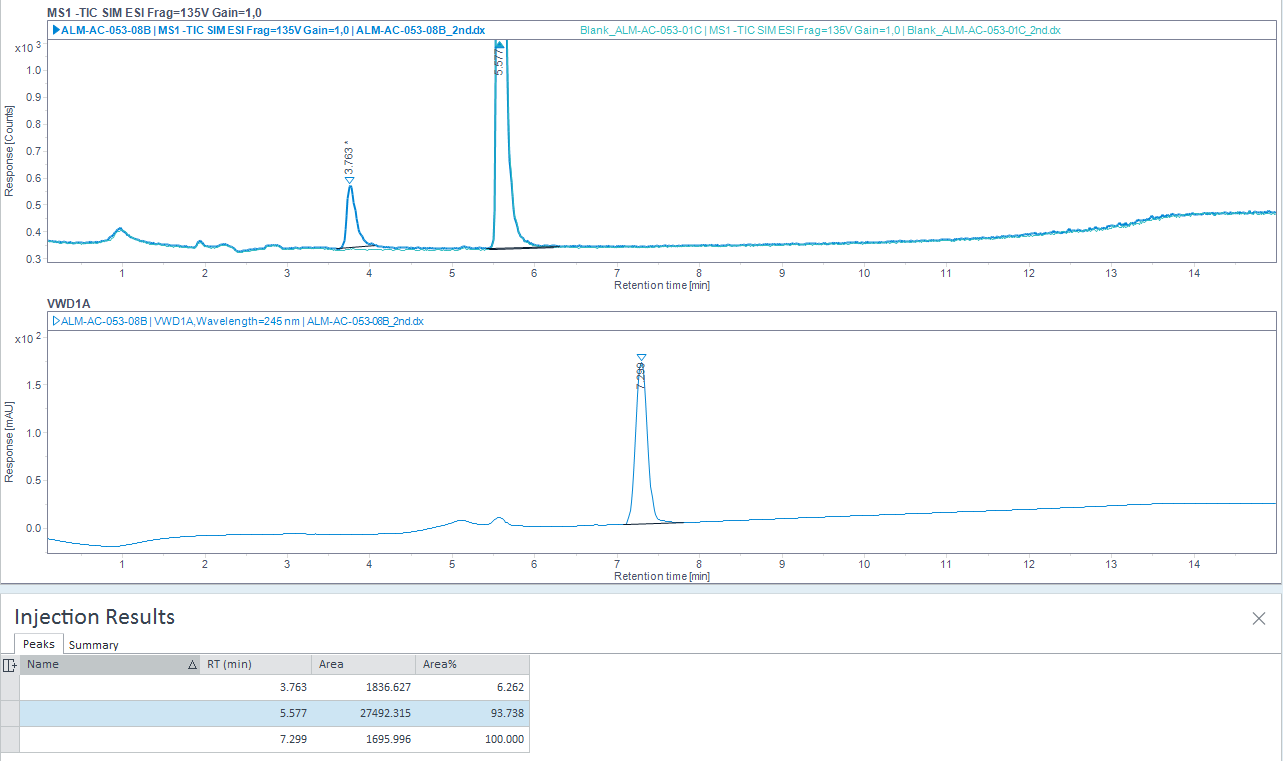


Figure S5.20: Hydronitration reaction catalysed by HsnADSL. Retention times: 3.8 malic acid, 5.6 min fumaric acid, 7.3 min cis,cis-muconic acid (IS). Analysis performed in SIM mode (top; M-1, negative mode; m/z 115, 133 for the detection of malic acid) with overlay (green: reaction control without enzyme, blue: biotransformation), and at 245 nm (bottom).

## Experimental procedure - hydroamination reaction

The reaction mixtures (final volume 500 µL) were prepared by solubilizing in NH_4_OH_(aq.)_ (prepared from an aqueous 25% solution, to reach 3.0 M as final concentration), in order: sodium fumarate_(s)_ (to reach final concentration of 50 mM), HEPES_(s)_ (to reach final concentration of 400 mM). The pH was adjusted to 9.0 with HCl_(aq)_ (37 w%) and the enzyme solution was added (to reach final concentration of 10 μM). The reactions were incubated in an orbital shaker for 18 h at 30 °C (120 rpm). For analysis, aspartic acid was derivatized (see Figure S5.21) by adding to 25 µL of the reaction mixture, in order: NaHCO_3(sat)_ (10 µL) and 1-fluoro-2,4-dinitrobenzene (DNFB, 40 µL, 0.28 M in acetone). The samples were incubated in a thermoshaker at 40 °C for 1.5 h (500 rpm). After addition of HCl_(aq)_ (10 µL, 1 M), they were diluted with CH_3_CN:H_2_O (20:80) (165 µL; spiked with IS 1-(4-hydroxy-3-methylphenyl)ethan-1-one)), centrifuged (18,000 rpm, 2 min) and filtered with cotton. The solutions were analyzed with HPLC-UV (Method UV-02) for the quantification of **1** and **6**. The side product **4** was quantified with HPLC-MS (Method MS-03) following dilution of the samples 1:2 with CH_3_CN:H_2_O (20:80) (spiked with IS, 5 mM). The reactions were performed in triplicates (average value reported with standard deviation).

Derivatization reaction for the quantification of l-aspartic acid via HPLC-UV

Figure S5.21: Derivatization reaction for the quantification of l-aspartic acid in hydroamination reactions

## Preparation of the calibration curves – hydroamination reaction

### Sodium fumarate and l-aspartic acid

The calibration curves (Figure S5.22-Figure S5.24) were obtained according to the following procedures. A solution containing sodium fumarate_(s)_ or L-aspartic acid_(s)_ (50 mM) was prepared in a mixture of *Sc*CreD storage buffer and reaction buffer containing NH_3(aq)_ (to reach final concentration of 3.0 M), and the pH was adjusted to 9.0 with HCl_(aq)_ (37 w%). The ratio of *Sc*CreD storage buffer and reaction buffer was the same as in the biotransformations. This solution was diluted with the storage/reaction buffer mixture to reach the concentrations reported in Figure S5.22Figure S5.23. The samples were incubated for 3 h (30 °C, 120 rpm), 25 μL of the reaction were worked up according to the standard procedure (see Section 5.5) and **6** and **1** were quantified with HPLC-UV (Method UV-02).

### Malic acid

The calibration curve (Figure S5.24) for the side product **4** was prepared as described in Section 5.6.1, using *rac*-malic acid as the analyte. The resulting solution was diluted 1:2 with CH_3_CN:H_2_O (20:80) (spiked with IS) and analysed by HPLC–MS (Method MS-03).

### Calibration curves

| \| [6]  (mM) \| Normalized area^1^ \| \| --- \| --- \| \| 0.1 \| 0.009 ± 0.009 \| \| 0.25 \| 0.007 \| \| 0.5 \| 0.015 \| \| 0.75 \| 0.022 ± 0.001 \| \| 1 \| 0.029 ± 0.001 \| \| 2.5 \| 0.077 ± 0.001 \| \| 5 \| 0.147 ± 0.003 \| \| 10 \| 0.301 ± 0.002 \| \| 25 \| 0.738 ± 0.009 \| \| 50 \| 1.417 ± 0.009 \| |  |
| --- | --- | --- | --- | --- | --- | --- | --- | --- | --- | --- | --- | --- | --- | --- | --- | --- | --- | --- | --- | --- | --- | --- | --- |

Figure S5.22: Calibration curve for L-aspartic acid. Left: table with (normalized area) vs [aspartic acid] (Note: if not indicated, RMSD < 0.001); Right: plot of the calibration points in the linear range. **^1^**: the calibration points were plotted as arithmetic average of triplicates calculated as [counts]_l-aspartic acid_/[counts]_IS_. The error bars are the standard deviation between the triplicates. If not displayed error bars are smaller than the data points.

| \| [1a]  (mM) \| Normalized area^1^ \| \| --- \| --- \| \| 0.25 \| 0.016 \| \| 0.5 \| 0.029 ± 0.001 \| \| 0.75 \| 0.047 \| \| 1 \| 0.063 ± 0.001 \| \| 2.5 \| 0.155 \| \| 5 \| 0.310 ± 0.007 \| \| 10 \| 0.616 ± 0.003 \| \| 25 \| 1.481 ± 0.003 \| \| 50 \| 2.776 ± 0.012 \| |  |
| --- | --- | --- | --- | --- | --- | --- | --- | --- | --- | --- | --- | --- | --- | --- | --- | --- | --- | --- | --- | --- | --- |

Figure S5.23: Calibration curve for fumarate. Left: table with (normalized area) vs [sodium fumarate] (Note: if not indicated, RMSD < 0.001); Right: plot of the calibration points in linear range. **^1^**: the calibration points were plotted as arithmetic average of triplicates calculated as [counts]_fumarate_/[counts]_IS_. The error bars are the standard deviation between the triplicates. If not displayed error bars are smaller than the data points.

| \| [4]  (mM) \| Normalized area^1^ \| \| --- \| --- \| \| 0.1 \| 0.25 ± 0.04 \| \| 1 \| 2.91 ± 0.58 \| \| 2.5 \| 6.16 ± 0.59 \| \| 10 \| 24.40 ± 0.77 \| \| 25 \| 57.51 ± 3.42 \| |  |
| --- | --- | --- | --- | --- | --- | --- | --- | --- | --- | --- | --- | --- | --- |

Figure S5.24: Calibration curve for malic acid. Left: table with (normalized area) vs [malic acid]; Right: plot of the calibration points in linear range. **^1^**: the calibration points were plotted as arithmetic average of triplicates calculated as [counts]_malic acid_/[counts]_IS_. The error bars are the standard deviation between the triplicates. If not displayed error bars are smaller than the data points.

## Chromatograms

### HPLC-UV traces (analyses performed with **Method UV-02**)

Note: the following chromatograms present a peak at 15.9 min, which represents the compound obtained during the workup from the derivatization of the excess of NH_3_ with DNFB.


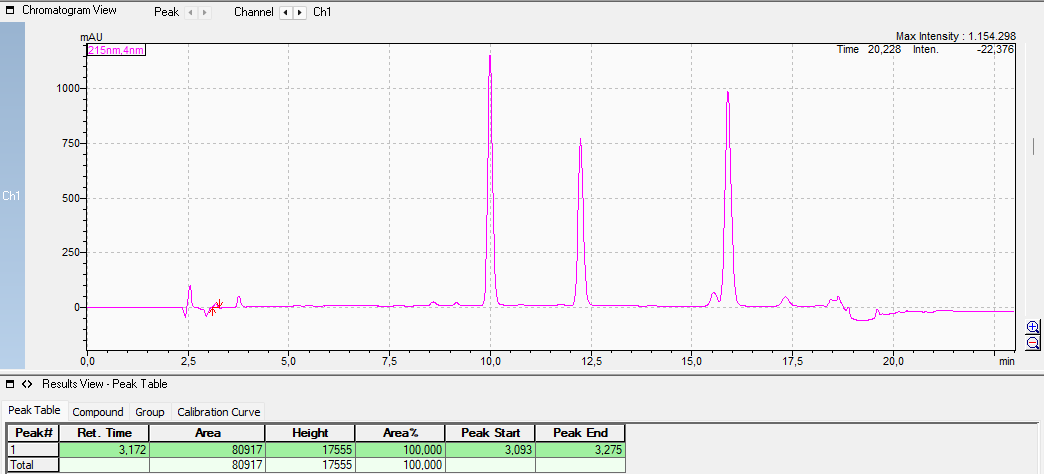


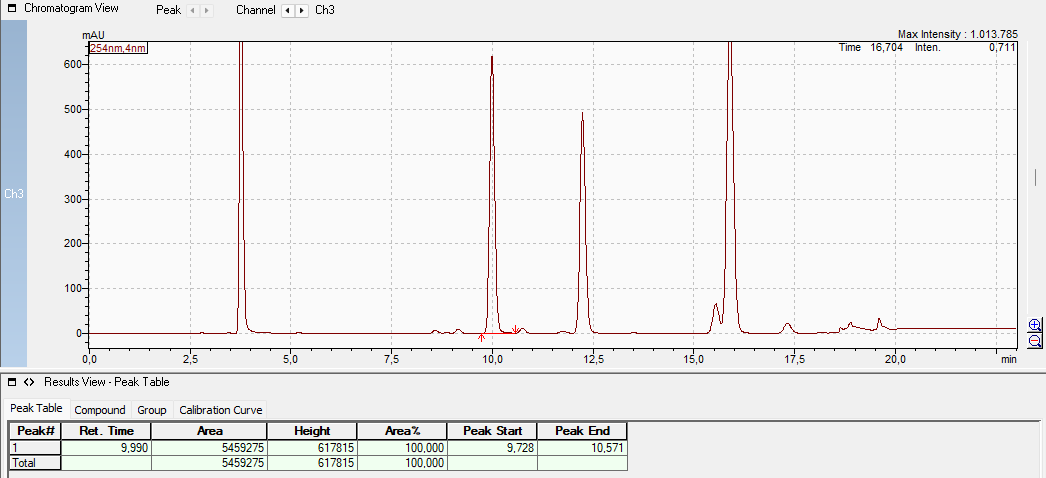


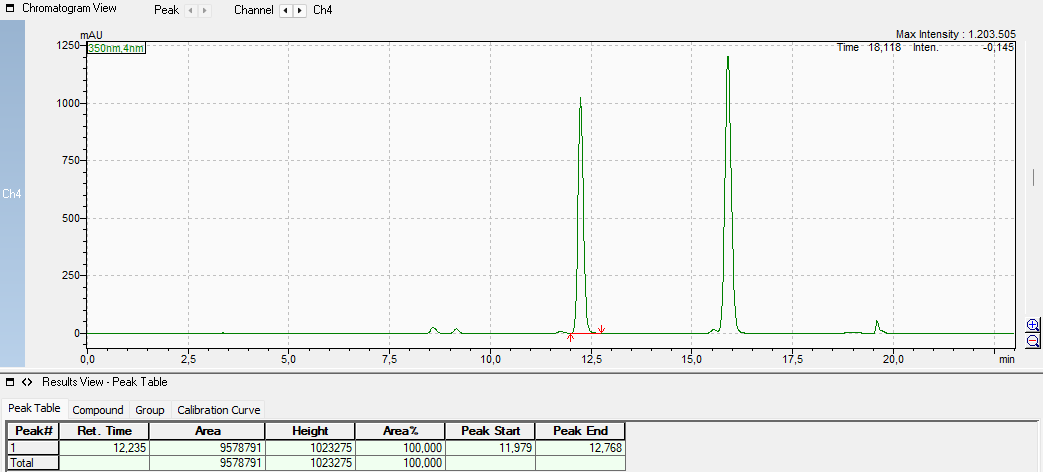


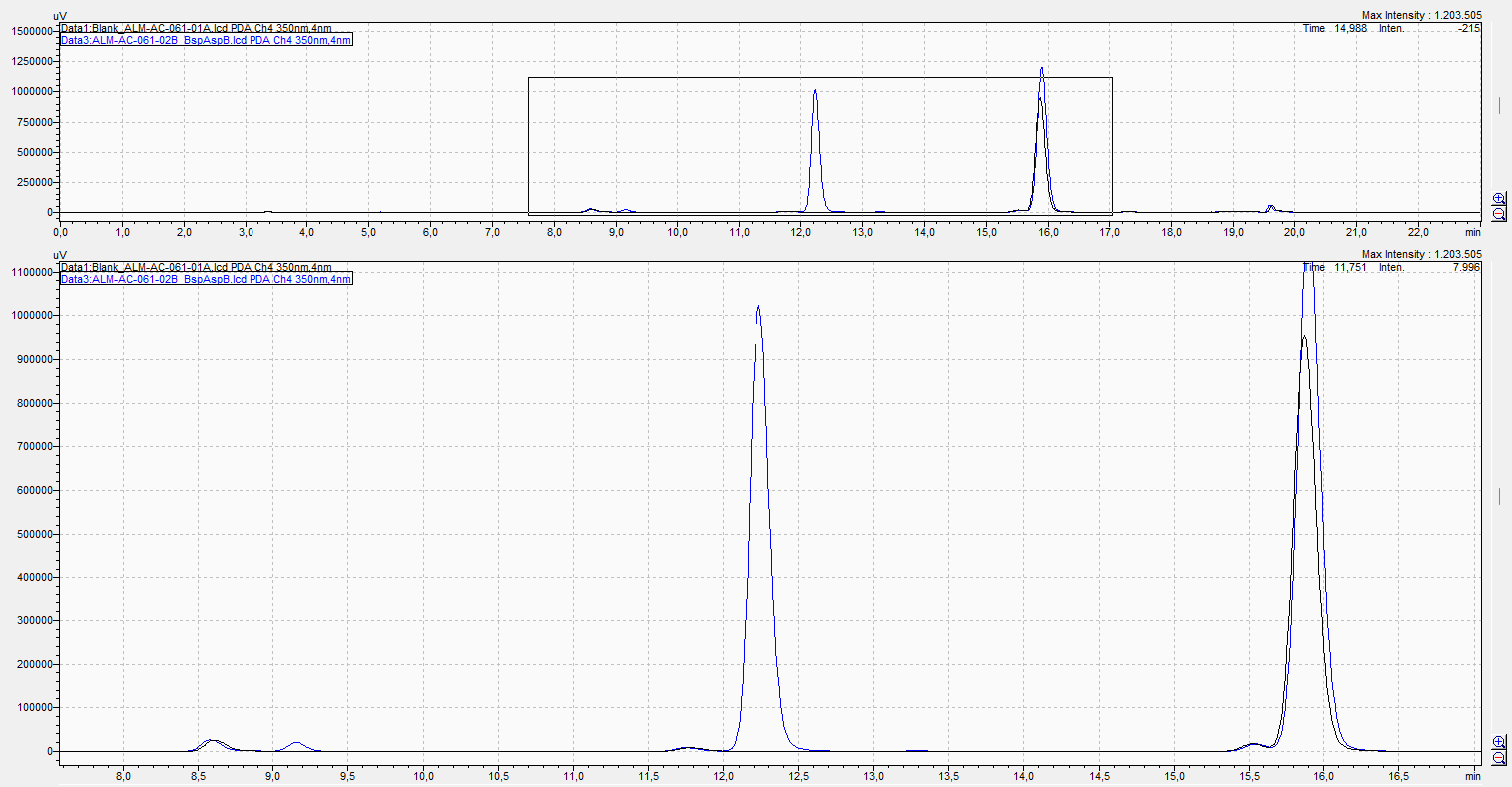


Figure S5.25: Hydroamination reaction catalyzed by BspAspB. Retention times: 3.2 min fumaric acid, 10.0 min 1-(4-hydroxy-3-methylphenyl)ethan-1-one (IS), 12.2 min aspartic acid (derivatized). Analysis performed at 215 nm (first chromatogram), 254 nm (second chromatogram), 350 nm (third chromatogram, and bottom: overlayed chromatograms, black: reaction control without enzyme, blue: biotransformation)


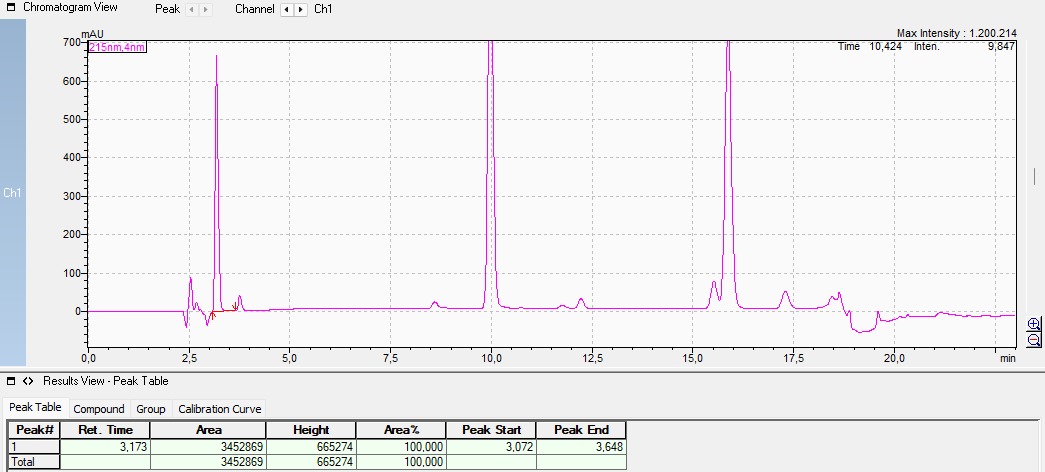


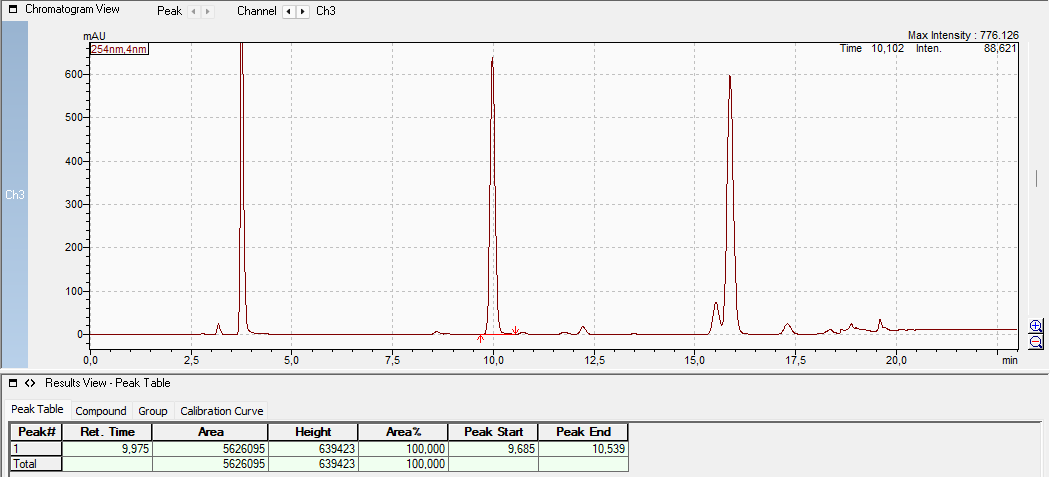


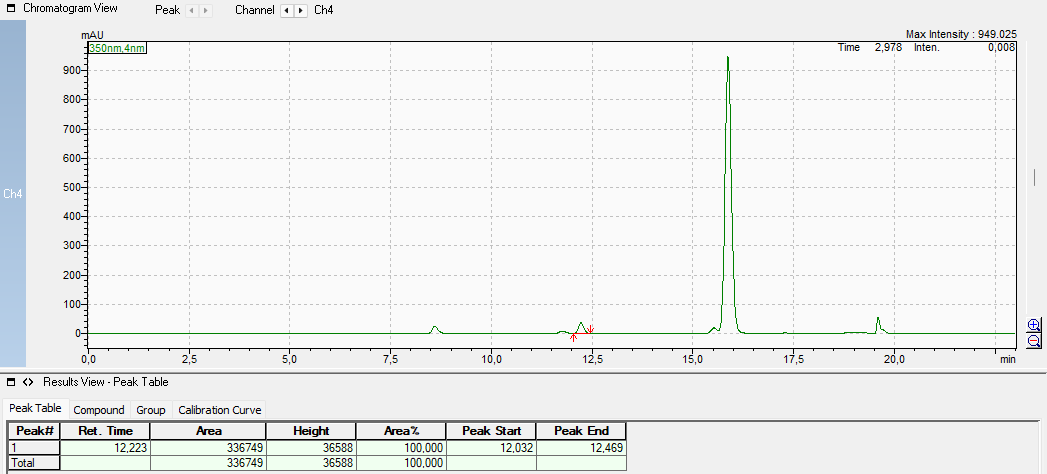


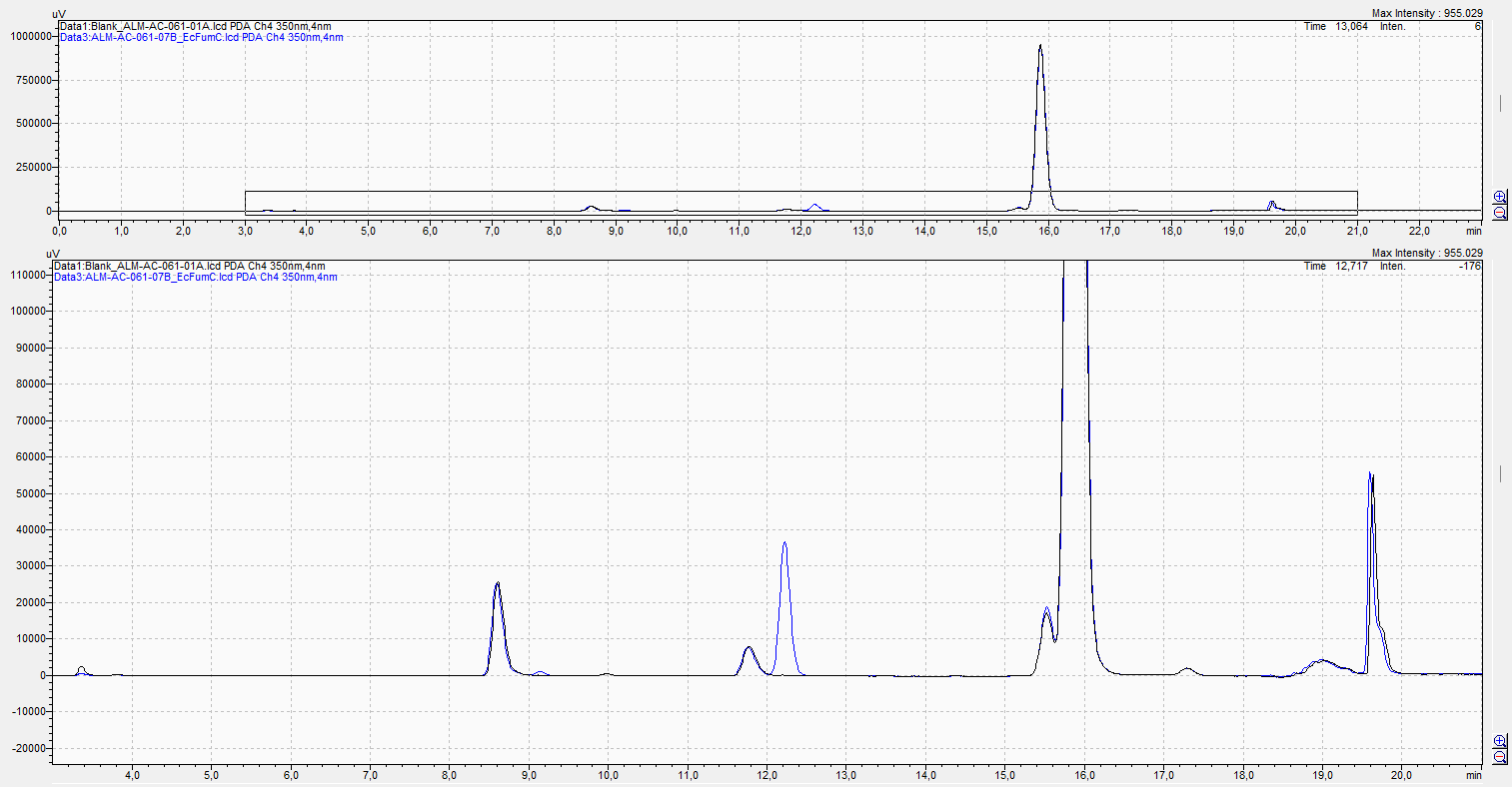


Figure S5.26: Hydroamination reaction catalyzed by EcFumC. Retention times: 3.2 min fumaric acid, 10.0 min 1-(4-hydroxy-3-methylphenyl)ethan-1-one (IS), 12.2 min aspartic acid (derivatized). Analysis performed at 215 nm (first chromatogram), 254 nm (second chromatogram), 350 nm (third chromatogram, and bottom: overlayed chromatograms, black: reaction control without enzyme, blue: biotransformation)


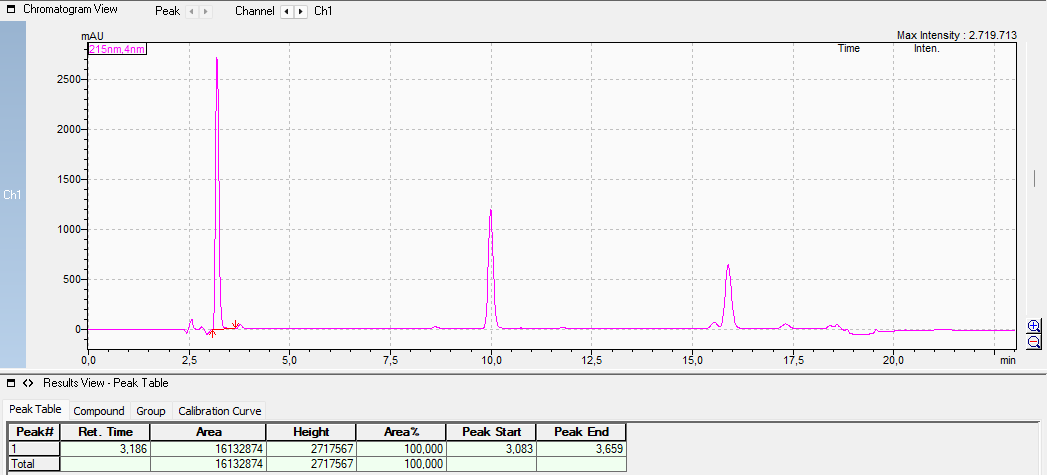


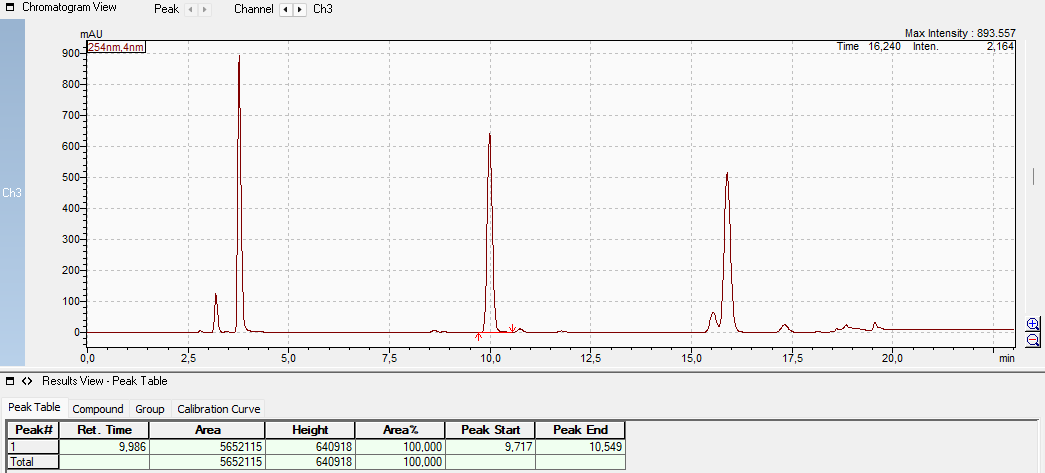


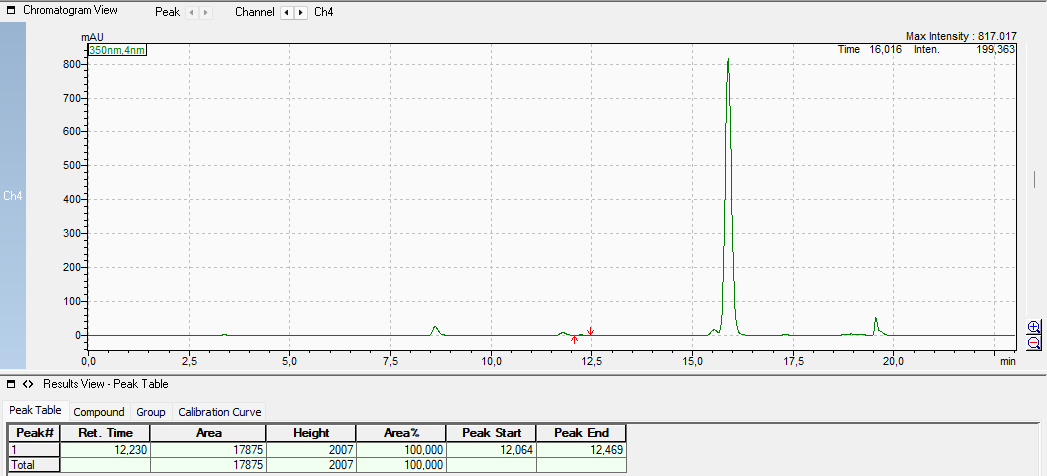


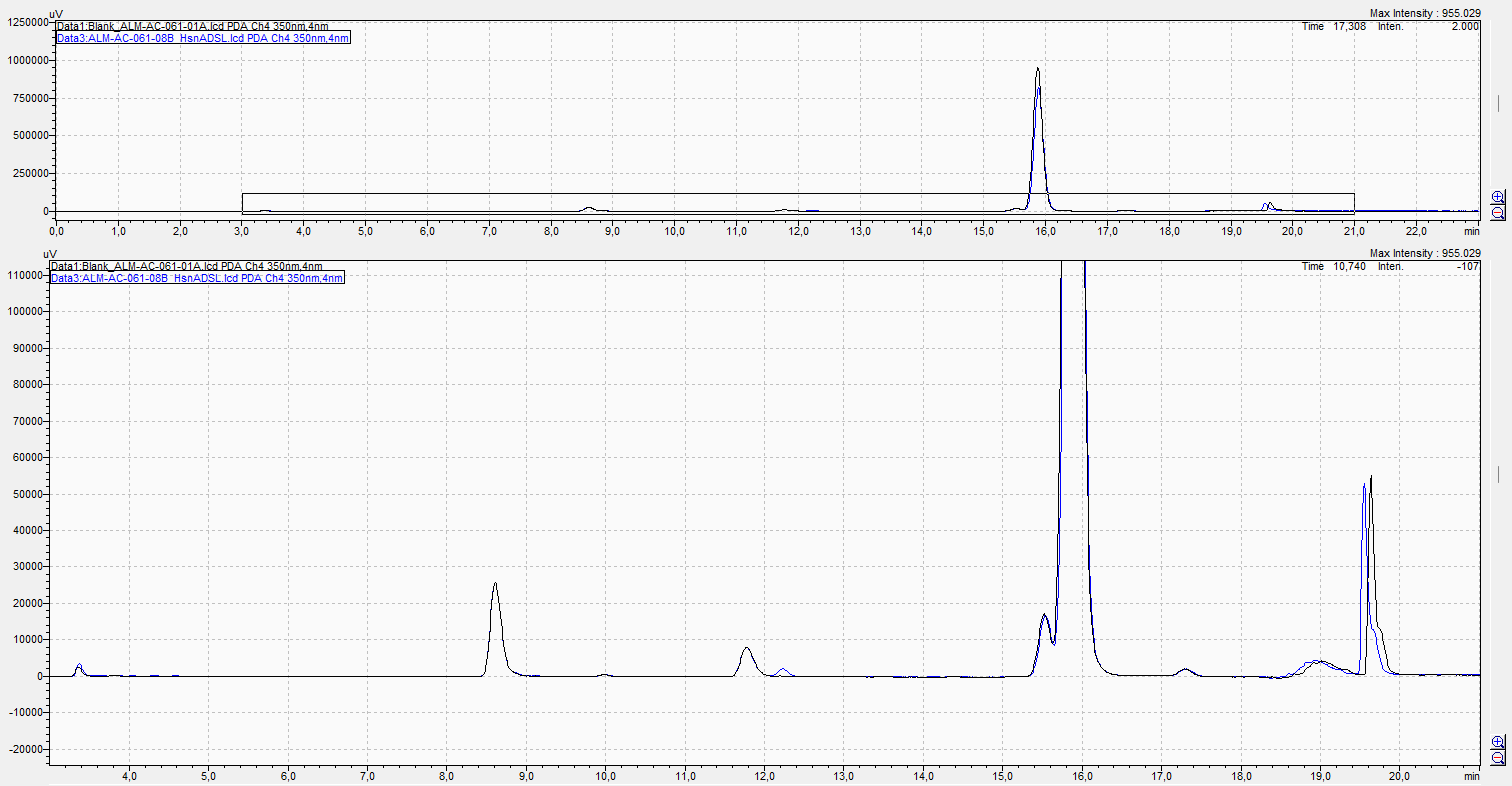


Figure S5.27: Hydroamination reaction catalyzed by HsnADSL. Retention times: 3.2 min fumaric acid, 10.0 min 1-(4-hydroxy-3-methylphenyl)ethan-1-one (IS), 12.2 min aspartic acid (derivatized). Analysis performed at 215 nm (first chromatogram), 254 nm (second chromatogram), 350 nm (third chromatogram, and bottom: overlayed chromatograms, black: reaction control without enzyme, blue: biotransformation)


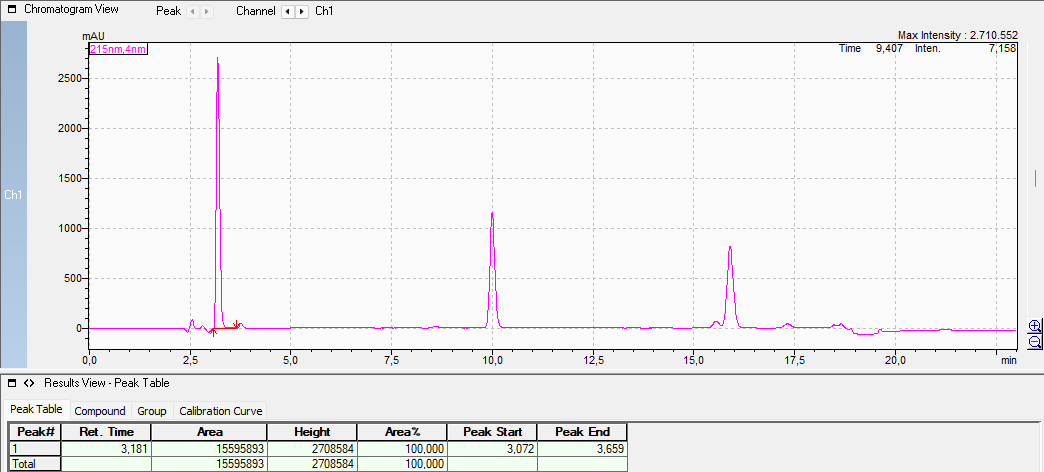


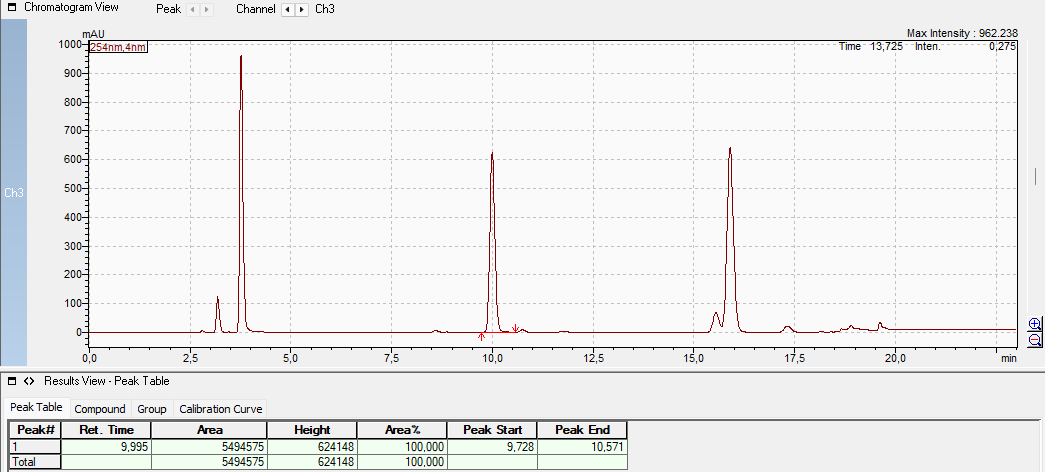


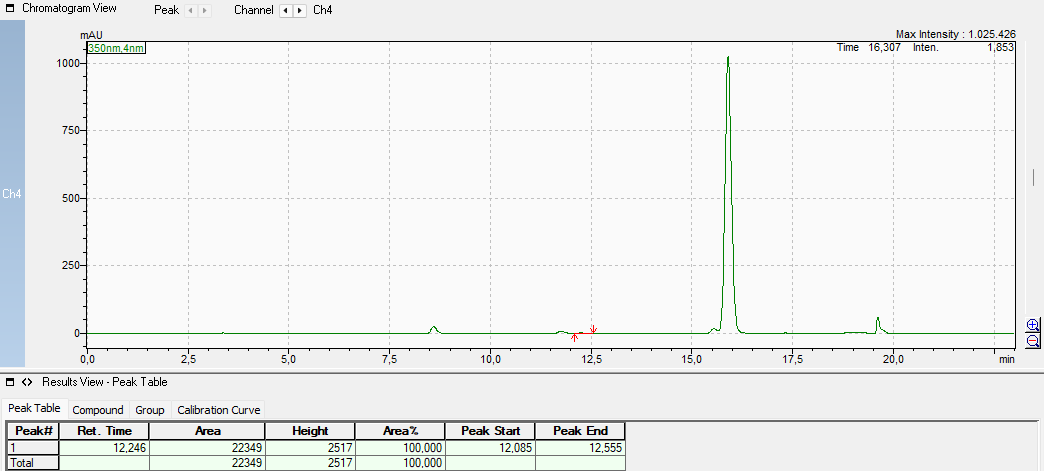


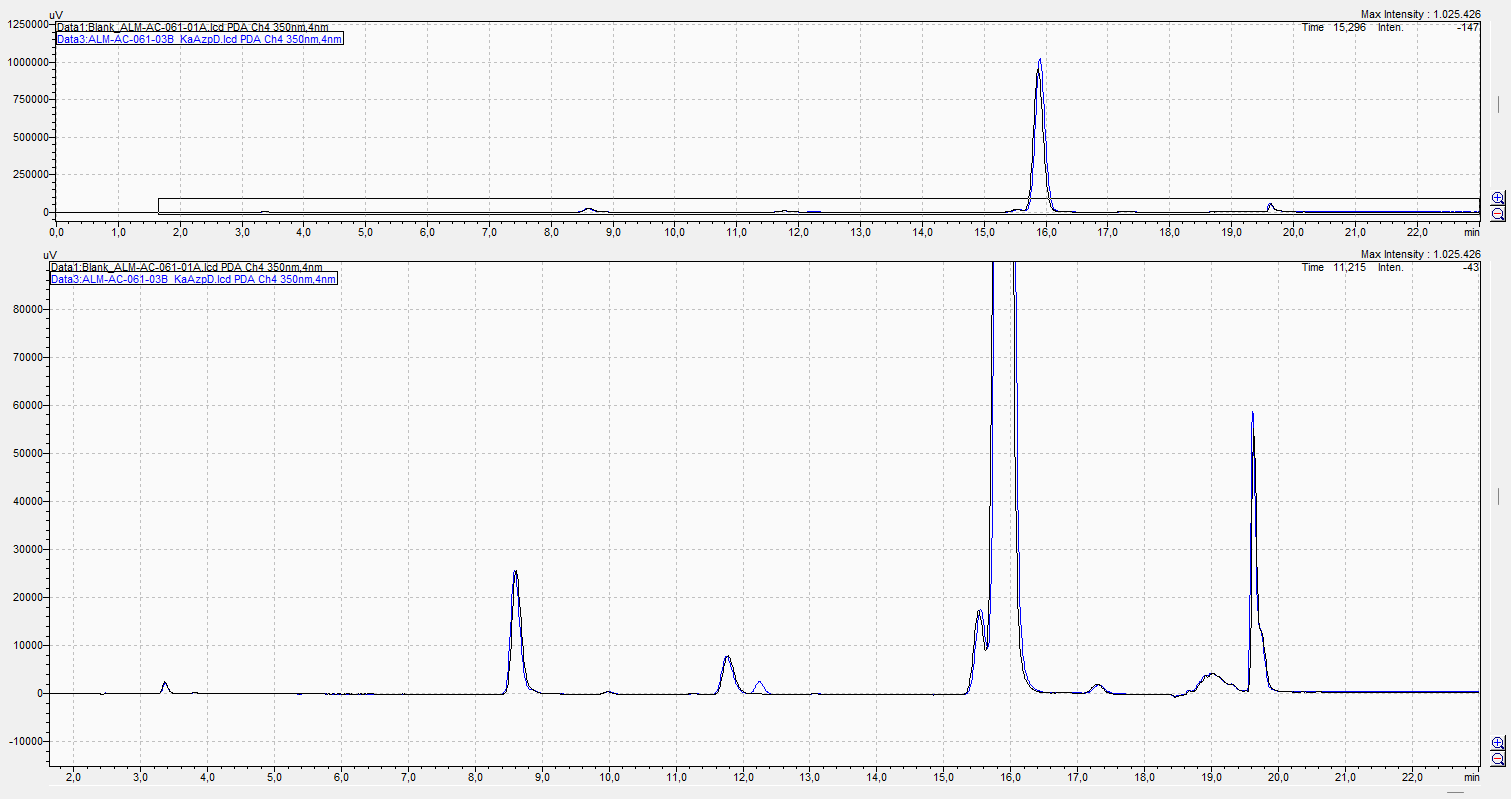


Figure S5.28: Hydroamination reaction catalyzed by KaAzpD. Retention times: 3.2 min fumaric acid, 10.0 min 1-(4-hydroxy-3-methylphenyl)ethan-1-one (IS), 12.2 min aspartic acid (derivatized). Analysis performed at 215 nm (first chromatogram), 254 nm (second chromatogram), 350 nm (third chromatogram, and bottom: overlayed chromatograms, black: reaction control without enzyme, blue: biotransformation)


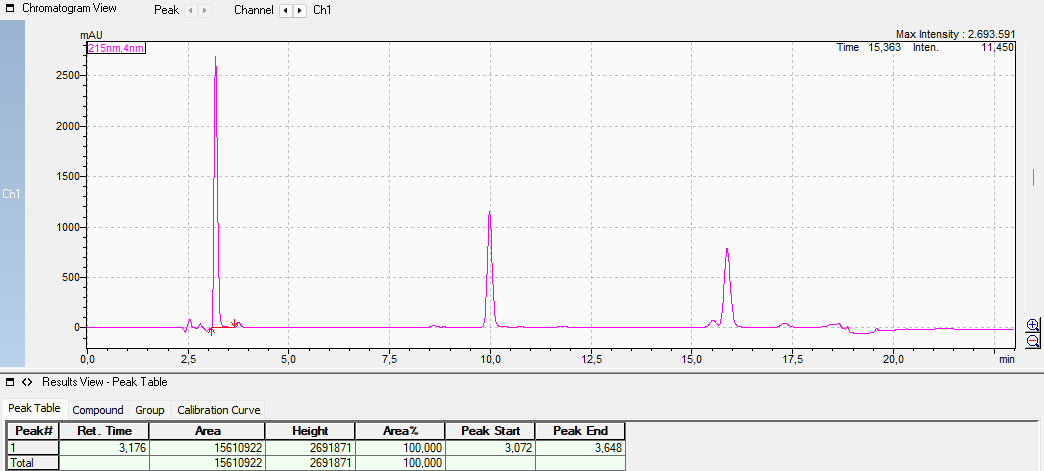


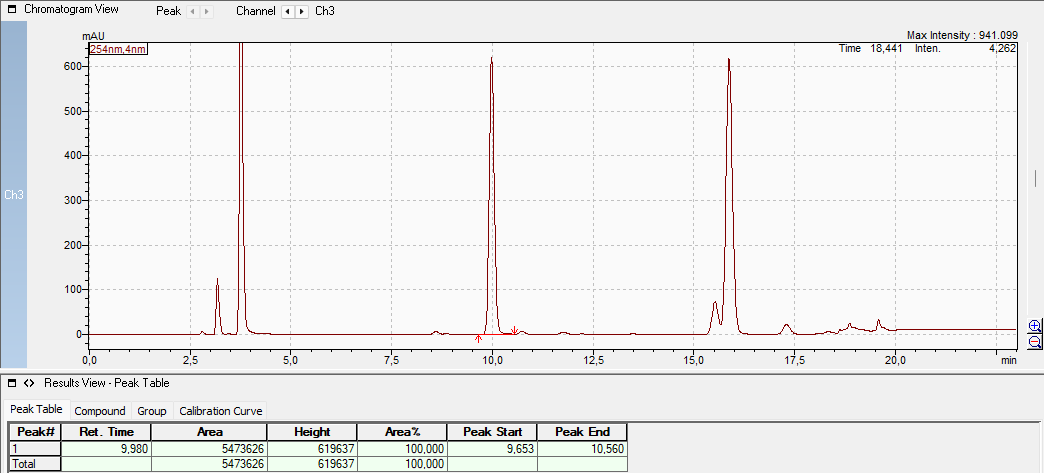


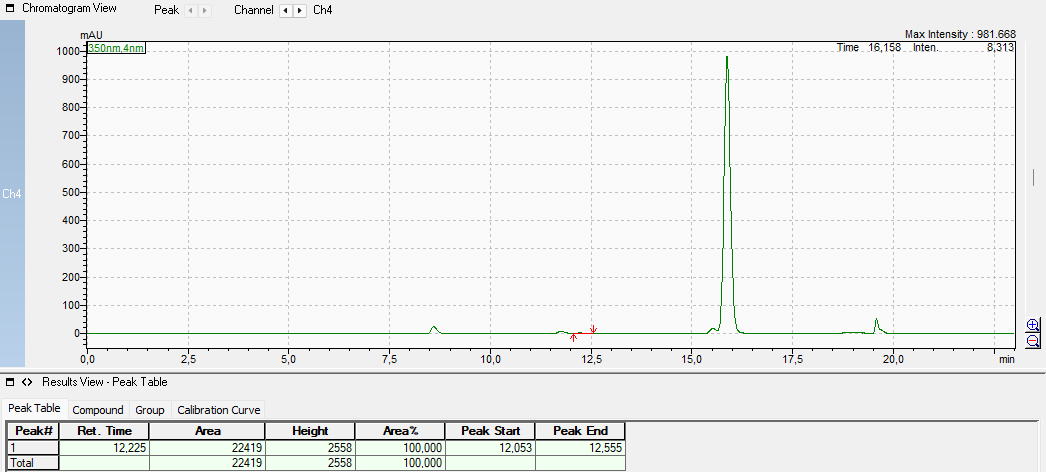


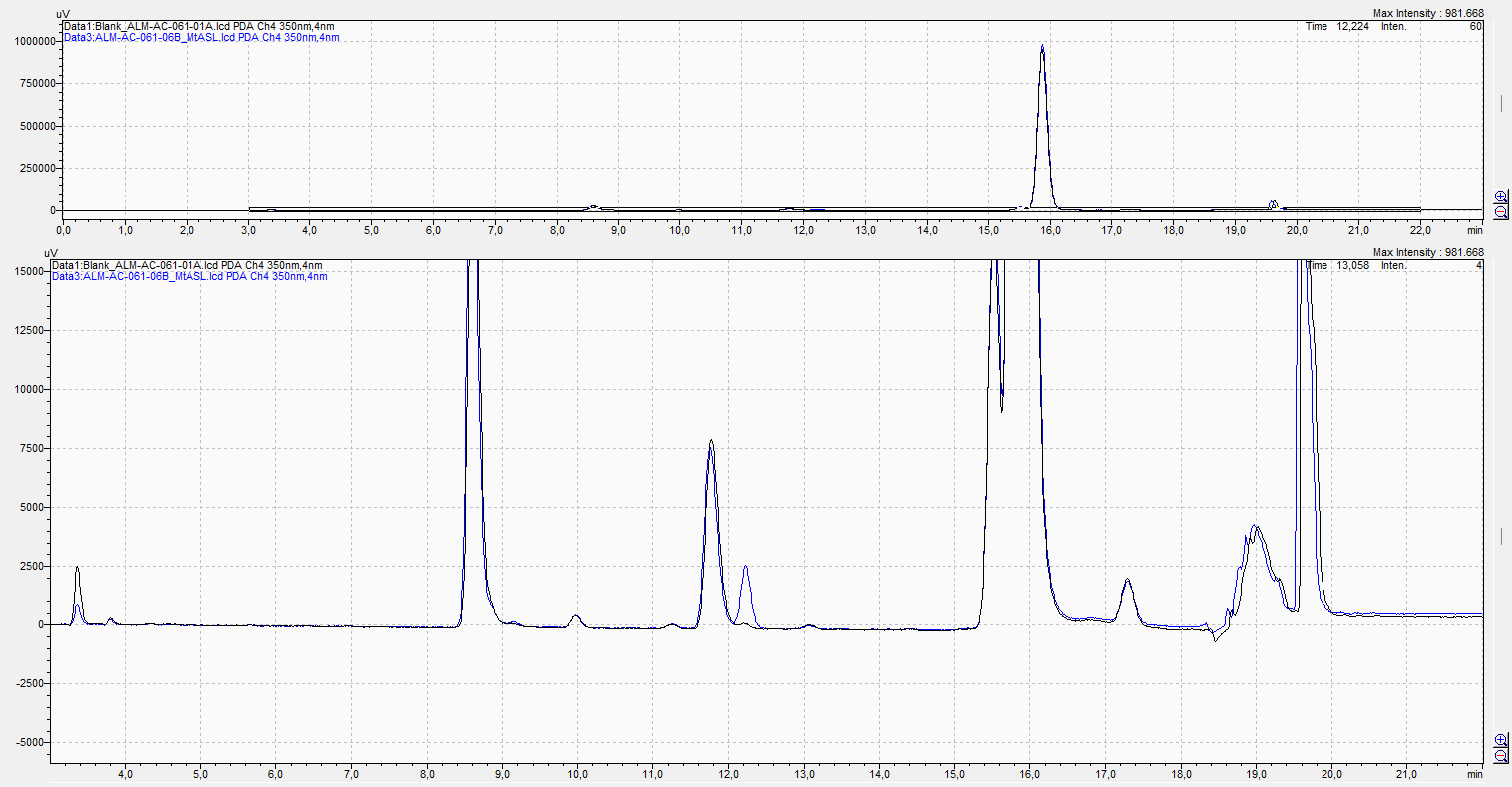


Figure S5.29: Hydroamination reaction catalyzed by MtASL. Retention times: 3.2 min fumaric acid, 10.0 min 1-(4-hydroxy-3-methylphenyl)ethan-1-one (IS), 12.2 min aspartic acid (derivatized). Analysis performed at 215 nm (first chromatogram), 254 nm (second chromatogram), 350 nm (third chromatogram, and bottom: overlayed chromatograms, black: reaction control without enzyme, blue: biotransformation)


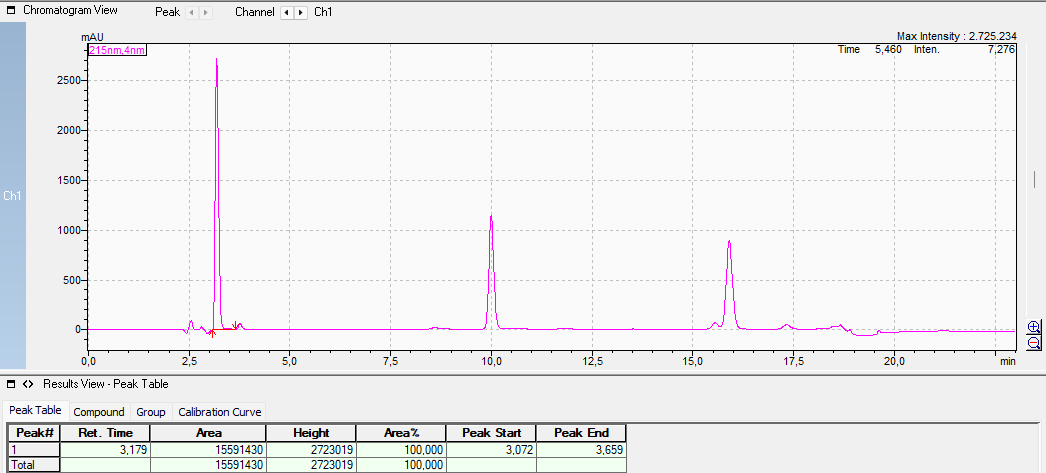


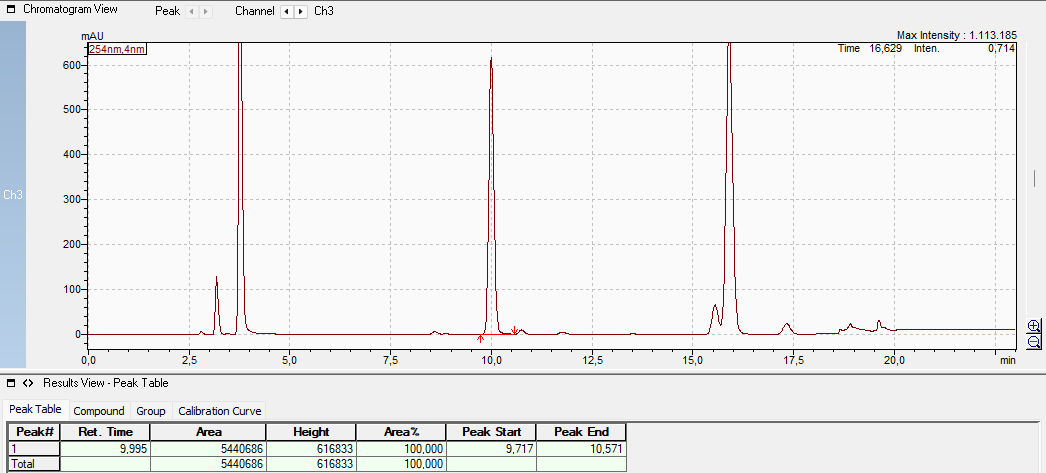


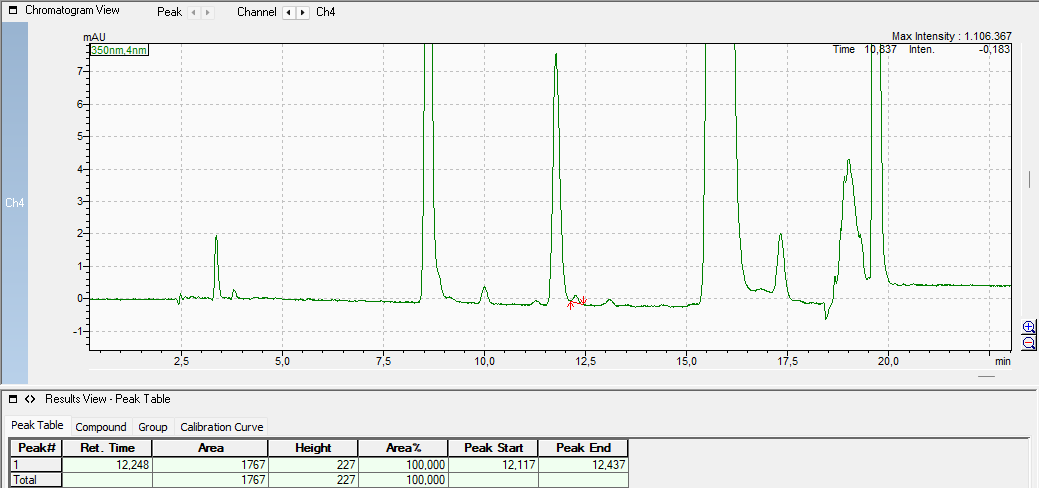


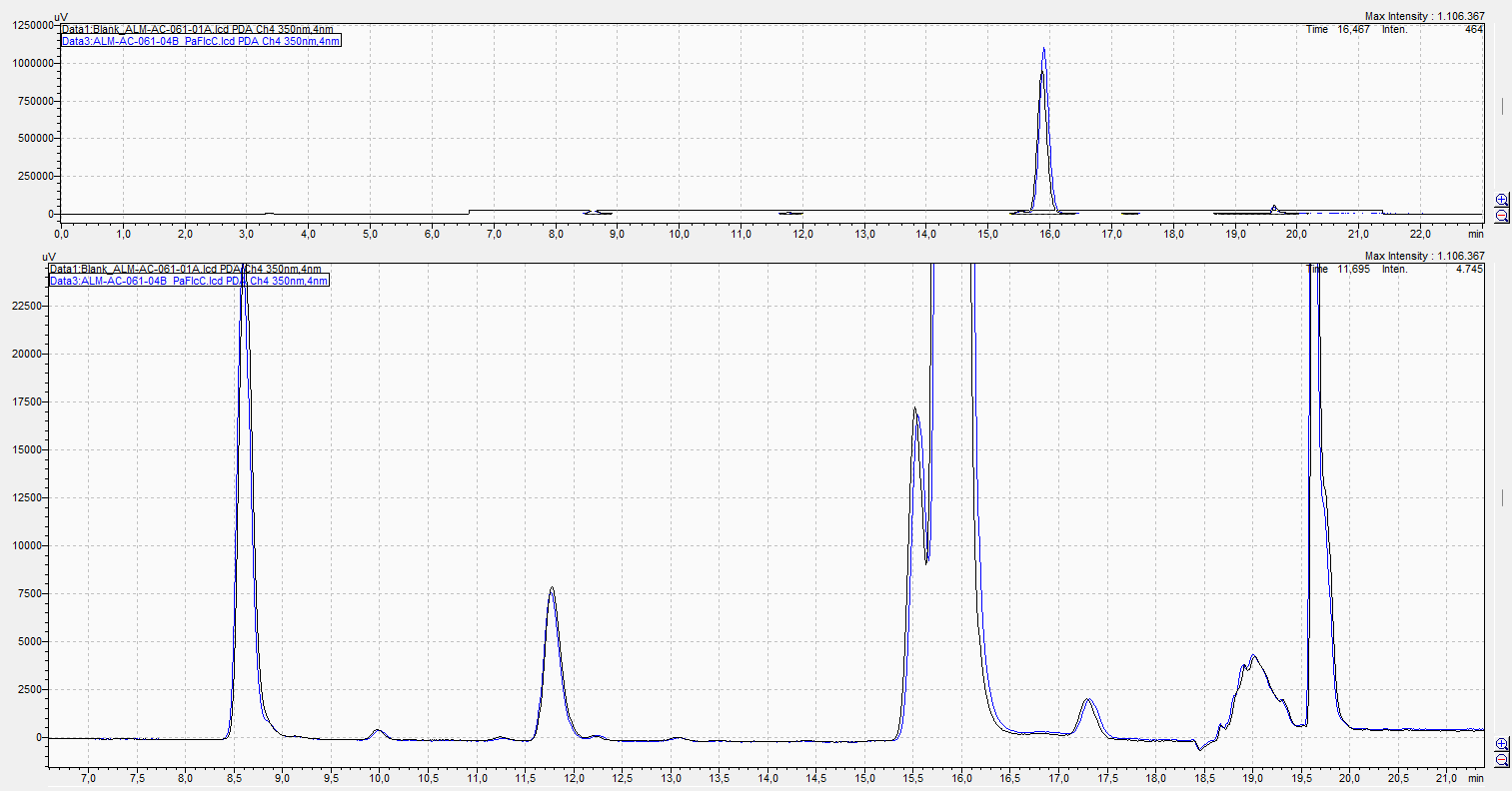


Figure S5.30: Hydroamination reaction catalyzed by PaFlcC. Retention times: 3.2 min fumaric acid, 10.0 min 1-(4-hydroxy-3-methylphenyl)ethan-1-one (IS), 12.2 min aspartic acid (derivatized). Analysis performed at 215 nm (first chromatogram), 254 nm (second chromatogram), 350 nm (third chromatogram, and bottom: overlayed chromatograms, black: reaction control without enzyme, blue: biotransformation)


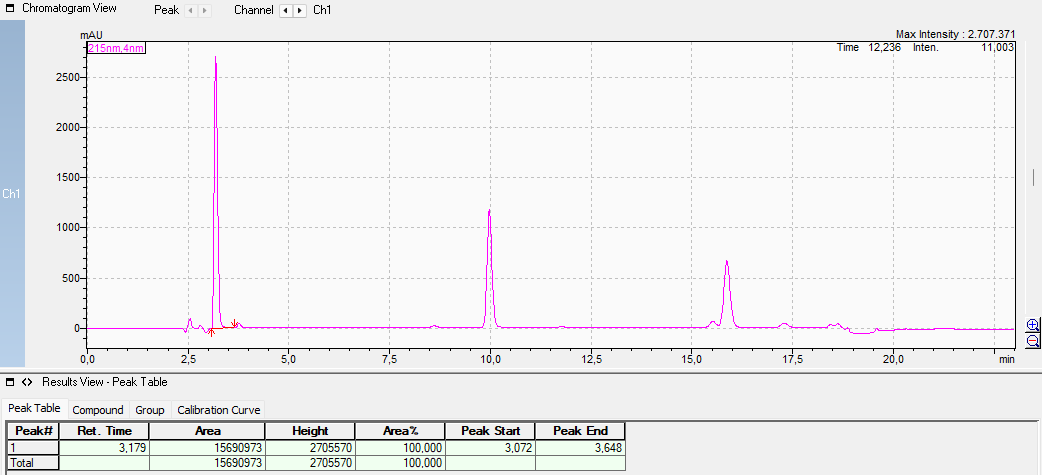


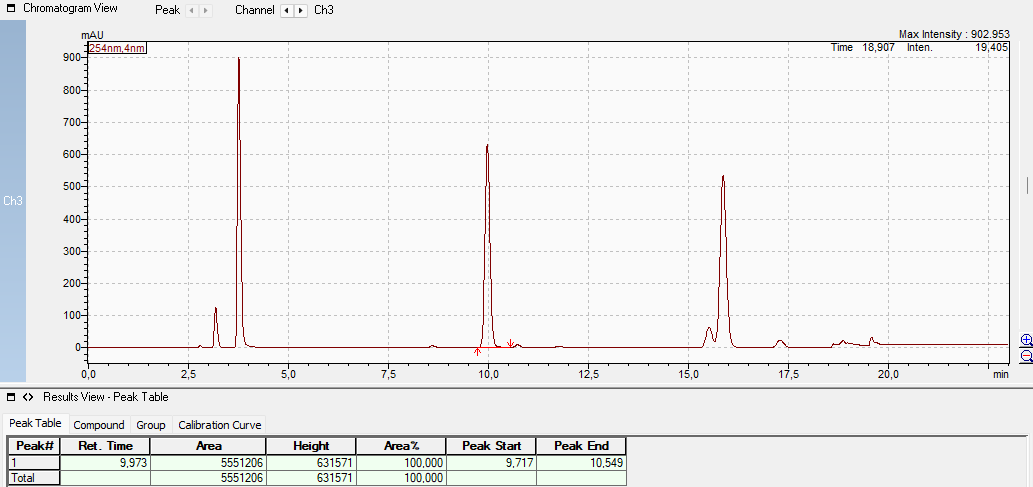


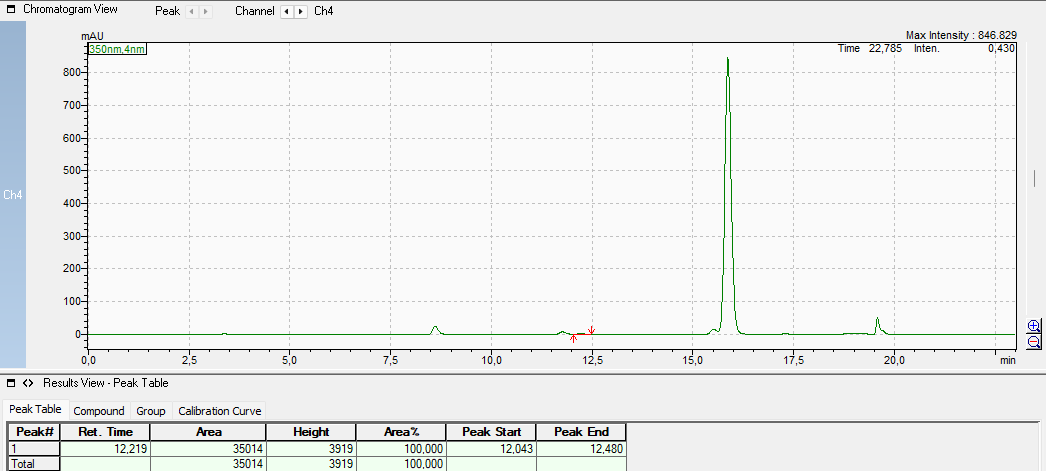


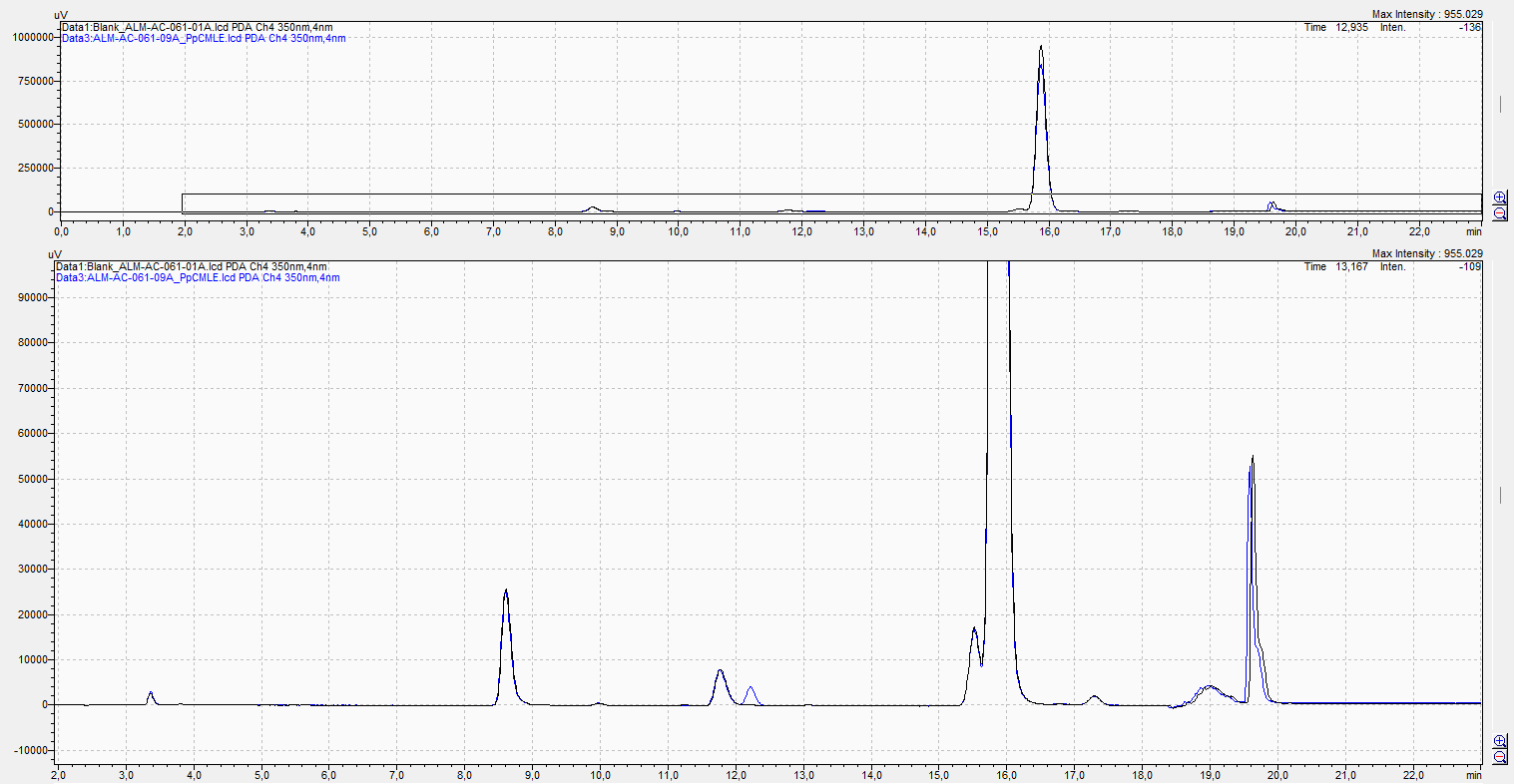


Figure S5.31: Hydroamination reaction catalyzed by PpCMLE. Retention times: 3.2 min fumaric acid, 10.0 min 1-(4-hydroxy-3-methylphenyl)ethan-1-one (IS), 12.2 min aspartic acid (derivatized). Analysis performed at 215 nm (first chromatogram), 254 nm (second chromatogram), 350 nm (third chromatogram, and bottom: overlayed chromatograms, black: reaction control without enzyme, blue: biotransformation)


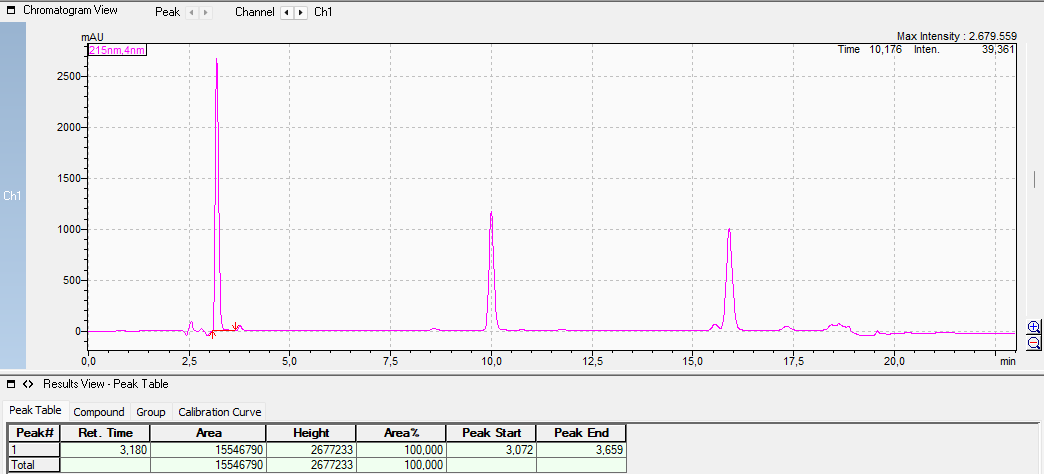


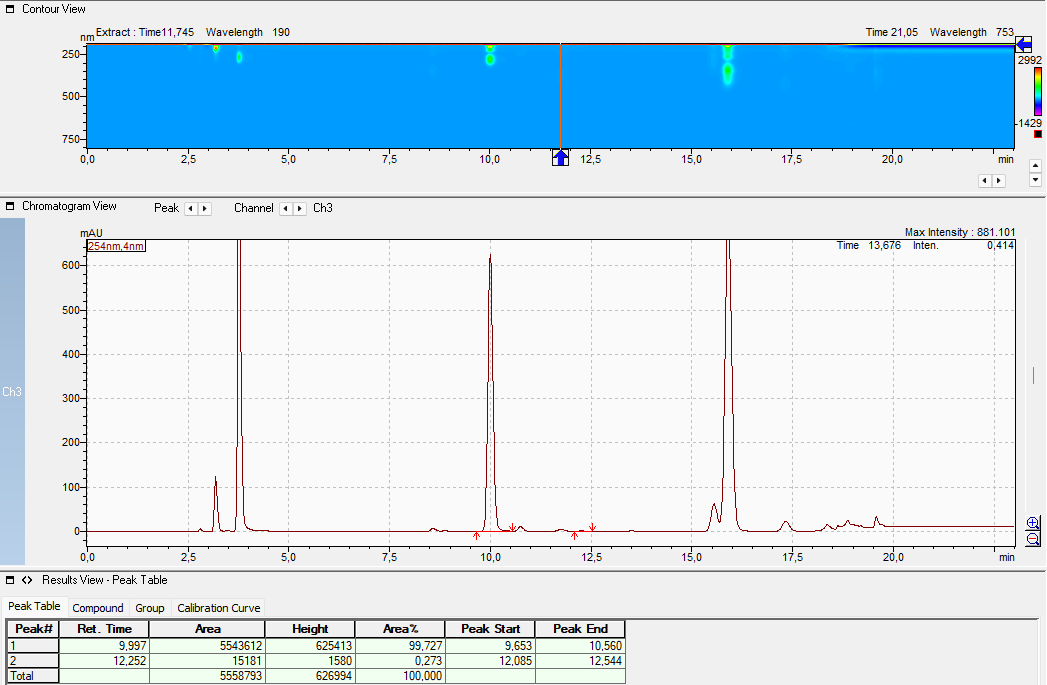


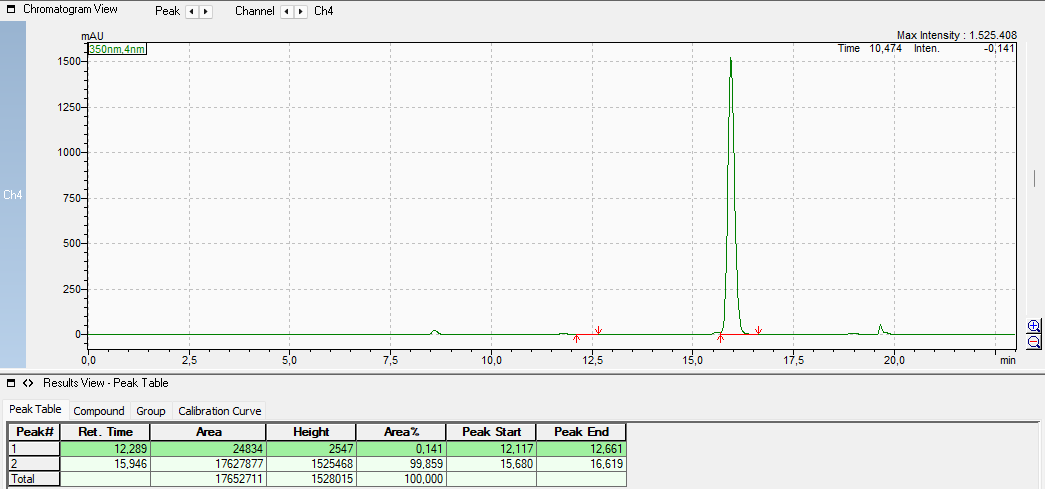


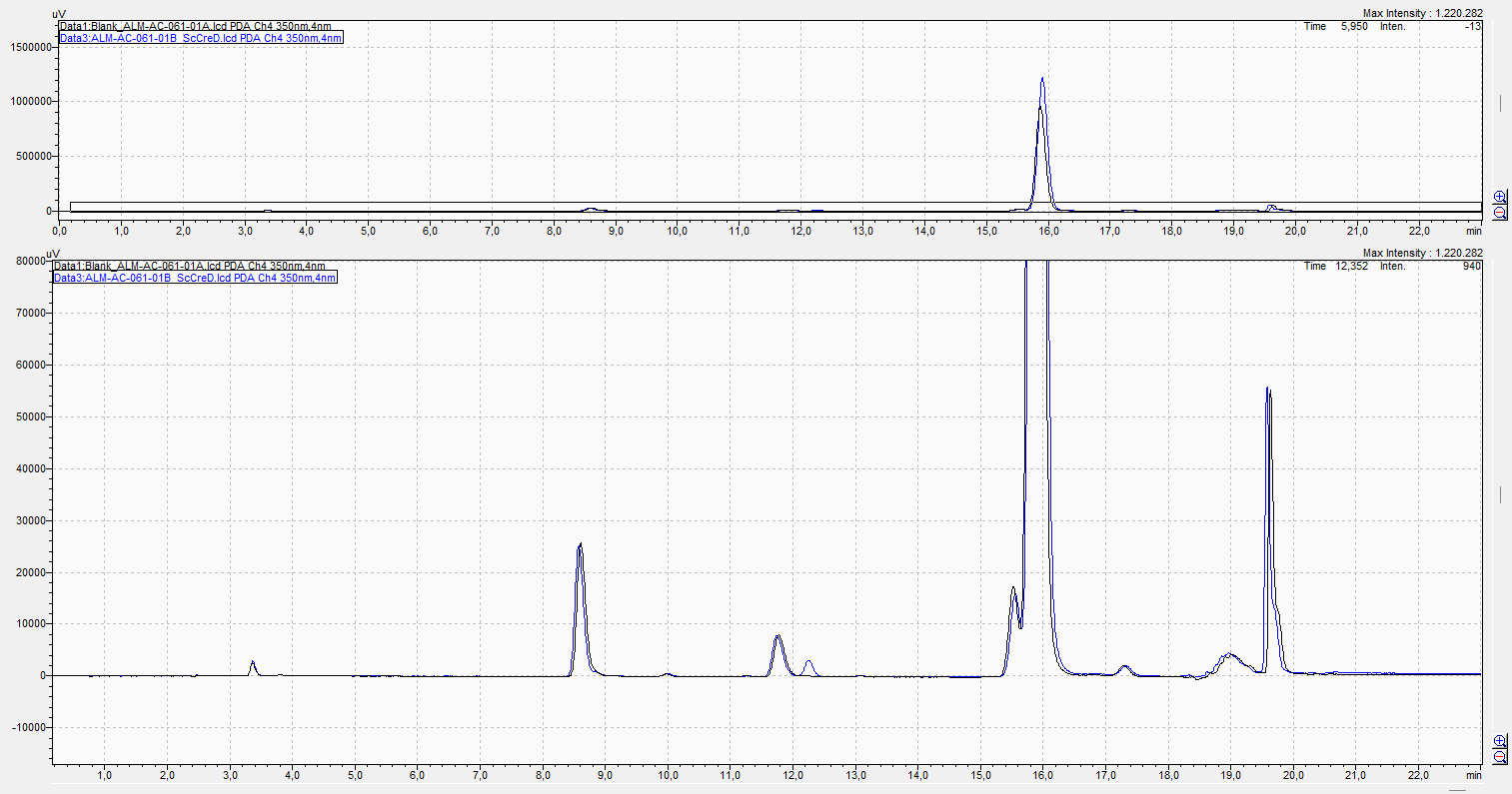


Figure S5.32: hydroamination reaction catalyzed by ScCreD. Retention times: 3.2 min fumaric acid, 10.0 min 1-(4-hydroxy-3-methylphenyl)ethan-1-one (IS), 12.2 min aspartic acid (derivatized). Analysis performed at 215 nm (first chromatogram), 254 nm (second chromatogram), 350 nm (third chromatogram, and bottom: overlayed chromatograms, black: reaction control without enzyme, blue: biotransformation)


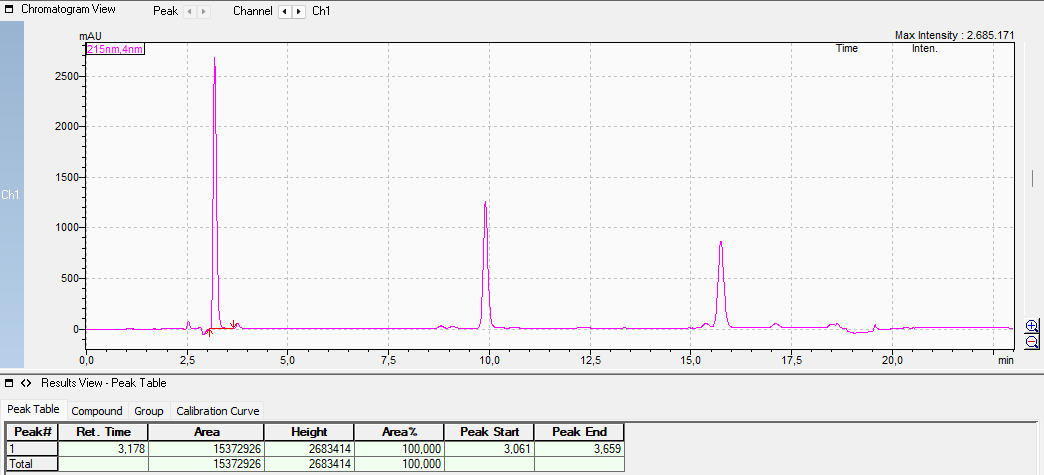


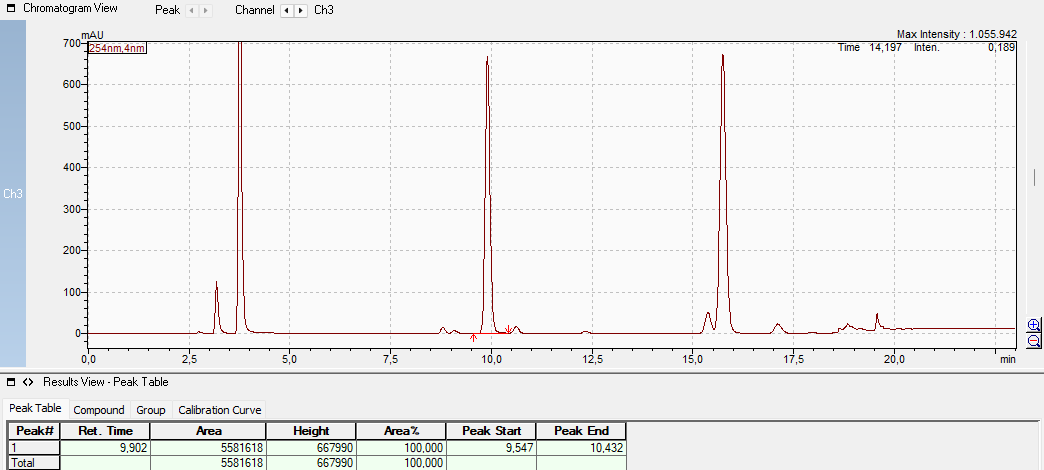


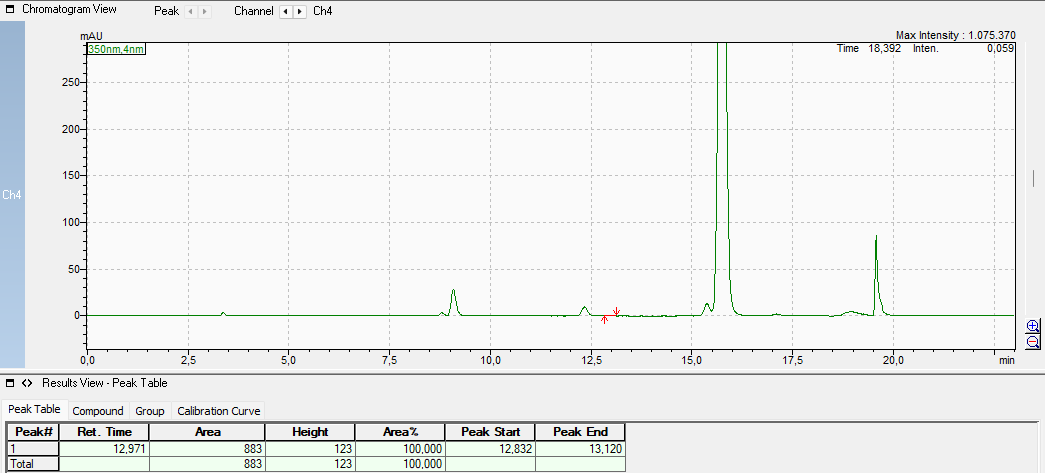


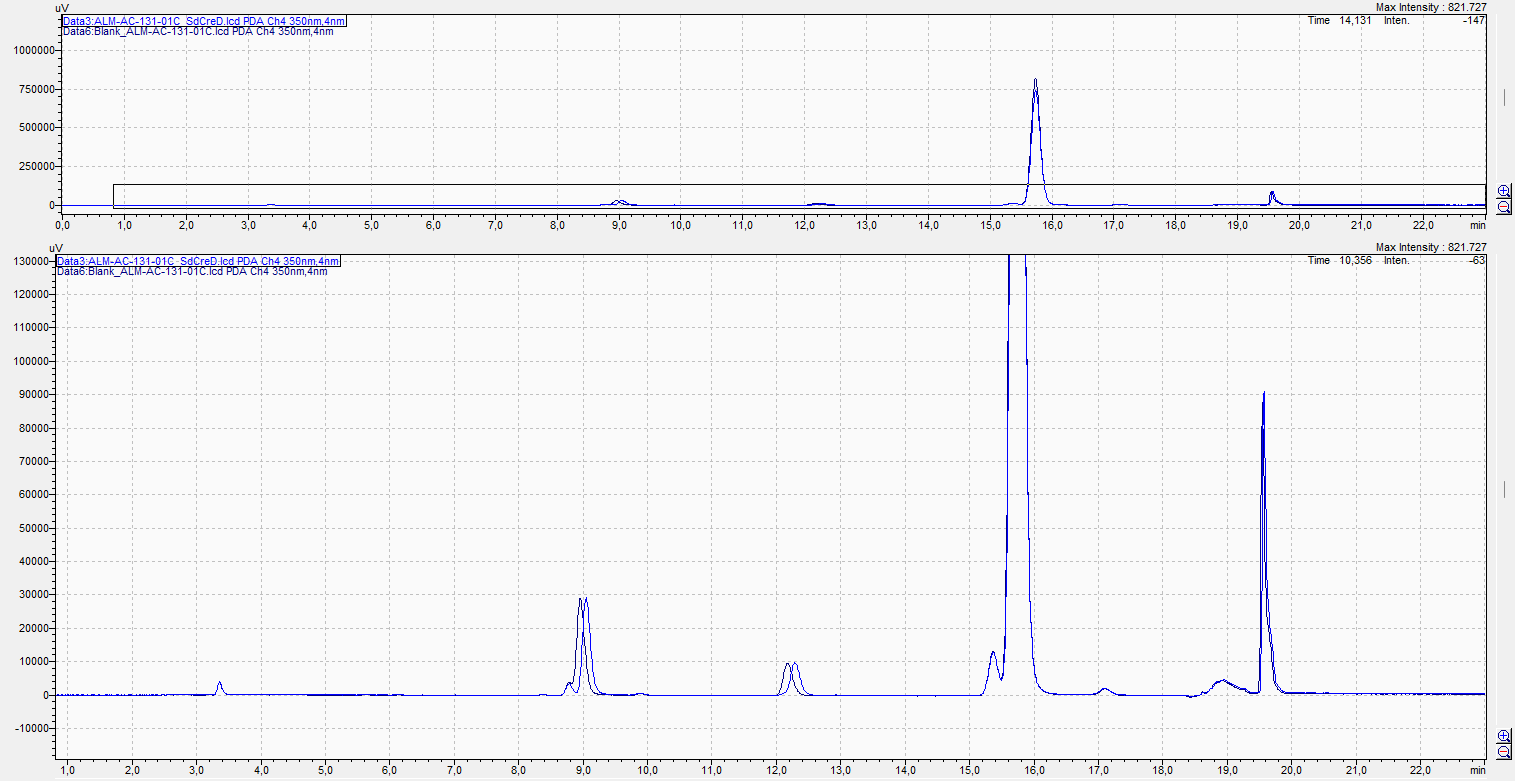


Figure S5.33: Hydroamination reaction catalyzed by SdCreD. T_ret_: 3.2 min fumaric acid, 10.0 min 1-(4-hydroxy-3-methylphenyl)ethan-1-one (IS), 12.9 min aspartic acid (derivatized). Analysis performed at 215 nm (first chromatogram), 254 nm (second chromatogram), 350 nm (third chromatogram), 350 nm overlayed (below; black: reaction control without enzyme, blue: biotransformation)


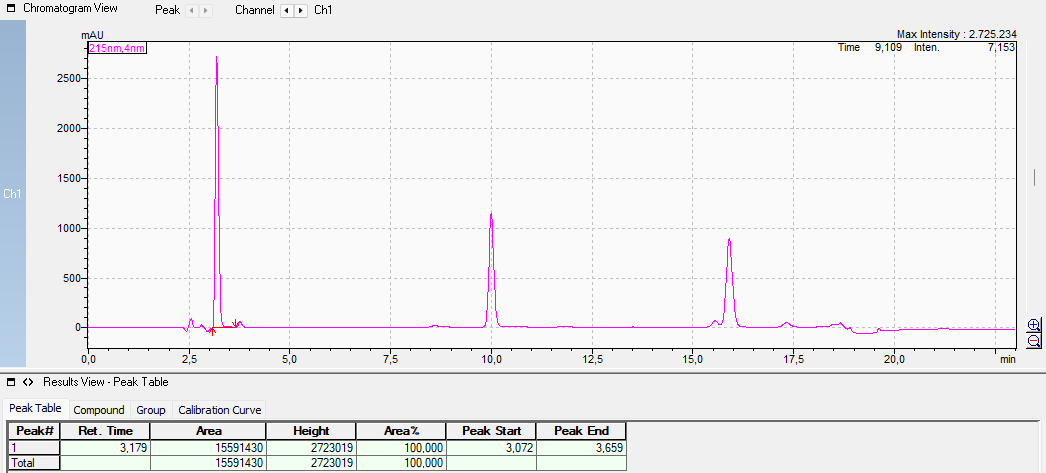


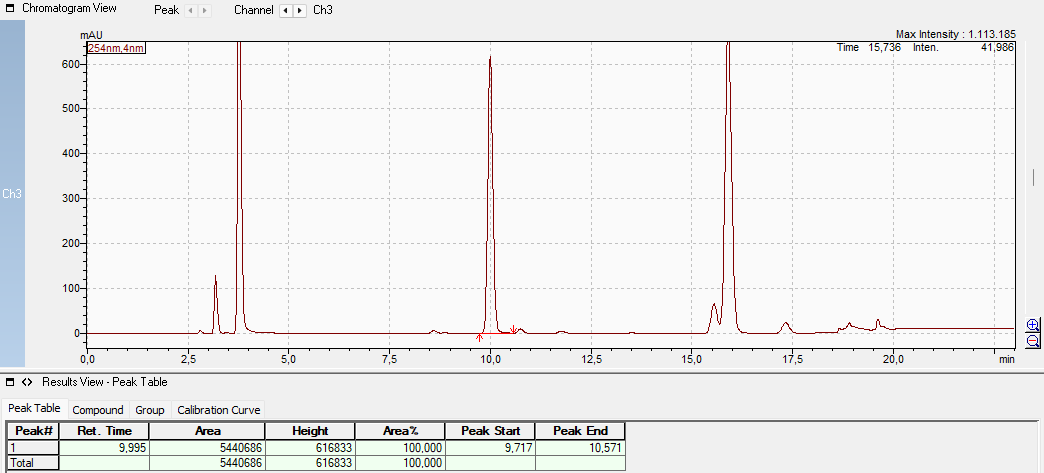


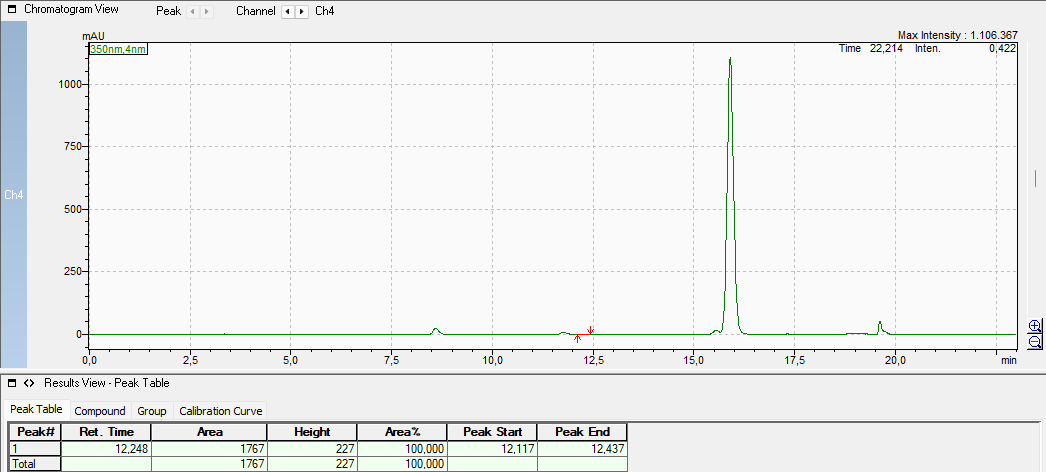


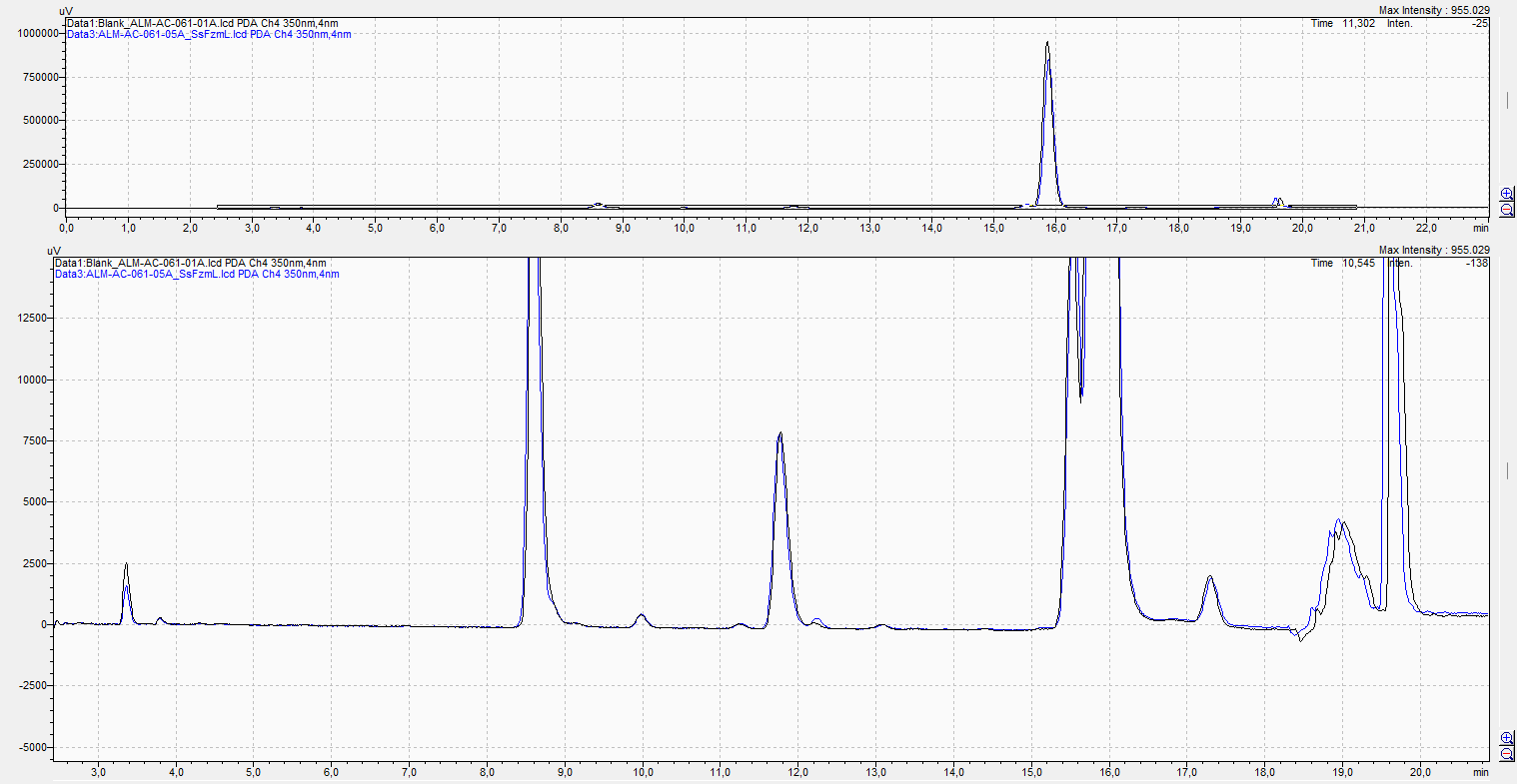


Figure S5.34: Hydroamination reaction catalyzed by SsFzmL. Retention times: 3.2 min fumaric acid, 10.0 min 1-(4-hydroxy-3-methylphenyl)ethan-1-one (IS), 12.2 min aspartic acid (derivatized). Analysis performed at 215 nm (first chromatogram), 254 nm (second chromatogram), 350 nm (third chromatogram, and bottom: overlayed chromatograms, black: reaction control without enzyme, blue: biotransformation)

### HPLC-MS traces (analyses performed with **Method MS-03**)

Note: only HPLC traces of reaction samples showing hydration activity are reported.


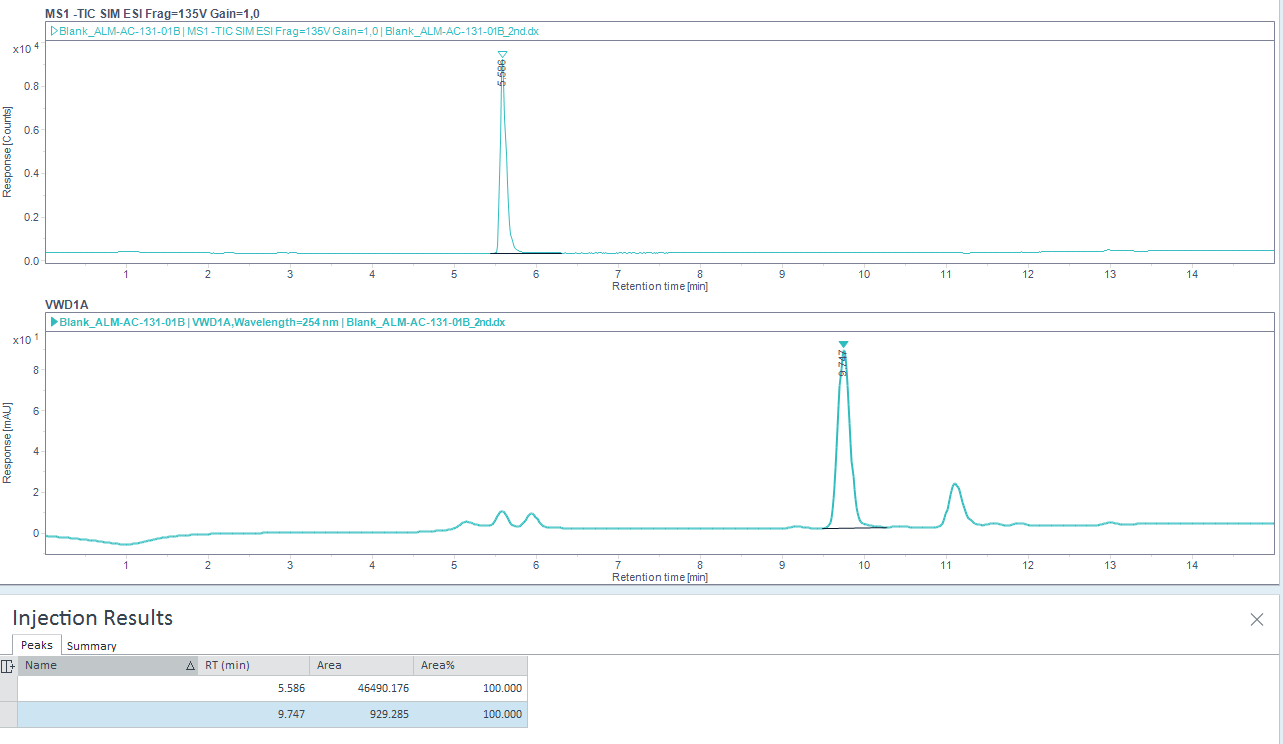


Figure S5.35: Hydroamination reaction control without enzyme. Retention times: 5.6 min fumaric acid, 9.7 min 1-(4-hydroxy-3-methylphenyl)ethan-1-one (IS). Analysis performed in SIM mode (top; M-1, negative mode; m/z 115, 133 for the detection of malic acid), and at 254 nm (bottom).


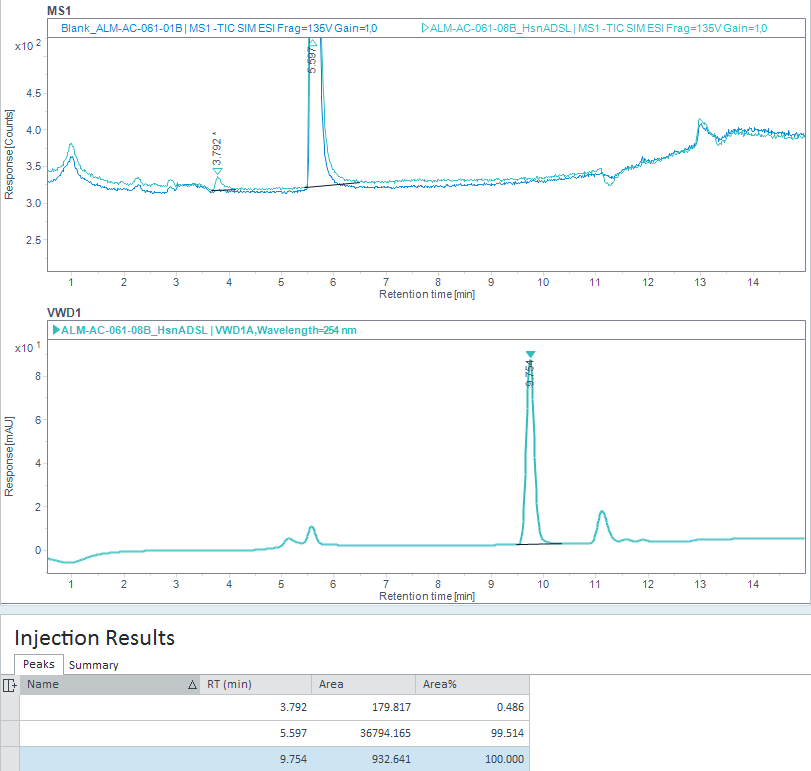


Figure S5.36: Hydroamination reaction catalyzed by HsnADSL. Retention times: 3.8 malic acid, 5.6 min fumaric acid, 9.7 min 1-(4-hydroxy-3-methylphenyl)ethan-1-one (IS). Analysis performed in SIM mode (top; M-1, negative mode; m/z 115, 133 for the detection of malic acid) and at 254 nm (bottom). Overlay with blue: reaction control without enzyme, green: biotransformation.


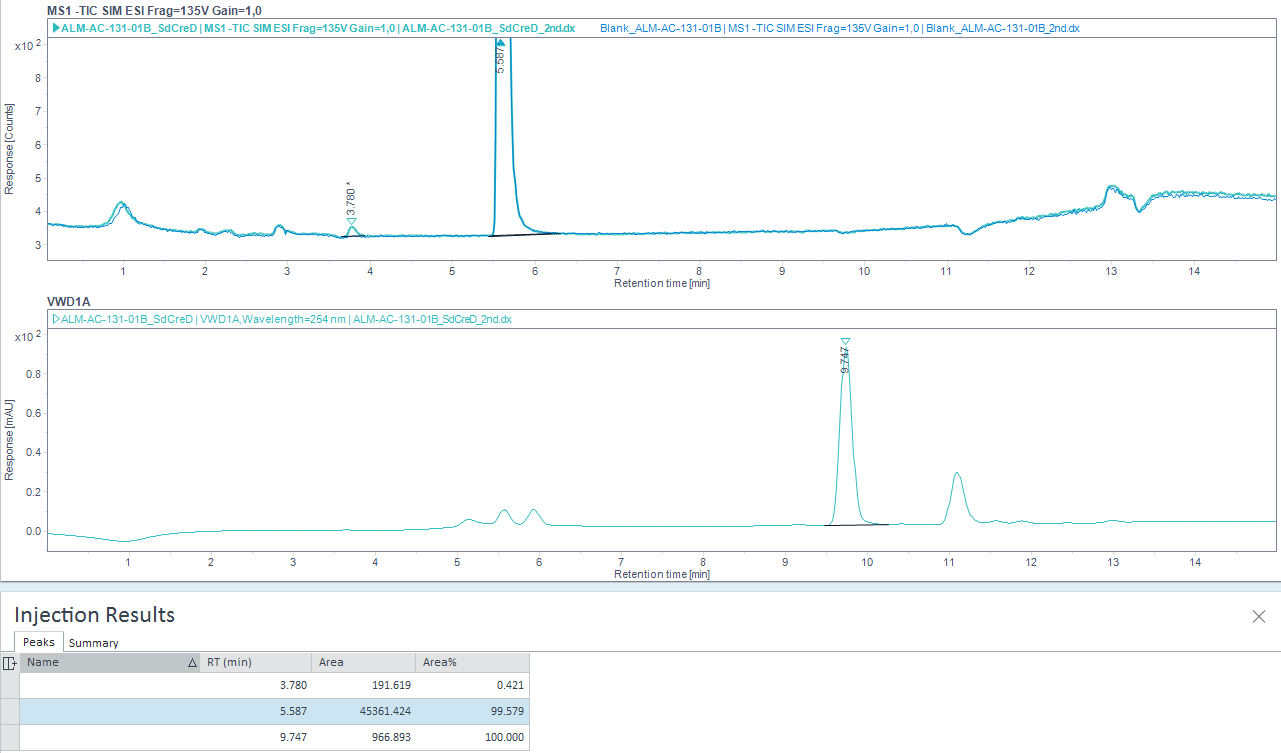


Figure S5.37: Hydroamination reaction catalyzed by SdCreD. Retention times: 3.8 malic acid, 5.6 min fumaric acid, 9.7 min 1-(4-hydroxy-3-methylphenyl)ethan-1-one (IS). Analysis performed in SIM mode (top; M-1, negative mode; m/z 115, 133 for the detection of malic acid) and at 254 nm (bottom). Overlay with blue: reaction control without enzyme, green: biotransformation.


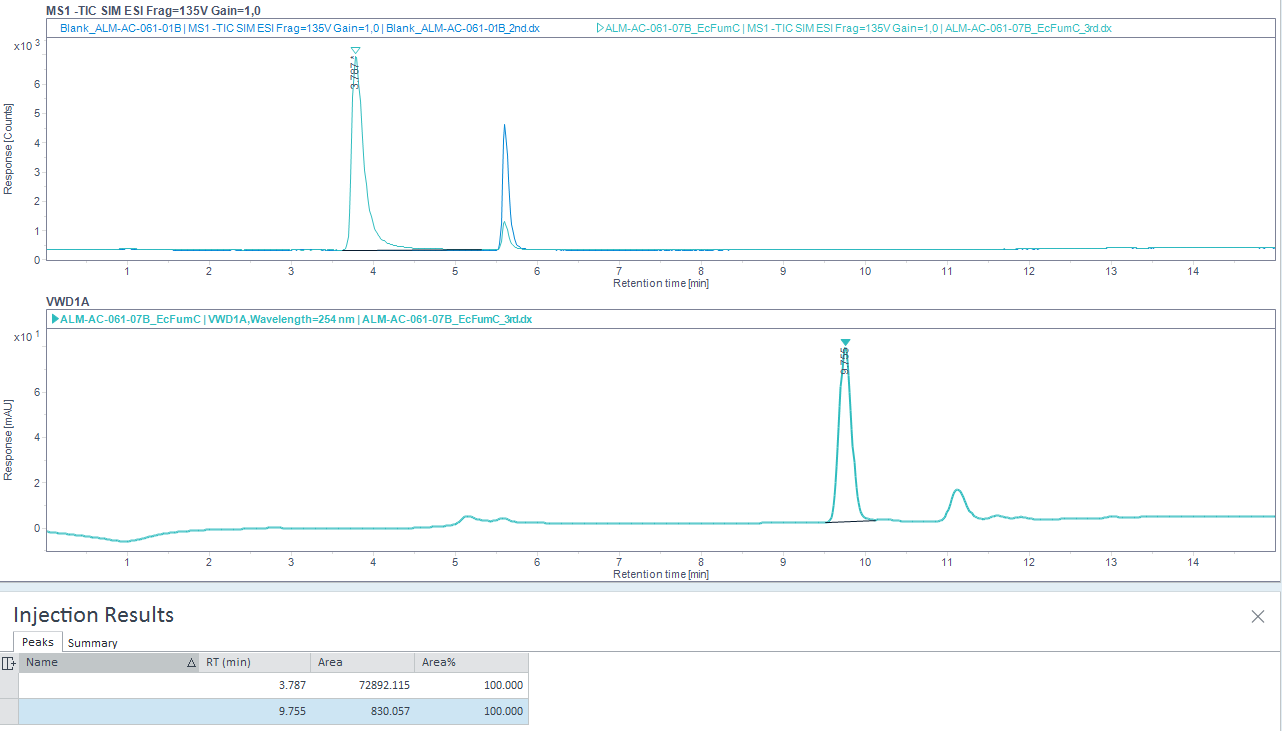


Figure S5.38: Hydroamination reaction catalyzed by EcFumC. Retention times: 3.8 malic acid, 5.6 min fumaric acid, 9.7 min 1-(4-hydroxy-3-methylphenyl)ethan-1-one (IS). Analysis performed in SIM mode (top; M-1, negative mode; m/z 115, 133 for the detection of malic acid) and at 254 nm (bottom). Overlay with blue: reaction control without enzyme, green: biotransformation.

## Experimental procedure - hydration reaction

The reaction mixtures were prepared by combining in deionized water, in order: sodium fumarate_(s)_ (to reach final concentration of 50 mM), HEPES_(s)_ (to reach final concentration of 400 mM). The pH was adjusted to 7.0 with NaOH_(aq)_ (10 M) and the enzyme solution was added to reach final concentration of 10 μM in a total volume of 500 µL. The reactions were incubated in an orbital shaker for 18 h at 30 °C (120 rpm). Then, 25 µL of the reaction were quenched with HCl_(aq)_ (200 µL, 1 M) and diluted with deionized water (275 µL, spiked with IS, 10 mM, pH 8), centrifuged (18,000 rpm, 2 min) and filtered with cotton. **1** and **4** were quantified with HPLC-MS (Method MS-04).

## Preparation of the calibration curves

### Sodium fumarate and *rac-*malic acid

The calibration curves (Figure S5.39Figure S5.40) were obtained according to the following procedures. A solution containing sodium fumarate_(s)_ or *rac*-malic acid_(s)_ (50 mM) in a mixture of *Sc*CreD storage buffer and HEPES_(aq)_ buffer (final concentration 400 mM) was prepared, and the pH adjusted to 7.0 with NaOH_(aq)_ (10 M). The ratio of *Sc*CreD storage buffer and reaction buffer was the same as in the biotransformations. This solution was diluted with the storage/HEPES buffer mixture to reach the concentrations reported in Figure S5.39 and Figure S5.40. The samples were incubated for 3 h (30 °C, 120 rpm), 25 μL of the mixture were worked up according to the standard procedure (see Section 5.8) and **1** and **4** were quantified with HPLC-MS (Method MS-04).

### Calibration curves

| \| [1a]  (mM) \| Normalized area^1^ \| \| --- \| --- \| \| 0.1 \| 0.06 ± 0.01 \| \| 1 \| 0.40 \| \| 2.5 \| 0.92 ± 0.01 \| \| 5 \| 1.82 ± 0.03 \| \| 10 \| 3.48 ± 0.13 \| \| 25 \| 8.23 ± 0.03 \| \| 50 \| 17.06 ± 0.05 \| |  |
| --- | --- | --- | --- | --- | --- | --- | --- | --- | --- | --- | --- | --- | --- | --- | --- | --- | --- |

Figure S5.39: Calibration curve for fumarate. Left: table with (normalized area) vs [sodium fumarate] (Note: if not indicated, RMSD < 0.01); Right: plot of the calibration points in the linear range. **^1^**: the calibration points were plotted as arithmetic average of triplicates calculated as [counts]_sodium fumarate_/[counts]_IS_. The error bars are the standard deviation between the triplicates. If not displayed error bars are smaller than the data points.

| \| [4]  (mM) \| Normalized area^1^ \| \| --- \| --- \| \| 0.1 \| 0.12 ± 0.02 \| \| 1 \| 1.60 ± 0.03 \| \| 2.5 \| 3.88 ± 0.04 \| \| 5 \| 8.11 ± 0.19 \| \| 10 \| 14.88 ± 0.20 \| \| 25 \| 33.70 ± 0.49 \| \| 50 \| 58.17 ± 0.24 \| |  |
| --- | --- | --- | --- | --- | --- | --- | --- | --- | --- | --- | --- | --- | --- | --- | --- | --- | --- |

Figure S5.40: Calibration curve for rac-malic acid. Left: table with (normalized area) vs [malic acid]; Right: plot of the calibration points in linear range. **^1^**: the calibration points were plotted as arithmetic average of triplicates calculated as [counts]_malic acid_/[counts]_IS_. The error bars are the standard deviation between the triplicates. If not displayed error bars are smaller than the data points.

## Chromatograms

### HPLC-MS traces (analyses performed with **Method MS-04**)


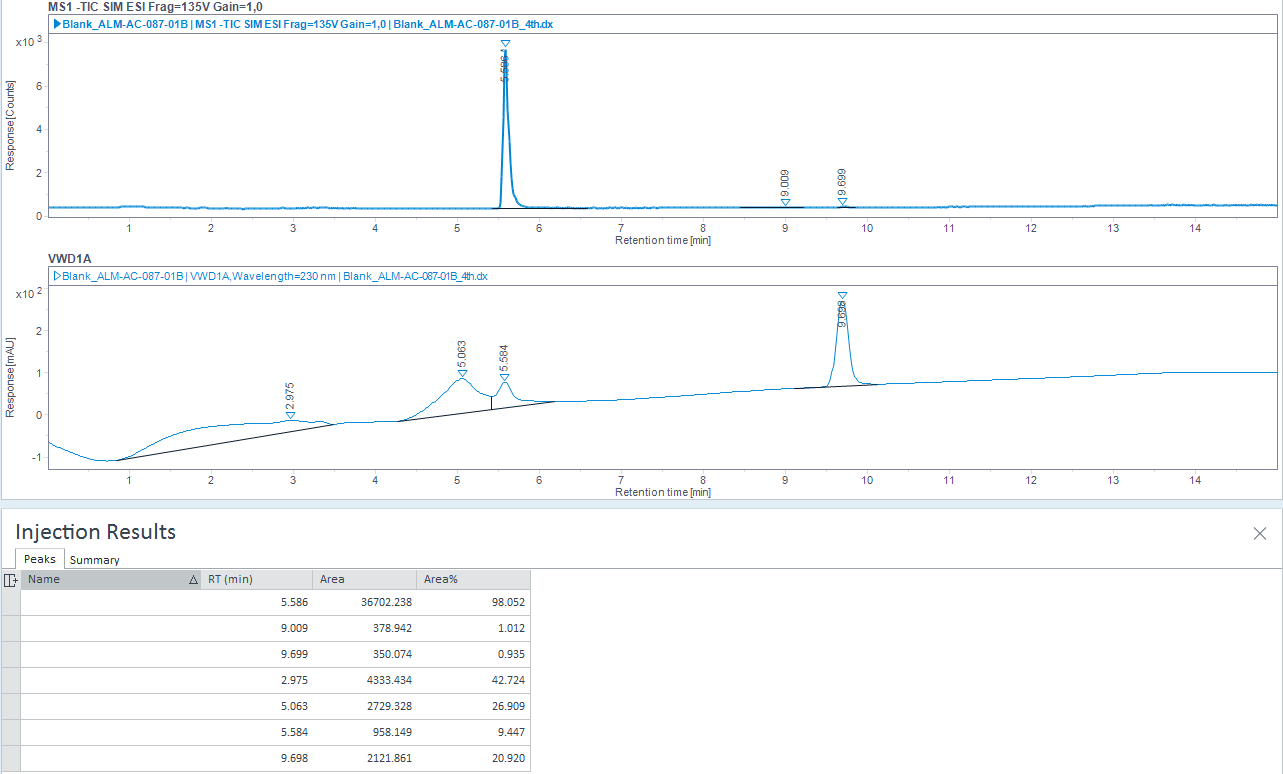


Figure S5.41: Hydration reaction control without enzyme. Retention times: 5.6 min fumaric acid, 9.7 min benzoic acid (IS). Analysis performed in SIM mode (top; M-1, negative mode; m/z 115 for the determination of fumaric acid, 133 for the determination of malic acid), and at 230 nm (bottom).


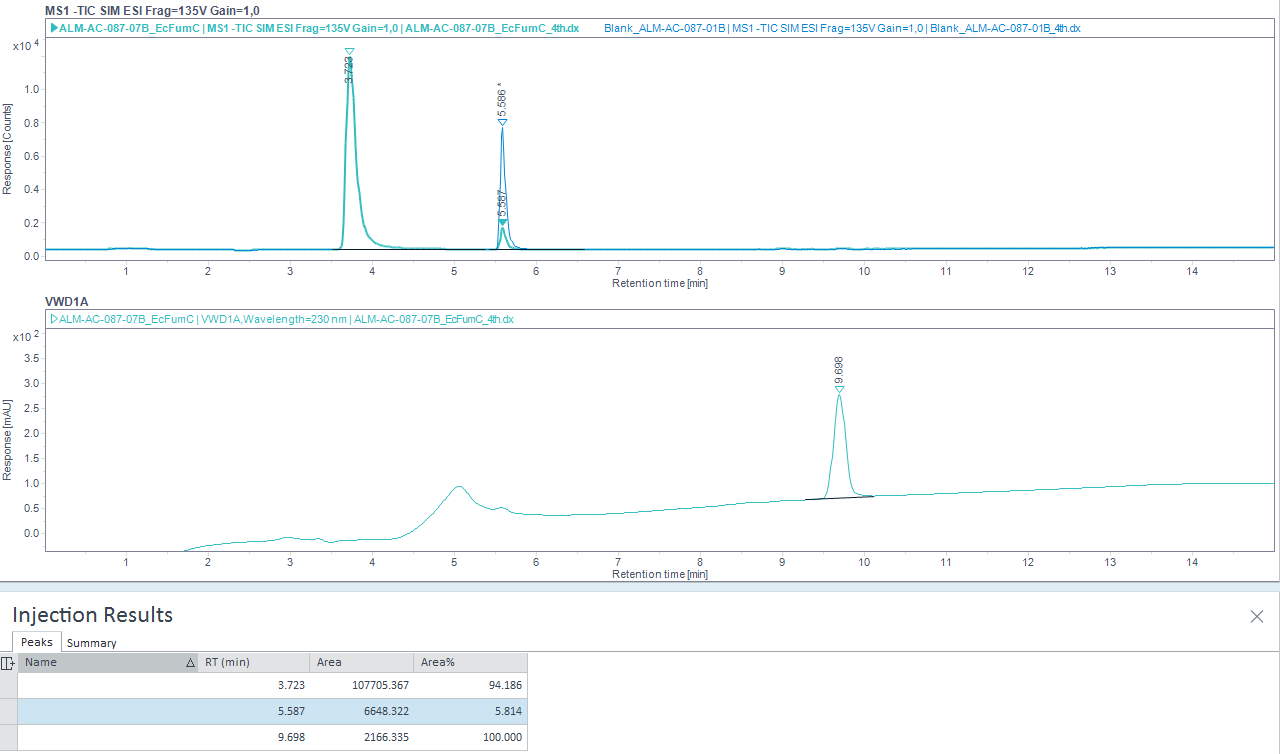


Figure S5.42: Hydration reaction catalyzed by EcFumC. Retention times: 3.7 malic acid, 5.6 min fumaric acid, 9.7 min benzoic acid (IS). Analysis performed in SIM mode (top; M-1, negative mode; m/z 115 for the determination of fumaric acid, 133 for the determination of malic acid) with overlay (blue: reaction control without enzyme, green: biotransformation), and at 230 nm (bottom).


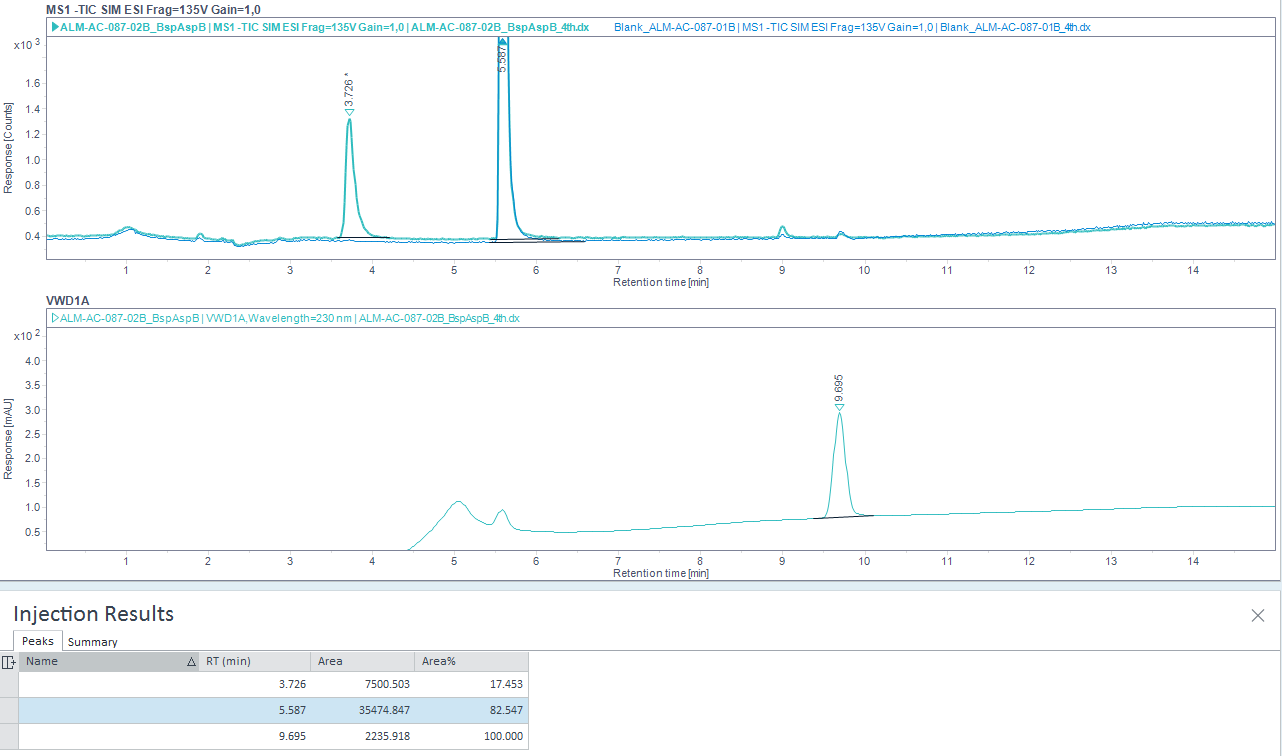


Figure S5.43: Hydration reaction catalyzed by BspAspB. Retention times: 3.7 malic acid, 5.6 min fumaric acid, 9.7 min benzoic acid (IS). Analysis performed in SIM mode (top; M-1, negative mode; m/z 115 for the determination of fumaric acid, 133 for the determination of malic acid) with overlay (blue: reaction control without enzyme, green: biotransformation), and at 230 nm (bottom).

Figure S5.44: Hydration reaction catalyzed by HsnADSL. Retention times: 3.7 malic acid, 5.6 min fumaric acid, 9.7 min benzoic acid (IS). Analysis performed in SIM mode (top; M-1, negative mode; m/z 115 for the determination of fumaric acid, 133 for the determination of malic acid) with overlay (blue: reaction control without enzyme, green: biotransformation), and at 230 nm (bottom).

Figure S5.45: Hydration reaction catalyzed by KaAzpD. Retention times: 3.7 malic acid, 5.6 min fumaric acid, 9.7 min benzoic acid (IS). Analysis performed in SIM mode (top; M-1, negative mode; m/z 115 for the determination of fumaric acid, 133 for the determination of malic acid) with overlay (blue: reaction control without enzyme, green: biotransformation), and at 230 nm (bottom).

Figure S5.46: Hydration reaction catalyzed by MtASL. T_ret_: 3.7 malic acid, 5.6 min fumaric acid, 9.7 min benzoic acid (IS). Analysis performed in SIM mode (above; M-1, negative mode; m/z 115 for the determination of fumaric acid, 133 for the determination of malic acid) overlay (blue: reaction control without enzyme, green: biotransformation), 230 nm (below).

Figure S5.47: Hydration reaction catalyzed by PaFlcC. Retention times: 3.7 malic acid, 5.6 min fumaric acid, 9.7 min benzoic acid (IS). Analysis performed in SIM mode (top; M-1, negative mode; m/z 115 for the determination of fumaric acid, 133 for the determination of malic acid) with overlay (blue: reaction control without enzyme, green: biotransformation), and at 230 nm (bottom).

Figure S5.48: Hydration reaction catalyzed by PpCMLE. Retention times: 3.7 malic acid, 5.6 min fumaric acid, 9.7 min benzoic acid (IS). Analysis performed in SIM mode (top; M-1, negative mode; m/z 115 for the determination of fumaric acid, 133 for the determination of malic acid) with overlay (blue: reaction control without enzyme, green: biotransformation), and at 230 nm (bottom).

Figure S5.49: Hydration reaction catalysed by ScCreD. Retention times: 3.7 malic acid, 5.6 min fumaric acid, 9.7 min benzoic acid (IS). Analysis performed in SIM mode (top; M-1, negative mode; m/z 115 for the determination of fumaric acid, 133 for the determination of malic acid) with overlay (blue: reaction control without enzyme, purple: biotransformation), and at 230 nm (bottom).

Figure S5.50: Hydration reaction catalyzed by SdCreD. Retention times: 3.7 malic acid, 5.6 min fumaric acid, 9.7 min benzoic acid (IS). Analysis performed in SIM mode (top; M-1, negative mode; m/z 115 for the determination of fumaric acid, 133 for the determination of malic acid) with overlay (blue: reaction control without enzyme, green: biotransformation), and at 230 nm (bottom).

Figure S5.51: Hydration reaction catalyzed by SsFzmL. Retention times: 3.7 malic acid, 5.6 min fumaric acid, 9.7 min benzoic acid (IS). Analysis performed in SIM mode (top; M-1, negative mode; m/z 115 for the determination of fumaric acid, 133 for the determination of malic acid) with overlay (blue: reaction control without enzyme, green: biotransformation), and at 230 nm (bottom).

# Study of the variants - Nitration reaction

## Experimental procedure – nitration reaction

The reaction mixtures were prepared in 96-well microtiter plates by combining in deionized water, in order: sodium fumarate_(s)_ (to reach final concentration of 50 mM), NaNO_2(s)_ (to reach final concentration of 500 mM), HEPES_(s)_ (to reach final concentration of 400 mM). The pH was adjusted to 7.0 with NaOH (10 M) and the enzyme solution was added to reach final concentration of 10 μM in a total volume of 200 µL. The reactions were incubated in a thermoshaker for 18 h at 30 °C (400 rpm). Then, in a 2 mL 96-deep well plate, 25 µL of the reaction were mixed with a solution of urea_(aq)_ (125 µL, 0.5 M; spiked with crotonic acid (IS) 5 mM), HCl_(aq)_ (100 µL, 1 M) and incubated in benchtop shaker (40 °C, 1 h, 500 rpm). Then, the reactions were centrifuged (4,000 rpm, 1 min) and transferred to 96-well microtiter plates for analysis. The solutions were analyzed with HPLC-UV (Method UV-03), measuring the relative activity on formation of 3-nitropropanoic acid, and HPLC-MS (Method MS-05), determining the relative activity on formation of malic acid.

## Chromatograms

### HPLC-UV traces (analyses performed with **Method UV-03**)

Figure S6.1: Reaction control for hydronitration reaction without the enzyme. Retention times: 6.4 min fumaric acid, 11.1 min crotonic acid (IS). Analysis performed at 215 nm.

Figure S6.2: Hydronitration reaction catalyzed by ScCreD wildtype. Retention times: 6.4 min fumaric acid, 7.3 min 3-nitropropanoic acid, 11.1 min crotonic acid (IS). Analysis performed at 215 nm (top), overlayed (bottom; black: reaction control without enzyme, pink: biotransformation).

Figure S6.3: Hydronitration reaction catalysed by ScCreD_H118N. Retention times: 6.4 min fumaric acid, 7.3 min 3-nitropropanoic acid, 11.1 min crotonic acid (IS). Analysis performed at 215 nm (top), zoom on the product (middle), overlayed (bottom; black: reaction control without enzyme, pink: biotransformation).

Figure S6.4: Hydronitration reaction catalysed by ScCreD_E49Q. Retention times: 6.4 min fumaric acid, 7.3 min 3-nitropropanoic acid, 11.1 min crotonic acid (IS). Analysis performed at 215 nm (top), zoom on the product (middle), overlayed (bottom; black: reaction control without enzyme, pink: biotransformation).

Figure S6.5: Hhydronitration reaction catalysed by ScCreD_S123C. Retention times: 6.4 min fumaric acid, 7.3 min 3-nitropropanoic acid, 11.1 min crotonic acid (IS). Analysis performed at 215 nm (top), overlayed (bottom; black: reaction control without enzyme, pink: biotransformation).

Figure S6.6: Hydronitration reaction catalysed by ScCreD_S123T. Retention times: 6.4 min fumaric acid, 7.3 min 3-nitropropanoic acid, 11.1 min crotonic acid (IS). Analysis performed at 215 nm (top), overlayed (bottom; black: reaction control without enzyme, pink: biotransformation).

Figure S6.7: Hydronitration reaction catalysed by ScCreD_T122A. Retention times: 6.4 min fumaric acid, 7.3 min 3-nitropropanoic acid, 11.1 min crotonic acid (IS). Analysis performed at 215 nm (top), overlayed (bottom; black: reaction control without enzyme, pink: biotransformation).

Figure S6.8: Hhydronitration reaction catalysed by ScCreD_V96A. Retention times: 6.4 min fumaric acid, 7.3 min 3-nitropropanoic acid, 11.1 min crotonic acid (IS). Analysis performed at 215 nm (top), overlayed (bottom; black: reaction control without enzyme, pink: biotransformation).

Figure S6.9: Hydronitration reaction catalysed by ScCreD_H347A. Retention times: 6.4 min fumaric acid, 7.3 min 3-nitropropanoic acid, 11.1 min crotonic acid (IS). Analysis performed at 215 nm (top), zoom on the product (middle), overlayed (bottom; black: reaction control without enzyme, pink: biotransformation).

Figure S6.10: Hydronitration reaction catalysed by ScCreD_P306G. Retention times: 6.4 min fumaric acid, 7.3 min 3-nitropropanoic acid, 11.1 min crotonic acid (IS). Analysis performed at 215 nm (top), zoom on the product (middle), overlayed (bottom; black: reaction control without enzyme, pink: biotransformation).

### HPLC-MS traces (analyses performed with Method MS-05)

Figure S6.11: Hydration reaction control without enzyme. Retention times: 5.6 min fumaric acid, 7.6 min crotonic acid (IS). Analysis performed in SIM mode (top; M-1, negative mode; m/z 115 & 133 for the monitoring of malic acid formation), and at 215 nm (bottom).

Figure S6.12: Hydration reaction catalyzed by ScCreD wild type under nitrating conditions. Retention times: 3.8 malic acid, 5.6 min fumaric acid, 7.6 min crotonic acid (IS). Analysis performed in SIM mode (top; M-1, negative mode; m/z 115 & 133 for the monitoring of malic acid formation) with overlay (blue: reaction control without enzyme, green: biotransformation), and at 215 nm (bottom).

Figure S6.13: Hydration reaction catalyzed by ScCreD_R341A under nitrating conditions. Retention times: 3.8 malic acid, 5.6 min fumaric acid, 7.6 min crotonic acid (IS). Analysis performed in SIM mode (top; M-1, negative mode; m/z 115 & 133 for the monitoring of malic acid formation) with overlay (blue: reaction control without enzyme, green: biotransformation), and at 215 nm (bottom).

Figure S6.14: Hydration reaction catalyzed by ScCreD_H253N under nitrating conditions. Retention times: 3.8 malic acid, 5.6 min fumaric acid, 7.6 min crotonic acid (IS). Analysis performed in SIM mode (top; M-1, negative mode; m/z 115 & 133 for the monitoring of malic acid formation) with overlay (blue: reaction control without enzyme, green: biotransformation), and at 215 nm (bottom).

Figure S6.15: Hydration reaction catalyzed by ScCreD_S123N under nitrating conditions. Retention times: 3.8 malic acid, 5.6 min fumaric acid, 7.6 min crotonic acid (IS). Analysis performed in SIM mode (top; M-1, negative mode; m/z 115 & 133 for the monitoring of malic acid formation) with overlay (blue: reaction control without enzyme, green: biotransformation), and at 215 nm (bottom).

Figure S6.16: Hydration reaction catalyzed by ScCreD_H118N under nitrating conditions. Retention times: 3.8 malic acid, 5.6 min fumaric acid, 7.6 min crotonic acid (IS). Analysis performed in SIM mode (top; M-1, negative mode; m/z 115 & 133 for the monitoring of malic acid formation) with overlay (blue: reaction control without enzyme, green: biotransformation), and at 215 nm (bottom).

Figure S6.17: Hydration reaction catalyzed by ScCreD_E49Q under nitrating conditions. Retention times: 3.8 malic acid, 5.6 min fumaric acid, 7.6 min crotonic acid (IS). Analysis performed in SIM mode (top; M-1, negative mode; m/z 115 & 133 for the monitoring of malic acid formation) with overlay (blue: reaction control without enzyme, green: biotransformation), and at 215 nm (bottom).

Figure S6.18: Hydration reaction catalyzed by ScCreD_R341K under nitrating conditions. Retention times: 3.8 malic acid, 5.6 min fumaric acid, 7.6 min crotonic acid (IS). Analysis performed in SIM mode (top; M-1, negative mode; m/z 115 & 133 for the monitoring of malic acid formation) with overlay (blue: reaction control without enzyme, green: biotransformation), and at 215 nm (bottom).

Figure S6.19: Hydration reaction catalyzed by ScCreD_S123C under nitrating conditions. Retention times: 3.8 malic acid, 5.6 min fumaric acid, 7.6 min crotonic acid (IS). Analysis performed in SIM mode (top; M-1, negative mode; m/z 115 & 133 for the monitoring of malic acid formation) with overlay (blue: reaction control without enzyme, green: biotransformation), and at 215 nm (bottom).

Figure S6.20: Hydration reaction catalyzed by ScCreD_S123Tunder nitrating conditions. Retention times: 3.8 malic acid, 5.6 min fumaric acid, 7.6 min crotonic acid (IS). Analysis performed in SIM mode (top; M-1, negative mode; m/z 115 & 133 for the monitoring of malic acid formation) with overlay (blue: reaction control without enzyme, green: biotransformation), and at 215 nm (bottom).

Figure S6.21: Hydration reaction catalyzed by ScCreD_T122A under nitrating conditions. Retention times: 3.8 malic acid, 5.6 min fumaric acid, 7.6 min crotonic acid (IS). Analysis performed in SIM mode (top; M-1, negative mode; m/z 115 & 133 for the monitoring of malic acid formation) with overlay (blue: reaction control without enzyme, green: biotransformation), and at 215 nm (bottom).

Figure S6.22: Hydration reaction catalyzed by ScCreD_V96A under nitrating conditions. Retention times: 3.8 malic acid, 5.6 min fumaric acid, 7.6 min crotonic acid (IS). Analysis performed in SIM mode (top; M-1, negative mode; m/z 115 & 133 for the monitoring of malic acid formation) with overlay (blue: reaction control without enzyme, green: biotransformation), and at 215 nm (bottom).

Figure S6.23: Hydration reaction catalyzed by ScCreD_H347A under nitrating conditions. Retention times: 3.8 malic acid, 5.6 min fumaric acid, 7.6 min crotonic acid (IS). Analysis performed in SIM mode (top; M-1, negative mode; m/z 115 & 133 for the monitoring of malic acid formation) with overlay (blue: reaction control without enzyme, green: biotransformation), and at 215 nm (bottom).

Figure S6.24: Hydration reaction catalyzed by ScCreD_W346A under nitrating conditions. Retention times: 3.8 malic acid, 5.6 min fumaric acid, 7.6 min crotonic acid (IS). Analysis performed in SIM mode (top; M-1, negative mode; m/z 115 & 133 for the monitoring of malic acid formation) with overlay (blue: reaction control without enzyme, green: biotransformation), and at 215 nm (bottom).

Figure S6.25: Hydration reaction catalyzed by ScCreD_P306G under nitrating conditions. Retention times: 3.8 malic acid, 5.6 min fumaric acid, 7.6 min crotonic acid (IS). Analysis performed in SIM mode (top; M-1, negative mode; m/z 115 & 133 for the monitoring of malic acid formation) with overlay (blue: reaction control without enzyme, green: biotransformation), and at 215 nm (bottom).

Figure S6.26: Hydration reaction catalyzed by ScCreD_K308A under nitrating conditions. Retention times: 3.8 malic acid, 5.6 min fumaric acid, 7.6 min crotonic acid (IS). Analysis performed in SIM mode (top; M-1, negative mode; m/z 115 & 133 for the monitoring of malic acid formation) with overlay (blue: reaction control without enzyme, green: biotransformation), and at 215 nm (bottom).

Figure S6.27: Hydration reaction catalyzed by ScCreD_S302A under nitrating conditions. Retention times: 3.8 malic acid, 5.6 min fumaric acid, 7.6 min crotonic acid (IS). Analysis performed in SIM mode (top; M-1, negative mode; m/z 115 & 133 for the monitoring of malic acid formation) with overlay (blue: reaction control without enzyme, green: biotransformation), and at 215 nm (bottom).

Figure S6.28: Hydration reaction catalyzed by ScCreD_S303A under nitrating conditions. Retention times: 3.8 malic acid, 5.6 min fumaric acid, 7.6 min crotonic acid (IS). Analysis performed in SIM mode (top; M-1, negative mode; m/z 115 & 133 for the monitoring of malic acid formation) with overlay (blue: reaction control without enzyme, green: biotransformation), and at 215 nm (bottom).

Figure S6.29: Hydration reaction catalysed by ScCreD_N310A under nitrating conditions. Retention times: 3.8 malic acid, 5.6 min fumaric acid, 7.6 min crotonic acid (IS). Analysis performed in SIM mode (top; M-1, negative mode; m/z 115 & 133 for the monitoring of malic acid formation) with overlay (blue: reaction control without enzyme, green: biotransformation), and at 215 nm (bottom).

Figure S6.30: Hydration reaction catalyzed by ScCreD_L170A under nitrating conditions. Retention times: 3.8 malic acid, 5.6 min fumaric acid, 7.6 min crotonic acid (IS). Analysis performed in SIM mode (top; M-1, negative mode; m/z 115 & 133 for the monitoring of malic acid formation) with overlay (blue: reaction control without enzyme, green: biotransformation), and at 215 nm (bottom).

Figure S6.31: Hydration reaction catalyzed by ScCreD_T122A_S123A under nitrating conditions. Retention times: 3.8 malic acid, 5.6 min fumaric acid, 7.6 min crotonic acid (IS). Analysis performed in SIM mode (top; M-1, negative mode; m/z 115 & 133 for the monitoring of malic acid formation) with overlay (blue: reaction control without enzyme, green: biotransformation), and at 215 nm (bottom).

Figure S6.32: Hydration reaction catalyzed by BspAspB wild type under nitrating conditions. Retention times: 3.8 malic acid, 5.6 min fumaric acid, 7.6 min crotonic acid (IS). Analysis performed in SIM mode (top; M-1, negative mode; m/z 115 & 133 for the monitoring of malic acid formation) with overlay (blue: reaction control without enzyme, green: biotransformation), and at 215 nm (bottom).

Figure S6.33: Hydration reaction catalyzed by BspAspB_L358R under nitrating conditions. Retention times: 3.8 malic acid, 5.6 min fumaric acid, 7.6 min crotonic acid (IS). Analysis performed in SIM mode (top; M-1, negative mode; m/z 115 & 133 for the monitoring of malic acid formation) with overlay (blue: reaction control without enzyme, green: biotransformation), and at 215 nm (bottom).

Figure S6.34: Hydration reaction catalyzed by BspAspB_L358R_N142Q under nitrating conditions. Retention times: 3.8 malic acid, 5.6 min fumaric acid, 7.6 min crotonic acid (IS). Analysis performed in SIM mode (top; M-1, negative mode; m/z 115 & 133 for the monitoring of malic acid formation) with overlay (blue: reaction control without enzyme, green: biotransformation), and at 215 nm (bottom).

# Substrate scope – hydronitration reaction

## Tested substrates

## Experimental procedure – substrate scope

The reaction mixtures were prepared in triplicates in 96-well microtiter plates by combining in deionized water, in order: the substrate (**15**-**29** to reach final concentration of 100 mM, 50 mM for **20**), NaNO_2(s)_ (to reach final concentration of 500 mM, 250 mM for **20**), HEPES_(s)_ (to reach final concentration of 400 mM). The pH was adjusted to 7.0 with NaOH (10 M) and the enzyme solution was added to reach final concentration of 50 μM (total volume of 200 µL). The samples were incubated in a thermoshaker for 18 h at 30 °C (400 rpm). For work-up, 25 µL of the reaction mixtures were mixed in a 2 mL 96-deep well plate with a solution of urea_(aq)_ (125 µL, 0.5 M; spiked with 3,3-dimethylacrylic acid (IS) 5 mM), HCl_(aq)_ (100 µL, 1 M) and incubated in a benchtop shaker (40 °C, 1 h, 500 rpm). The reactions were centrifuged (4,000 rpm, 1 min) and transferred to 96-well microtiter plates for analysis. The solutions were analyzed with HPLC-UV (Method UV-04) for the formation of new product peaks.

## Substrate concentration optimization on the best candidates (**28** and **29**)

The procedure 7.2 was performed on **28** and **29** at different concentrations (100 mM, 200 mM and 500 mM) with constant NaNO_2(aq)_ (500 mM) and HEPES_(aq)_ (400 mM). Reaction controls were performed in absence of sodium nitrite and/or in absence of enzyme. The normalized areas between the nitro-dependent peak (6.0 min for **28**, 7.0 min for **29**) and the internal standard were used to calculate the relative activities (100 mM samples as reference activity, Figure S7.1 and Figure S7.2).

Figure S7.1: optimization of the substrate loading for **28**. The tests were performed at three substrate concentrations (100, 200 and 500 mM, shown on the x-axis). In order: the reactions in hydronitration conditions (NaNO_2_ and ScCreD), reaction controls in absence of sodium nitrite (No NaNO_2_ and ScCreD) and the blanks without enzyme with (NaNO_2_ and no ScCreD) or without (No NaNO_2_ and no ScCreD) sodium nitrite. Relative activity was determined by calculating the ratio of the normalized peak area of the new product to that of the reference sample (100 mM substrate loading). The mean values and corresponding standard deviations were determined from triplicates.

Figure S7.2: optimization of the substrate loading for **29**. The tests were performed at three substrate concentrations (100, 200 and 500 mM, shown on the x-axis). In order: the reactions in hydronitration conditions (NaNO_2_ and ScCreD), reaction controls in absence of sodium nitrite (No NaNO_2_ and ScCreD) and the blanks without enzyme with (NaNO_2_ and no ScCreD) or without (No NaNO_2_ and no ScCreD) sodium nitrite. Relative activity was determined by calculating the ratio of the normalized peak area of the new product to that of the reference sample (100 mM substrate loading). The mean values and corresponding standard deviations were determined from triplicates.

## HPLC-UV traces (analyses performed with Method UV-04)

Figure S7.3: Hydronitration of **28** by ScCreD wildtype (200 mM substrate loading). Retention times: 2.3 min peaks derived from the nitrite quenching, 6.0 min new peak, 7.3 min **28**, 14.5 min 3,3-dimethylacrylic acid (IS). Analysis performed at 215 nm. The figure shows overlayed chromatograms (pink: reaction control without enzyme in presence of sodium nitrite, blue: reaction control without enzyme in absence of sodium nitrite, brown: biotransformation without sodium nitrite, black: biotransformation).

Figure S7.4: Hydronitration of **29** by ScCreD wildtype (200 mM substrate loading). Retention times: 2.3 min **29**, 4.6 min new peak, 7.0 min new peak, 14.5 min 3,3-dimethylacrylic acid (IS). Analysis performed at 215 nm. The figure shows overlayed chromatograms (black: reaction control without enzyme in presence of sodium nitrite, blue: reaction control without enzyme in absence of sodium nitrite, green: biotransformation without sodium nitrite, brown: biotransformation).

# Structural characterization of *Sc*CreD enzyme variants

**Size-exclusion chromatography (SEC)**

To evaluate structural changes arising from the introduced mutations and to assess potential effects on enzyme assembly and activity, size-exclusion chromatography (SEC) was performed. Following Ni–NTA affinity purification, proteins were further purified by size-exclusion chromatography using a Superdex™ 200 Increase 10/300 GL column (ÄKTA avant modern, Cytiva) equilibrated with buffer containing 150 mM K_2_HPO_4_ and 50 mM KCl (pH 8.0) at a flow rate of 0.67 mL/min. Elution was monitored at 280 nm, and fractions corresponding to the major peaks were analyzed by SDS–PAGE.

The elution profiles of the *Sc*CreD variants were compared with that of the wild-type (WT) enzyme. Among the 18 variants analyzed, three exhibited elution peaks preceding that of the WT, two eluted after it, and the remaining 13 showed identical elution volumes (Figure S8.1). The shift in peak elution of some variants indicates alterations in hydrodynamic radius, shape, or oligomeric state. Specifically, earlier elution of T122A_S123A, L170A, and H347A suggests an increase in radius or oligomeric state, whereas the later elution of H118N and S123N is consistent with a decrease (Table S8.1). Due to the lack of baseline separation and the minimal changes in elution volume, these effects are likely not caused by a change in oligomeric state but rather by partial unfolding or destabilization of the tetramer. For other members of the superfamily, we have observed far more pronounced effects upon alterations in oligomeric state using the same equipment (a shift of 0.98 mL in elution volume), which were later verified via SEC–MALS (data not shown). The fractions corresponding to the major peaks of all *Sc*CreD variants were analyzed by SDS–PAGE to assess their purity (Figure S8.2).

**Circular dichroism (CD) spectroscopy**

Circular dichroism spectroscopy was employed to examine potential alterations in the secondary structure of *Sc*CreD variants relative to the WT protein. For secondary structure analysis, protein samples purified by SEC were quantified spectrophotometrically at 280 nm using a NanoDrop™ 2000. CD spectra were recorded on a Chirascan Plus spectropolarimeter (Applied Photophysics) using 0.1 mm quartz cuvette. Measurements were performed at 25 °C over a wavelength range of 200–250 nm. Spectra were baseline-corrected with buffer (150 mM K_2_HPO_4_, 50 mM KCl, pH 8.0) and smoothed using the manufacturer’s default algorithm. Secondary structure composition was estimated from the CD spectra using the BeStSel web server.^[14]^

All variants displayed characteristic α-helical spectra with two negative peaks at 208 and 222 nm. The CD spectra were further analyzed using the BeStSel web server to estimate the secondary structure composition of each variant relative to the WT and PDB structures (Figure S8.3 and Table S8.2).

The ratio of ellipticity at 222 and 208 nm ($R=[\theta]_{222}/[\theta]_{208}$), which reflects α-helix organization and inter-helix coupling,^[15]^ was comparable to that of the WT ($R\geq1$) in most cases, consistent with a well-coupled helix-bundle oligomeric structure (Figure S8.3A and Table S8.3). In contrast, six variants exhibited reduced ellipticity at 222 nm and lower $R$ values ($R<1$), indicating weakened inter-helix coupling, affecting hydrodynamic radius, and suggesting alterations destabilizing the tetramer in oligomeric state, consistent with the SEC results for five of these variants. Variants T122A_S123A, L170A, and H347A, which eluted earlier than the WT, likely form assemblies with increased hydrodynamic radius, whereas H118N and S123N, which eluted later, may represent smaller or less associated forms. The remaining variant, H253N, eluted at a similar volume to the WT in SEC, indicating retention of the overall oligomeric state or hydrodynamic radius but a partially loosened helical-bundle organization (Figure S7.3B and Table S7.3). The CD measurements are in good agreement with the SEC results. Overall, the variants show the same content of secondary elements. Variants with an affected $R$ value are also likely to behave differently in the SEC due to a change in shape or hydrodynamic radius.

*Sc*CreD_WT

*Sc*CreD_E49Q

*Sc*CreD_H118N

*Sc*CreD_S123C

*Sc*CreD_S123N

*Sc*CreD_H253N

*Sc*CreD_R341K

*Sc*CreD_N310A

*Sc*CreD_S303A

*Sc*CreD_R341A

*Sc*CreD_K308A

*Sc*CreD_H347A

*Sc*CreD_W346A

*Sc*CreD_T122A

*Sc*CreD_L170A

*Sc*CreD_P306G

*Sc*CreD_S302A

*Sc*CreD_T122A_S123

*Sc*CreD_V96A

Figure S8.1: Size-exclusion chromatography (SEC) profiles showing the elution volumes of ScCreD variants relative to the wild type (WT) monitored at 280 nm.

Table S8.1: Elution volumes of ScCreD variants exhibiting shifts relative to the WT.

| Variant | Elution volume (mL) |
| --- | --- |
| T122A_S123A | 12.27 (-0.49) |
| L170A | 12.35 (-0.41) |
| H347A | 12.38 (-0.38) |
| *Sc*CreD_WT | 12.76 |
| S123N | 12.98 (+0,22) |
| H118N | 13.27 (+0.51) |

Figure S8.2: SDS–PAGE analysis of ScCreD variants compared with the wild-type (WT) protein after size-exclusion chromatography (SEC).

Figure S8.3: Circular dichroism (CD) spectra of ScCreD variants compared to the wild type (WT, shown in blue). **A.** Variant displaying a spectrum similar to the WT, with an ellipticity ratio R = [θ]_222_/[θ]_208_≥1; **B.** Variant exhibiting a distinct spectral profile relative to the WT, with R<1.

Table S8.2: CD data analysis using the BeStSel web server to estimate the secondary structure composition of each ScCreD variant relative to the wild type (WT), based on experimental spectra and crystal structure data.

| Protein variants | Helix | Antiparallel | Parallel | Turn | Others | RMSD |
| --- | --- | --- | --- | --- | --- | --- |
| [5XNY](https://www.rcsb.org/structure/5XNY#entity-1)  (PDB code) | 54.8 |  |  | 11.2 | 30.6 |  |
| [5XNZ](https://www.rcsb.org/structure/5XNZ#entity-1)  (PDB code) | 54.8 |  |  | 10.2 | 31.6 |  |
| WT | 52.2 | 0 | 0 | 9 | 38.8 | 0.0641 |
| V96A | 53.3 | 0 | 1.6 | 8.7 | 36.4 | 0.0896 |
| T122A_S123A | 54.5 | 0 | 0 | 11.7 | 33.9 | 0.0563 |
| T122A | 54.6 | 1.5 | 0 | 10.9 | 32.9 | 0.1391 |
| L170A | 54.5 | 0 | 0 | 10.3 | 35.2 | 0.0617 |
| S302A | 50.3 | 0.2 | 1 | 9.4 | 39.2 | 0.0945 |
| P306G | 50.5 | 1.5 | 0 | 9.9 | 38 | 0.1315 |
| R341K | 54.6 | 0 | 1.5 | 8.7 | 35.1 | 0.1039 |
| H347A | 55.8 | 0 | 0 | 10.4 | 33.8 | 0.0793 |
| K308A | 55.3 | 0 | 0.4 | 8.6 | 35.7 | 0.0973 |
| S303A | 51.2 | 0 | 0.3 | 8.2 | 40.4 | 0.1085 |
| R341K | 57.9 | 0.3 | 0 | 10 | 31.8 | 0.1369 |
| H253N | 54.5 | 0 | 0 | 9.9 | 35.6 | 0.0766 |
| S123N | 52.2 | 0 | 0 | 10.1 | 37.7 | 0.1108 |
| E49Q | 52.6 | 0 | 1.6 | 9.3 | 36.5 | 0.0786 |
| H118N | 56.6 | 4 | 0 | 11.3 | 28.1 | 0.2974 |

Table S8.3: Circular dichroism (CD) analysis of ScCreD variants relative to the wild type (WT), showing ellipticity ratios (R = [θ]_222_/[θ]_208_).

| Protein variants | Δε(208 nm) | Δε(222 nm) | R = \|Δε222\| / \|Δε208\| |
| --- | --- | --- | --- |
| ScCreD_WT | -6.2009 | -6.6277 | 1.069 |
| V96A | -6.4032 | -6.75 | 1.054 |
| R341K | -6.6132 | -6.9131 | 1.045 |
| K308A | -6.6702 | -6.9027 | 1.035 |
| S302A | -6.033 | -6.2416 | 1.035 |
| P306G | -6.1643 | -6.3744 | 1.034 |
| E49Q | -6.3691 | -6.554 | 1.029 |
| S303A | -6.5753 | -6.7169 | 1.022 |
| T122A | -6.3423 | -6.3761 | 1.005 |
| R341K | -6.7404 | -6.7392 | 1.000 |
| S123N | -6.7795 | -6.6933 | 0.987 |
| H253N | -6.4947 | -6.1141 | 0.941 |
| H347A | -6.5447 | -5.988 | 0.915 |
| H118N | -7.225 | -6.5643 | 0.909 |
| T122A_S123A | -6.3827 | -5.7453 | 0.900 |
| L170A | -6.5959 | -5.9319 | 0.899 |

# Alignment of protein sequences

The phylogenetic tree (Figure S8.1) and the sequence identity matrix (Figure S8.2) were obtained using the UniProt online tool (https://www.uniprot.org/align). The alignment of the lyase sequences is displayed in Figure S8.3.

Figure S9.1: Phylogenetic tree of the members of the aspartase/fumarase superfamily investigated in this study. In blue (left) the UniProt code of the lyase; in black (right) the enzyme name.

Figure S9.2: Sequence identity matrix of the investigated protein homologues. On the vertical axis: the UniProt code of the lyase (left) and the enzyme name (right) are given.

Figure S9.3: Amino acid sequence alignment (based on ClustalW in UniProt) of the investigated protein homologues. In blue (left) the UniProt code of the lyase; in black (right) the enzyme name. Highlighted residues according to similarity: dark purple highlight for very strong conservation, light purple highlight for less conserved but frequent residues.

# Analysis of tunnels among the studied proteins

## Procedure for the preparation of the structures

Protein structures were prepared using AlphaFold2 when experimentally resolved structures were unavailable in the Protein Data Bank (PDB, <https://www.rcsb.org/>) or when the characteristic SS-loop was not resolved and missing from the crystal structures, likely due to its high flexibility.

The polarity of the tunnels was determined as follows: For structures with available crystallographic data, electrostatic potentials were calculated using the APBS electrostatics^[16]^ plug-in in PyMOL 2.5.4. For AlphaFold2-derived models, electrostatic surfaces were generated using the “Vacuum electrostatics” command in PyMOL (see Table S8.1 for a general overview).

Table S10.1: Overview of available protein structures and relevant details for their analysis.

| Enzyme |  | PDB | Resolution | Loop resolved | Structure investigated | Determination of polarity |
| --- | --- | --- | --- | --- | --- | --- |
| PpCMLE |  | 1RE5  (Cit-enzyme complex) | 2.60 Å | Yes | 1RE5 | APBS electrostatics |
| PaFlcC |  | / | / | / | AlphaFold2 | Electrostatics in vacuum |
| BspAspB |  | 3R6V  (L-aspartate-enzyme complex) | 2.60 Å | Yes | 3R6V | APBS electrostatics |
|  |  | 3R6Q (apo-protein) | 2.40 Å | Yes |  |  |
|  |  | 1J3U (apo-protein) | 2.50 Å | Yes |  |  |
|  |  | 8RJ0 (apo-protein) | 1.90 Å | Yes |  |  |
| SspFzmL |  | / | / | / | AlphaFold2 | Electrostatics in vacuum |
| SdCreD |  | / | / | / | AlphaFold2 | Electrostatics in vacuum |
| ScCreD |  | 5XNZ  (Fum-enzyme complex) | 2.30 Å | No | AlphaFold2 | Electrostatics in vacuum |
|  |  | 5XNY (apo-protein) | 2.18 Å | No |  |  |
| EcFumC |  | 6NZ9  (Cit-enzyme complex) | 1.53 Å | Yes | AlphaFold2 | Electrostatics in vacuum |
|  |  | 1FUO  (Cit-enzyme complex) | 1.98 Å | Yes |  |  |
|  |  | 1FUP  (Pyr-enzyme complex) | 2.30 Å | Yes |  |  |
|  |  | 1FUQ  (Cit-enzyme complex) | 2.00 Å | Yes |  |  |
|  |  | 1YFE (apo-protein) | 2.19 Å | Yes |  |  |
| HsnADSL |  | 5NX8 (apo-protein) | 1.70 Å | Yes | 5NX9 | APBS electrostatics |
|  |  | 5NX9  (2SA- or Fum-AMP-  enzyme complex) | 2.30 Å | Yes |  |  |
|  |  | 5NXA  (SSS- or AMZ-FUM-  enzyme complex) | 2.40 Å | Yes |  |  |
| MtASL |  | 6IEM (apo-protein) | 2.20 Å | Yes | 6IEN | APBS electrostatics |
|  |  | 6IEN  (AS1- ARG-FUM-  enzyme complex) | 2.70 Å | Yes |  |  |
|  |  | 6IG5 (apo-protein) | 2.08 Å | Yes |  |  |
|  |  | 6IGA  (Sulphate-enzyme complex) | 2.78 Å | Yes |  |  |
| KaAzpD |  | / | / | / | AlphaFold2 | Electrostatics in vacuum |

The following labels correspond to the RCSB PDB website. *Fum* fumarate, *Cit* citrate, *Pyr* pyromellitic acid, *2SA* 2-[9-(3,4-dihydroxy-5-phosphonooxymethyl-tetrahydro-furan-2-yl)-9*H*-purin-6-ylamino]-succinic acid, *AMP* adenosine monophosphate, *SSS* *N*-[5-amino-1-(5-*O*-phosphono-beta-D-arabinofuranosyl)-1*H*-imidazol-4-yl]carbonyl-L-aspartic acid, *AMZ* aminoimidazole 4-carboxamide ribonucleotide, *AS1* argininosuccinate, *Arg* L-arginine.

## Analysis of tunnels and electrostatic potential surfaces

Tunnels in proximity to the active site and ligand binding were analyzed using CAVER 3.0^[17-18],^ (<http://www.caver.cz>). In all the representations, the loop is displayed as well as residues T122 and S123 (*Sc*CreD numbering) as reference points.

Figure S10.1: Analysis of predicted tunnels in PpCMLE. **A**. Cartoon representation of the crystal structure (PDB code: 1RE5) overlayed with putative tunnels predicted with CAVER 3.0. **B**. Tunnels without cartoon. **C**. Electrostatic potential surface of the cavity (Polarities represented as red negative, blue positive) overlayed with cartoon of the crystal structure of PpCMLE. **D**. Electrostatic potential surface without cartoon.

Figure S10.2: Analysis of predicted tunnels of PaFlcC. **A**. Cartoon representation of the tetramer (generated with Alphafold2) overlayed with putative tunnels predicted with Caver3.0. **B**. Detail of the caver output. **C**. Electrostatic surface of the cavity (Polarities represented as red negative, blue positive) overlayed with cartoon of the predicted AlphaFold2 model. **D**. Detail of the electrostatic surface. Note: for simplicity reasons the orientation of the four structures is identical. Highlighted loop and residues serve as spatial references to aid in orientation.

Figure S10.3: Analysis of predicted tunnels of BspAspB. **A**. Cartoon representation of the crystal structure (PDB code: 3R6V) overlayed with putative tunnels predicted with Caver3.0. **B**. Detail of the caver output. **C**. Electrostatic surface of the cavity (Polarities represented as red negative, blue positive) overlayed with cartoon of the crystal structure of BspAspB. **D**. Detail of the electrostatic surface. Note: for simplicity reasons the orientation of the four structures is identical. Highlighted loop and residues serve as spatial references to aid in orientation. In teal it is displayed L-aspartic acid as ligand in the investigated crystal structure.

Figure S10.4: Analysis of predicted tunnels of SspFzmL. **A**. Cartoon representation of the tetramer (generated with Alphafold2) overlayed with putative tunnels predicted with Caver3.0. **B**. Detail of the caver output. **C**. Electrostatic surface of the cavity (Polarities represented as red negative, blue positive) overlayed with cartoon of the predicted AlphaFold2 model. **D**. detail of the electrostatic surface. Note: for simplicity reasons the orientation of the four structures is identical. Highlighted loop and residues serve as spatial references to aid in orientation.

Figure S10.5: Analysis of predicted tunnels of SdCreD. **A**. Cartoon representation of the tetramer (generated with Alphafold2) overlayed with putative tunnels predicted with Caver3.0. **B**. Detail of the caver output. **C**. Electrostatic surface of the cavity (Polarities represented as red negative, blue positive) overlayed with cartoon of the predicted AlphaFold2 model. **D**. Detail of the electrostatic surface. Note: for simplicity reasons the orientation of the four structures is identical. Highlighted loop and residues serve as spatial references to aid in orientation.

Figure S10.6: Analysis of predicted tunnels of ScCreD. **A**. Cartoon representation of the tetramer (generated with Alphafold2) overlayed with putative tunnels predicted with Caver3.0. **B**. Detail of the caver output. **C**. Electrostatic surface of the cavity (Polarities represented as red negative, blue positive) overlayed with cartoon of the predicted AlphaFold2 model. D. Detail of the electrostatic surface. Note: for simplicity reasons the orientation of the four structures is identical. Highlighted loop and residues serve as spatial references to aid in orientation.

Figure S10.7: Analysis of predicted tunnels of EcFumC. **A**. Cartoon representation of the tetramer (generated with Alphafold2) overlayed with putative tunnels predicted with Caver3.0. **B**. Detail of the caver output. **C**. Electrostatic surface of the cavity (Polarities represented as red negative, blue positive) overlayed with cartoon of the predicted AlphaFold2 model. **D**. Detail of the electrostatic surface. Note: for simplicity reasons the orientation of the four structures is identical. Highlighted loop and residues serve as spatial references to aid in orientation.

Figure S10.8: Analysis of predicted tunnels of HsnADSL. **A**. Cartoon representation of the crystal structure (PDB code: 5NX9) overlayed with putative tunnels predicted with Caver3.0. **B**. Detail of the caver output. **C**. Electrostatic surface of the cavity (Polarities represented as red negative, blue positive) overlayed with cartoon of the crystal structure of HsnADSL. **D**. Detail of the electrostatic surface. Note: for simplicity reasons the orientation of the four structures is identical. Highlighted loop and residues serve as spatial references to aid in orientation. In green it is displayed adenosine monophosphate (AMP) while in grey fumarate as ligands in the investigated crystal structure.

Figure S10.9: Analysis of predicted tunnels of MtASL. **A**. Cartoon representation of the crystal structure (PDB code: 6IEN) overlayed with putative tunnels predicted with Caver3.0. **B**. Detail of the caver output. **C**. Electrostatic surface of the cavity (Polarities represented as red negative, blue positive) overlayed with cartoon of the crystal structure of HsnADSL. **D**. Detail of the electrostatic surface. Note: for simplicity reasons the orientation of the four structures is identical. Highlighted loop and residues serve as spatial references to aid in orientation. In grey and green it is displayed N-arginine succinic acid while in grey fumarate as ligands in the investigated crystal structure.

Figure S10.10: Analysis of predicted tunnels of KaAzpD. **A**. Cartoon representation of the tetramer (generated with Alphafold2) overlayed with putative tunnels predicted with Caver3.0. **B**. Detail of the caver output. **C**. Electrostatic surface of the cavity (Polarities represented as red negative, blue positive) overlayed with cartoon of the predicted Alphafold2 model. **D**. Detail of the electrostatic surface. Note: for simplicity reasons the orientation of the four structures is identical. Highlighted loop and residues serve as spatial references to aid in orientation.

# Chemical synthesis of 3-nitropropanoic acid

The procedure was adapted from the reported procedure used for the synthesis of dimethyl 2-nitrosuccinate.^[19]^

Under N_2_ atmosphere, 2-bromosuccinic acid (313.4 mg, 1.59 mmol), NaNO_2(s)_ (186.5 mg, 2.7 mmol) and phloroglucinol (186.5 mg, 1.47 mmol) were solubilized in DMF_(dry)_ (3.2 mL). The reaction was let stirring overnight. The mixture was washed with NaHCO_3(aq)_ (sat.), acidified with HCl (1 M) and extracted with EtOAc, washed with brine, dried over Na_2_SO_4(dry)_ and concentrated under vacuum. The crude was purified by flash chromatography (Eluent: Cyclohexane/EtOAc 6:4 + 1% TFA). The result was a mixture of fumaric acid (**1**, side product), 3-nitropropanoic acid (**11**) and 2-bromosuccinic acid (**33**). The mixture was again purified by flash chromatography (Eluent: Cyclohexane/EtOAc 1:1 + 1% AcOH), yielding 3-nitropropanoic acid as a light-yellow solid (28 mg, 15% isolated yield). The NMR data were in accordance with the literature (see section 10).^[20]^ ^1^H NMR (300 MHz, Methanol-*d*_4_) δ 4.67 (t, 2H; J: 6.0 Hz), 2.95 (t, 2H J: 6.0 Hz). ^13^C NMR (75 MHz, Methanol-*d*_4_) δ 172.08, 71.04, 31.73.

# Nuclear magnetic resonance (NMR)

**3-nitropropanoic acid obtained from the chemical synthesis**

^1^H NMR (300 MHz, Methanol-*d*_4_) δ 4.67 (t, *J* = 6 Hz, 1H), 2.95 (t, *J* = 6 Hz, 1H).

Figure S12.1: ^1^H-NMR of **11** obtained from chemical synthesis.

^13^C NMR (75 MHz, Methanol-*d*_4_) δ 172.08, 71.04, 31.73.

A.

B.

Figure S12.2: ^13^C-NMR NMR of **11** obtained from chemical synthesis. A. Full ^13^C-NMR spectrum; B. Zoom onto the characteristic peaks (173.5, 71.0, 31.7 ppm)

**Nitropropanoic acid isolated from the *Sc*CreD-catalyzed nitration reaction**

^13^C-NMR (75 MHz, methanol-*d*_4_) δ 173.5, 71.1, 31.8.

A.

B.

Figure S12.3: ^13^C-NMR of **11** obtained from scale-up of the hydronitration reaction. A. Full ^13^C-NMR spectrum; B. Zoom onto the characteristic peaks (173.5, 71.1, 31.8 ppm)

^1^H-NMR (300 MHz, methanol-*d*_4_): δ 4.68 (t, 2H), 2.95 (t, 2H).

1.

2.

3.

Figure S12.4: ^1^H-NMR of **11** obtained from scale-up of the hydronitration reaction. 1. Full ^1^H-NMR spectrum; 2. Zoom in the zone 1.9 - 5.3 ppm; 3. Top: spectrum from nitropropanoic acid obtained by chemical synthesis, bottom: spectrum of product isolated from the biotransformation (see Section 4.4)

**Identification of nitronate and nitrosuccinate during the enzymatic reaction**

For the NMR spectroscopic assignment of the nitrogen-containing reaction products, 1D ^1^H, 2D TOCSY (Figure S10.5) and 2D multiplicity-edited ^1^H-^13^C HSQC spectra (Figure S10.6) were recorded. The species, which is characterized by proton chemical shifts of 6.23 and 3.13 ppm, shows correlations to carbon chemical shifts of 114.9 ppm (the CH signal at 6.23 ppm) and 35.9 ppm (CH_2_ group at 3.13 ppm). This confirms its structure as the nitronate form of nitropropanoate. Nitrosuccinate can be assigned to the signals at 5.45 ppm (CH_2_) and 3.10 ppm (CH_2_). Confirmation of these structures was achieved by analyzing the products formed using ^15^N-labeled nitrite, showing the presence of additional signal splittings of the proton signals of the CH_2_ groups, which are separated by three bonds from the nitrogen atom (Figure S10.7).

Figure S12.5: 2D TOCSY showing the correlations of the nitronate (encircled in black) and nitrosuccinate (in green)

Figure S12.6: 2D ^1^H-^13^C-HSQC showing the correlations of the nitronate (encircled in black)

Figure S12.7: ^1^D 1H spectra of the intermediate products when using ^15^N-labeled nitrite. Additional signal splittings are observed for the -OOC-CH_2_ protons of nitronate (encircled in pink) and nitrosuccinate (in green). The insert shows two of the signals without splitting from the sample obtained in the reaction with non-labeled nitrite.

# Computational methods

## Molecular Dynamics (MD) simulations of *Sc*CreD

The tetrameric *Sc*CreD was simulated via classical molecular dynamics (MD) simulations in two conditions: in the presence of the substrates (fumarate, nitrite, Figure S11.1) and in the presence of the product of the nitration reaction ((*S*)-nitrosuccinate, Figure S11.2) at each of the four active sites. The force field parameters of Amber19SB were used to represent the protein residues in the system. The geometries of fumarate, (*S*)-nitrosuccinate, and nitrite were optimized using Density Functional Theory (DFT) and a double zeta basis set including the Grimme’s dispersion corrections^[21]^ with Gaussian16^[22]^ (BP86-D3/def2-SVP). AM1-BCC point charges^[23]^ were using antechamber (AmberTools24) on the previously optimized geometries. AMBER atom types were used to represent the substrate atoms. The protonation state at pH 7.5 of all titrable residues in *Sc*CreD were fixed by computing their p*K*_a_ values with PROPKA 3.5.0. The Cartesian coordinates of *Sc*CreD were collected from the AlphaFold2 model of the enzyme (AF-A0A0K2JL82-F1), which includes the missing SS loop residues of the crystal structure in complex with fumarate (PDB id. 5XNZ, 2.18 Å resolution). The orientation of the rest of substrates/products was done based on the orientation of fumarate in the former crystal structure. The two systems were embedded into a box of OPC water molecules^[24]^ and neutralized with 44 Na^+^ atoms. Before the production phase, the two solvated systems were minimized, heated up until 300 K and equilibrated into the final simulation conditions. The minimization was done in three consecutive steps, where the geometry of all hydrogens, all molecules except the solute, and the whole system, were allowed gradually to relax. The heating step was performed from 100 to 300 K in 500 ps at constant volume (NVT ensemble) using the Langevin thermostat (friction coefficient of 1 ps^-1^). During this step, the position of all solute atoms was constrained with a harmonic force of 40 kcal mol^-1^ Å^-2^. The resulting solutions were then equilibrated in 5 steps, where the constrains were gradually removed. In the last equilibration step, the system was equilibrated at constant pressure (NPT ensemble). The SHAKE algorithm was applied to all covalent bonds involving hydrogens and the electrostatic interactions in the long range were calculated using PME. Then, each system was further simulated for 500 ns per simulation without any constraint. For the *Sc*CreD/(*S*)-nitrosuccinate system, an additional simulation was done with the side chain of K308 deprotonated. Three independent simulations of 0.5 µs were run per system (total of 1.5 µs per system). The analysis of the trajectories was performed using cpptraj, the graphics for the MD simulations were generated with the open-source version of PyMOL, R, and Inkscape.

Figure S13.1: (**A**) Detail of fumarate (C-atoms shown in green) at the active site of ScCreD. (**B**) Distance distributions (Å) between (upper part) K308 NZ and the N310 ND2 nitrogen atoms with the carboxylate carbon C1 in fumarate; between OG of Ser303 and the carboxylate carbon C4; (lower part) between the R341’ NZ nitrogen, the S123 OG oxygen, and T122 OG1 oxygen with the carboxylate carbon C4 in fumarate. The distance distributions have been measured in the absence (-NO_2_^-^, blue surface) and presence (+NO_2_^-^, rosa surface) of nitrite. (**C**) Superimposition of different frames of the simulation of nitrite in site #2 along the MD simulation. One solution for the protein is shown for simplicity. The values consider the four active sites of ScCreD.

*Figure S13.2: (****A****) Detail of (S)-nitrosuccinate (C-atoms shown in green) at the active site of ScCreD. (****B****) Distance distributions (Å) between (upper part) K308 NZ and the N310 ND2 nitrogen atoms with the carboxylate carbon C1 in (S)-nitrosuccinate; between OG of Ser303 and the carboxylate carbon C4; (lower part) between the R341’ NZ nitrogen, the S123 OG oxygen, and T122 OG1 oxygen with the carboxylate carbon C4 in (S)-nitrosuccinate. The distance distributions have been measured for protonated (LYS, blue surface) and deprotonated (LYN, rosa surface) K308. (****C****) Distance distributions (Å) between K308 NZ and the nitrogen atom of the NO_2_ group and between the center-of-mass of the guanidinium group of R341’ and the NO_2_ group. All values consider the four active sites of ScCreD.*

## Quantum mechanics/molecular mechanics (QM/MM) MD simulations of *Sc*CreD

To shed light into the hydronitration reaction mechanism of fumarate by *Sc*CreD, initial exploration of the collective variables (CVs) of the reaction were done via steered QM/MM MD (sMD) simulations. First, the orientation of the substrates in the four active sites along the MD simulation trajectories were analyzed with the graphical interface of SmarTSzyme^[25]^ (<https://github.com/CAMDgraz/SmarTSzyme>). The distance between the OG atom of S302 and the protons HB31 and HB32 of (*S*)-nitrosuccinate was measured in all four sites for the three MD simulations (Figure S11.13). We selected both site #2 (44 frames) and the abstraction of H32 by S302 to compute the reaction mechanism using steered QM/MM MD simulations. The QM region consisted of (*S*)-nitrosuccinate and the side chains of S302, R341’ and K308, all of them from their CB atom (56 atoms in total). The QM region was treated with the density functional tight binding method (DFTB3)^[26]^ and the rest of the system with the amber force field amber19SB/OPC water model. The steered QM/MM MD simulations were run for 2 ps under the electrostatic embedding scheme (Figure 11.4). Two collective variables were defined: the enlarging of the bond between the nitro group and C2 (CV_1_) and the deprotonation of one proton on C3 by the side chain oxygen OG of Ser302 (CV_2_).

Figure 13.3: Distance distributions (Å) along the MD simulations between the protons H31 and H32 at C3 of (S)-nitrosuccinate and the oxygen atom OG of Ser302 in second active site.

Figure 13.4: sMD work values (kcal mol^-1^) vs time (ps) along the reaction mechanism of 44 frames from the MD simulation at active site #2.

## Nudged elastic band studies, geometry optimizations and calculation of frequencies

To get free energies for the reaction pathway, we selected a representative geometry from the former QM/MM MD simulations, and we run Nudged Elastic Band with TS optimization (NEB-TS)^[27]^ implemented in Orca 6.1.^[28]^ The same definition of the QM region as for the simulation of the previous QM/MM MD calculations was used (56 atoms). The system was treated with the one-parameter hybrid version of PBE, the DFT functional PBE0,^[29]^ including dispersion corrections D3^[30]^ and using the triple basis set def2-TZVP^[31]^ and an implicit solvent model for water with CPCM^[32]^. 20 images were traced along the reaction mechanism path. Intermediates and transition states identified along the path were taken for frequencies calculation. The initial, intermediate and final geometries were further optimized (only hydrogens) at the same level of theory (PBE0/def2-TZVP-D3/CPCM(water)). Analytical frequencies were calculated on the optimized geometries. The total thermal energy, including zero-point energy and vibrational, rotational and translational corrections, was calculated for each species (kcal mol^-1^). Only one negative frequency corresponding to the forming-breaking of the chemical bond in the transition state was identified. Other small additional negative frequencies below 100 cm^-1^ corresponding to bond rotations were obtained. The optimized geometries have been included into the data repository (<https://doi.org/10.5281/zenodo.18184253>).

# References

[1] Y. Sugai, Y. Katsuyama, Y. Ohnishi, *Nat. Chem. Biol.* **2016**, *12*, 73-75.

[2] R. Hagihara, Y. Katsuyama, Y. Sugai, H. Onaka, Y. Ohnishi, *J. Antibiot.* **2018**, *71*, 911-919.

[3] Z. Huang, K. A. Wang, W. A. van der Donk, *Chem. Sci.* **2016**, *7*, 5219-5223.

[4] X. Chen, J. Chen, W. Zhang, H. Wang, X. Liu, W. Zhou, H. Yang, Z. Rao, *Biochem. Biophys. Res. Commun.* **2019**, *510*, 116-121.

[5] V. Puthan Veetil, G. Fibriansah, H. Raj, A. M. Thunnissen, G. J. Poelarends, *Biochemistry* **2012**, *51*, 4237-4243.

[6] S. A. Woods, J. S. Miles, R. E. Roberts, J. R. Guest, *Biochem. J.* **1986**, *237*, 547-557.

[7] B. Van Laer, U. Kapp, M. Soler-Lopez, K. Moczulska, S. Paabo, G. Leonard, C. Mueller-Dieckmann, *Sci. Rep.* **2018**, *8*, 18008.

[8] J. Yang, Y. Wang, E. M. Woolridge, V. Arora, G. A. Petsko, J. W. Kozarich, D. Ringe, *Biochemistry* **2004**, *43*, 10424-10434.

[9] S. Kawai, Y. Sugaya, R. Hagihara, H. Tomita, Y. Katsuyama, Y. Ohnishi, *Angew. Chem. Int. Ed.* **2021**, *60*, 10319-10325.

[10] J. B. Patteson, A. T. Putz, L. Tao, W. C. Simke, L. H. Bryant, 3rd, R. D. Britt, B. Li, *Science* **2021**, *374*, 1005-1009.

[11] E. Tassano, K. Merusic, I. Buljubasic, O. Laggner, T. Reiter, A. Vogel, M. Hall, *Chem. Commun.* **2020**, *56*, 6340-6343.

[12] D. Tsikas, *J. Chromatogr. B* **2007**, *851*, 51-70.

[13] D. J. T. Porter, H. J. Bright, *J. Biol. Chem.* **1980**, *255*, 4772-4780.

[14] A. Micsonai, F. Wien, E. Bulyaki, J. Kun, E. Moussong, Y. H. Lee, Y. Goto, M. Refregiers, J. Kardos, *Nucleic Acids Res.* **2018**, *46*, W315-W322.

[15] N. J. Greenfield, *Nat. Protoc.* **2006**, *1*, 2876-2890.

[16] T. J. Dolinsky, J. E. Nielsen, J. A. McCammon, N. A. Baker, *Nucleic Acids Res.* **2004**, *32*, W665-W667.

[17] E. Chovancova, A. Pavelka, P. Benes, O. Strnad, J. Brezovsky, B. Kozlikova, A. Gora, V. Sustr, M. Klvana, P. Medek, L. Biedermannova, J. Sochor, J. Damborsky, *PLoS Comp. Biol.* **2012**, *8*, e1002708.

[18] A. Pavelka, E. Sebestova, B. Kozlikova, J. Brezovsky, J. Sochor, J. Damborsky, *IEEE/ACM Trans. Comput. Biol. Bioinform.* **2016**, *13*, 505-517.

[19] Q. Zhu, B. Meng, C. Gu, Y. Xu, J. Chen, C. Lei, X. Wu, *Org. Lett.* **2019**, *21*, 9985-9989.

[20] J. O. Hoberg, J. K. Addo, P. Teesdale-Spittle, *Synthesis* **2005**, *2005*, 1923-1925.

[21] S. Grimme, J. Antony, S. Ehrlich, H. Krieg, *J. Chem. Phys.* **2010**, *132*.

[22] M. J. Frisch, G. W. Trucks, H. B. Schlegel, G. E. Scuseria, M. A. Robb, J. R. Cheeseman, G. Scalmani, V. Barone, G. A. Petersson, H. Nakatsuji, X. Li, M. Caricato, A. V. Marenich, J. Bloino, B. G. Janesko, R. Gomperts, B. Mennucci, H. P. Hratchian, J. V. Ortiz, A. F. Izmaylov, J. L. Sonnenberg, Williams, F. Ding, F. Lipparini, F. Egidi, J. Goings, B. Peng, A. Petrone, T. Henderson, D. Ranasinghe, V. G. Zakrzewski, J. Gao, N. Rega, G. Zheng, W. Liang, M. Hada, M. Ehara, K. Toyota, R. Fukuda, J. Hasegawa, M. Ishida, T. Nakajima, Y. Honda, O. Kitao, H. Nakai, T. Vreven, K. Throssell, J. A. Montgomery Jr., J. E. Peralta, F. Ogliaro, M. J. Bearpark, J. J. Heyd, E. N. Brothers, K. N. Kudin, V. N. Staroverov, T. A. Keith, R. Kobayashi, J. Normand, K. Raghavachari, A. P. Rendell, J. C. Burant, S. S. Iyengar, J. Tomasi, M. Cossi, J. M. Millam, M. Klene, C. Adamo, R. Cammi, J. W. Ochterski, R. L. Martin, K. Morokuma, O. Farkas, J. B. Foresman, D. J. Fox, Wallingford, CT, **2016**.

[23] A. Jakalian, D. B. Jack, C. I. Bayly, *J. Comput. Chem.* **2002**, *23*, 1623-1641.

[24] S. Izadi, R. Anandakrishnan, A. V. Onufriev, *J. Phys. Chem. Lett.* **2014**, *5*, 3863-3871.

[25] D. Platero-Rochart, P. A. Sánchez-Murcia, *ChemRxiv* **2025**.

[26] M. Gaus, Q. Cui, M. Elstner, *J. Chem. Theory Comput.* **2012**, *7*, 931-948.

[27] V. Asgeirsson, B. O. Birgisson, R. Bjornsson, U. Becker, F. Neese, C. Riplinger, H. Jonsson, *J. Chem. Theory Comput.* **2021**, *17*, 4929-4945.

[28] F. Neese, *Wiley Interdisciplinary Reviews: Computational Molecular Science* **2025**, *15*.

[29] C. Adamo, V. Barone, *J. Chem. Phys.* **1999**, *110*, 6158-6170.

[30] S. Grimme, *J. Comput. Chem.* **2004**, *25*, 1463-1473.

[31] F. Weigend, R. Ahlrichs, *Phys. Chem. Chem. Phys.* **2005**, *7*, 3297-3305.

[32] A. V. Marenich, C. J. Cramer, D. G. Truhlar, *J. Phys. Chem. B* **2009**, *113*, 6378-6396.
